# Supplementary material for: Trends in cancer incidence in younger and older adults: an international comparative analysis
Source: medRxiv. 2025 Aug 6:2025.08.04.25332933. Preprint. [Version 1] doi: 10.1101/2025.08.04.25332933 (PMC12443049; doi:10.1101/2025.08.04.25332933)
Supplement: Supplement 1 [file media-1.pdf]

Supplemental Material: Table of Contents

Appendix Figures 1-13: Age-standardised incidence rates (ASR) per 100,000 for each cancer by age
Appendix Figure 14-26: Average annual percent change (AAPC) in cancer incidence (2003-2017) for each cancer by age, country & UN region
Appendix Table 1: Source of cancer incidence data by country in GLOBOCAN
Appendix Table 2: ICD-10 codes for cancers based on Globocan
Appendix Table 3: AAPC comparisons by age (Bayes False Discovery Probability)
Appendix Tables 4-16: Segment specific annual percentage change (APC) for cancer incidence trends from 2003 to 2017 by country
Appendix Tables 17-19: Summary statistics by cancer and UN region
Appendix Tables 20-21: Summary statistics by cancer and sex

Appendix Figure 1: Age-standardised incidence rates (ASR) per 100,000

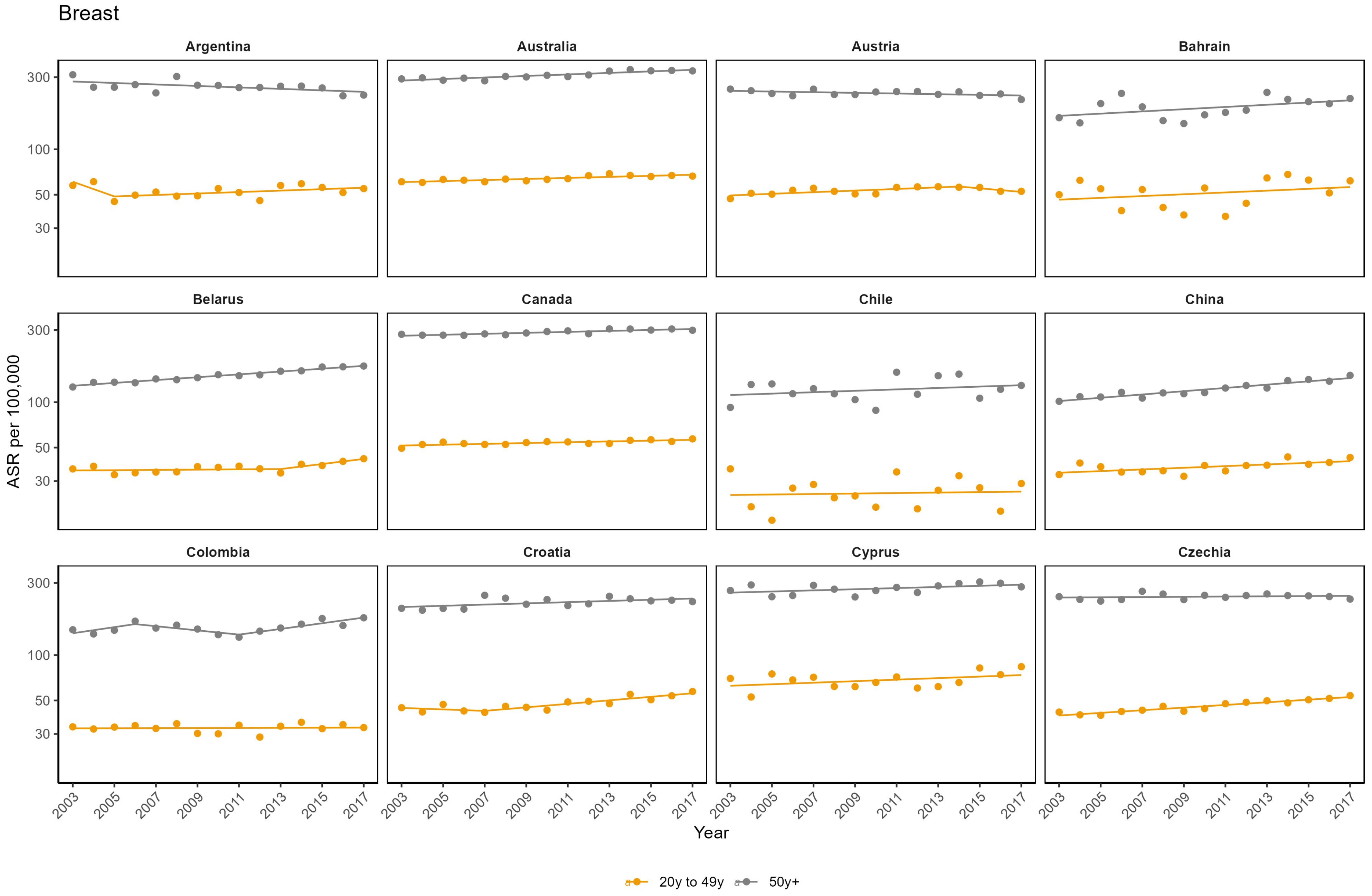

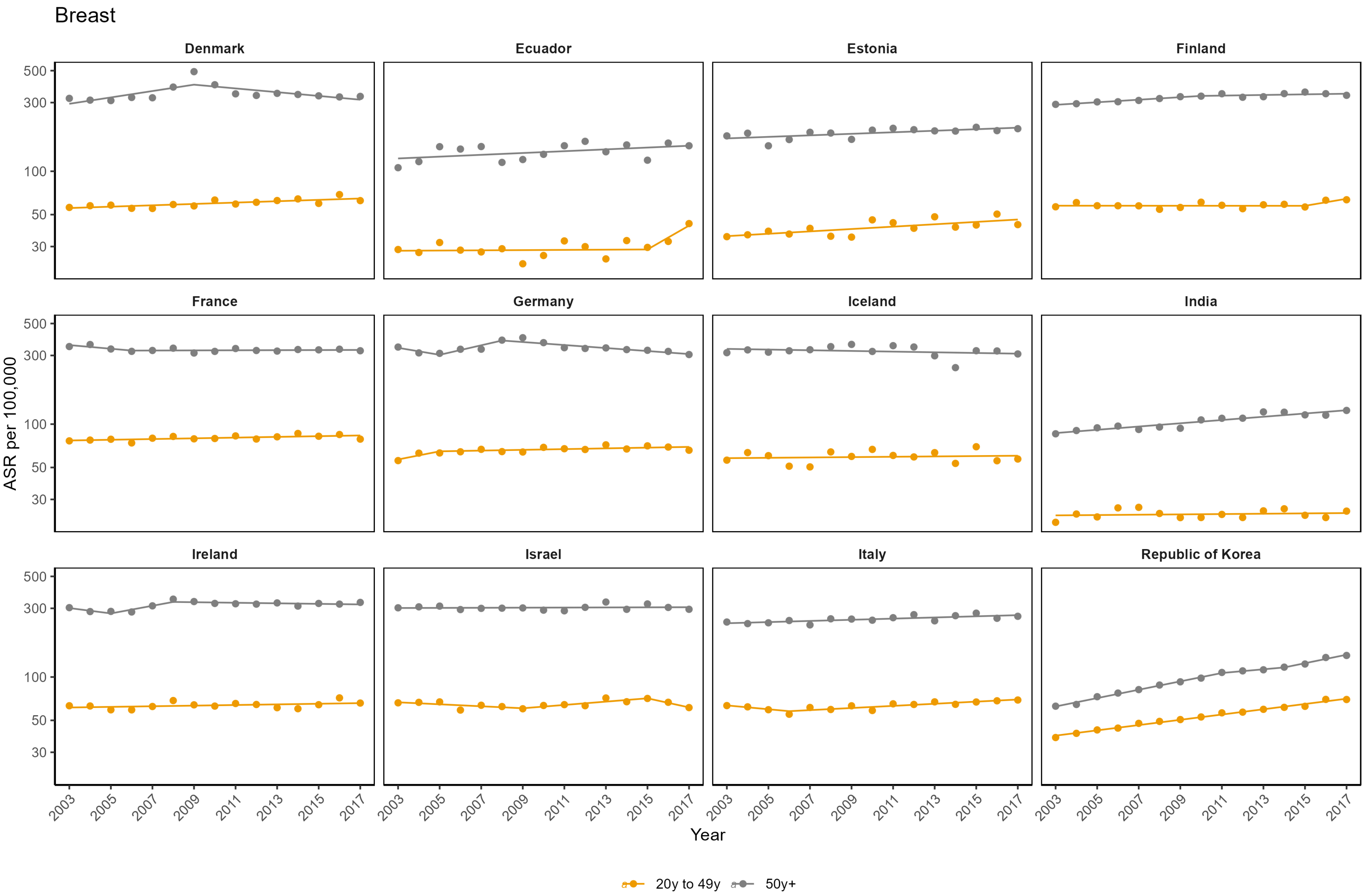

Breast
Denmark
Ecuador
Estonia
Finland
France
Germany
Iceland
India
Ireland
Israel
Italy
Republic of Korea
ASR per 100,000
500
300
100
50
30
2003
2005
2007
2009
2011
2013
2015
2017
Year
20y to 49y
50y+

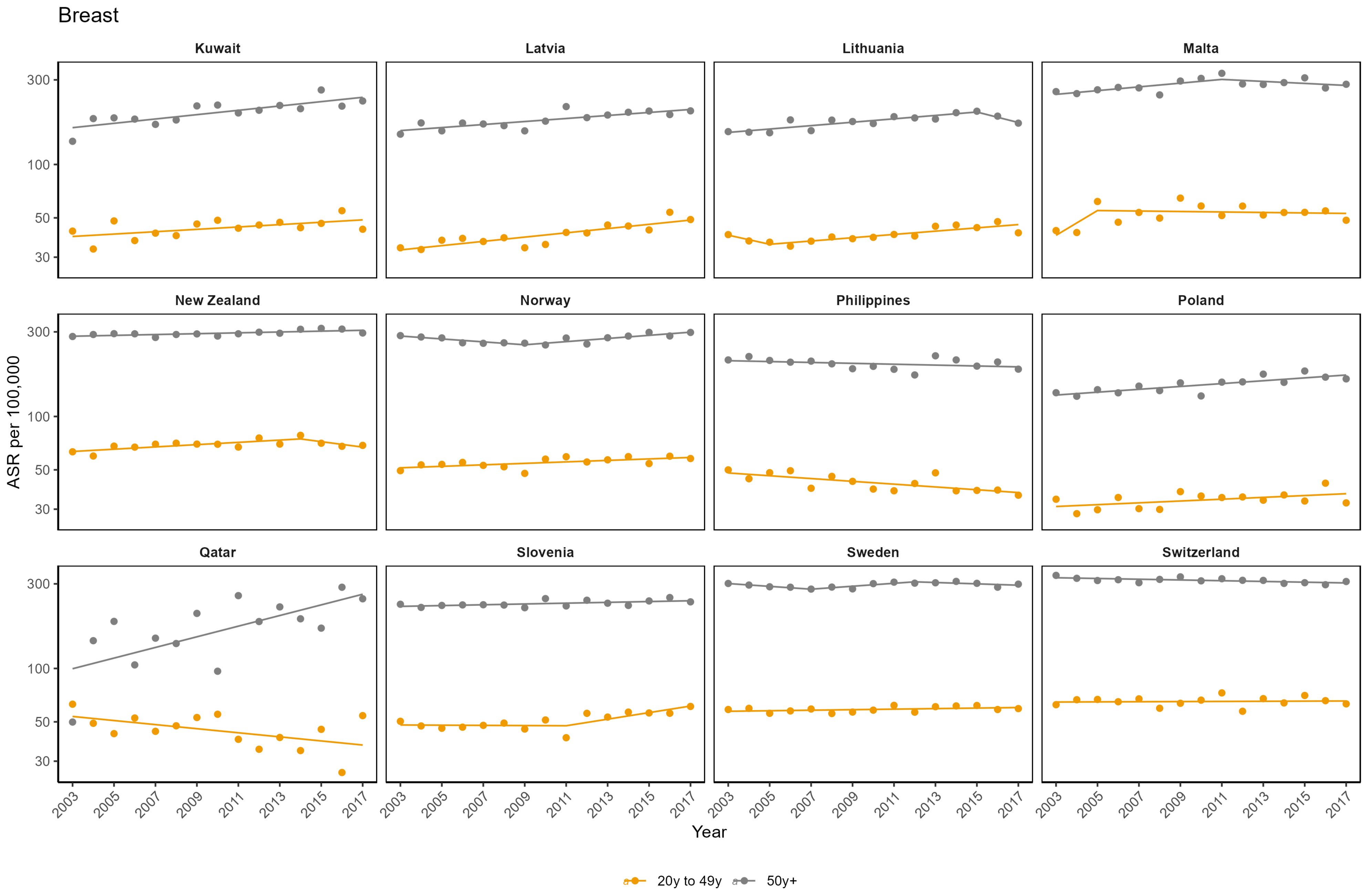

Breast
Kuwait
Latvia
Lithuania
Malta
New Zealand
Norway
Philippines
Poland
Qatar
Slovenia
Sweden
Switzerland
300
100
50
30
ASR per 100,000
2003
2005
2007
2009
2011
2013
2015
2017
Year
20y to 49y
50y+

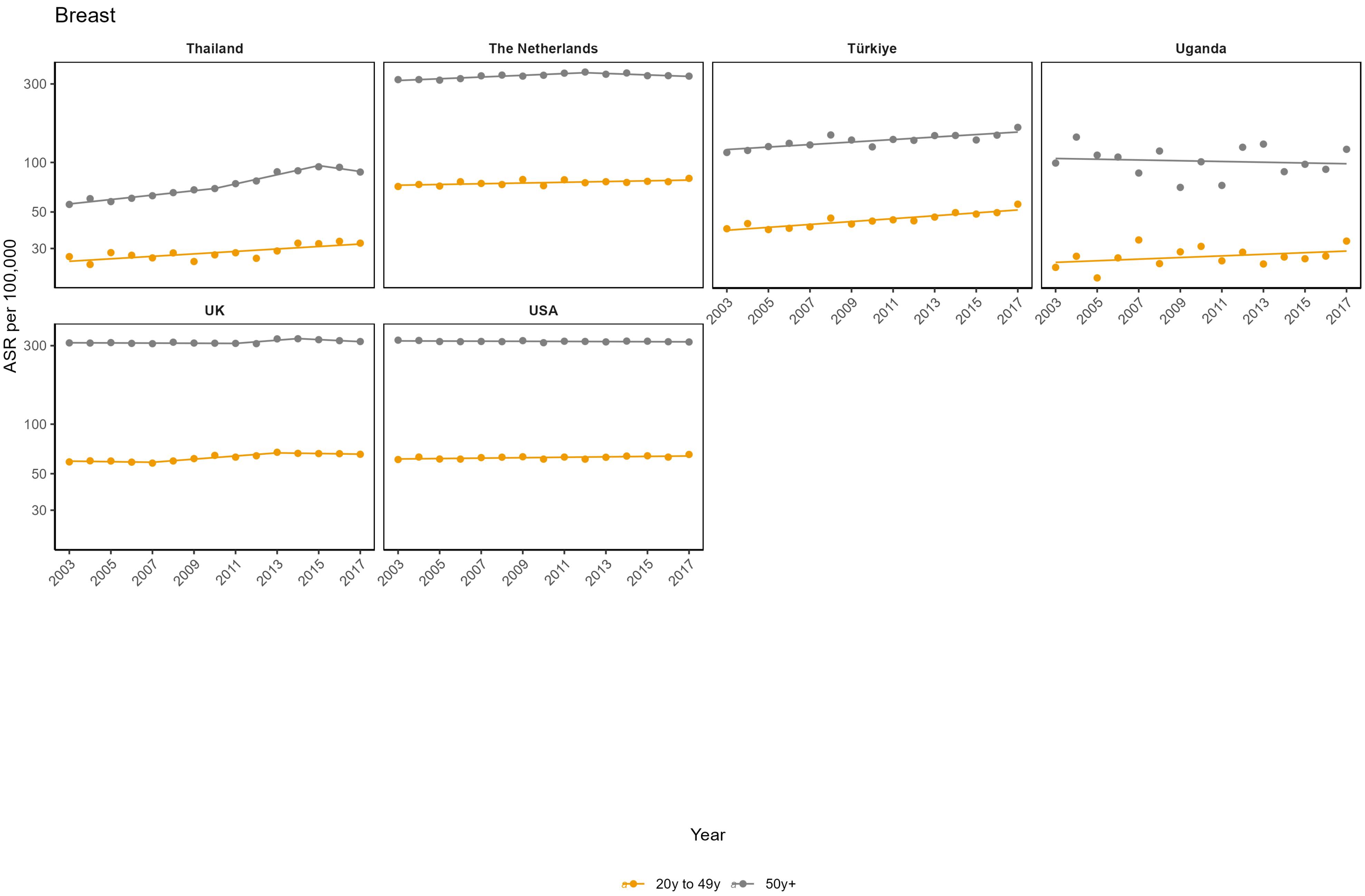
Breast
Thailand
The Netherlands
Türkiye
Uganda
UK
USA
ASR per 100,000
300
100
50
30
2003
2005
2007
2009
2011
2013
2015
2017
Year
20y to 49y
50y+

Appendix Figure 2: Age-standardised incidence rates (ASR) per 100,000

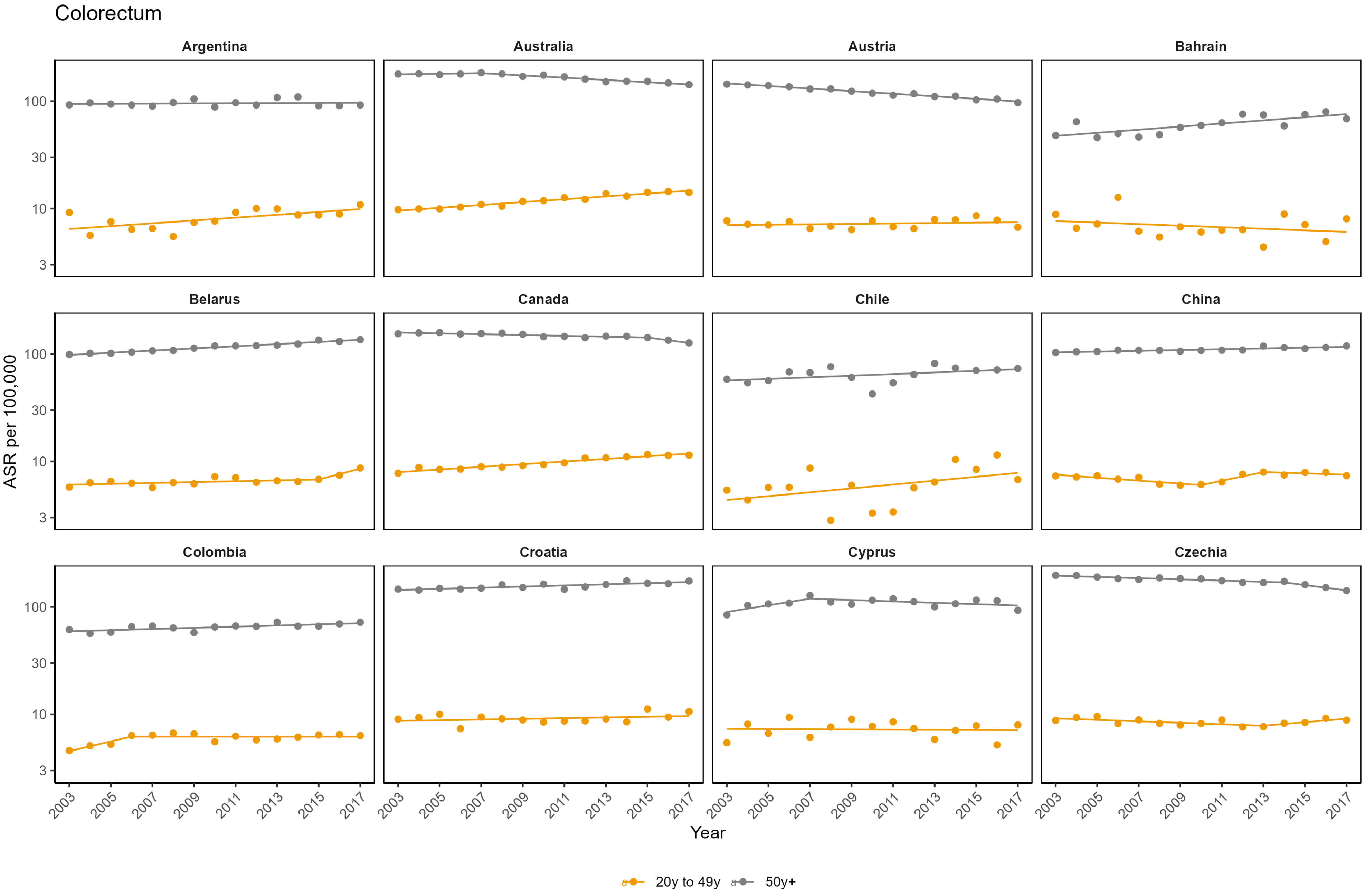

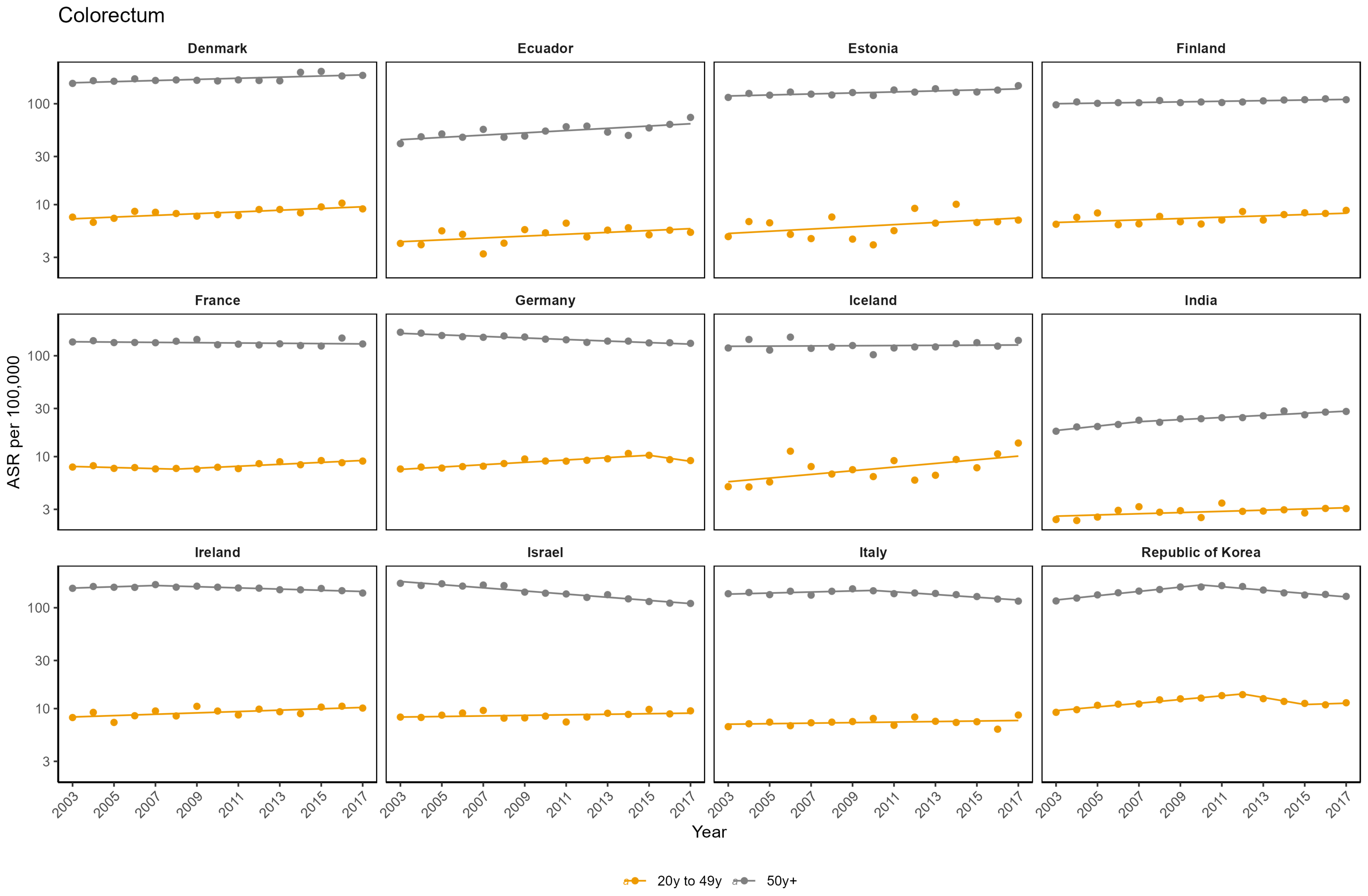

Colorectum
Denmark
Ecuador
Estonia
Finland
France
Germany
Iceland
India
Ireland
Israel
Italy
Republic of Korea
ASR per 100,000
100
30
10
3
2003
2005
2007
2009
2011
2013
2015
2017
Year
20y to 49y
50y+

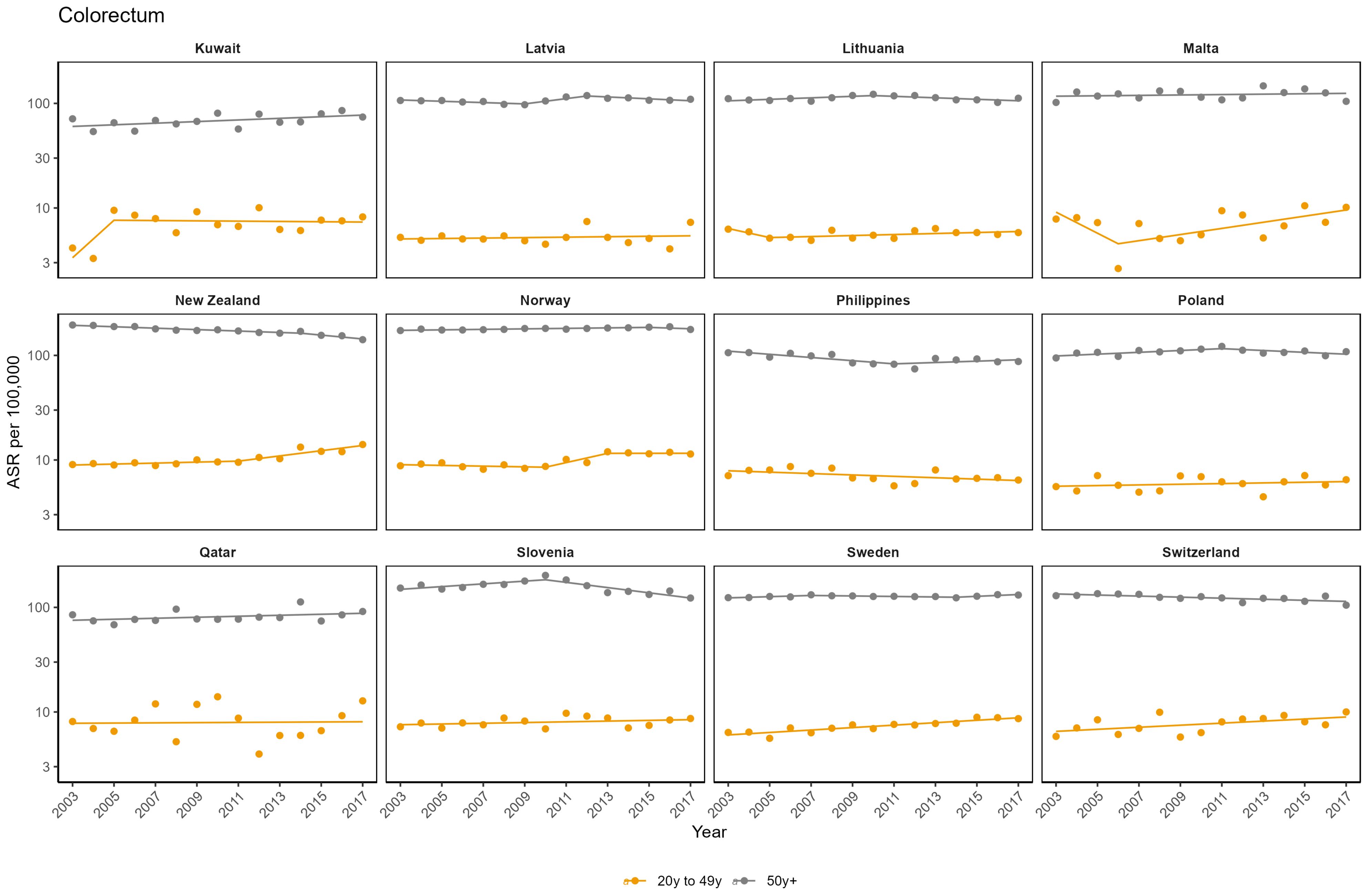

Colorectum
Kuwait
Latvia
Lithuania
Malta
New Zealand
Norway
Philippines
Poland
Qatar
Slovenia
Sweden
Switzerland
ASR per 100,000
100
30
10
3
2003
2005
2007
2009
2011
2013
2015
2017
Year
20y to 49y
50y+

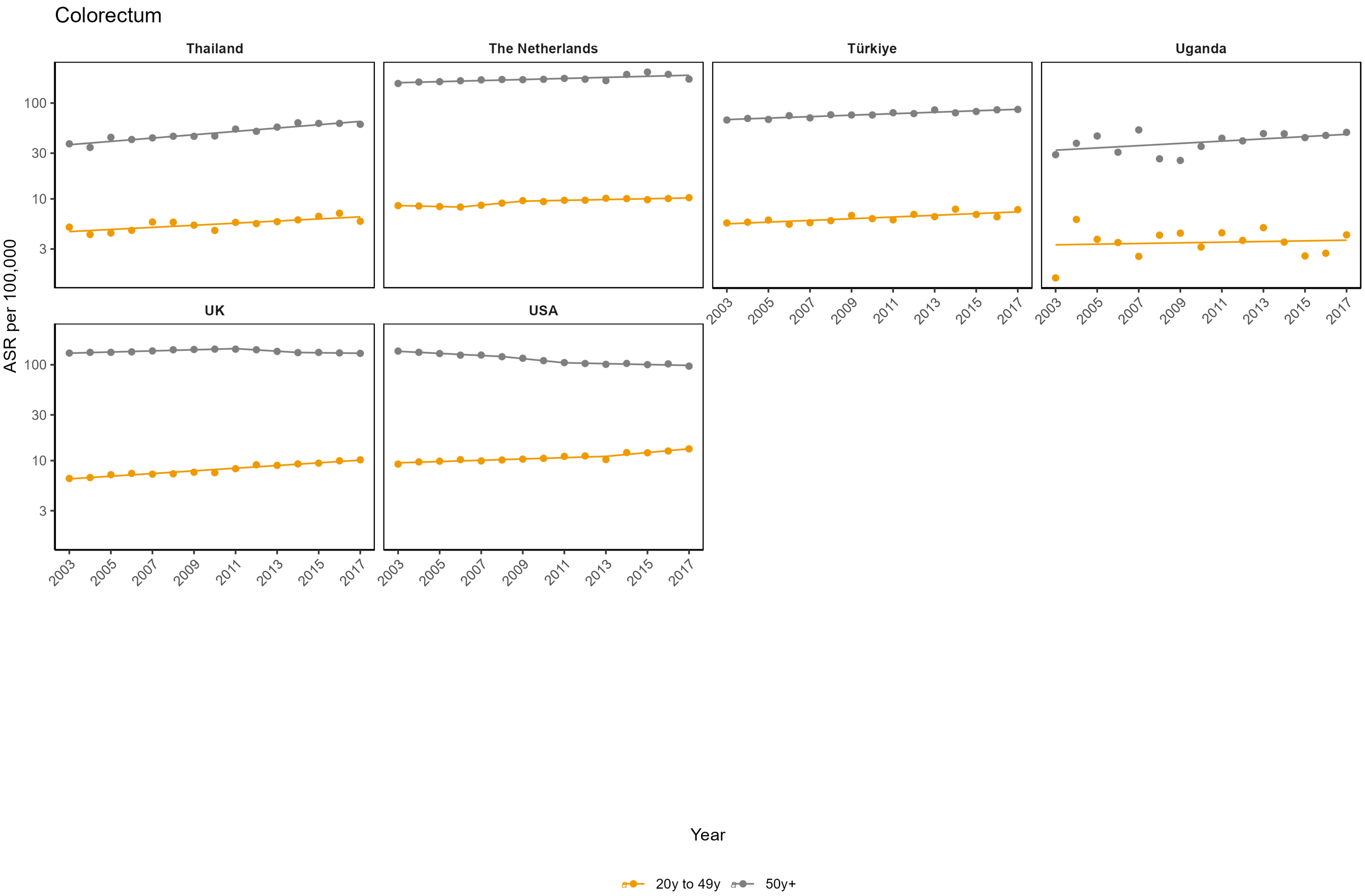
Colorectum
Thailand
The Netherlands
Türkiye
Uganda
UK
USA
ASR per 100,000
100
30
10
3
2003
2005
2007
2009
2011
2013
2015
2017
Year
20y to 49y
50y+

Appendix Figure 3: Age-standardised incidence rates (ASR) per 100,000

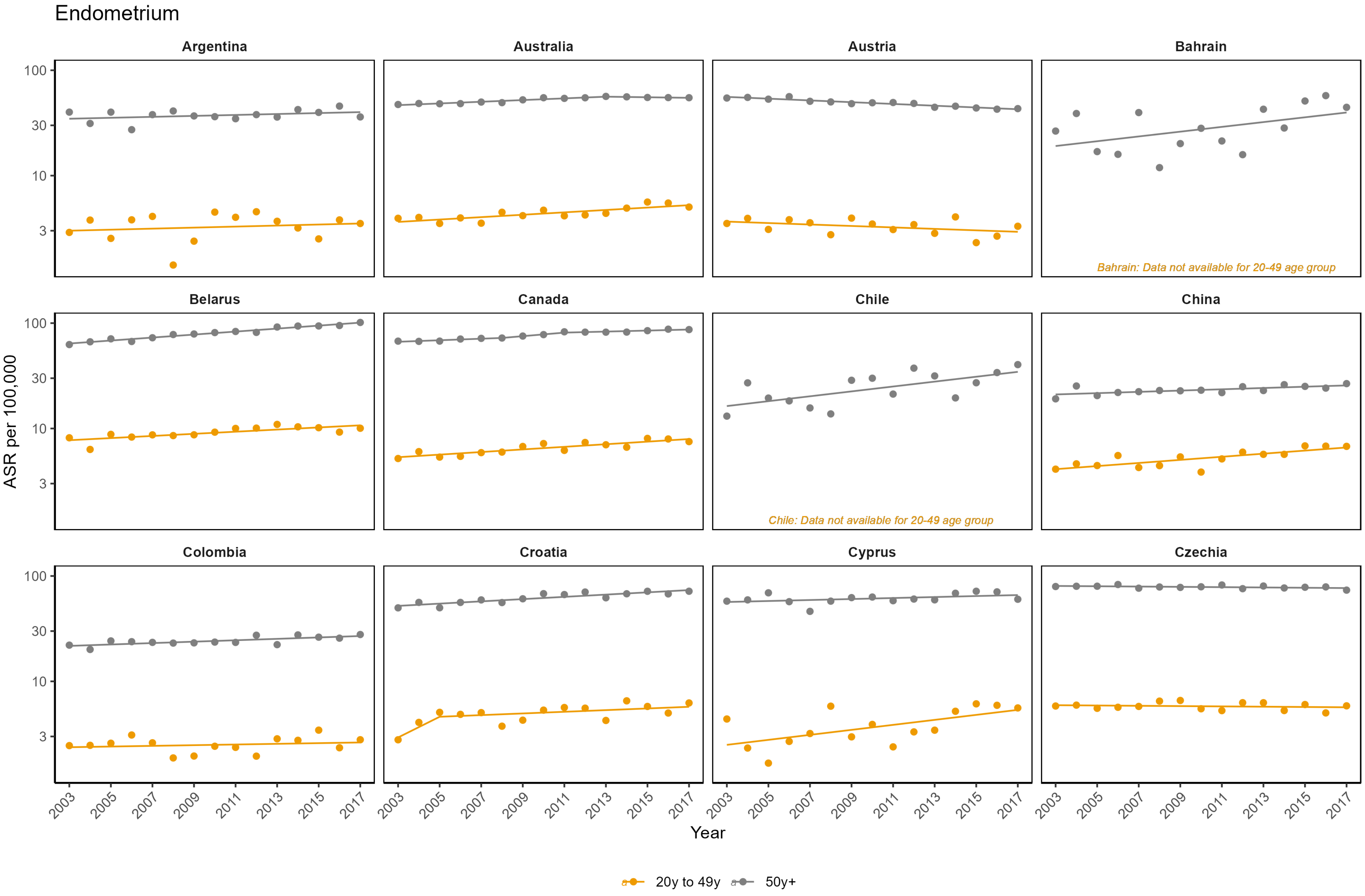

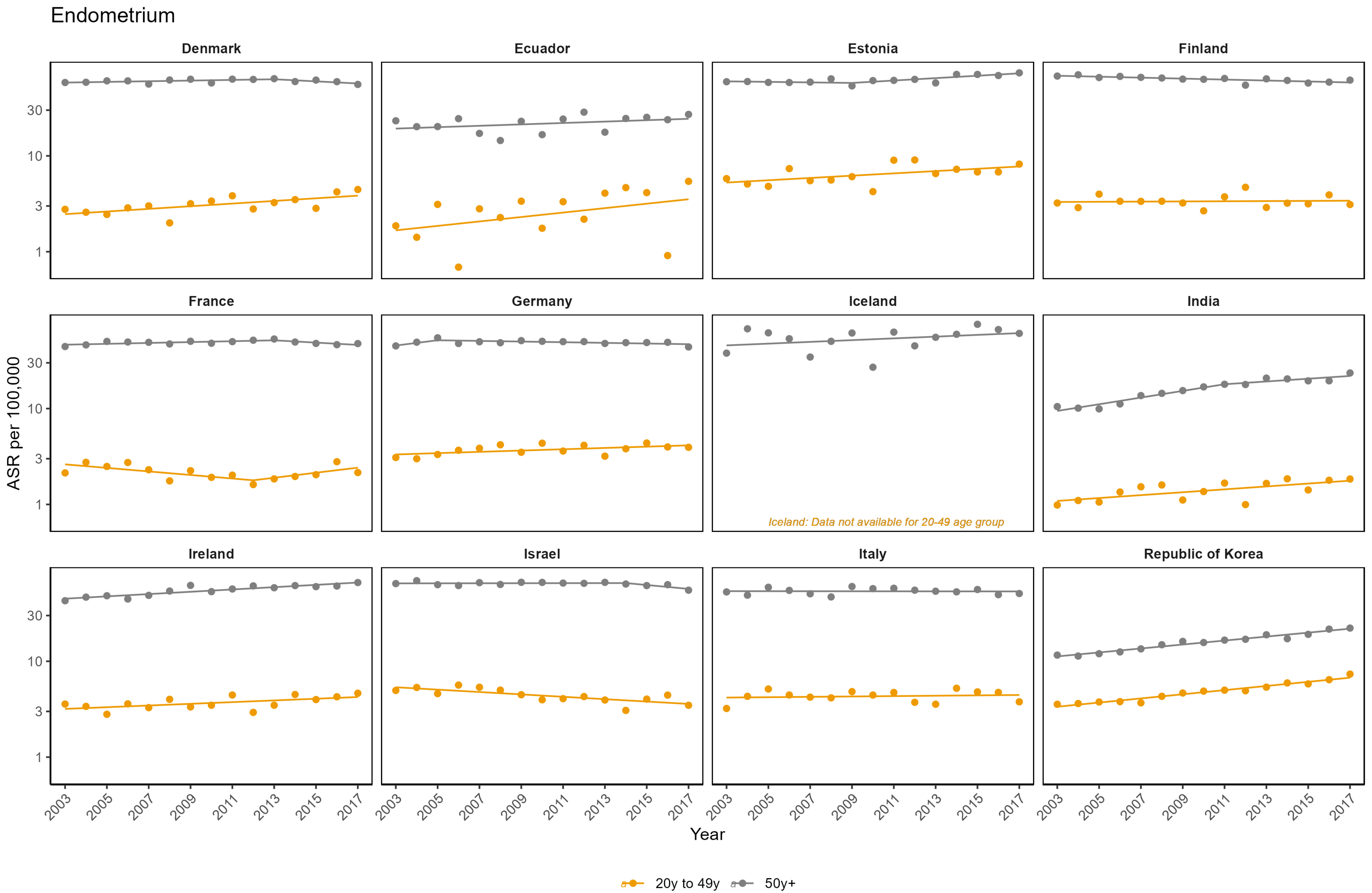

Endometrium
Denmark
Ecuador
Estonia
Finland
France
Germany
Iceland
India
Ireland
Israel
Italy
Republic of Korea
Iceland: Data not available for 20-49 age group
ASR per 100,000
Year
1
3
10
30
2003
2005
2007
2009
2011
2013
2015
2017
20y to 49y
50y+

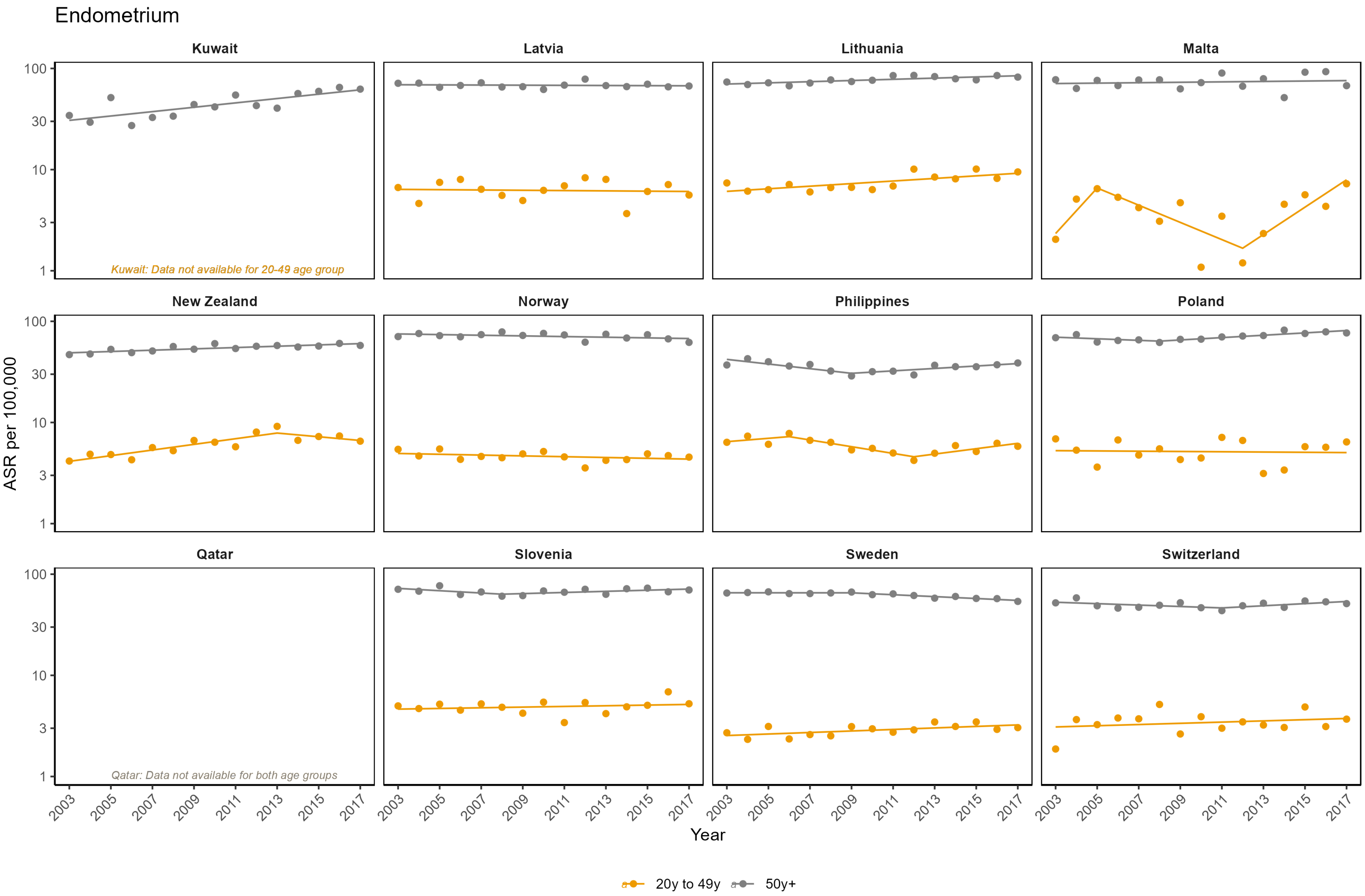
Endometrium
Kuwait
Latvia
Lithuania
Malta
New Zealand
Norway
Philippines
Poland
Qatar
Slovenia
Sweden
Switzerland
Kuwait: Data not available for 20-49 age group
Qatar: Data not available for both age groups
ASR per 100,000
Year
20y to 49y
50y+

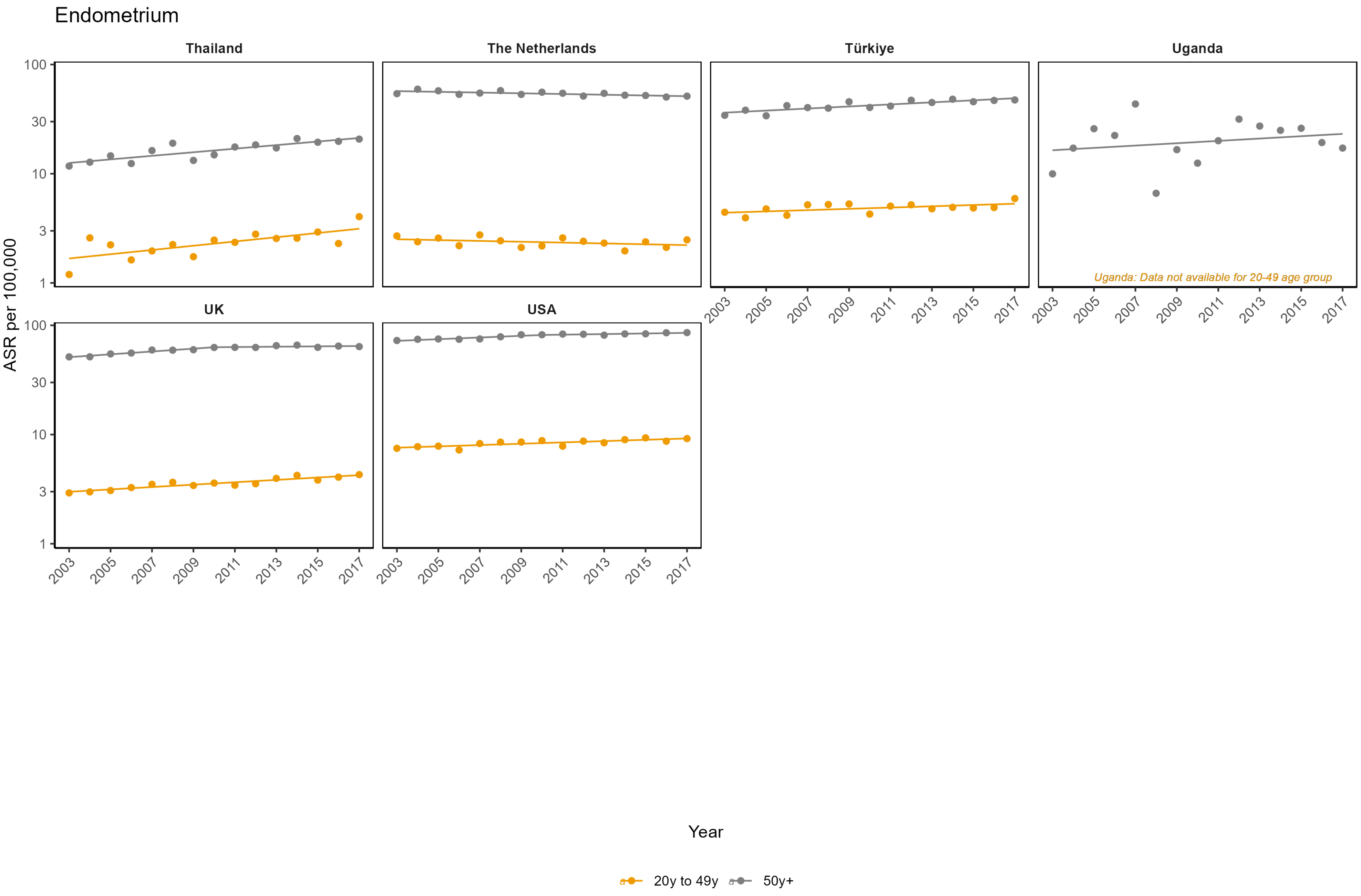

Endometrium
Thailand
The Netherlands
Türkiye
Uganda
UK
USA
Uganda: Data not available for 20-49 age group
ASR per 100,000
100
30
10
3
1
2003
2005
2007
2009
2011
2013
2015
2017
Year
20y to 49y
50y+

Appendix Figure 4: Age-standardised incidence rates (ASR) per 100,000

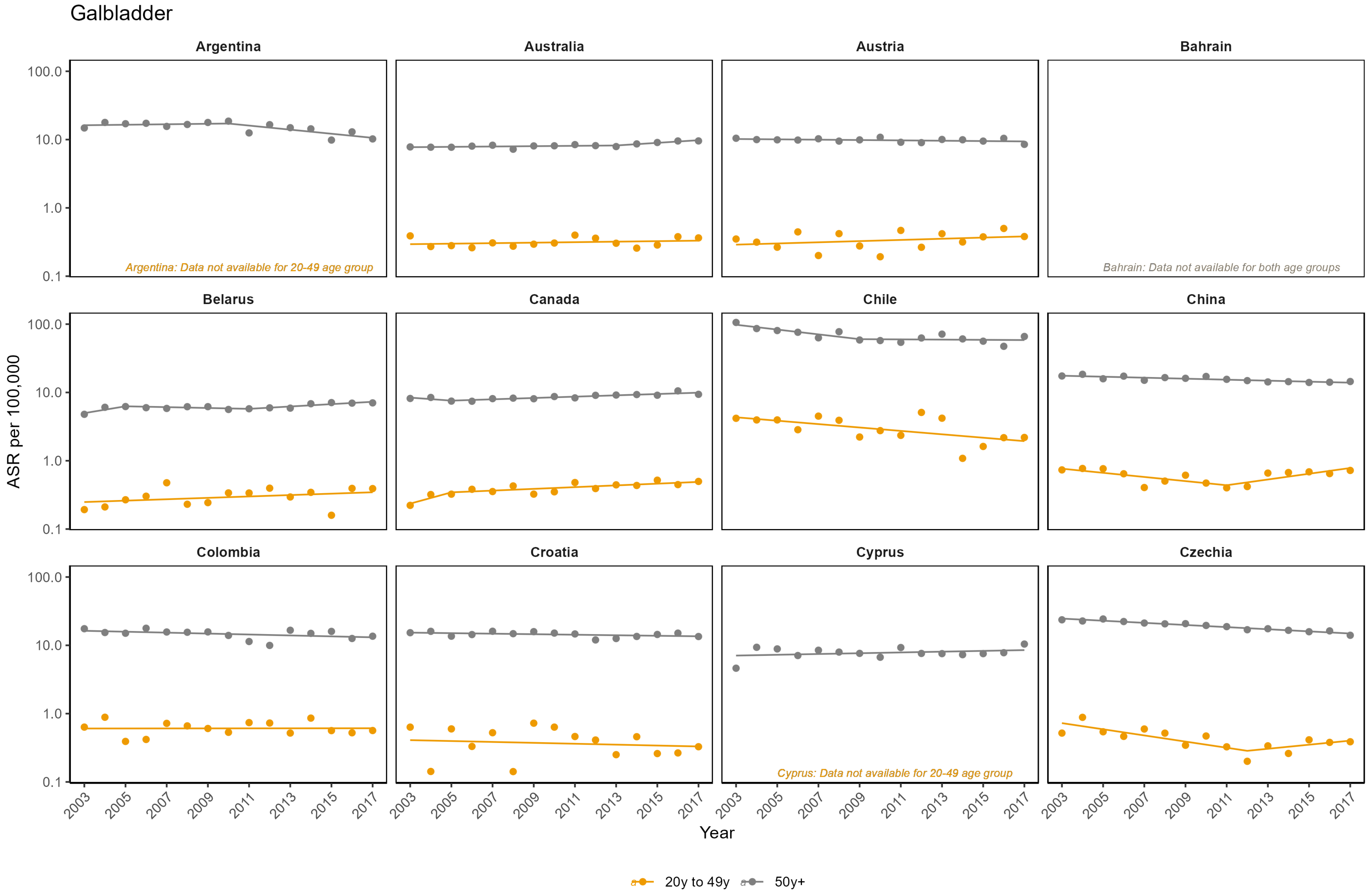

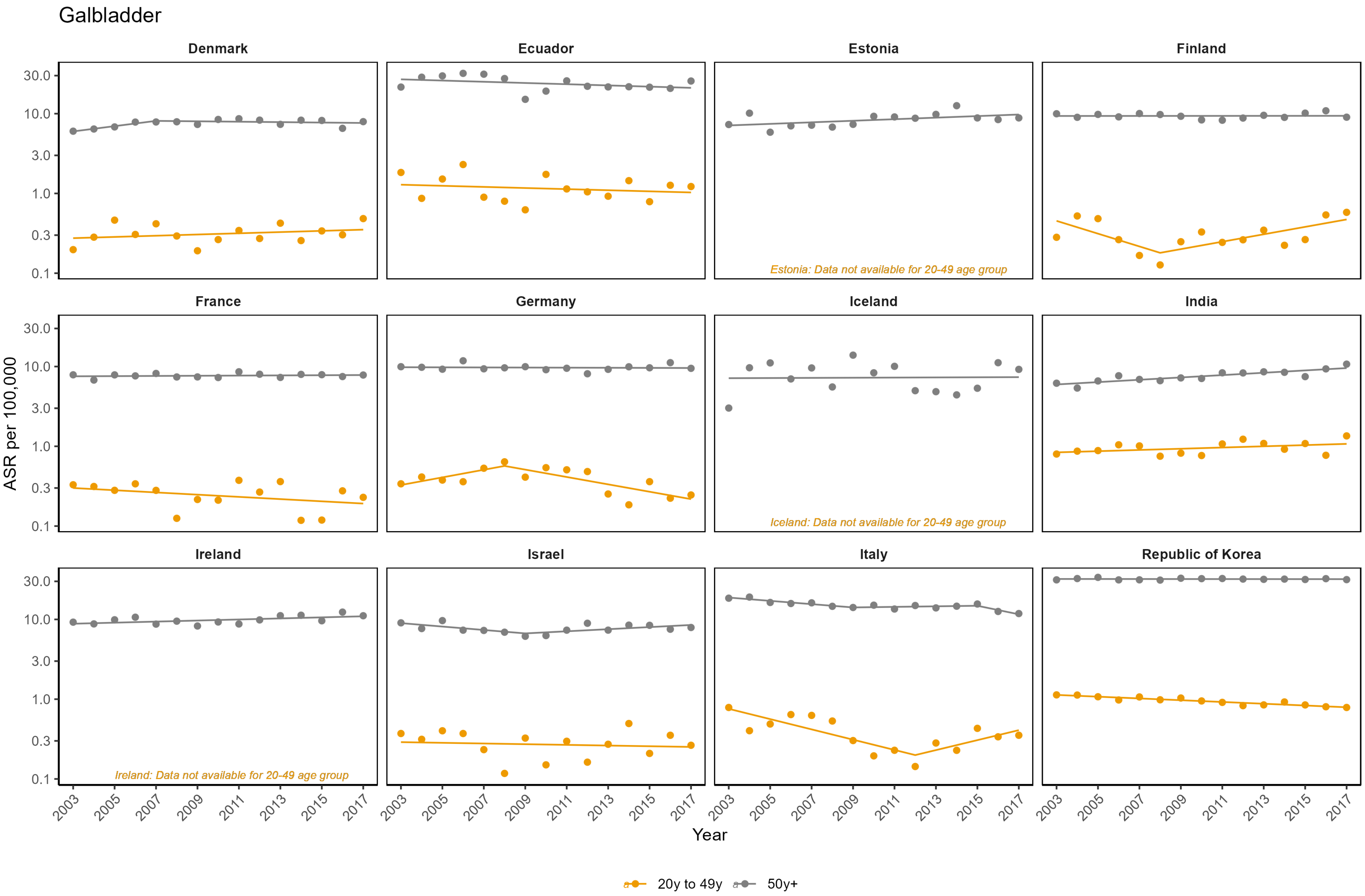
Galbladder
Denmark
Ecuador
Estonia
Finland
France
Germany
Iceland
India
Ireland
Israel
Italy
Republic of Korea
Estonia: Data not available for 20-49 age group
Iceland: Data not available for 20-49 age group
Ireland: Data not available for 20-49 age group
ASR per 100,000
Year
20y to 49y
50y+

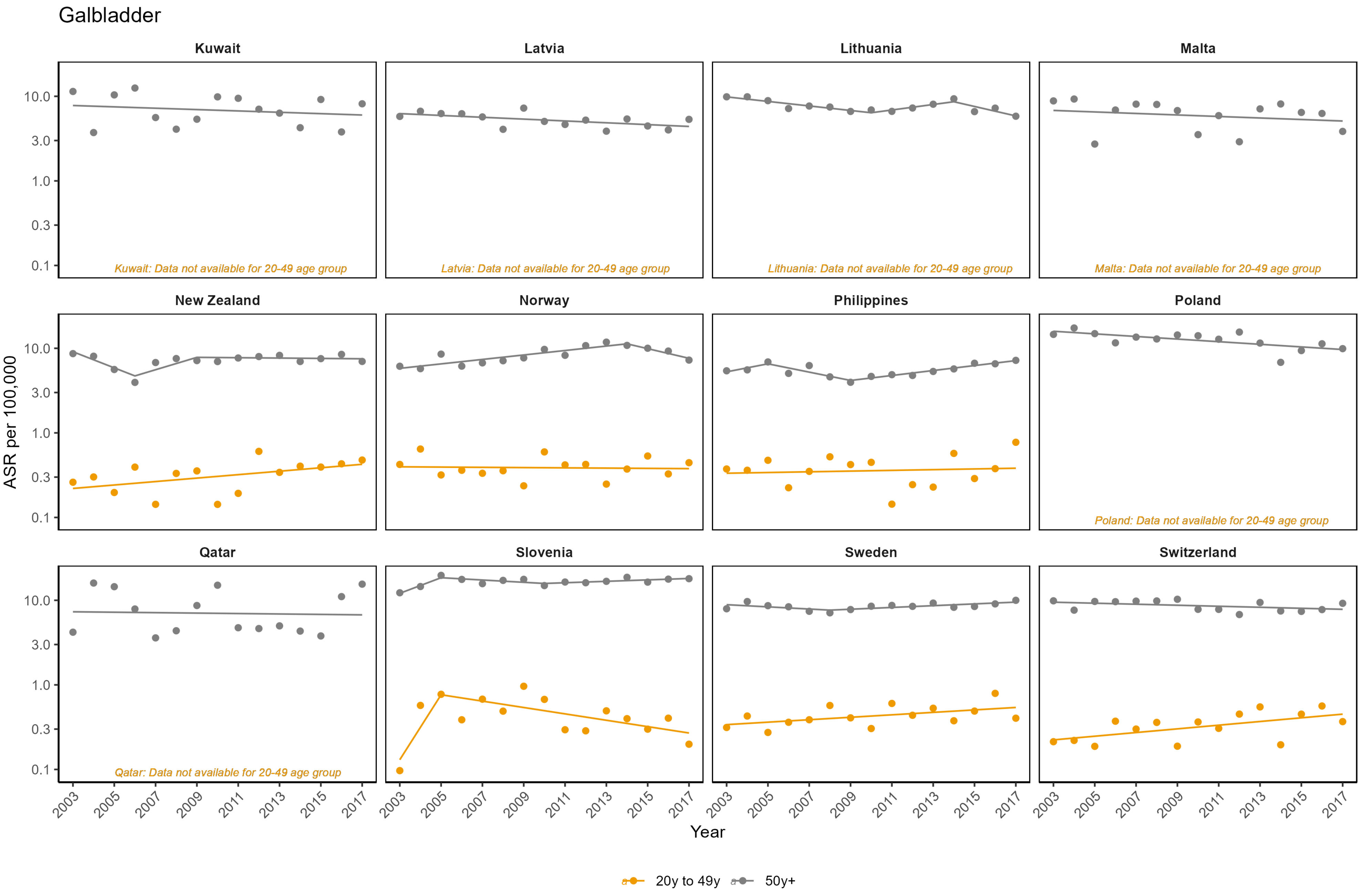
Galbladder
Kuwait
Latvia
Lithuania
Malta
New Zealand
Norway
Philippines
Poland
Qatar
Slovenia
Sweden
Switzerland
Kuwait: Data not available for 20-49 age group
Latvia: Data not available for 20-49 age group
Lithuania: Data not available for 20-49 age group
Malta: Data not available for 20-49 age group
Poland: Data not available for 20-49 age group
Qatar: Data not available for 20-49 age group
ASR per 100,000
10.0
3.0
1.0
0.3
0.1
2003
2005
2007
2009
2011
2013
2015
2017
Year
20y to 49y
50y+

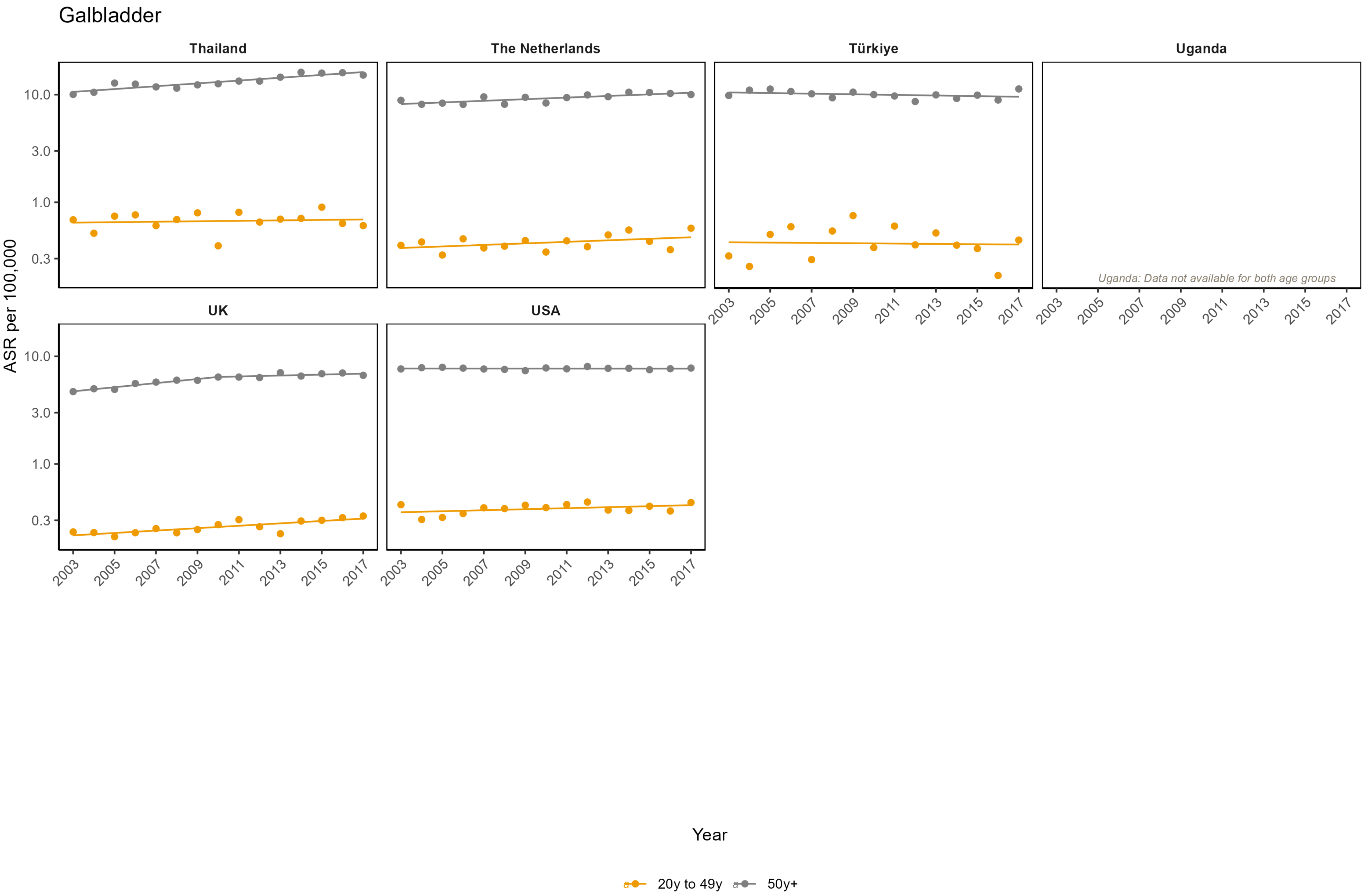
Galbladder
Thailand
The Netherlands
Türkiye
Uganda
UK
USA
Uganda: Data not available for both age groups
ASR per 100,000
10.0
3.0
1.0
0.3
2003
2005
2007
2009
2011
2013
2015
2017
Year
20y to 49y
50y+

Appendix Figure 5: Age-standardised incidence rates (ASR) per 100,000

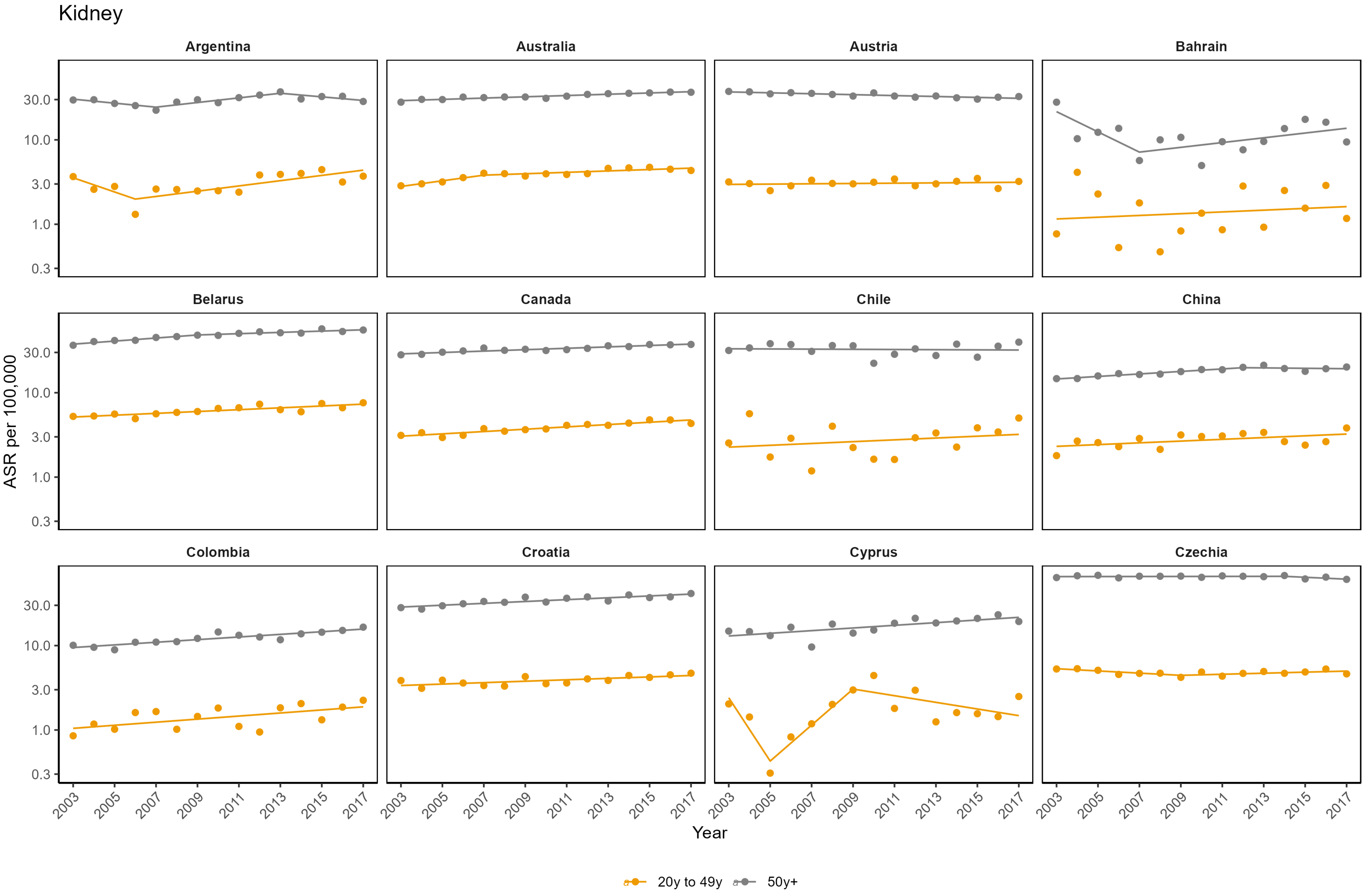

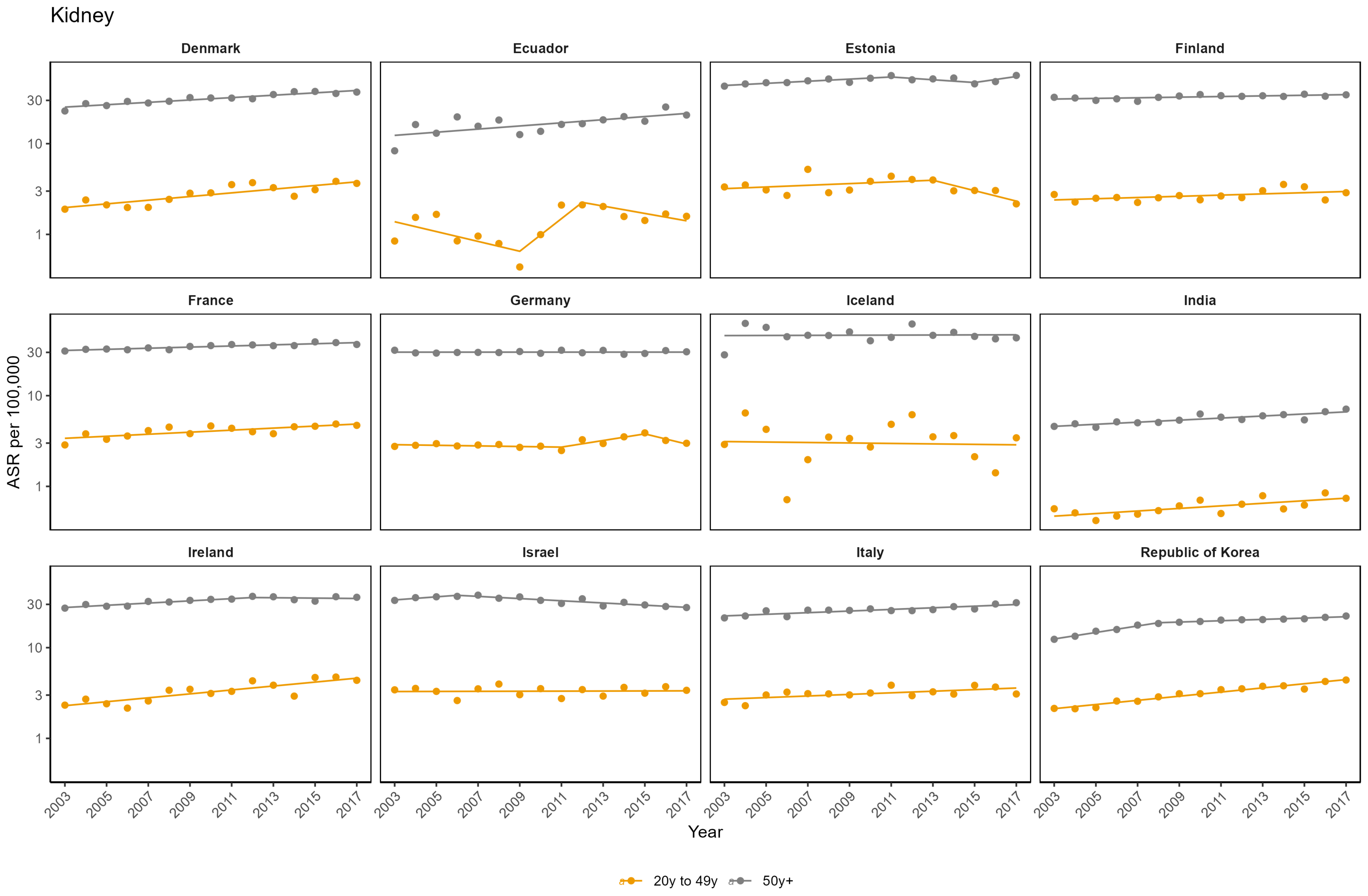

Kidney
Denmark
Ecuador
Estonia
Finland
France
Germany
Iceland
India
Ireland
Israel
Italy
Republic of Korea
ASR per 100,000
30
10
3
1
2003
2005
2007
2009
2011
2013
2015
2017
Year
20y to 49y
50y+

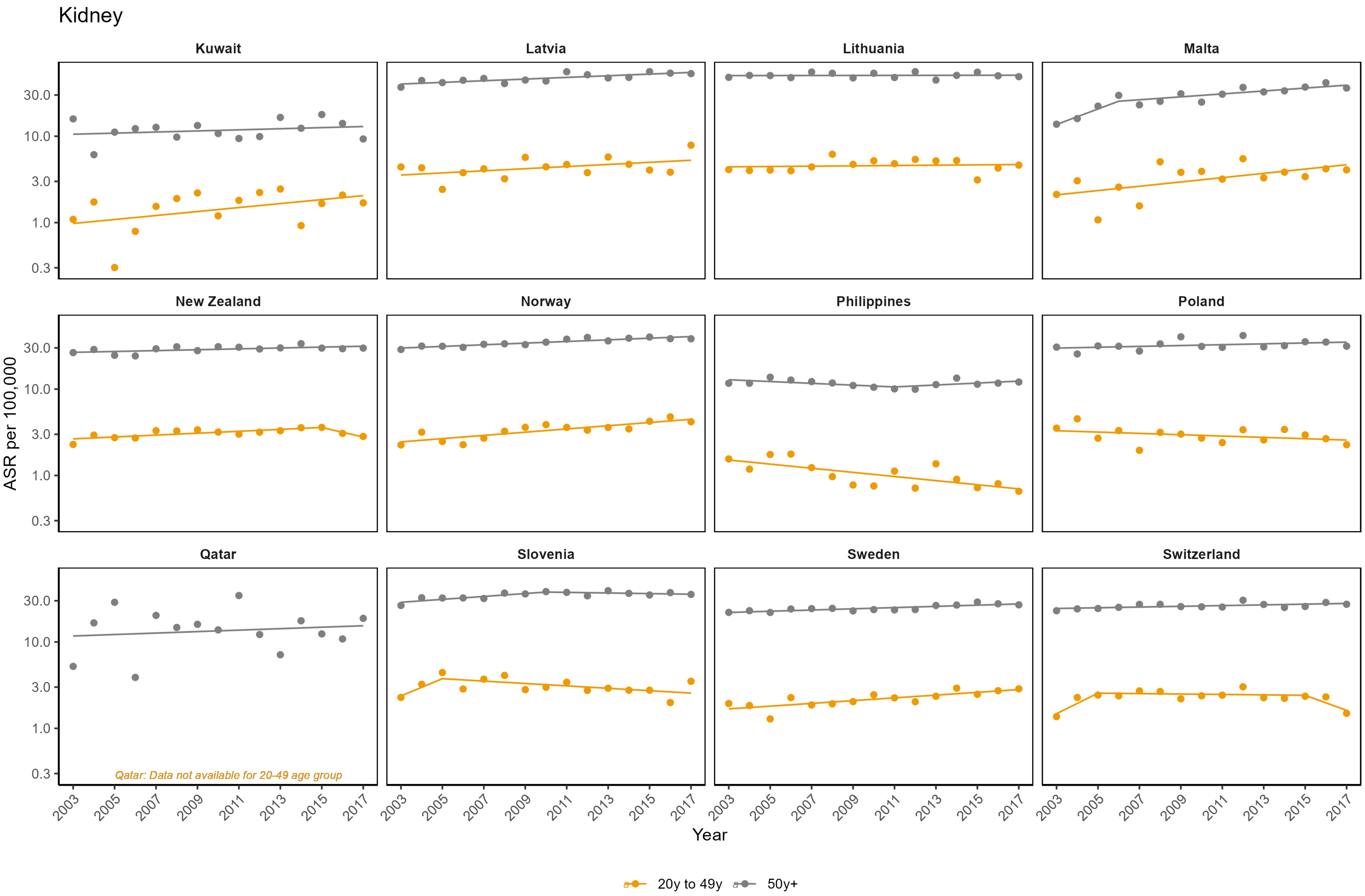
Kidney
Kuwait
Latvia
Lithuania
Malta
New Zealand
Norway
Philippines
Poland
Qatar
Slovenia
Sweden
Switzerland
ASR per 100,000
30.0
10.0
3.0
1.0
0.3
Qatar: Data not available for 20-49 age group
2003
2005
2007
2009
2011
2013
2015
2017
Year
20y to 49y
50y+

Kidney

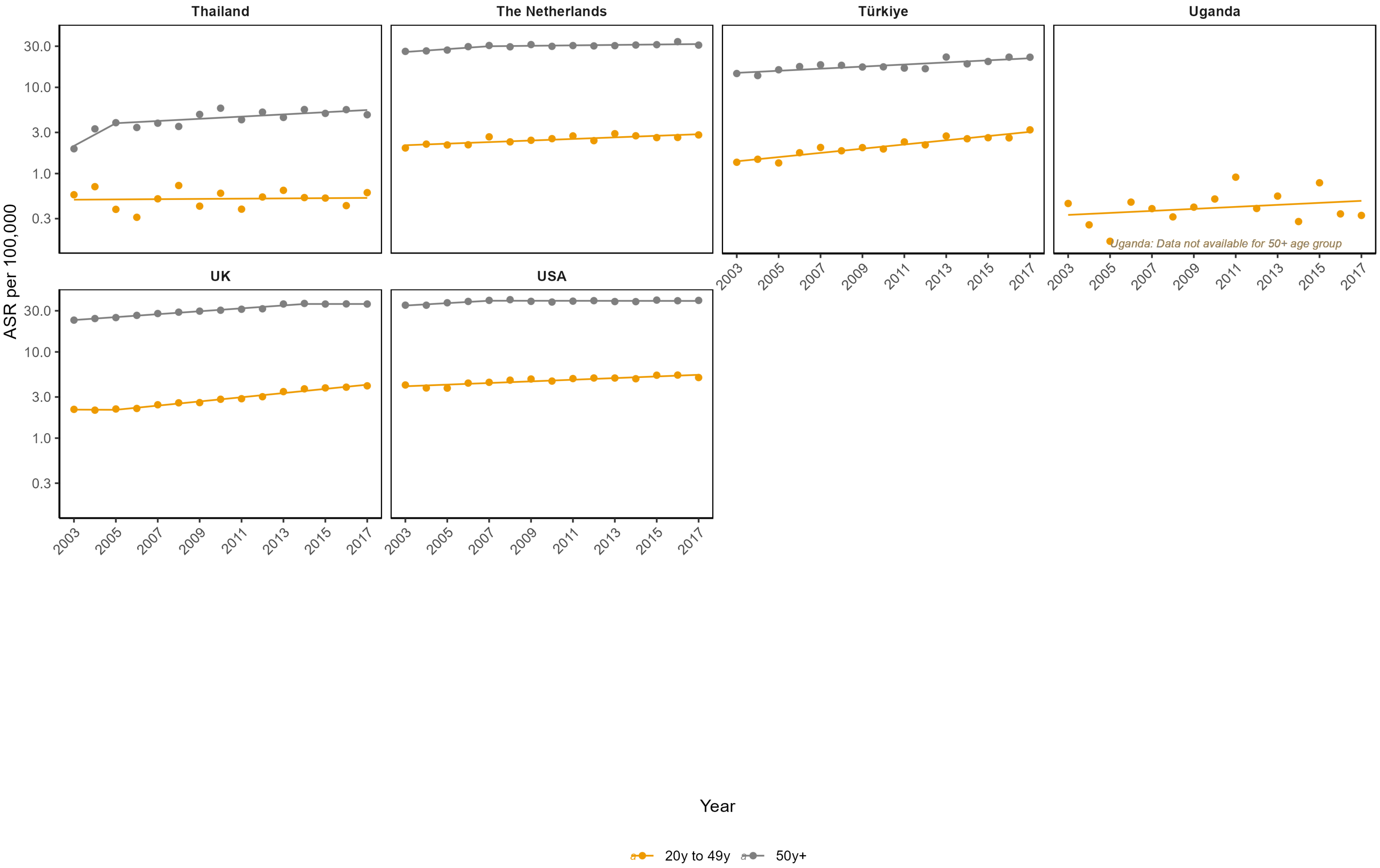
Thailand
The Netherlands
Türkiye
Uganda
UK
USA
ASR per 100,000
30.0
10.0
3.0
1.0
0.3
2003
2005
2007
2009
2011
2013
2015
2017
Uganda: Data not available for 50+ age group
Year
20y to 49y
50y+

Appendix Figure 6: Age-standardised incidence rates (ASR) per 100,000

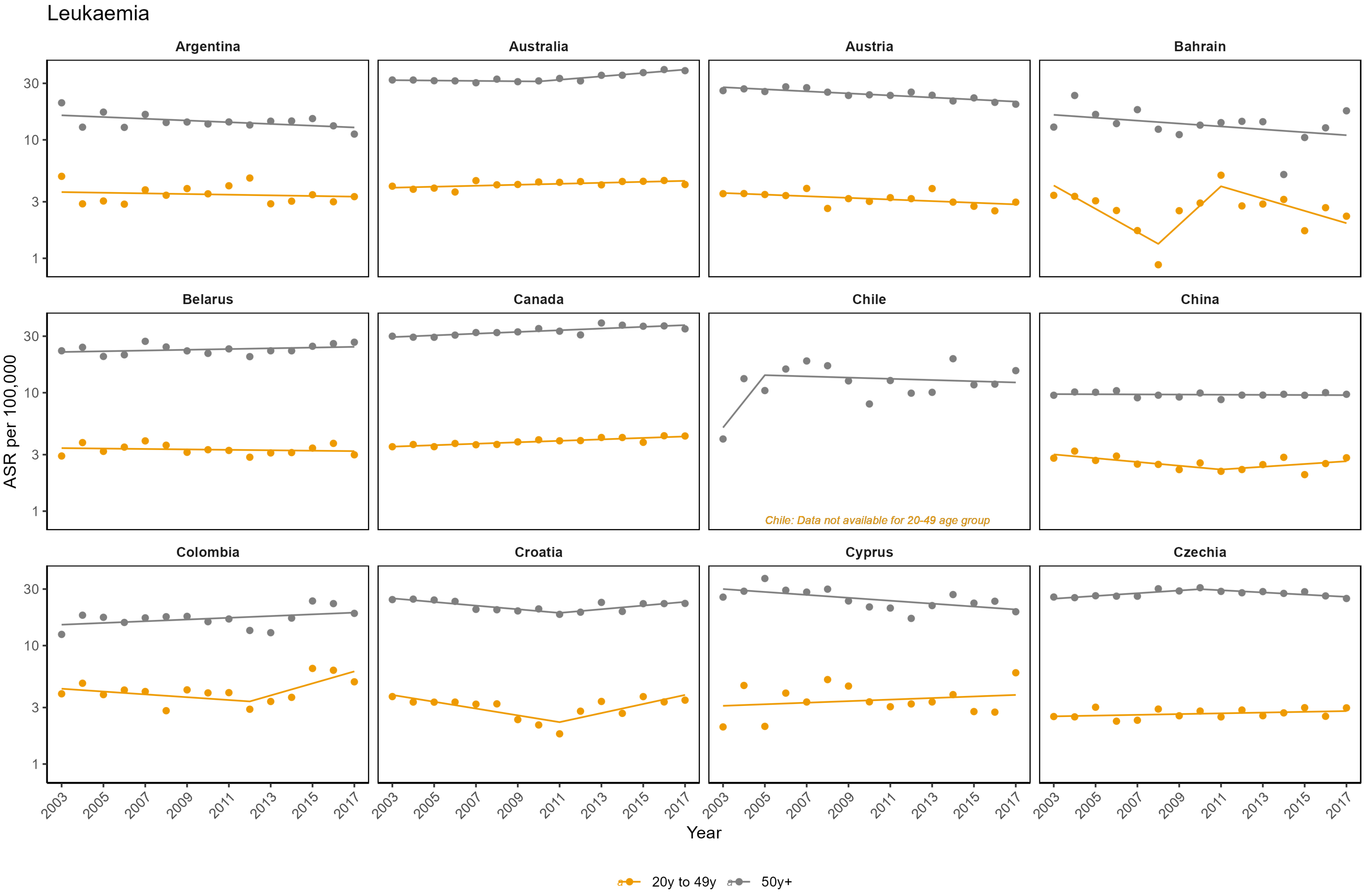

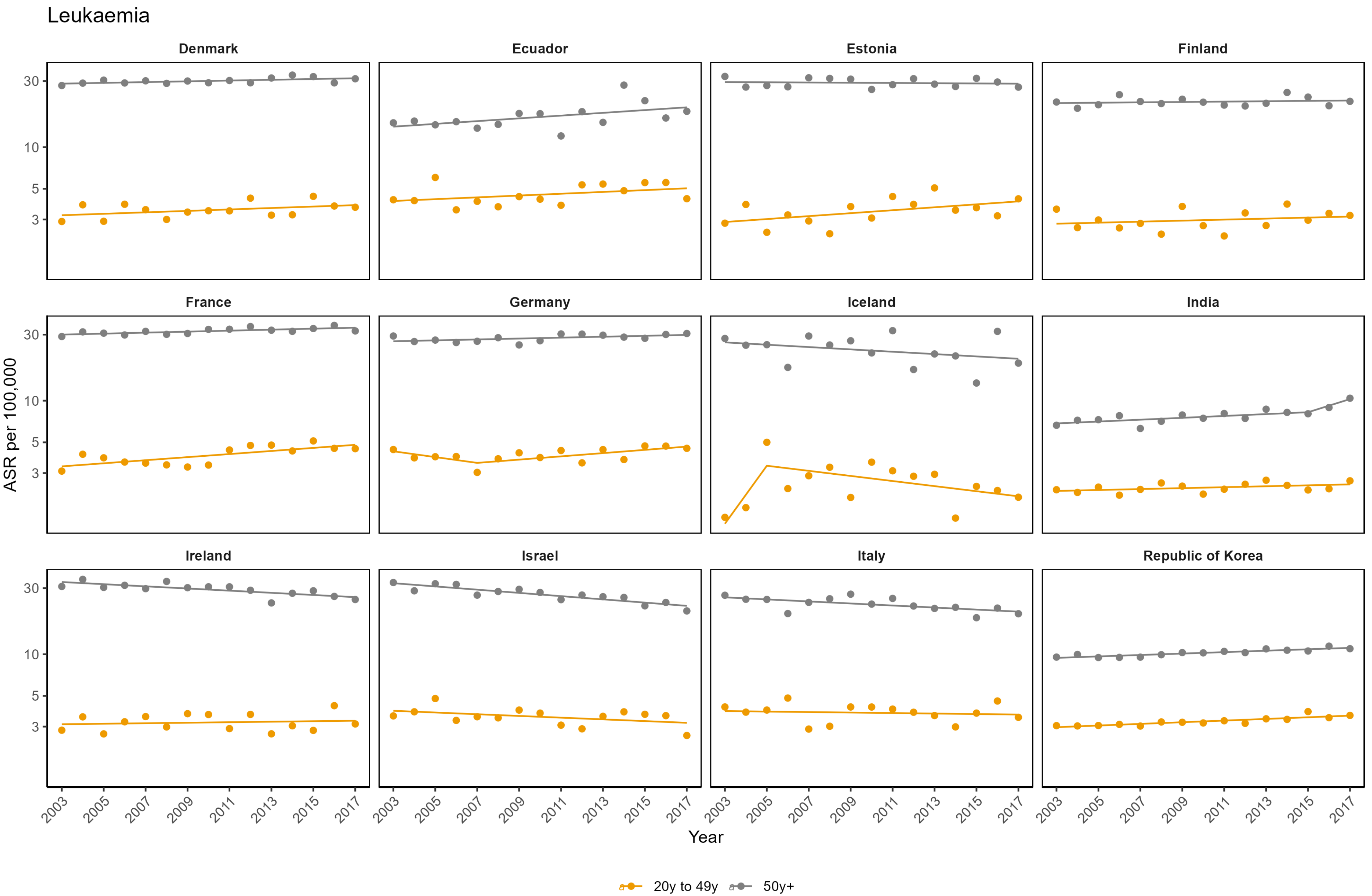

Leukaemia
Denmark
Ecuador
Estonia
Finland
France
Germany
Iceland
India
Ireland
Israel
Italy
Republic of Korea
ASR per 100,000
30
10
5
3
2003
2005
2007
2009
2011
2013
2015
2017
Year
20y to 49y
50y+

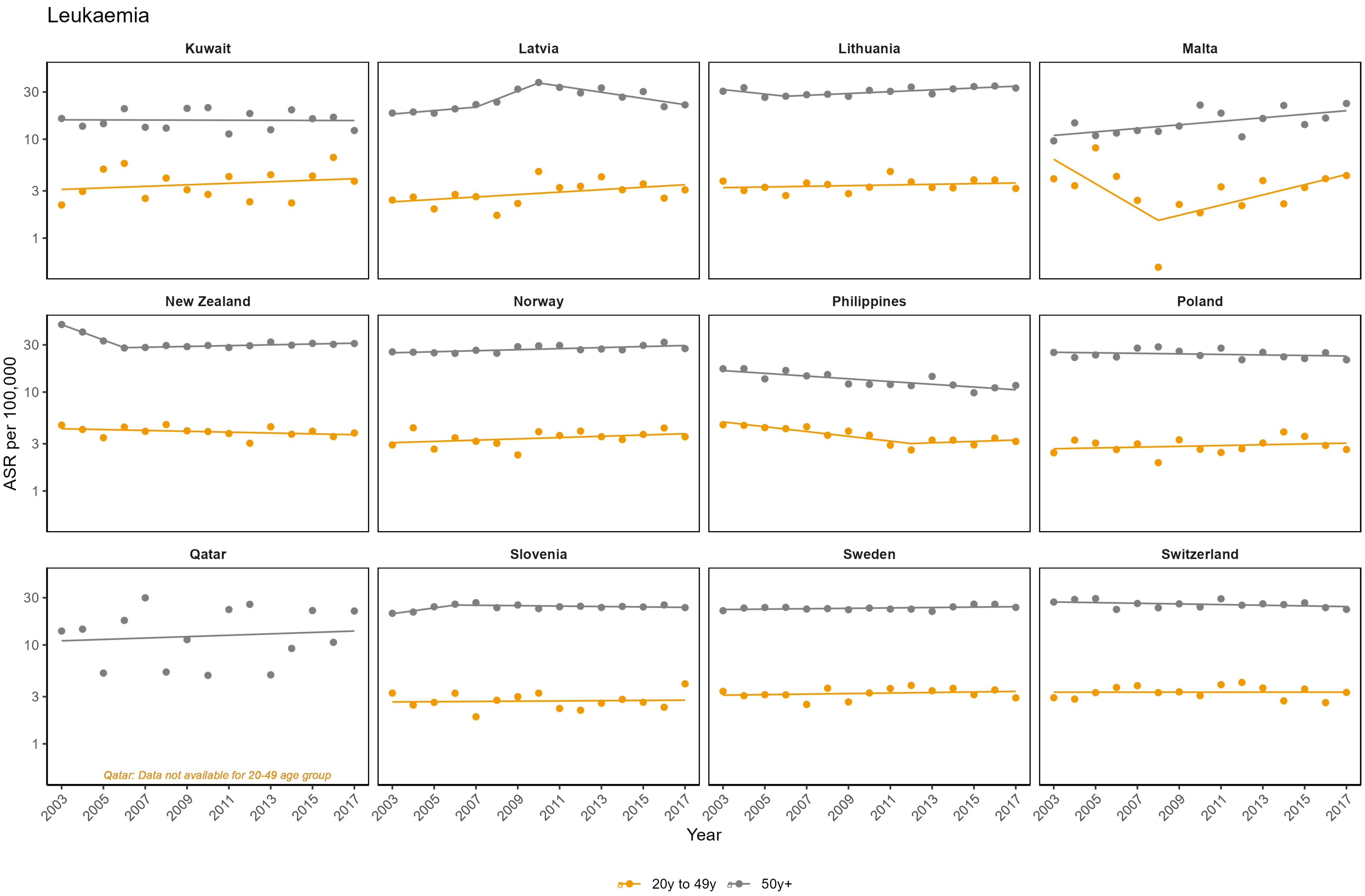

Leukaemia
Kuwait
Latvia
Lithuania
Malta
New Zealand
Norway
Philippines
Poland
Qatar
Slovenia
Sweden
Switzerland
ASR per 100,000
30
10
3
1
Qatar: Data not available for 20-49 age group
2003
2005
2007
2009
2011
2013
2015
2017
Year
20y to 49y
50y+

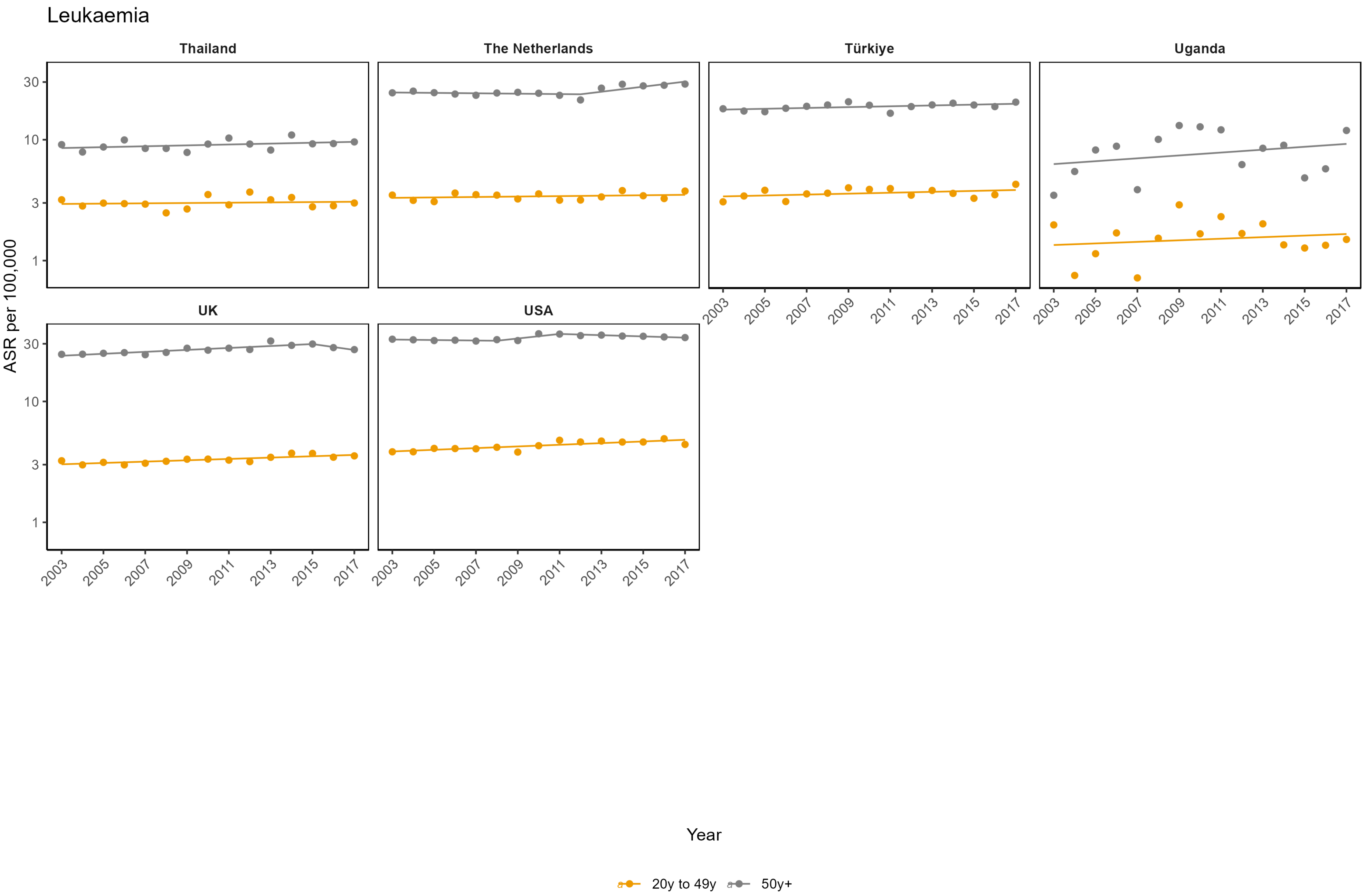
Leukaemia
Thailand
The Netherlands
Türkiye
Uganda
UK
USA
ASR per 100,000
30
10
3
1
2003
2005
2007
2009
2011
2013
2015
2017
Year
20y to 49y
50y+

Appendix Figure 7: Age-standardised incidence rates (ASR) per 100,000

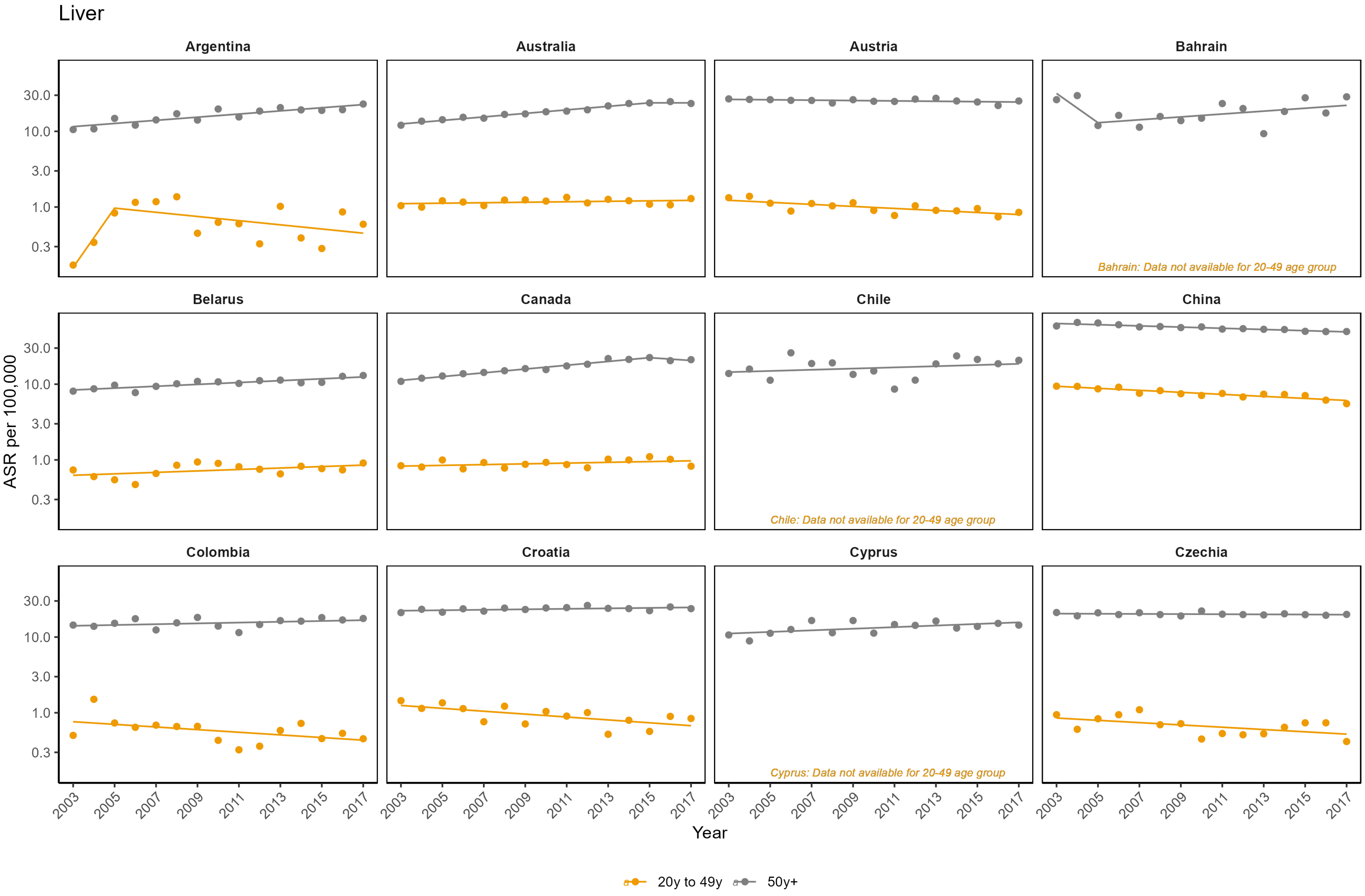

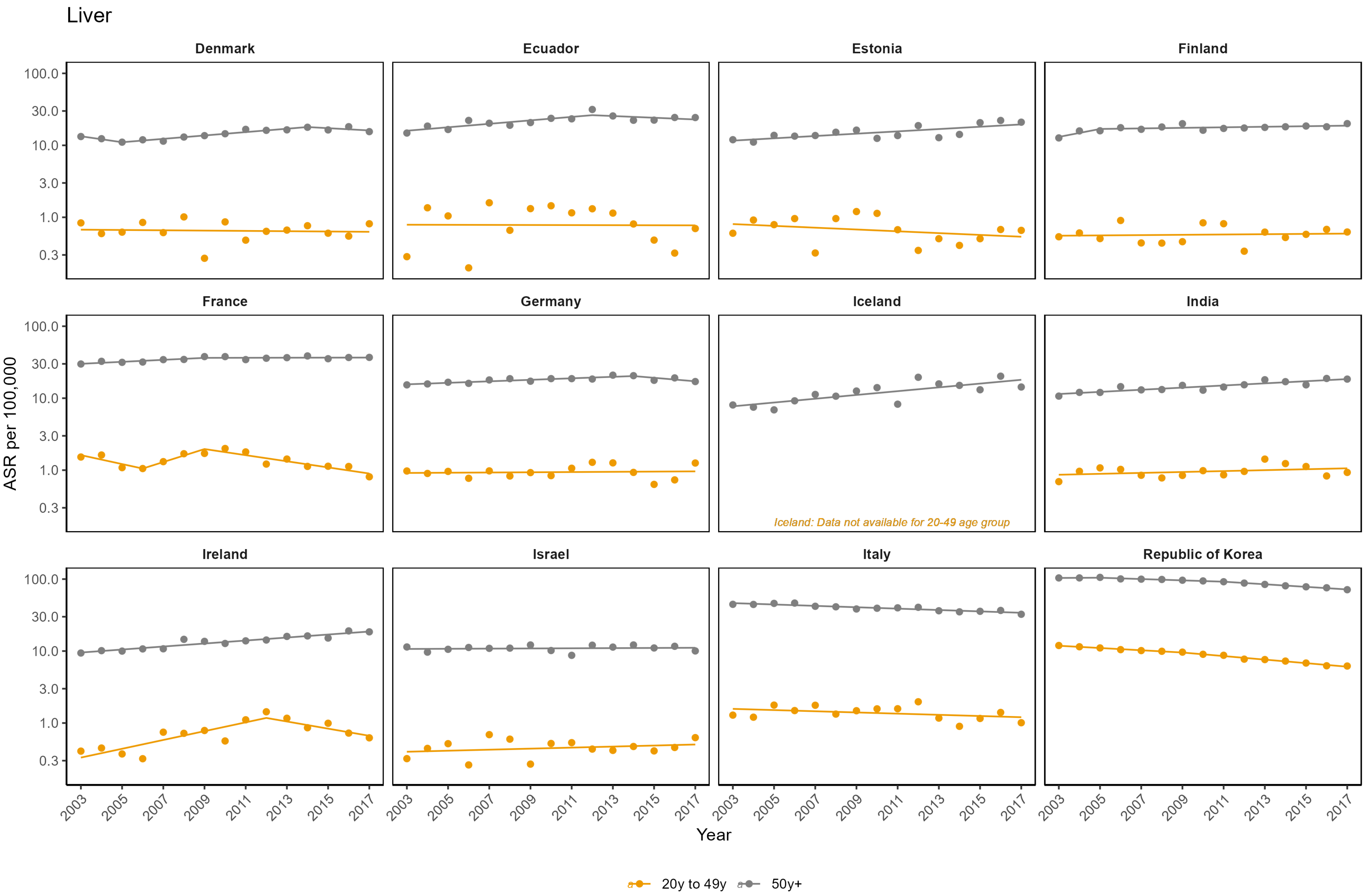

Liver
Denmark
Ecuador
Estonia
Finland
France
Germany
Iceland
India
Ireland
Israel
Italy
Republic of Korea
Iceland: Data not available for 20-49 age group
ASR per 100,000
100.0
30.0
10.0
3.0
1.0
0.3
2003
2005
2007
2009
2011
2013
2015
2017
Year
20y to 49y
50y+

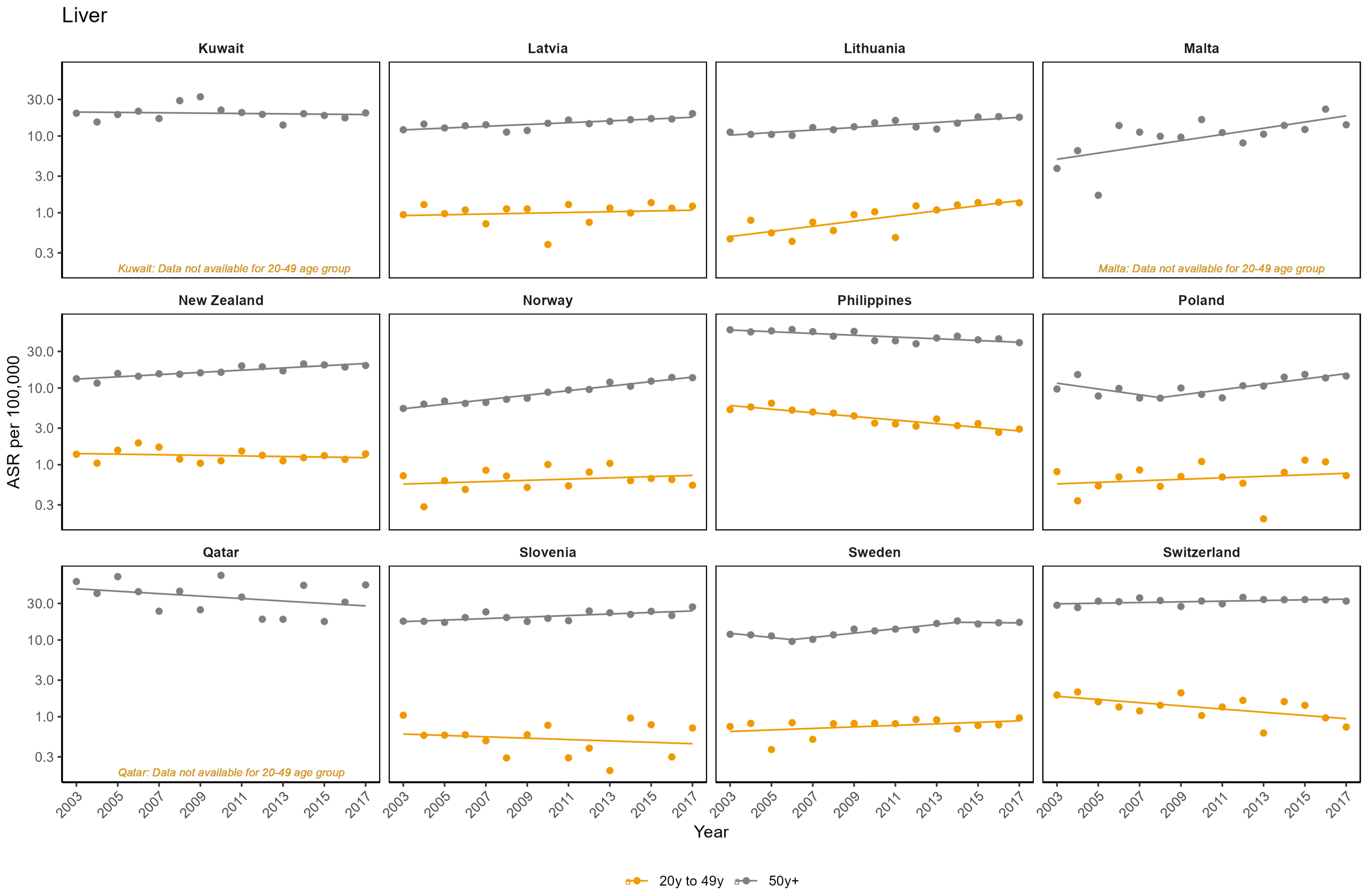
Liver
Kuwait
Latvia
Lithuania
Malta
New Zealand
Norway
Philippines
Poland
Qatar
Slovenia
Sweden
Switzerland
Kuwait: Data not available for 20-49 age group
Malta: Data not available for 20-49 age group
Qatar: Data not available for 20-49 age group
30.0
10.0
3.0
1.0
0.3
ASR per 100,000
2003
2005
2007
2009
2011
2013
2015
2017
Year
20y to 49y
50y+

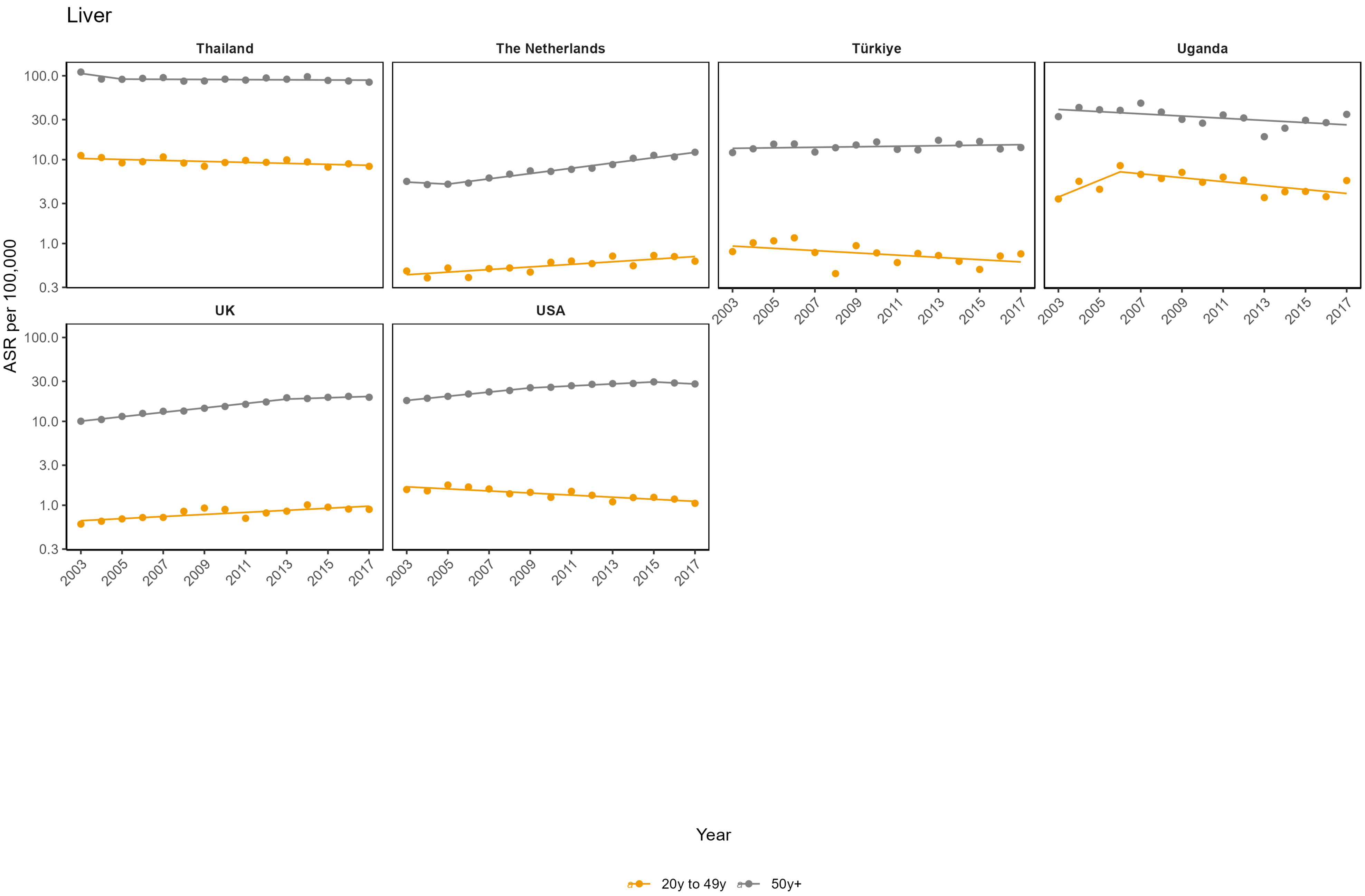

Liver
Thailand
The Netherlands
Türkiye
Uganda
UK
USA
ASR per 100,000
100.0
30.0
10.0
3.0
1.0
0.3
2003
2005
2007
2009
2011
2013
2015
2017
Year
20y to 49y
50y+

Appendix Figure 8: Age-standardised incidence rates (ASR) per 100,000

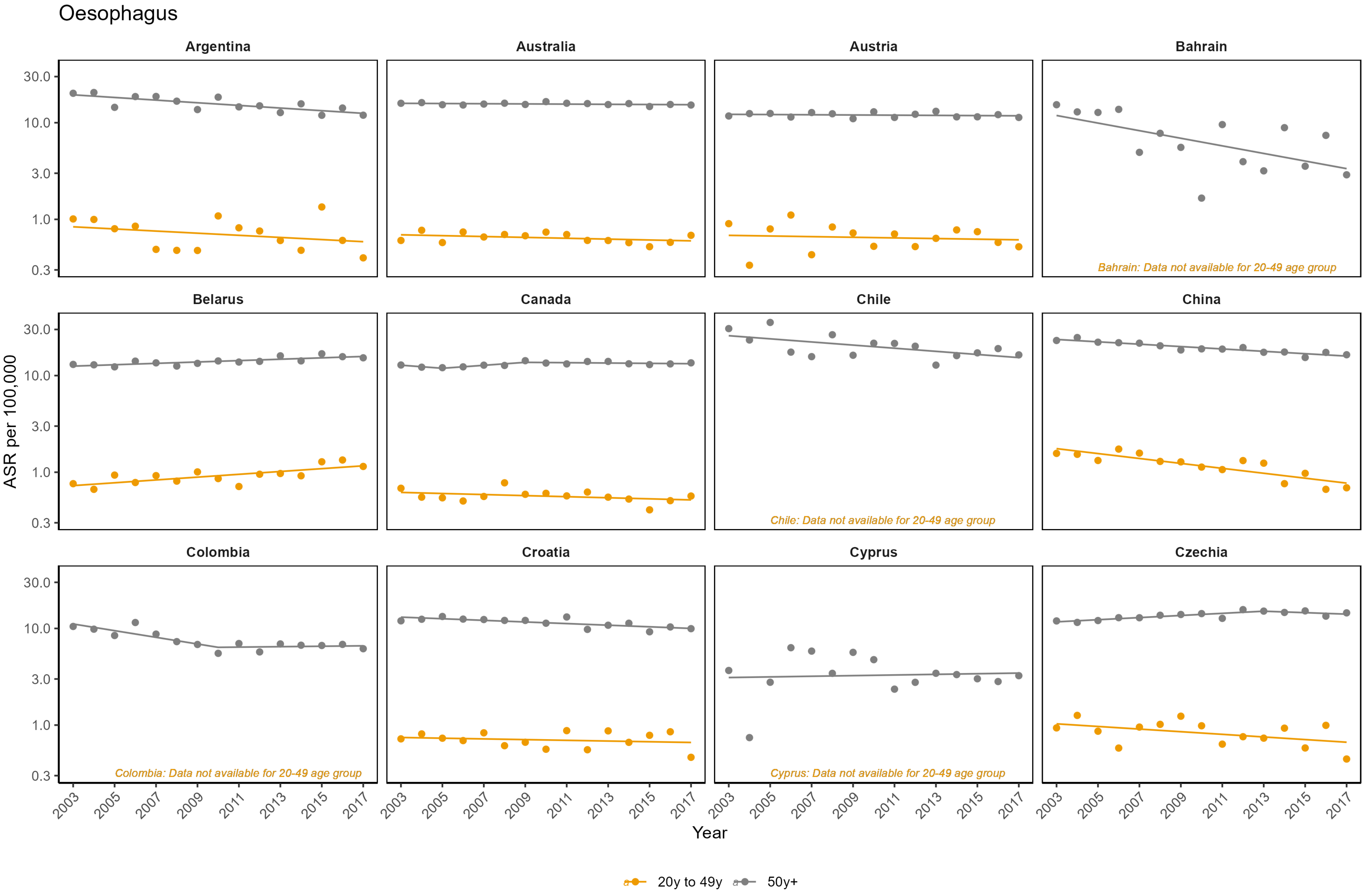

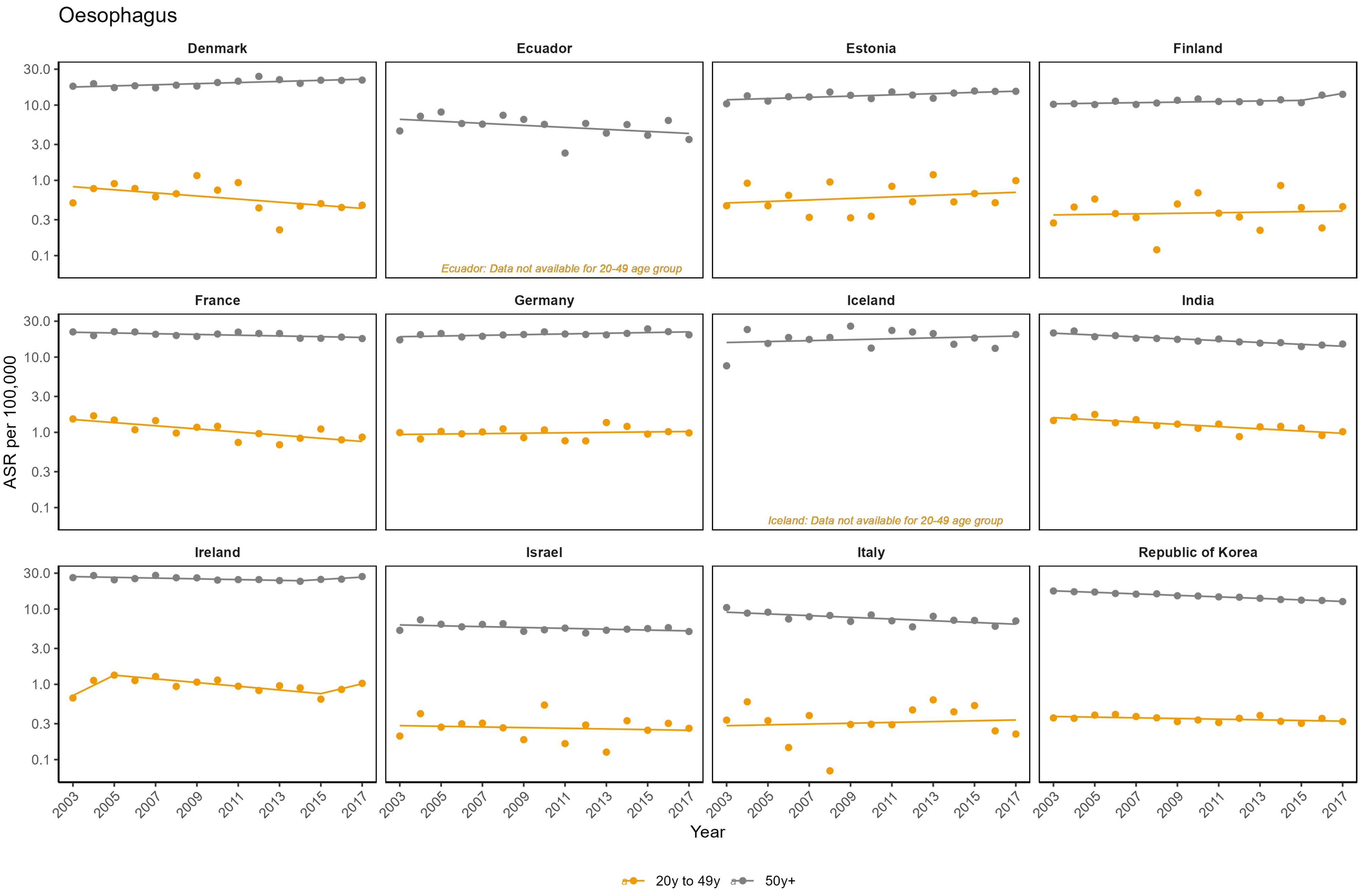
Oesophagus
Denmark
Ecuador
Estonia
Finland
France
Germany
Iceland
India
Ireland
Israel
Italy
Republic of Korea
Ecuador: Data not available for 20-49 age group
Iceland: Data not available for 20-49 age group
ASR per 100,000
30.0
10.0
3.0
1.0
0.3
0.1
2003
2005
2007
2009
2011
2013
2015
2017
Year
20y to 49y
50y+

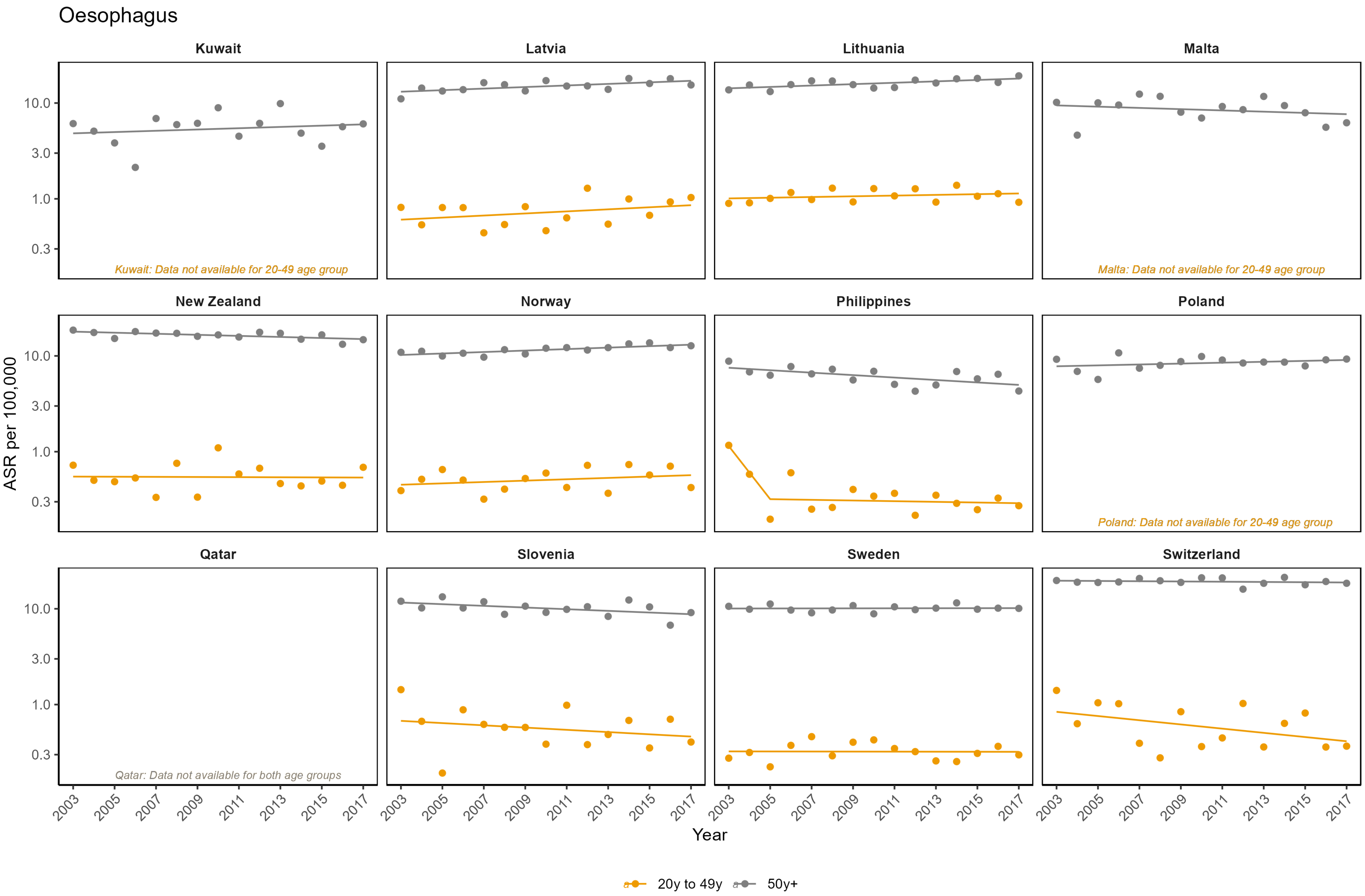

Oesophagus
Kuwait
Latvia
Lithuania
Malta
New Zealand
Norway
Philippines
Poland
Qatar
Slovenia
Sweden
Switzerland
Kuwait: Data not available for 20-49 age group
Malta: Data not available for 20-49 age group
Poland: Data not available for 20-49 age group
Qatar: Data not available for both age groups
ASR per 100,000
10.0
3.0
1.0
0.3
2003
2005
2007
2009
2011
2013
2015
2017
Year
20y to 49y
50y+

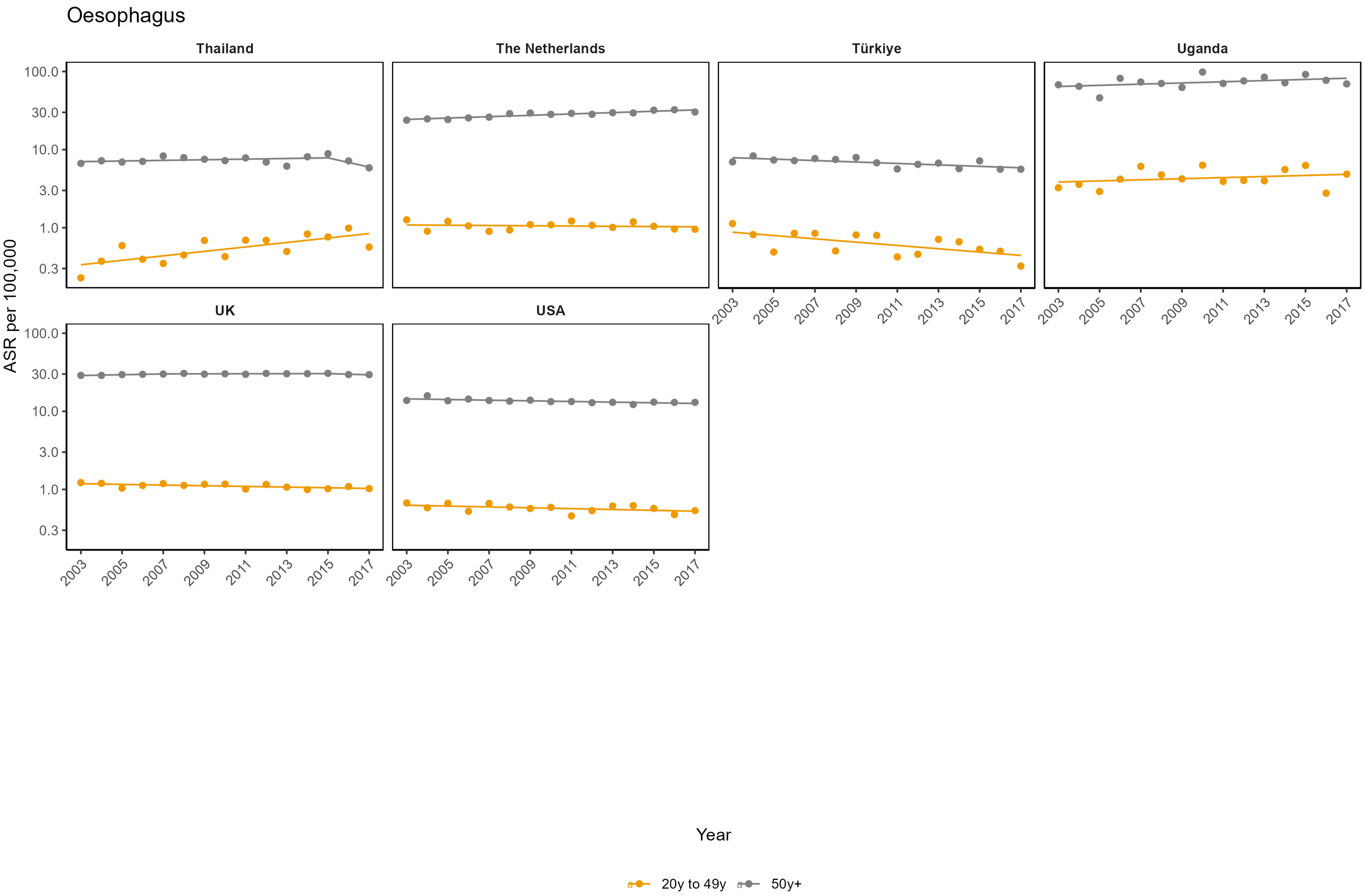

Oesophagus
Thailand
The Netherlands
Türkiye
Uganda
UK
USA
ASR per 100,000
100.0
30.0
10.0
3.0
1.0
0.3
2003
2005
2007
2009
2011
2013
2015
2017
Year
20y to 49y
50y+

Appendix Figure 9: Age-standardised incidence rates (ASR) per 100,000

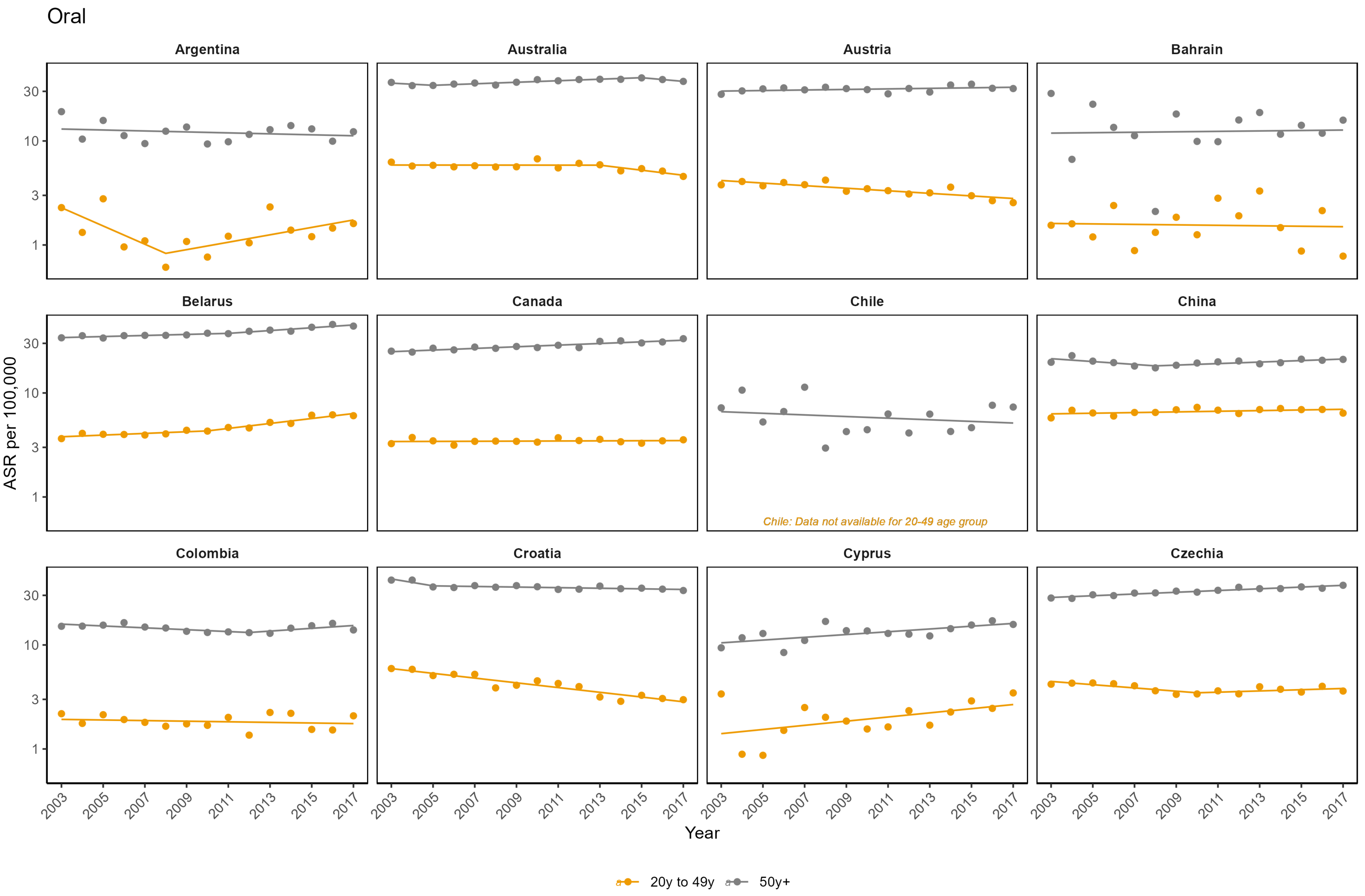

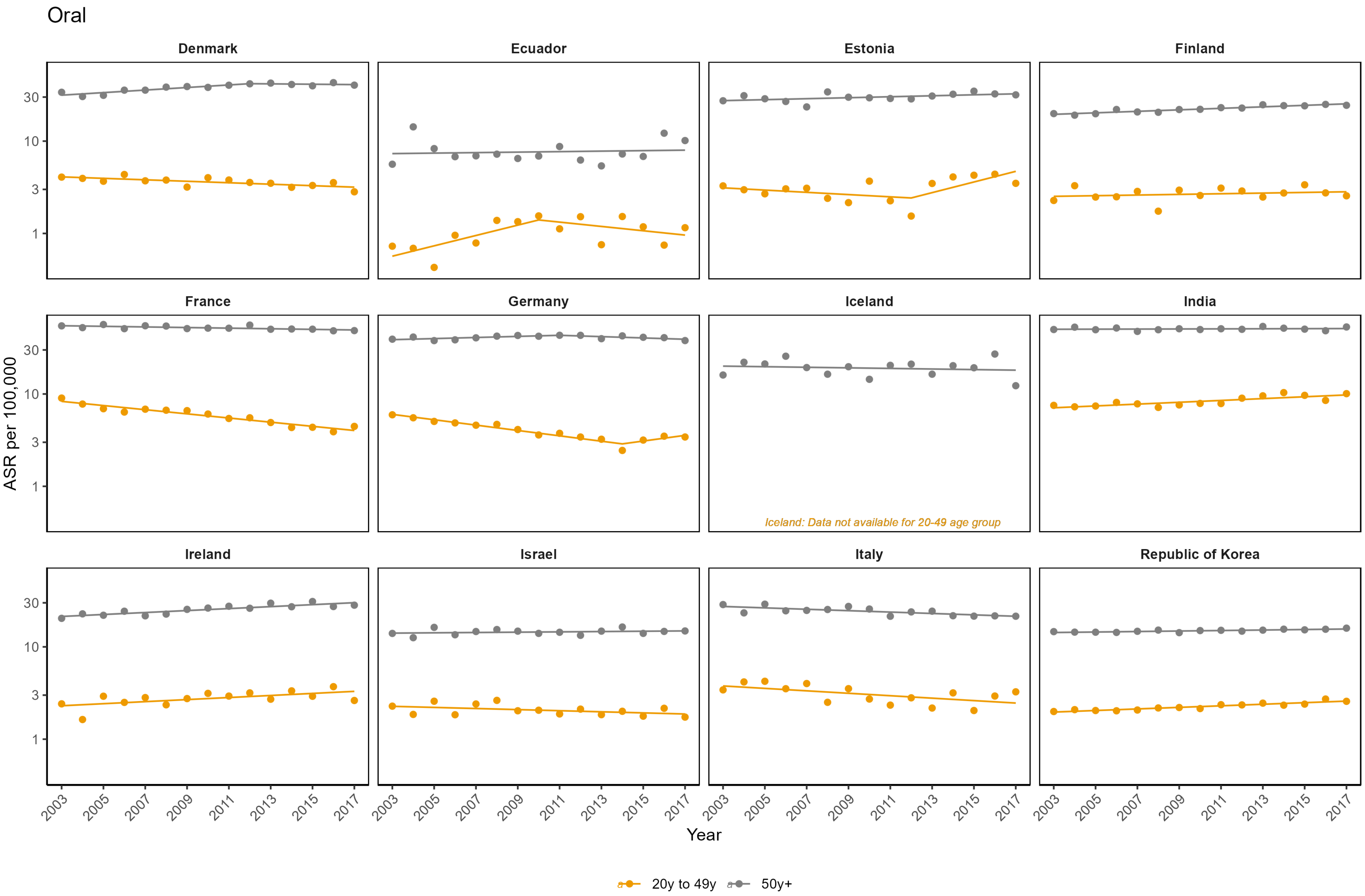
Oral
Denmark
Ecuador
Estonia
Finland
France
Germany
Iceland
India
Ireland
Israel
Italy
Republic of Korea
Iceland: Data not available for 20-49 age group
ASR per 100,000
1
3
10
30
2003
2005
2007
2009
2011
2013
2015
2017
Year
20y to 49y
50y+

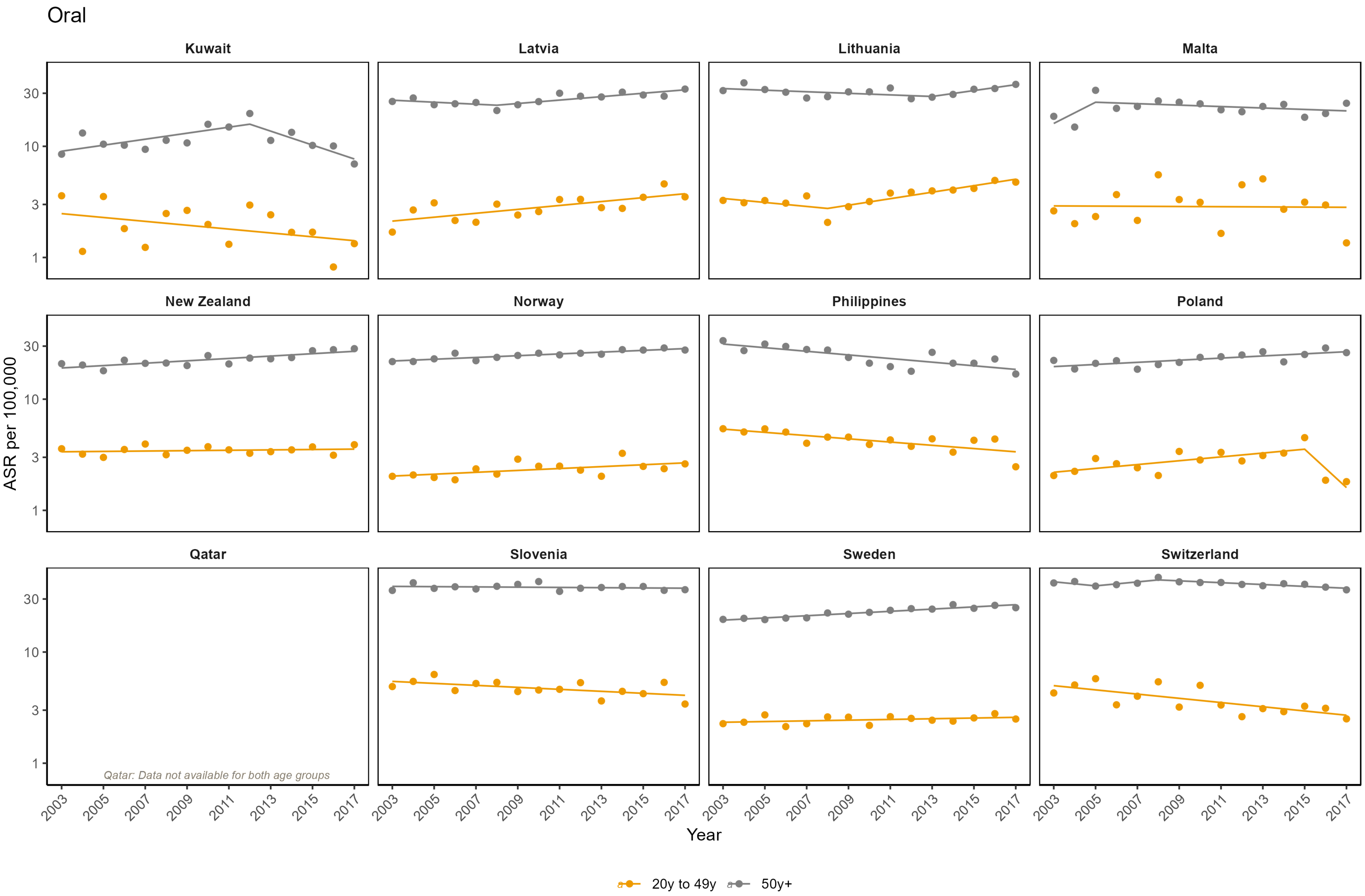

Oral
Kuwait
Latvia
Lithuania
Malta
New Zealand
Norway
Philippines
Poland
Qatar
Slovenia
Sweden
Switzerland
Qatar: Data not available for both age groups
ASR per 100,000
30
10
3
1
2003
2005
2007
2009
2011
2013
2015
2017
Year
20y to 49y
50y+

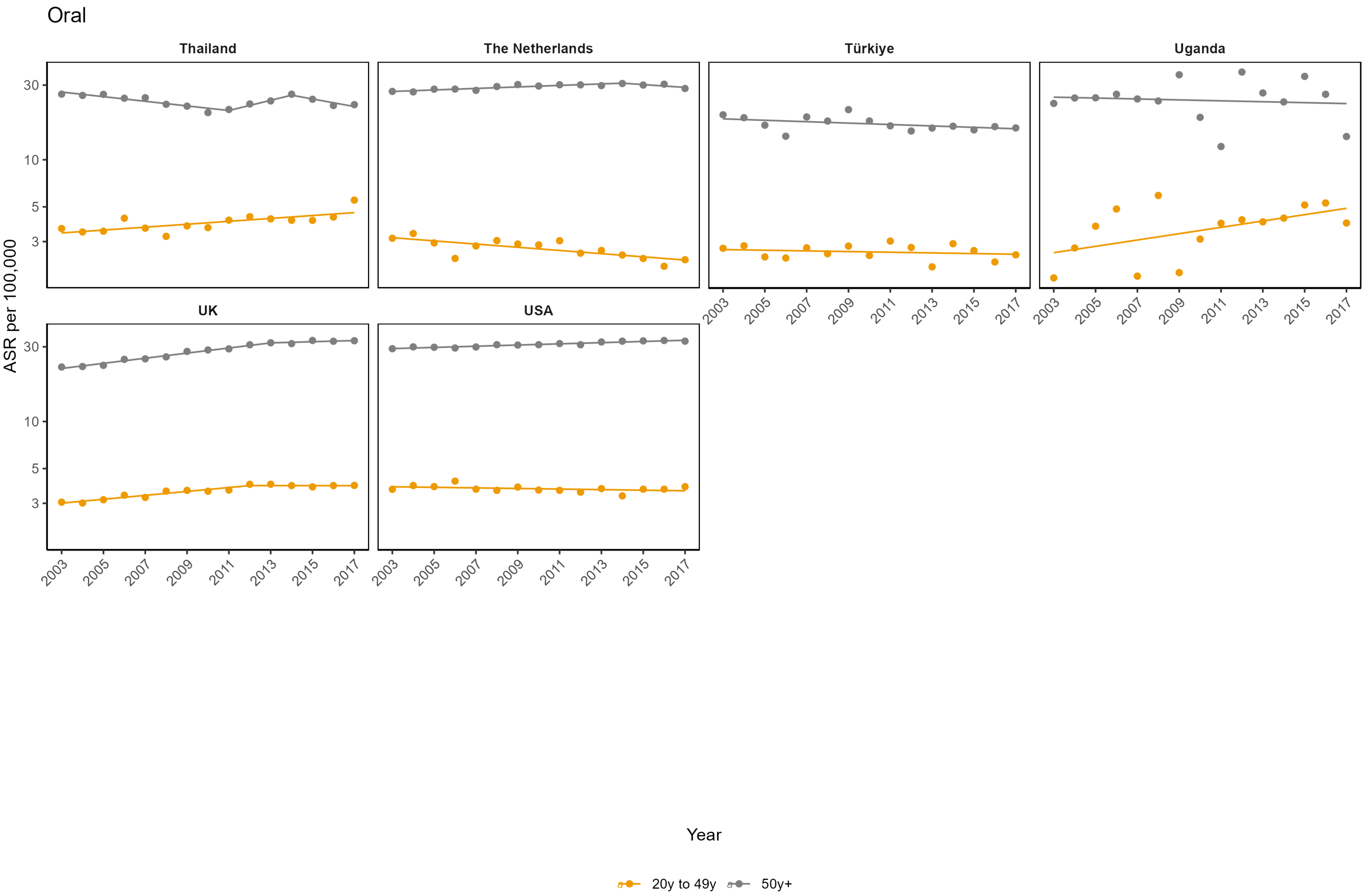

Oral
Thailand
The Netherlands
Türkiye
Uganda
UK
USA
30
10
5
3
ASR per 100,000
2003
2005
2007
2009
2011
2013
2015
2017
Year
20y to 49y
50y+

Appendix Figure 10: Age-standardised incidence rates (ASR) per 100,000

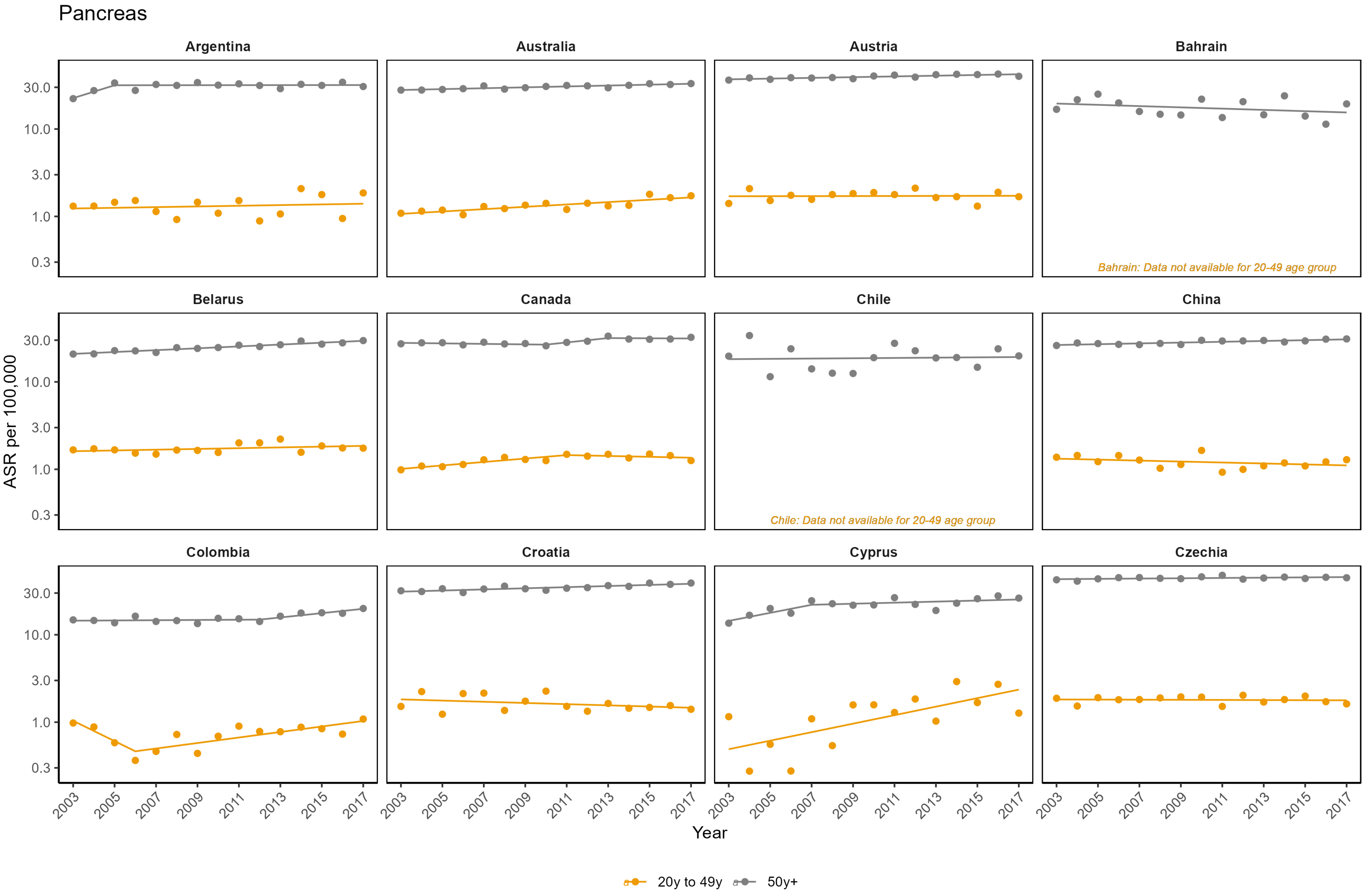

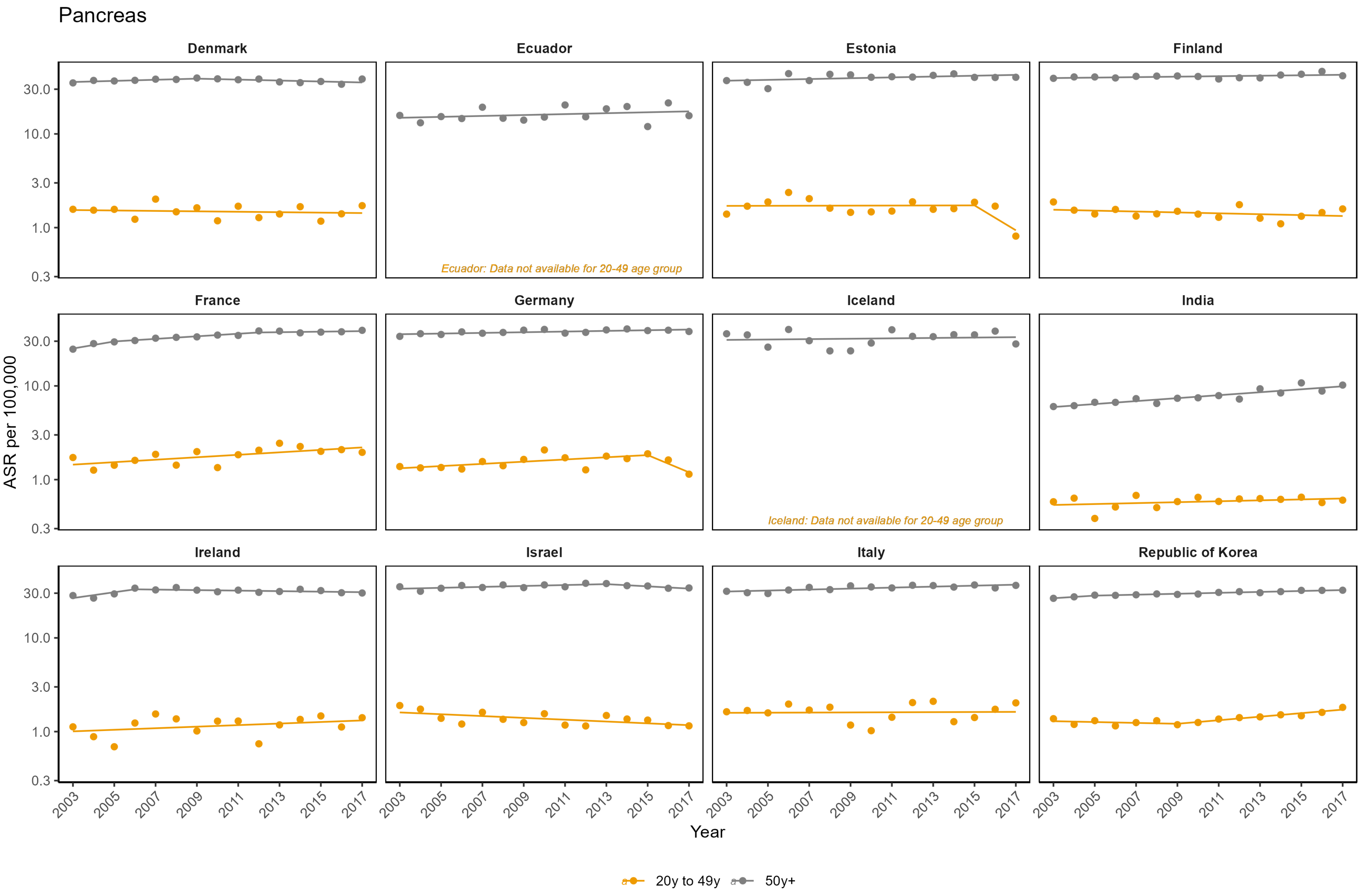

Pancreas
Denmark
Ecuador
Estonia
Finland
France
Germany
Iceland
India
Ireland
Israel
Italy
Republic of Korea
Ecuador: Data not available for 20-49 age group
Iceland: Data not available for 20-49 age group
ASR per 100,000
30.0
10.0
3.0
1.0
0.3
Year
2003
2005
2007
2009
2011
2013
2015
2017
20y to 49y
50y+

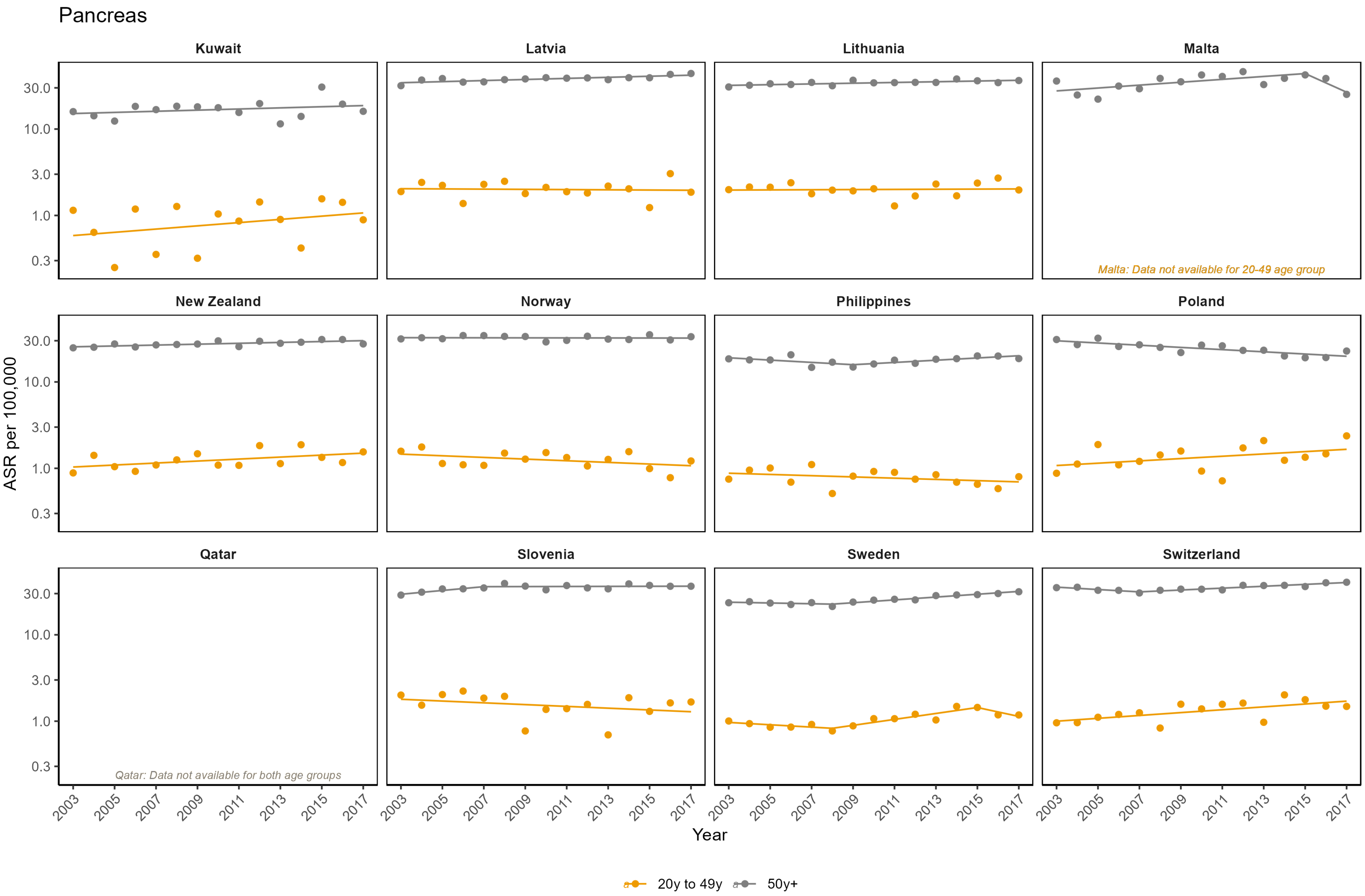
Pancreas
Kuwait
Latvia
Lithuania
Malta
New Zealand
Norway
Philippines
Poland
Qatar
Slovenia
Sweden
Switzerland
Malta: Data not available for 20-49 age group
Qatar: Data not available for both age groups
ASR per 100,000
30.0
10.0
3.0
1.0
0.3
Year
2003
2005
2007
2009
2011
2013
2015
2017
20y to 49y
50y+

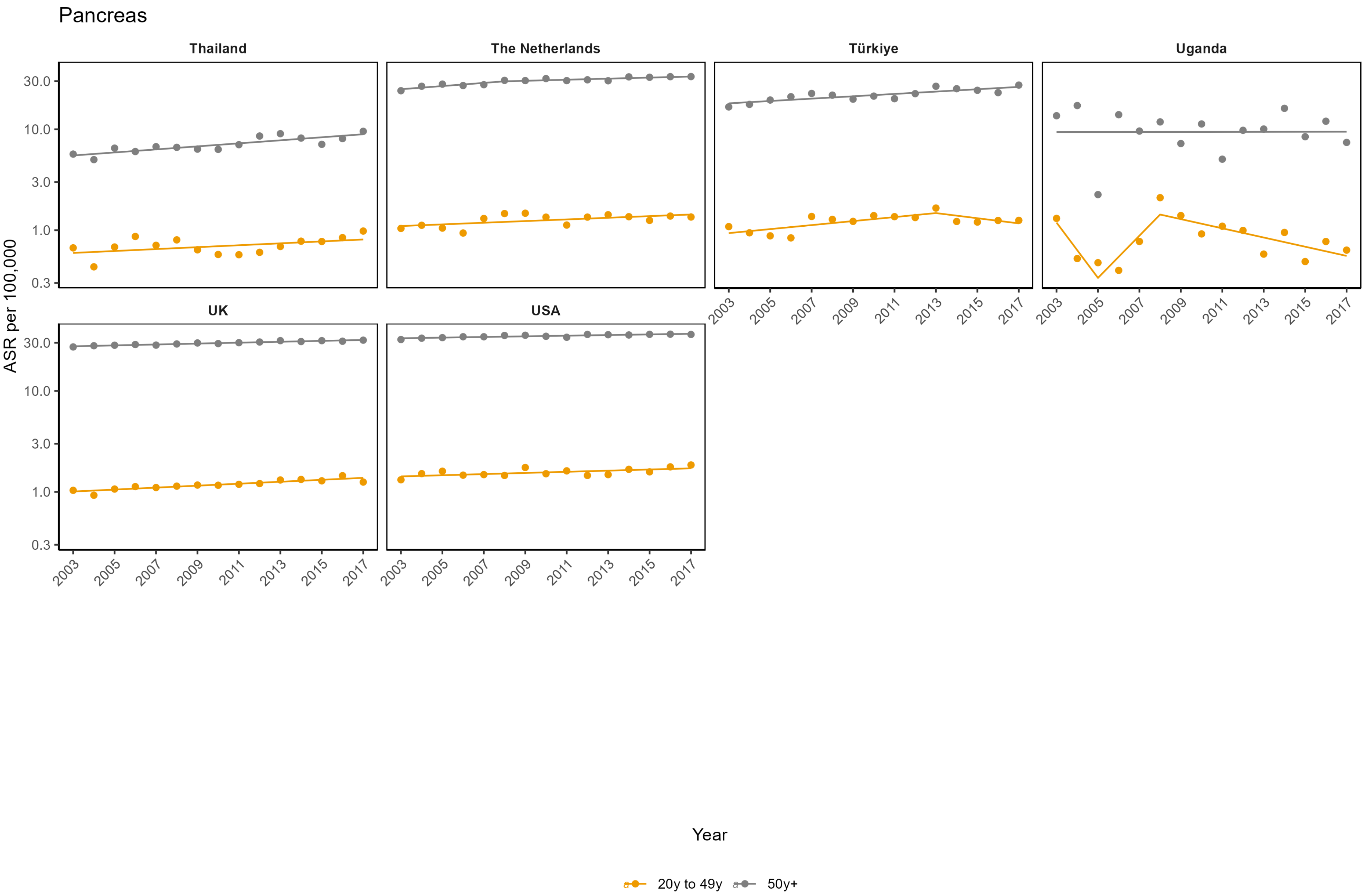

Pancreas
Thailand
The Netherlands
Türkiye
Uganda
UK
USA
ASR per 100,000
30.0
10.0
3.0
1.0
0.3
2003
2005
2007
2009
2011
2013
2015
2017
Year
20y to 49y
50y+

Appendix Figure 11: Age-standardised incidence rates (ASR) per 100,000

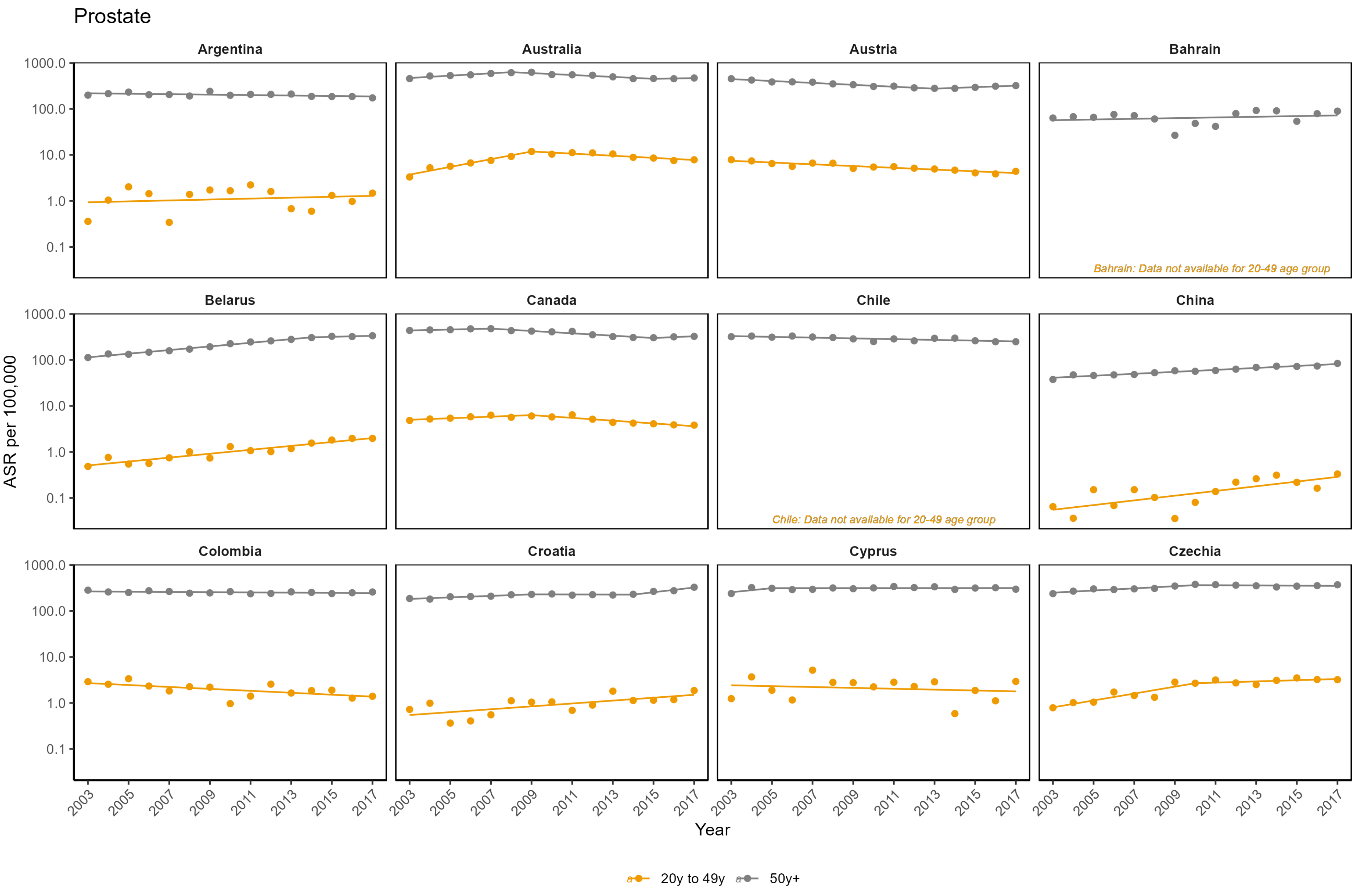

Prostate

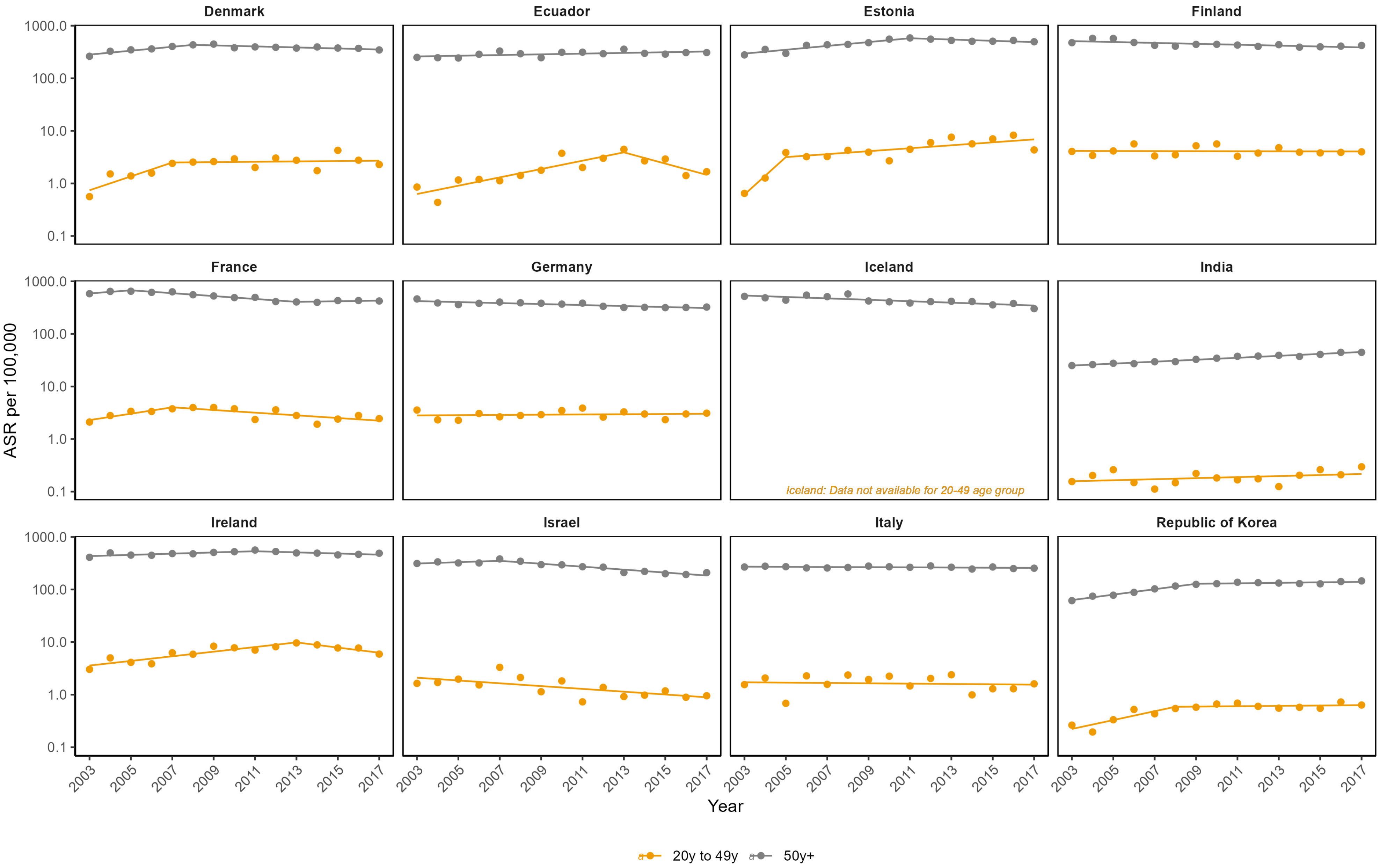

Denmark
Ecuador
Estonia
Finland
France
Germany
Iceland
India
Ireland
Israel
Italy
Republic of Korea
1000.0
100.0
10.0
1.0
0.1
ASR per 100,000
Iceland: Data not available for 20-49 age group
2003
2005
2007
2009
2011
2013
2015
2017
Year
20y to 49y
50y+

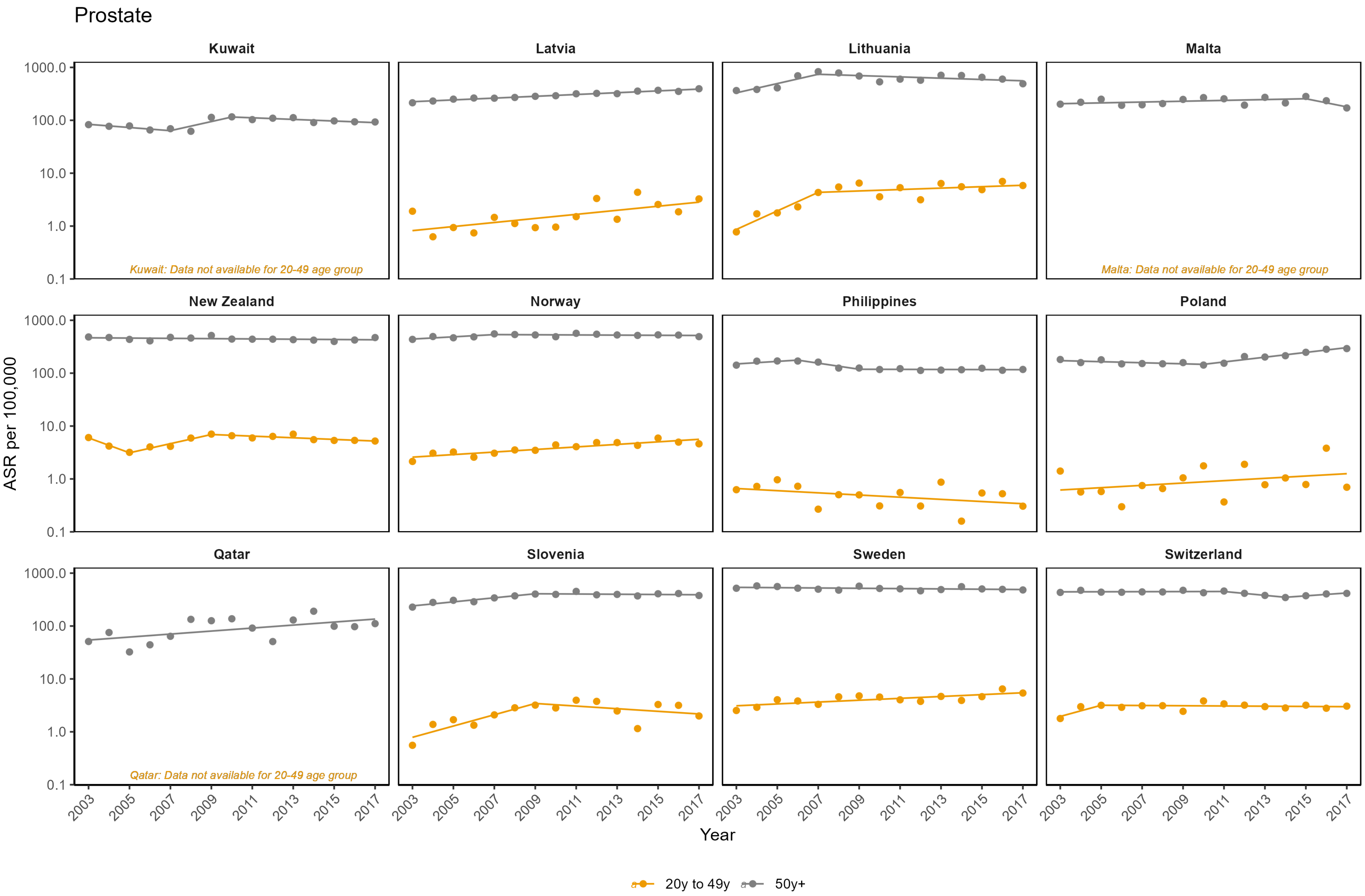

Prostate
Kuwait
Latvia
Lithuania
Malta
New Zealand
Norway
Philippines
Poland
Qatar
Slovenia
Sweden
Switzerland
Kuwait: Data not available for 20-49 age group
Malta: Data not available for 20-49 age group
Qatar: Data not available for 20-49 age group
ASR per 100,000
1000.0
100.0
10.0
1.0
0.1
2003
2005
2007
2009
2011
2013
2015
2017
Year
20y to 49y
50y+

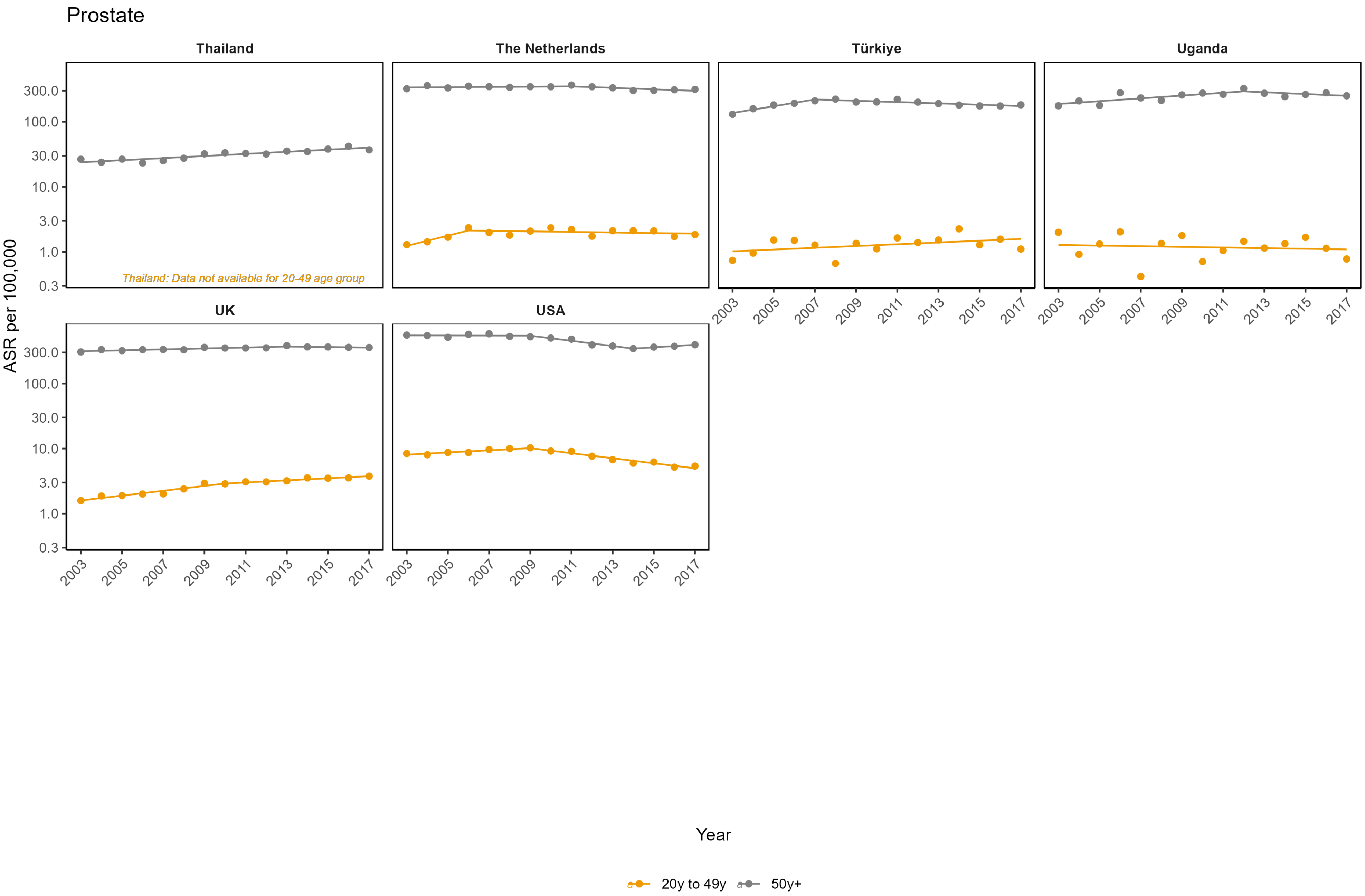
Prostate
Thailand
The Netherlands
Türkiye
Uganda
UK
USA
Thailand: Data not available for 20-49 age group
ASR per 100,000
300.0
100.0
30.0
10.0
3.0
1.0
0.3
2003
2005
2007
2009
2011
2013
2015
2017
Year
20y to 49y
50y+

Appendix Figure 12: Age-standardised incidence rates (ASR) per 100,000

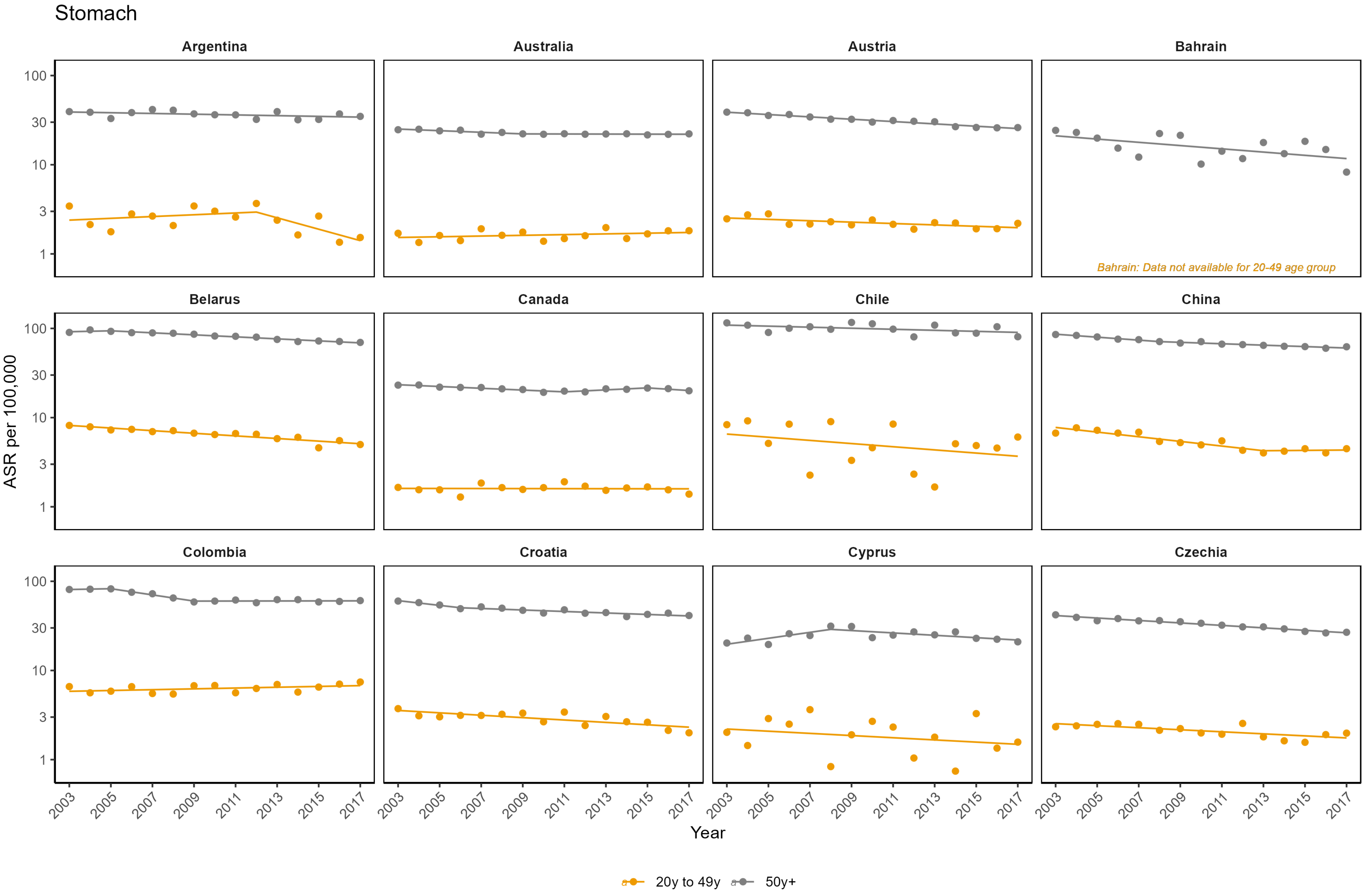

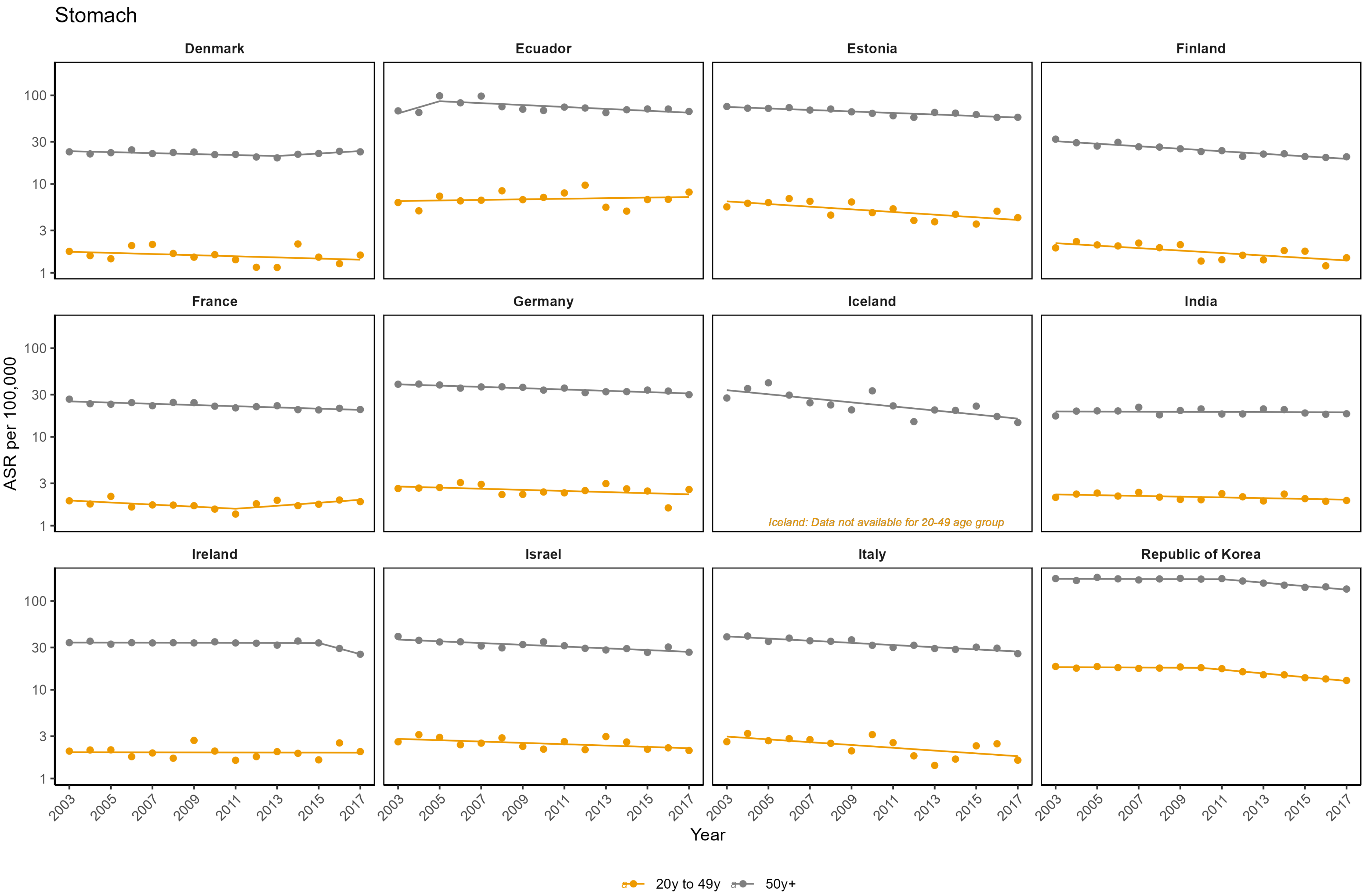
Stomach
Denmark
Ecuador
Estonia
Finland
France
Germany
Iceland
India
Ireland
Israel
Italy
Republic of Korea
Iceland: Data not available for 20-49 age group
ASR per 100,000
1
3
10
30
100
2003
2005
2007
2009
2011
2013
2015
2017
Year
20y to 49y
50y+

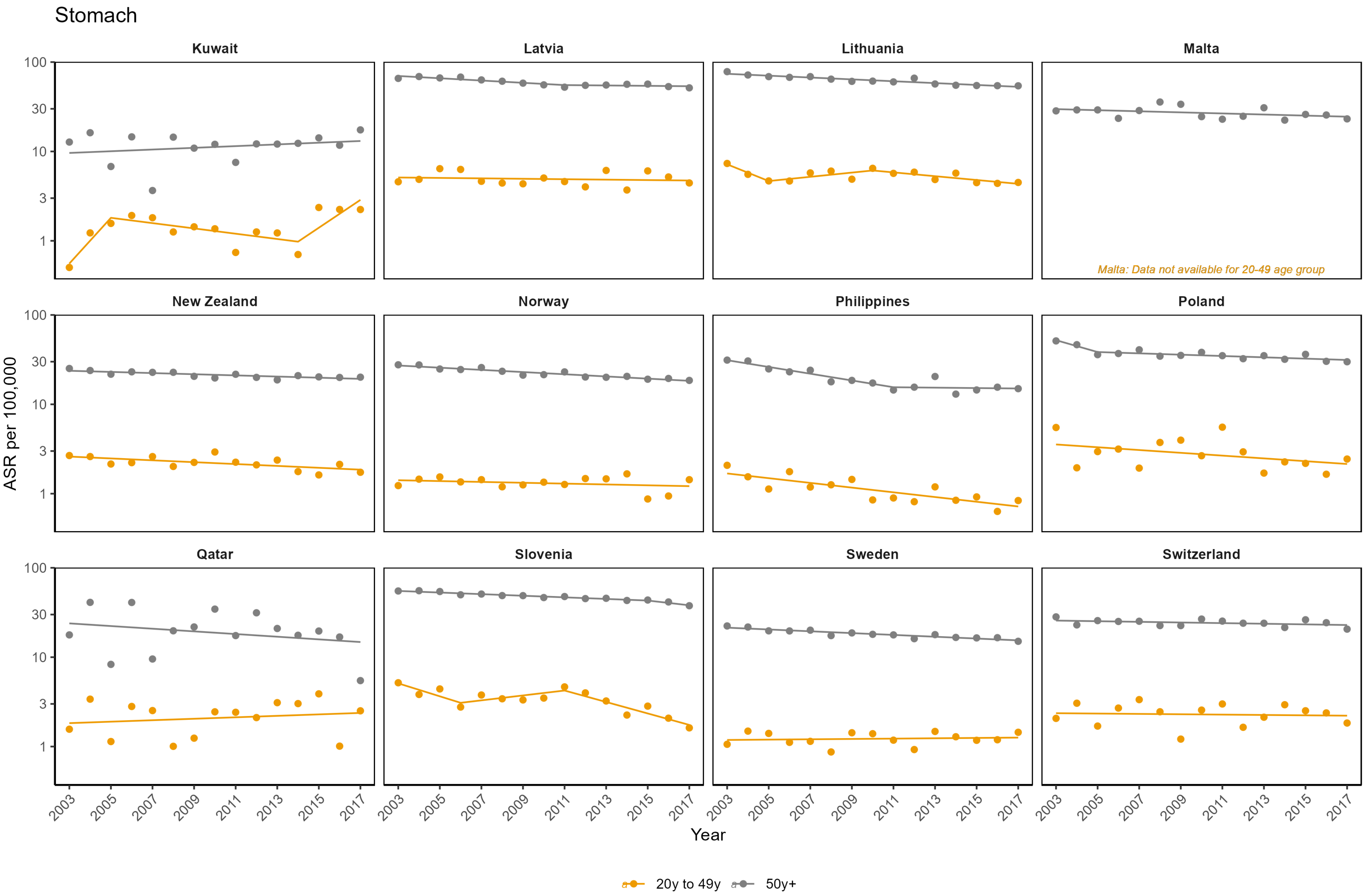
Stomach
Kuwait
Latvia
Lithuania
Malta
New Zealand
Norway
Philippines
Poland
Qatar
Slovenia
Sweden
Switzerland
Malta: Data not available for 20-49 age group
ASR per 100,000
100
30
10
3
1
2003
2005
2007
2009
2011
2013
2015
2017
Year
20y to 49y
50y+

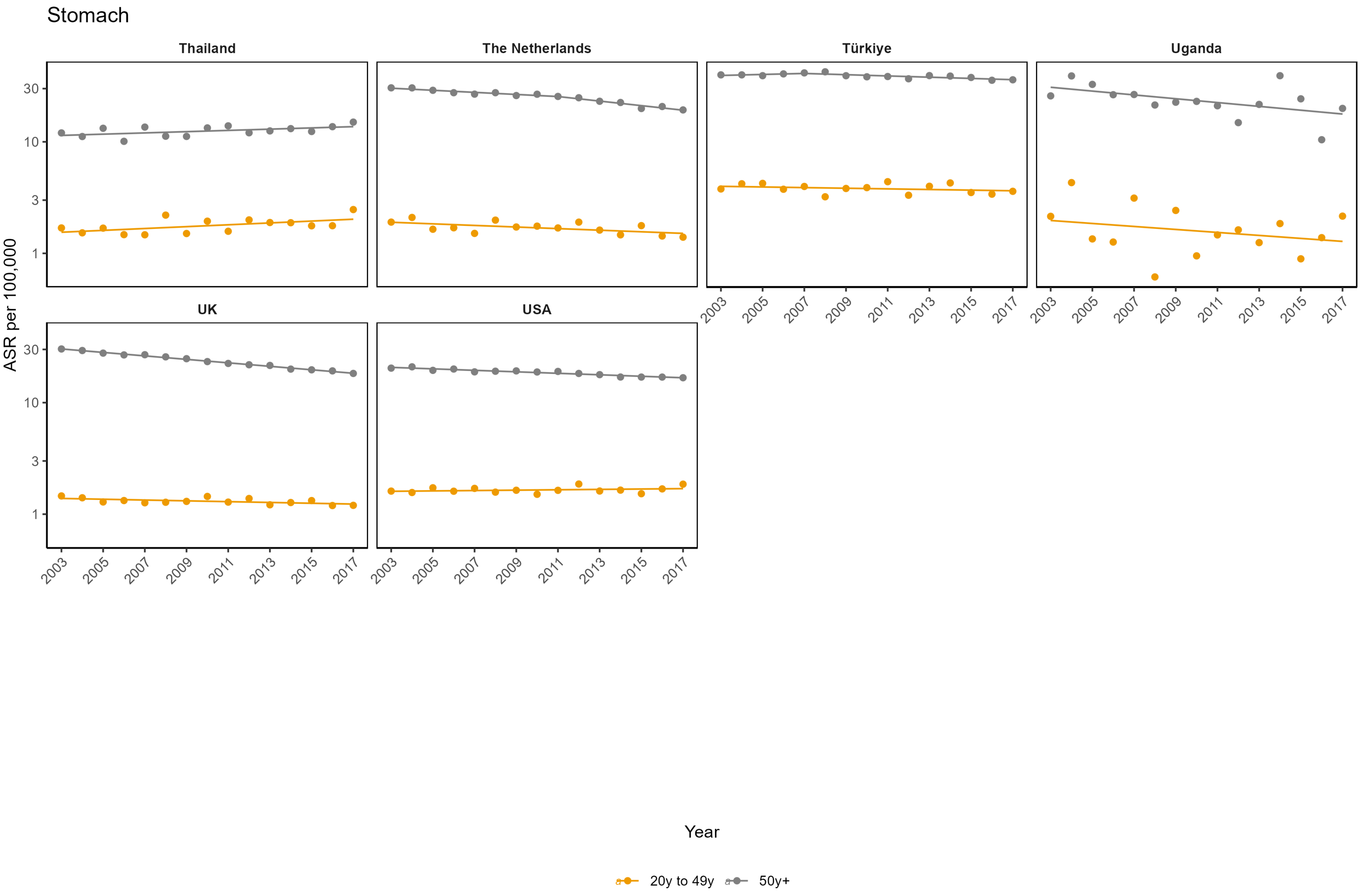

Stomach
Thailand
The Netherlands
Türkiye
Uganda
UK
USA
ASR per 100,000
30
10
3
1
2003
2005
2007
2009
2011
2013
2015
2017
Year
20y to 49y
50y+

Appendix Figure 13: Age-standardised incidence rates (ASR) per 100,000

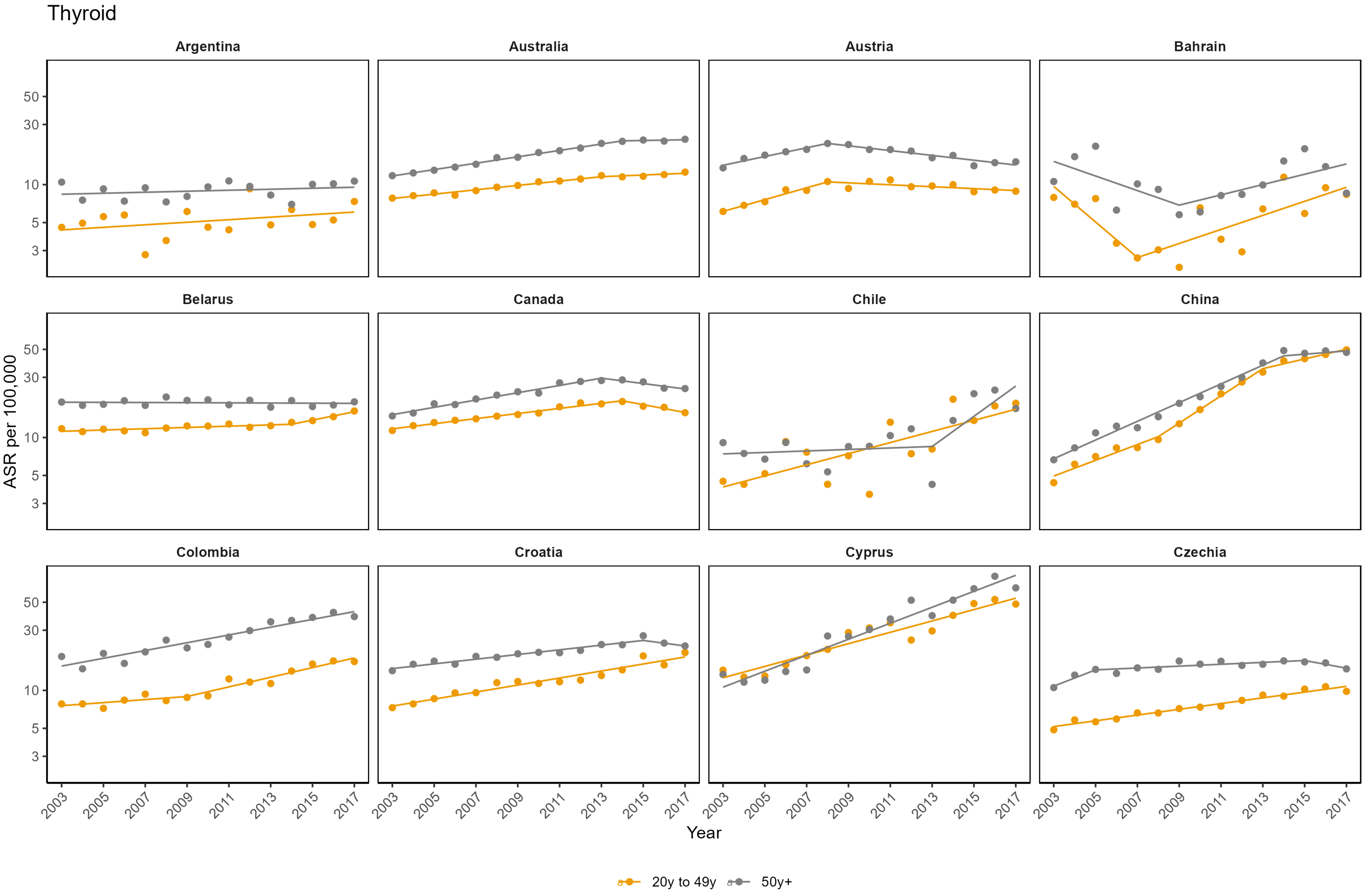

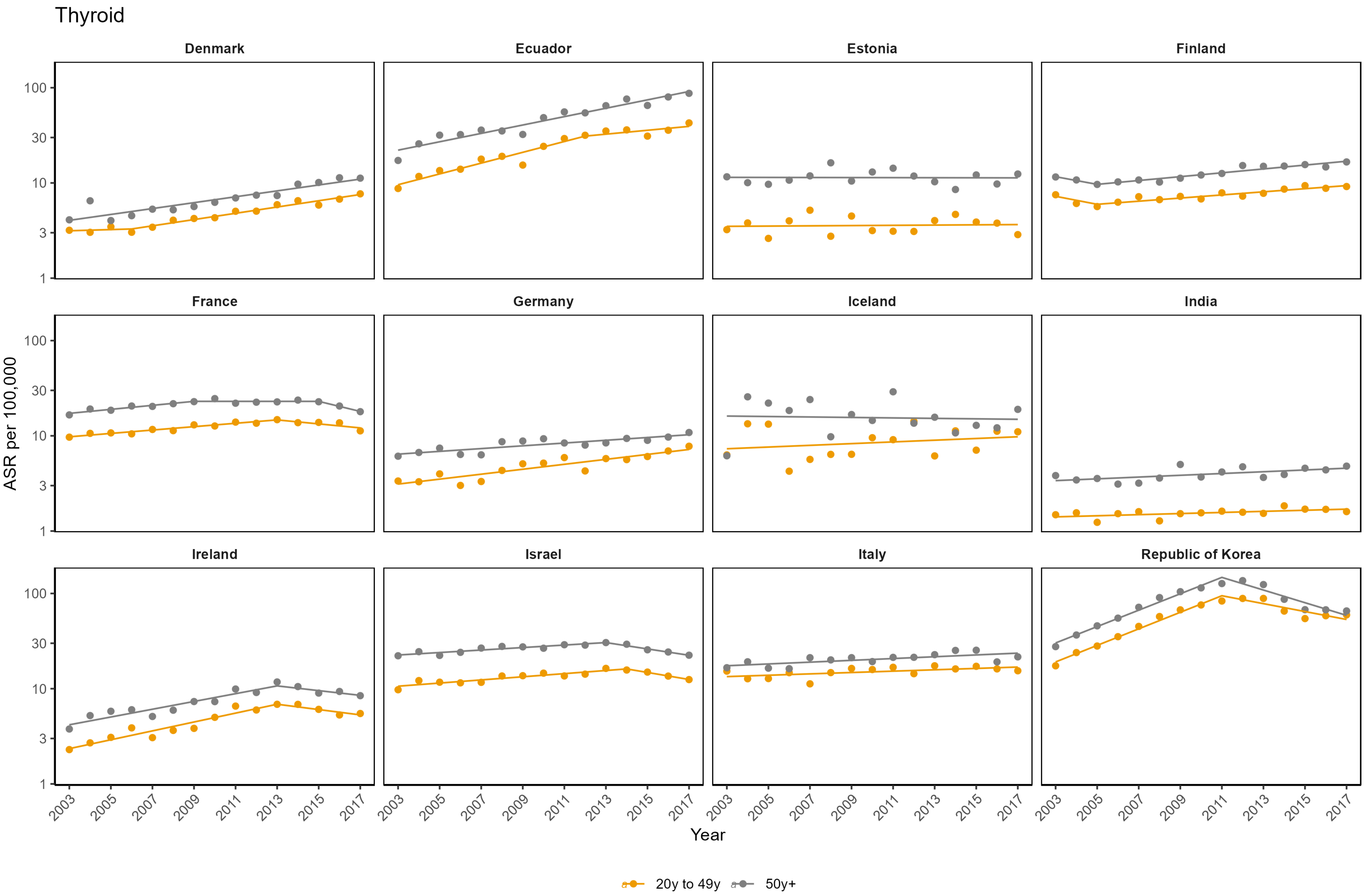

Thyroid
Denmark
Ecuador
Estonia
Finland
France
Germany
Iceland
India
Ireland
Israel
Italy
Republic of Korea
ASR per 100,000
1
3
10
30
100
2003
2005
2007
2009
2011
2013
2015
2017
Year
20y to 49y
50y+

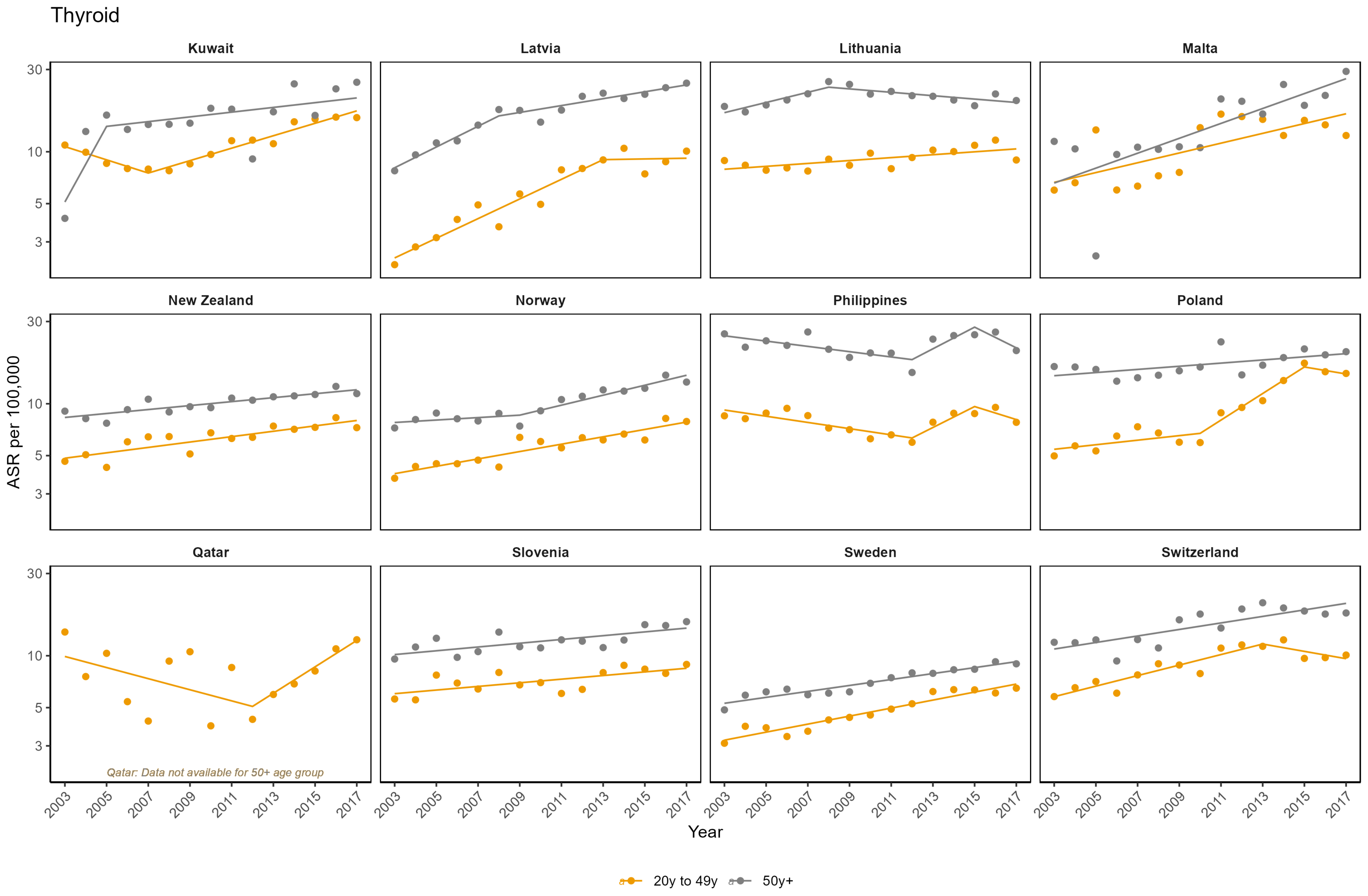
Thyroid
Kuwait
Latvia
Lithuania
Malta
New Zealand
Norway
Philippines
Poland
Qatar
Slovenia
Sweden
Switzerland
ASR per 100,000
30
10
5
3
Qatar: Data not available for 50+ age group
2003
2005
2007
2009
2011
2013
2015
2017
Year
20y to 49y
50y+

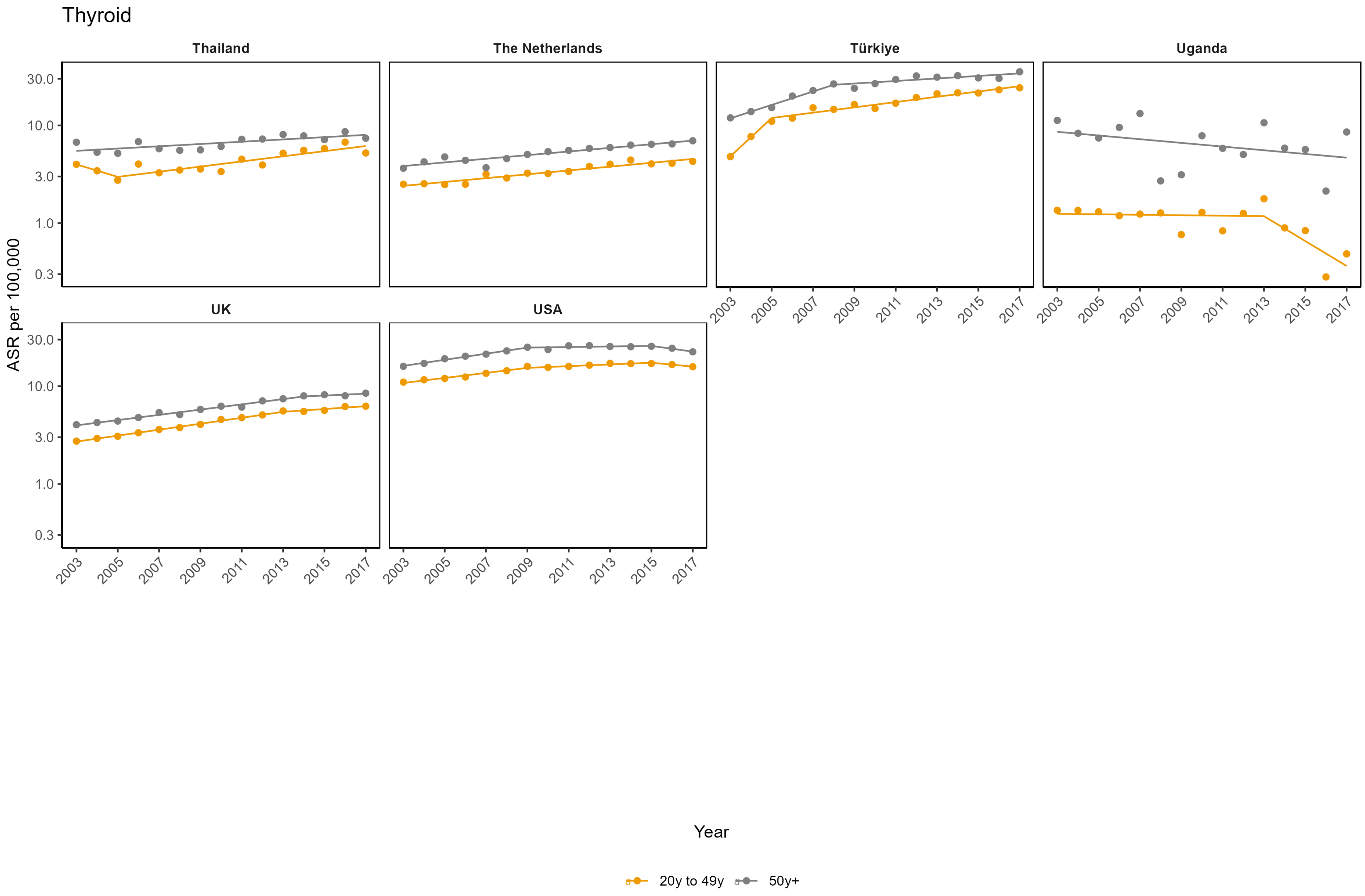

Thyroid
Thailand
The Netherlands
Türkiye
Uganda
UK
USA
30.0
10.0
3.0
1.0
0.3
ASR per 100,000
2003
2005
2007
2009
2011
2013
2015
2017
Year
20y to 49y
50y+

Appendix Figure 14: Average annual percent change (AAPC) for cancer incidence (2003-2017) by UN region, country and age - Breast Cancer

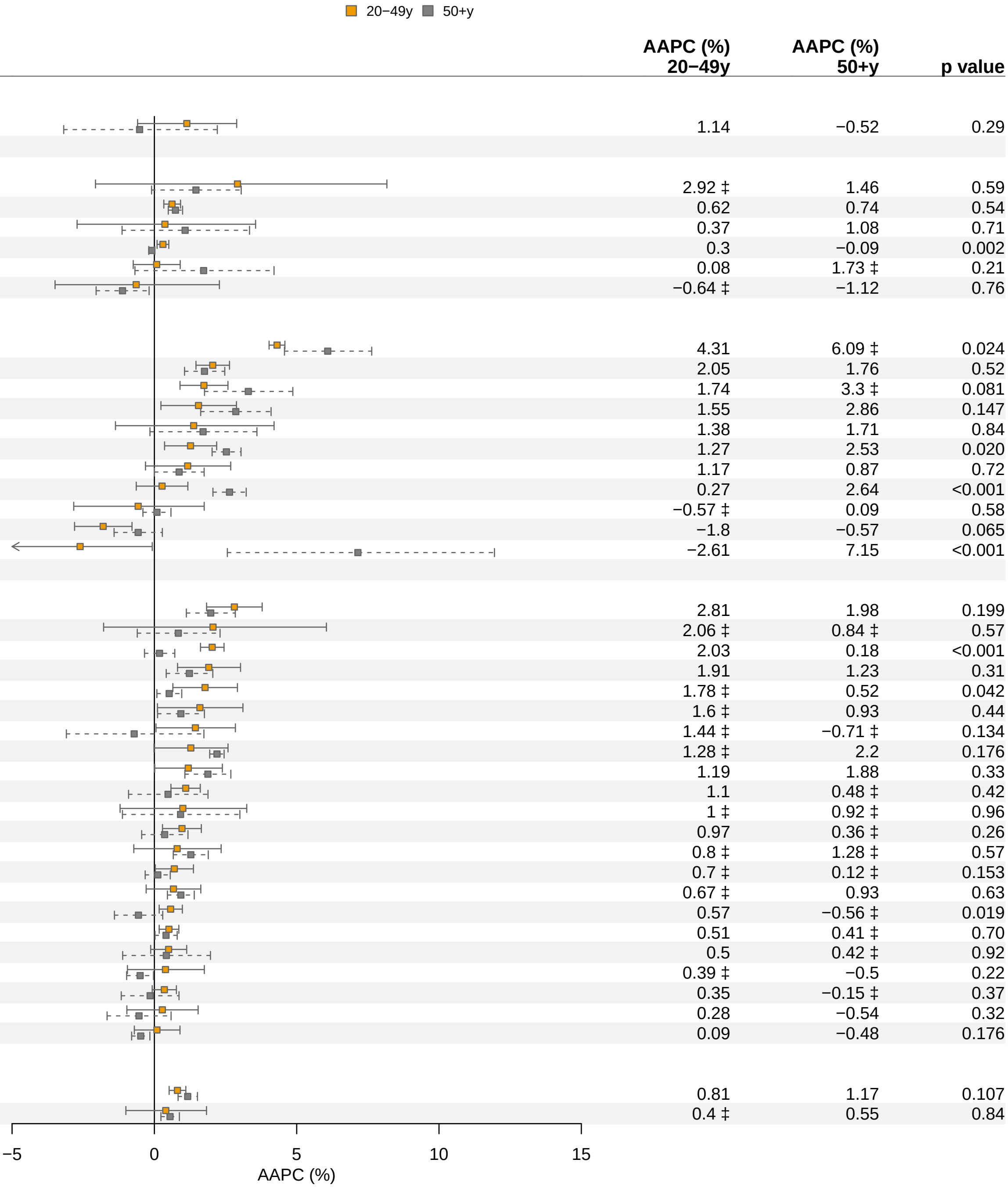

Country	AAPC (%) 20−49y	AAPC (%) 50+y	p value
**Africa**			
Uganda	1.14	−0.52	0.29
**Americas**			
Ecuador	2.92 ‡	1.46	0.59
Canada	0.62	0.74	0.54
Chile	0.37	1.08	0.71
USA	0.3	−0.09	0.002
Colombia	0.08	1.73 ‡	0.21
Argentina	−0.64 ‡	−1.12	0.76
**Asia**			
Republic of Korea	4.31	6.09 ‡	0.024
Türkiye	2.05	1.76	0.52
Thailand	1.74	3.3 ‡	0.081
Kuwait	1.55	2.86	0.147
Bahrain	1.38	1.71	0.84
China	1.27	2.53	0.020
Cyprus	1.17	0.87	0.72
India	0.27	2.64	<0.001
Israel	−0.57 ‡	0.09	0.58
Philippines	−1.8	−0.57	0.065
Qatar	−2.61	7.15	<0.001
**Europe**			
Latvia	2.81	1.98	0.199
Malta	2.06 ‡	0.84 ‡	0.57
Czechia	2.03	0.18	<0.001
Estonia	1.91	1.23	0.31
Slovenia	1.78 ‡	0.52	0.042
Croatia	1.6 ‡	0.93	0.44
Germany	1.44 ‡	−0.71 ‡	0.134
Belarus	1.28 ‡	2.2	0.176
Poland	1.19	1.88	0.33
Denmark	1.1	0.48 ‡	0.42
Lithuania	1 ‡	0.92 ‡	0.96
Norway	0.97	0.36 ‡	0.26
Finland	0.8 ‡	1.28 ‡	0.57
UK	0.7 ‡	0.12 ‡	0.153
Italy	0.67 ‡	0.93	0.63
France	0.57	−0.56 ‡	0.019
The Netherlands	0.51	0.41 ‡	0.70
Ireland	0.5	0.42 ‡	0.92
Austria	0.39 ‡	−0.5	0.22
Sweden	0.35	−0.15 ‡	0.37
Iceland	0.28	−0.54	0.32
Switzerland	0.09	−0.48	0.176
**Oceania**			
Australia	0.81	1.17	0.107
New Zealand	0.4 ‡	0.55	0.84

Data for female sex only.
‡ best fitted Joinpoint model > 0 joins; * p−value for a difference between AAPC 20−49y and AAPC 50+y.

Appendix Figure 15: Average annual percent change (AAPC) for cancer incidence (2003-2017) by UN region, country and age - Colorectal Cancer

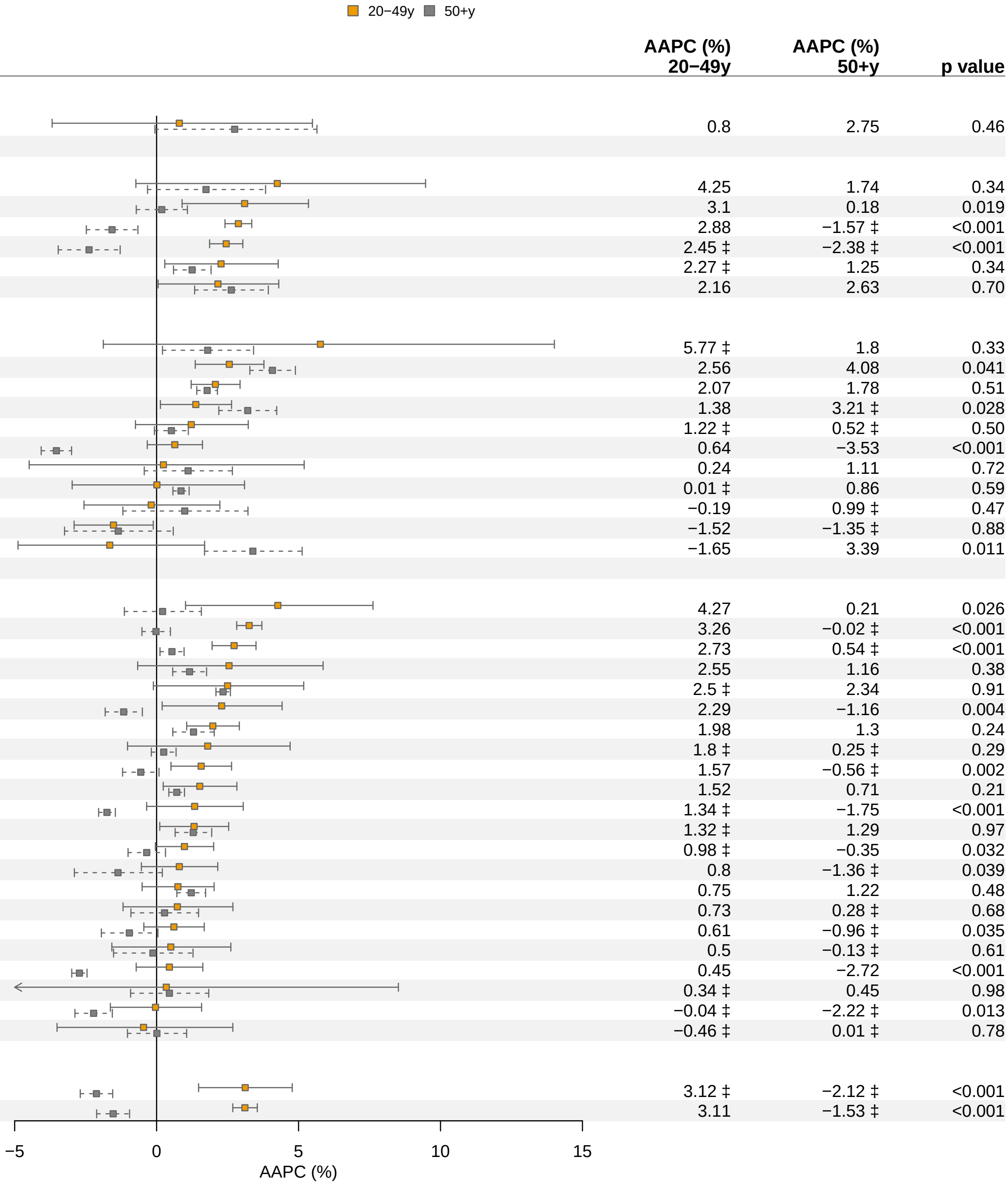

Country	AAPC (%) 20−49y	AAPC (%) 50+y	p value
**Africa**			
Uganda	0.8	2.75	0.46
**Americas**			
Chile	4.25	1.74	0.34
Argentina	3.1	0.18	0.019
Canada	2.88	−1.57 ‡	<0.001
USA	2.45 ‡	−2.38 ‡	<0.001
Colombia	2.27 ‡	1.25	0.34
Ecuador	2.16	2.63	0.70
**Asia**			
Kuwait	5.77 ‡	1.8	0.33
Thailand	2.56	4.08	0.041
Türkiye	2.07	1.78	0.51
India	1.38	3.21 ‡	0.028
Republic of Korea	1.22 ‡	0.52 ‡	0.50
Israel	0.64	−3.53	<0.001
Qatar	0.24	1.11	0.72
China	0.01 ‡	0.86	0.59
Cyprus	−0.19	0.99 ‡	0.47
Philippines	−1.52	−1.35 ‡	0.88
Bahrain	−1.65	3.39	0.011
**Europe**			
Iceland	4.27	0.21	0.026
UK	3.26	−0.02 ‡	<0.001
Sweden	2.73	0.54 ‡	<0.001
Estonia	2.55	1.16	0.38
Belarus	2.5 ‡	2.34	0.91
Switzerland	2.29	−1.16	0.004
Denmark	1.98	1.3	0.24
Norway	1.8 ‡	0.25 ‡	0.29
Ireland	1.57	−0.56 ‡	0.002
Finland	1.52	0.71	0.21
Germany	1.34 ‡	−1.75	<0.001
The Netherlands	1.32 ‡	1.29	0.97
France	0.98 ‡	−0.35	0.032
Slovenia	0.8	−1.36 ‡	0.039
Croatia	0.75	1.22	0.48
Poland	0.73	0.28 ‡	0.68
Italy	0.61	−0.96 ‡	0.035
Latvia	0.5	−0.13 ‡	0.61
Austria	0.45	−2.72	<0.001
Malta	0.34 ‡	0.45	0.98
Czechia	−0.04 ‡	−2.22 ‡	0.013
Lithuania	−0.46 ‡	0.01 ‡	0.78
**Oceania**			
New Zealand	3.12 ‡	−2.12 ‡	<0.001
Australia	3.11	−1.53 ‡	<0.001

‡ best fitted Joinpoint model > 0 joins; * p−value for a difference between AAPC 20−49y and AAPC 50+y.

Appendix Figure 16: Average annual percent change (AAPC) for cancer incidence (2003-2017) by UN region, country and age - Endometrial Cancer

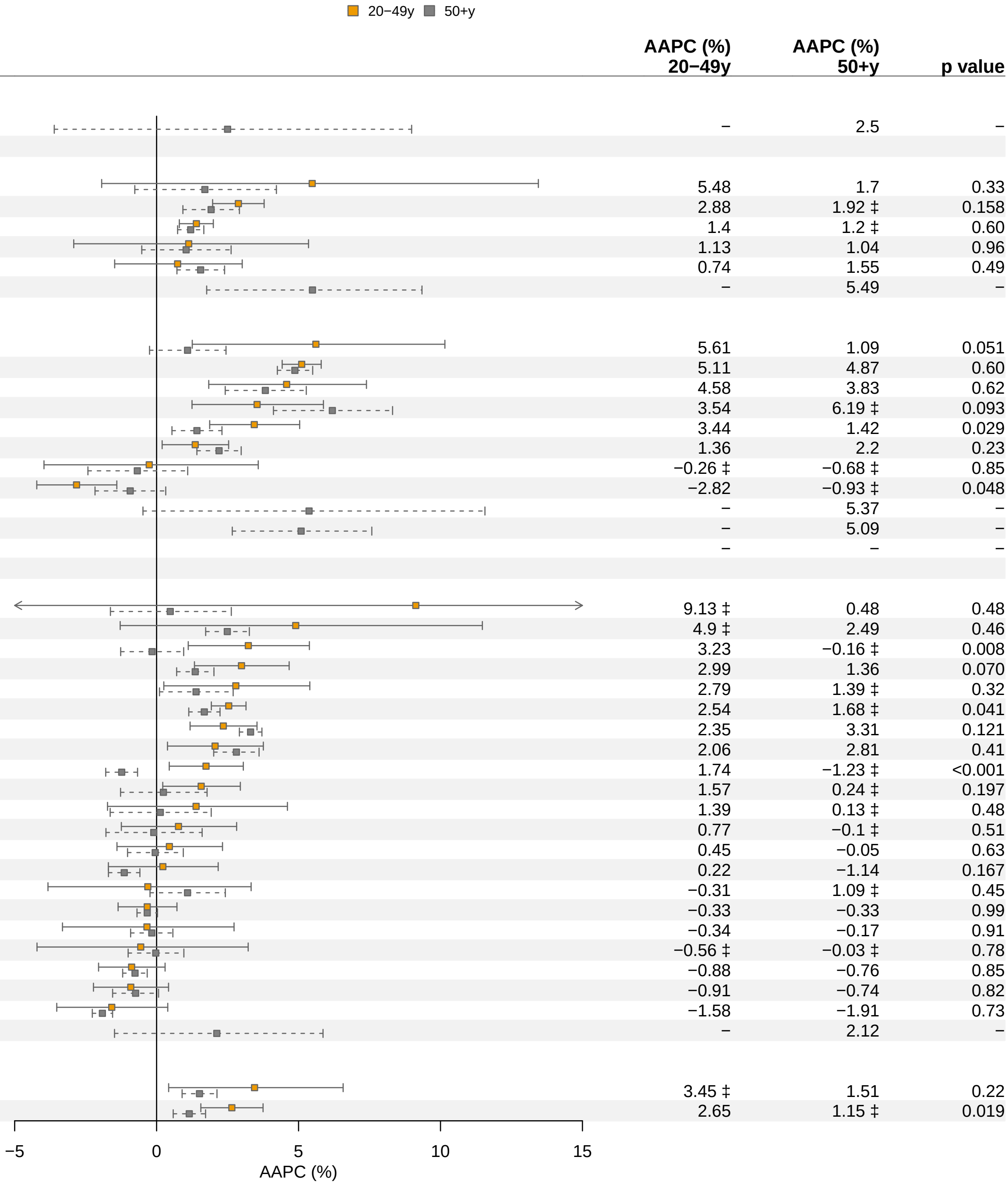

Country	AAPC (%) 20−49y	AAPC (%) 50+y	p value
**Africa**			
Uganda	−	2.5	−
**Americas**			
Ecuador	5.48	1.7	0.33
Canada	2.88	1.92 ‡	0.158
USA	1.4	1.2 ‡	0.60
Argentina	1.13	1.04	0.96
Colombia	0.74	1.55	0.49
Chile	−	5.49	−
**Asia**			
Cyprus	5.61	1.09	0.051
Republic of Korea	5.11	4.87	0.60
Thailand	4.58	3.83	0.62
India	3.54	6.19 ‡	0.093
China	3.44	1.42	0.029
Türkiye	1.36	2.2	0.23
Philippines	−0.26 ‡	−0.68 ‡	0.85
Israel	−2.82	−0.93 ‡	0.048
Bahrain	−	5.37	−
Kuwait	−	5.09	−
Qatar	−	−	−
**Europe**			
Malta	9.13 ‡	0.48	0.48
Croatia	4.9 ‡	2.49	0.46
Denmark	3.23	−0.16 ‡	0.008
Lithuania	2.99	1.36	0.070
Estonia	2.79	1.39 ‡	0.32
UK	2.54	1.68 ‡	0.041
Belarus	2.35	3.31	0.121
Ireland	2.06	2.81	0.41
Sweden	1.74	−1.23 ‡	<0.001
Germany	1.57	0.24 ‡	0.197
Switzerland	1.39	0.13 ‡	0.48
Slovenia	0.77	−0.1 ‡	0.51
Italy	0.45	−0.05	0.63
Finland	0.22	−1.14	0.167
Poland	−0.31	1.09 ‡	0.45
Czechia	−0.33	−0.33	0.99
Latvia	−0.34	−0.17	0.91
France	−0.56 ‡	−0.03 ‡	0.78
The Netherlands	−0.88	−0.76	0.85
Norway	−0.91	−0.74	0.82
Austria	−1.58	−1.91	0.73
Iceland	−	2.12	−
**Oceania**			
New Zealand	3.45 ‡	1.51	0.22
Australia	2.65	1.15 ‡	0.019

Data for female sex only.
‡ best fitted Joinpoint model > 0 joins; * p−value for a difference between AAPC 20−49y and AAPC 50+y.

Appendix Figure 17: Average annual percent change (AAPC) for cancer incidence (2003-2017) by UN region, country and age - Gallbladder Cancer

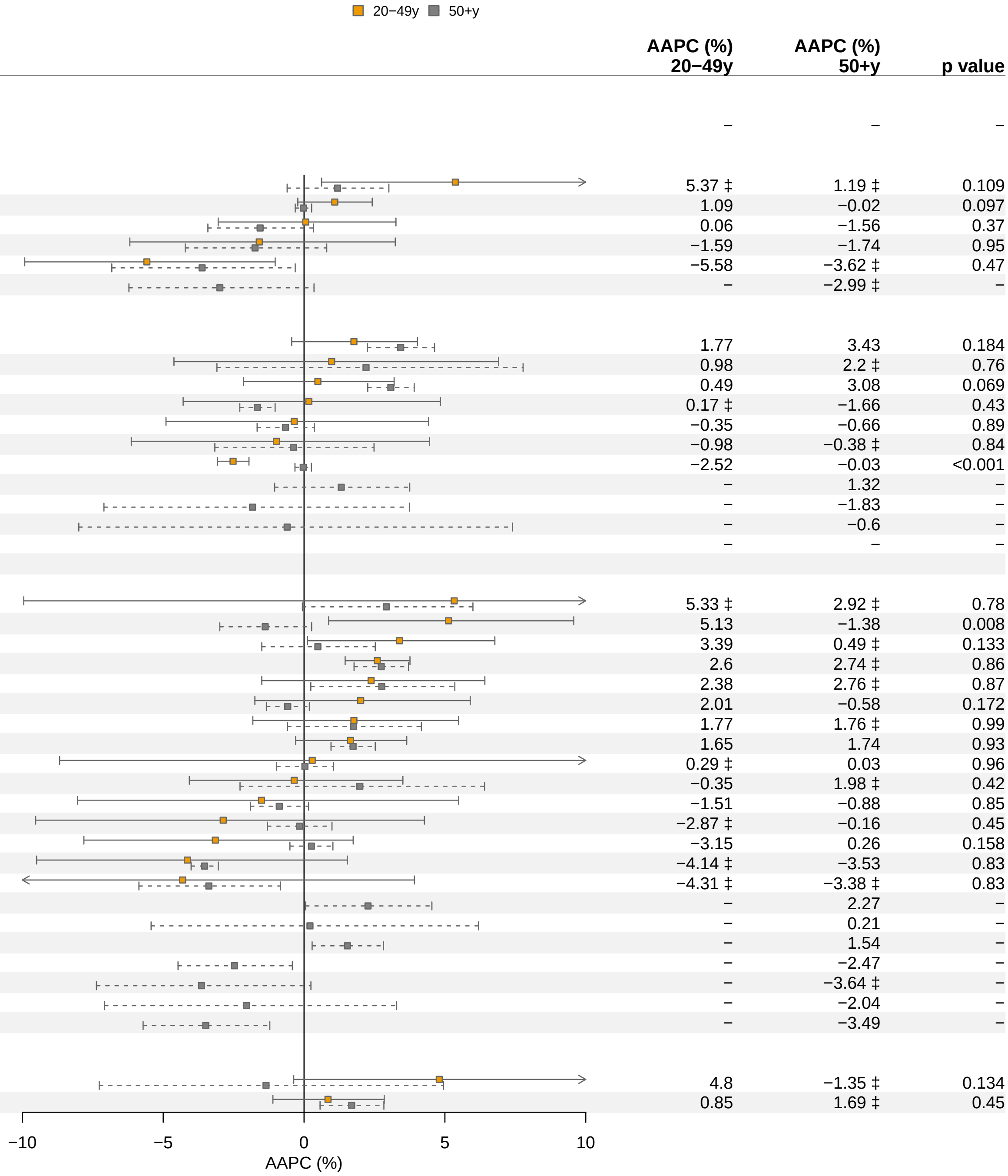

Country	AAPC (%) 20−49y	AAPC (%) 50+y	p value
**Africa**			
Uganda	–	–	–
**Americas**			
Canada	5.37 ‡	1.19 ‡	0.109
USA	1.09	−0.02	0.097
Colombia	0.06	−1.56	0.37
Ecuador	−1.59	−1.74	0.95
Chile	−5.58	−3.62 ‡	0.47
Argentina	–	−2.99 ‡	–
**Asia**			
India	1.77	3.43	0.184
Philippines	0.98	2.2 ‡	0.76
Thailand	0.49	3.08	0.069
China	0.17 ‡	−1.66	0.43
Türkiye	−0.35	−0.66	0.89
Israel	−0.98	−0.38 ‡	0.84
Republic of Korea	−2.52	−0.03	<0.001
Cyprus	–	1.32	–
Kuwait	–	−1.83	–
Qatar	–	−0.6	–
Bahrain	–	–	–
**Europe**			
Slovenia	5.33 ‡	2.92 ‡	0.78
Switzerland	5.13	−1.38	0.008
Sweden	3.39	0.49 ‡	0.133
UK	2.6	2.74 ‡	0.86
Belarus	2.38	2.76 ‡	0.87
Austria	2.01	−0.58	0.172
Denmark	1.77	1.76 ‡	0.99
The Netherlands	1.65	1.74	0.93
Finland	0.29 ‡	0.03	0.96
Norway	−0.35	1.98 ‡	0.42
Croatia	−1.51	−0.88	0.85
Germany	−2.87 ‡	−0.16	0.45
France	−3.15	0.26	0.158
Czechia	−4.14 ‡	−3.53	0.83
Italy	−4.31 ‡	−3.38 ‡	0.83
Estonia	–	2.27	–
Iceland	–	0.21	–
Ireland	–	1.54	–
Latvia	–	−2.47	–
Lithuania	–	−3.64 ‡	–
Malta	–	−2.04	–
Poland	–	−3.49	–
**Oceania**			
New Zealand	4.8	−1.35 ‡	0.134
Australia	0.85	1.69 ‡	0.45

‡ best fitted Joinpoint model > 0 joins; * p−value for a difference between AAPC 20−49y and AAPC 50+y.

Appendix Figure 18: Average annual percent change (AAPC) for cancer incidence (2003-2017) by UN region, country and age - Kidney Cancer

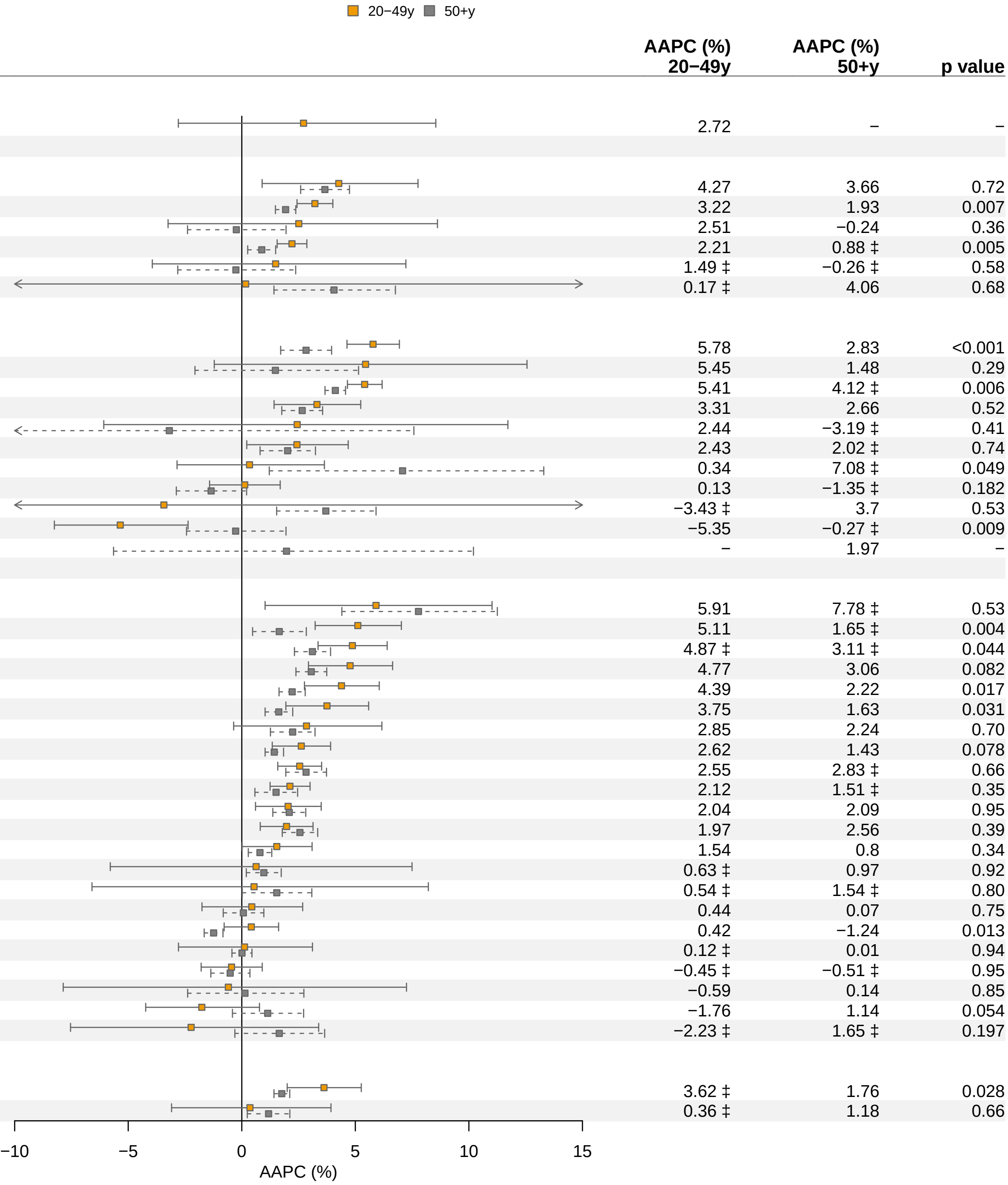

Country	AAPC (%) 20−49y	AAPC (%) 50+y	p value
**Africa**			
Uganda	2.72	–	–
**Americas**			
Colombia	4.27	3.66	0.72
Canada	3.22	1.93	0.007
Chile	2.51	−0.24	0.36
USA	2.21	0.88 ‡	0.005
Argentina	1.49 ‡	−0.26 ‡	0.58
Ecuador	0.17 ‡	4.06	0.68
**Asia**			
Türkiye	5.78	2.83	<0.001
Kuwait	5.45	1.48	0.29
Republic of Korea	5.41	4.12 ‡	0.006
India	3.31	2.66	0.52
Bahrain	2.44	−3.19 ‡	0.41
China	2.43	2.02 ‡	0.74
Thailand	0.34	7.08 ‡	0.049
Israel	0.13	−1.35 ‡	0.182
Cyprus	−3.43 ‡	3.7	0.53
Philippines	−5.35	−0.27 ‡	0.009
Qatar	–	1.97	–
**Europe**			
Malta	5.91	7.78 ‡	0.53
Ireland	5.11	1.65 ‡	0.004
UK	4.87 ‡	3.11 ‡	0.044
Denmark	4.77	3.06	0.082
Norway	4.39	2.22	0.017
Sweden	3.75	1.63	0.031
Latvia	2.85	2.24	0.70
France	2.62	1.43	0.078
Belarus	2.55	2.83 ‡	0.66
The Netherlands	2.12	1.51 ‡	0.35
Italy	2.04	2.09	0.95
Croatia	1.97	2.56	0.39
Finland	1.54	0.8	0.34
Switzerland	0.63 ‡	0.97	0.92
Slovenia	0.54 ‡	1.54 ‡	0.80
Lithuania	0.44	0.07	0.75
Austria	0.42	−1.24	0.013
Germany	0.12 ‡	0.01	0.94
Czechia	−0.45 ‡	−0.51 ‡	0.95
Iceland	−0.59	0.14	0.85
Poland	−1.76	1.14	0.054
Estonia	−2.23 ‡	1.65 ‡	0.197
**Oceania**			
Australia	3.62 ‡	1.76	0.028
New Zealand	0.36 ‡	1.18	0.66

‡ best fitted Joinpoint model > 0 joins; * p−value for a difference between AAPC 20−49y and AAPC 50+y.

Appendix Figure 19: Average annual percent change (AAPC) for cancer incidence (2003-2017) by UN region, country and age - Leukaemia

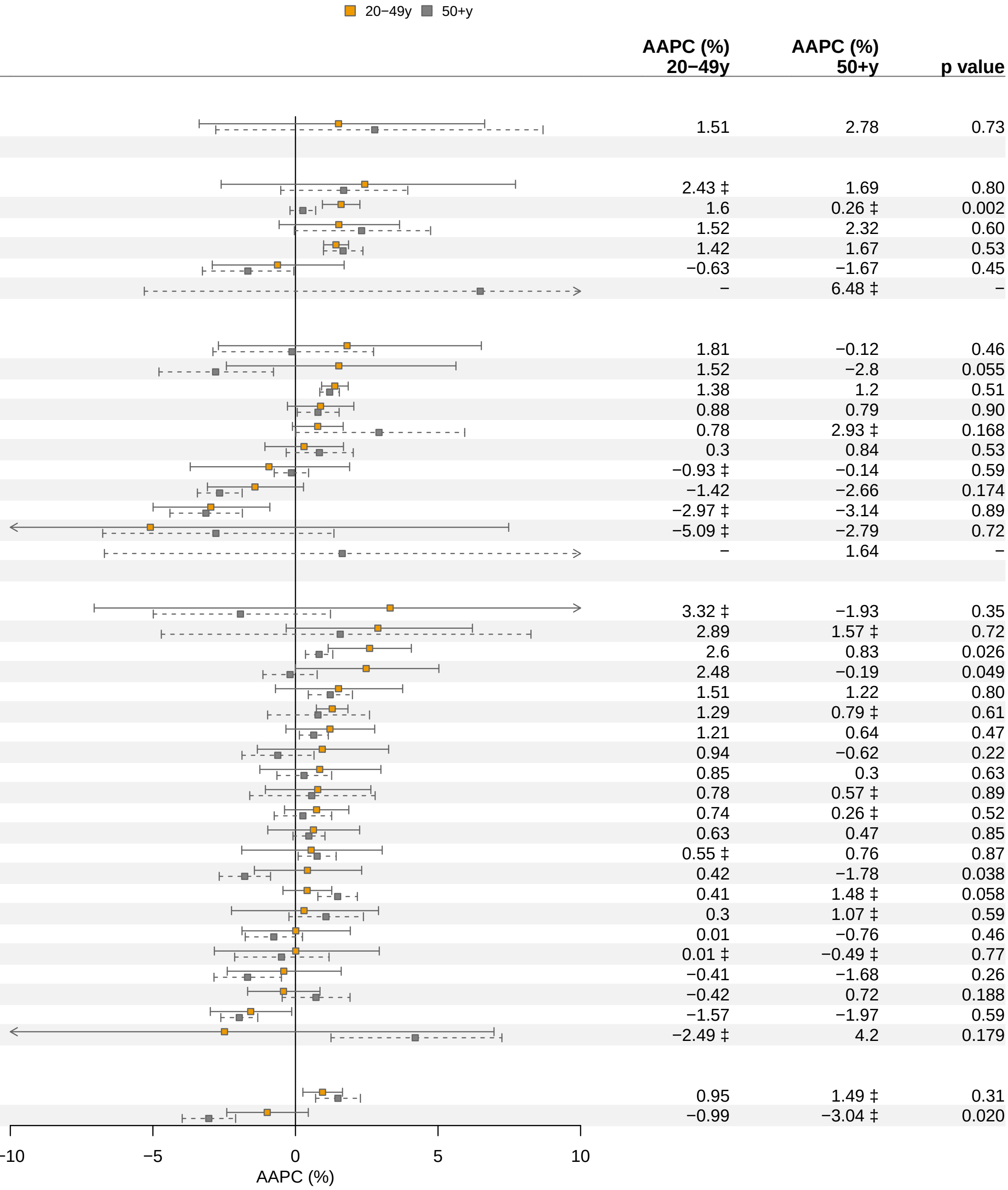

Country	AAPC (%) 20−49y	AAPC (%) 50+y	p value
**Africa**			
Uganda	1.51	2.78	0.73
**Americas**			
Colombia	2.43 ‡	1.69	0.80
USA	1.6	0.26 ‡	0.002
Ecuador	1.52	2.32	0.60
Canada	1.42	1.67	0.53
Argentina	−0.63	−1.67	0.45
Chile	−	6.48 ‡	−
**Asia**			
Kuwait	1.81	−0.12	0.46
Cyprus	1.52	−2.8	0.055
Republic of Korea	1.38	1.2	0.51
Türkiye	0.88	0.79	0.90
India	0.78	2.93 ‡	0.168
Thailand	0.3	0.84	0.53
China	−0.93 ‡	−0.14	0.59
Israel	−1.42	−2.66	0.174
Philippines	−2.97 ‡	−3.14	0.89
Bahrain	−5.09 ‡	−2.79	0.72
Qatar	−	1.64	−
**Europe**			
Iceland	3.32 ‡	−1.93	0.35
Latvia	2.89	1.57 ‡	0.72
France	2.6	0.83	0.026
Estonia	2.48	−0.19	0.049
Norway	1.51	1.22	0.80
UK	1.29	0.79 ‡	0.61
Denmark	1.21	0.64	0.47
Poland	0.94	−0.62	0.22
Finland	0.85	0.3	0.63
Lithuania	0.78	0.57 ‡	0.89
Czechia	0.74	0.26 ‡	0.52
Sweden	0.63	0.47	0.85
Germany	0.55 ‡	0.76	0.87
Ireland	0.42	−1.78	0.038
The Netherlands	0.41	1.48 ‡	0.058
Slovenia	0.3	1.07 ‡	0.59
Switzerland	0.01	−0.76	0.46
Croatia	0.01 ‡	−0.49 ‡	0.77
Italy	−0.41	−1.68	0.26
Belarus	−0.42	0.72	0.188
Austria	−1.57	−1.97	0.59
Malta	−2.49 ‡	4.2	0.179
**Oceania**			
Australia	0.95	1.49 ‡	0.31
New Zealand	−0.99	−3.04 ‡	0.020

‡ best fitted Joinpoint model > 0 joins; * p−value for a difference between AAPC 20−49y and AAPC 50+y.

Appendix Figure 20: Average annual percent change (AAPC) for cancer incidence (2003-2017) by UN region, country and age - Liver Cancer

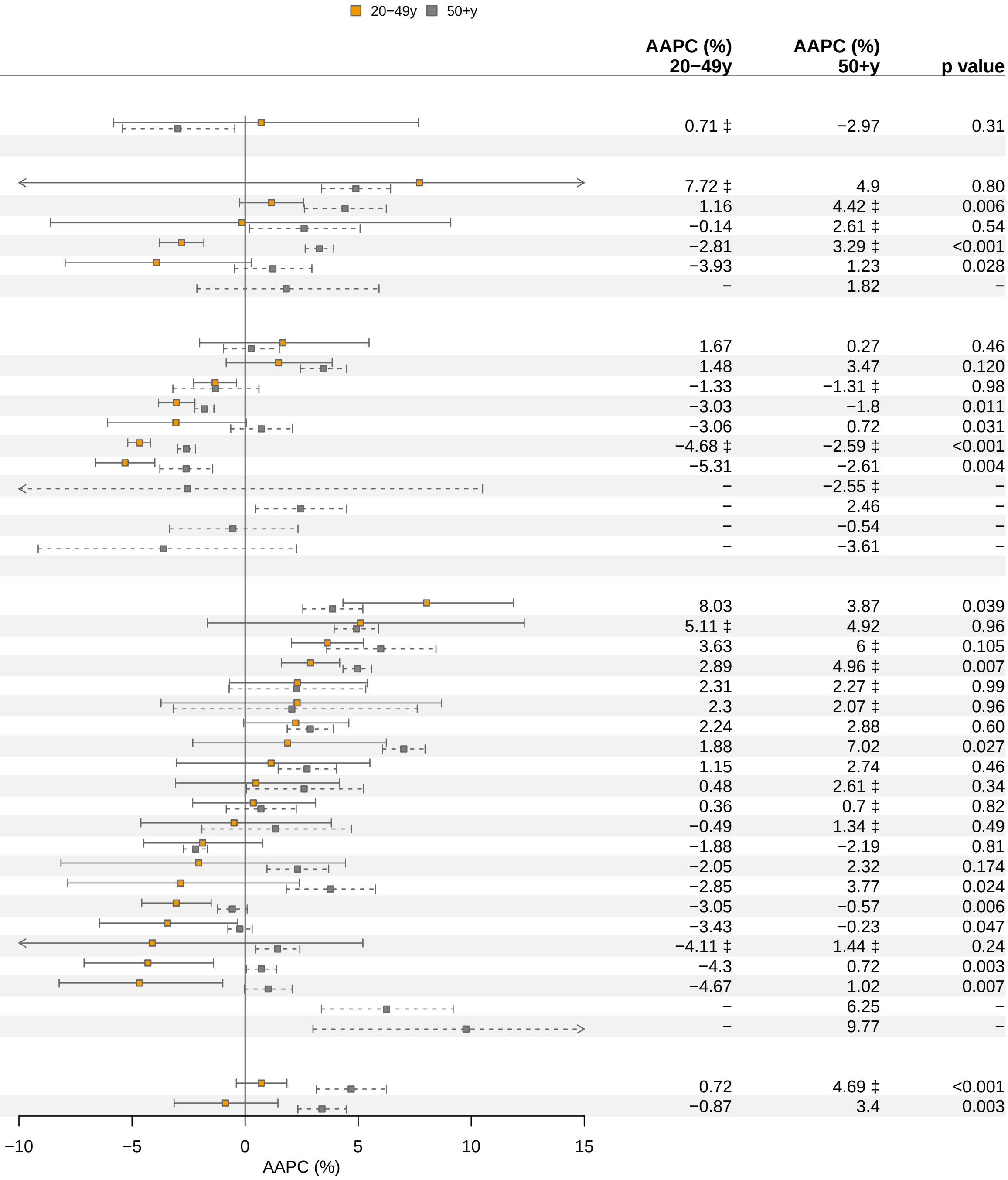

Country	AAPC (%) 20−49y	AAPC (%) 50+y	p value
**Africa**			
Uganda	0.71 ‡	−2.97	0.31
**Americas**			
Argentina	7.72 ‡	4.9	0.80
Canada	1.16	4.42 ‡	0.006
Ecuador	−0.14	2.61 ‡	0.54
USA	−2.81	3.29 ‡	<0.001
Colombia	−3.93	1.23	0.028
Chile	−	1.82	−
**Asia**			
Israel	1.67	0.27	0.46
India	1.48	3.47	0.120
Thailand	−1.33	−1.31 ‡	0.98
China	−3.03	−1.8	0.011
Türkiye	−3.06	0.72	0.031
Republic of Korea	−4.68 ‡	−2.59 ‡	<0.001
Philippines	−5.31	−2.61	0.004
Bahrain	−	−2.55 ‡	−
Cyprus	−	2.46	−
Kuwait	−	−0.54	−
Qatar	−	−3.61	−
**Europe**			
Lithuania	8.03	3.87	0.039
Ireland	5.11 ‡	4.92	0.96
The Netherlands	3.63	6 ‡	0.105
UK	2.89	4.96 ‡	0.007
Sweden	2.31	2.27 ‡	0.99
Poland	2.3	2.07 ‡	0.96
Belarus	2.24	2.88	0.60
Norway	1.88	7.02	0.027
Latvia	1.15	2.74	0.46
Finland	0.48	2.61 ‡	0.34
Germany	0.36	0.7 ‡	0.82
Denmark	−0.49	1.34 ‡	0.49
Italy	−1.88	−2.19	0.81
Slovenia	−2.05	2.32	0.174
Estonia	−2.85	3.77	0.024
Austria	−3.05	−0.57	0.006
Czechia	−3.43	−0.23	0.047
France	−4.11 ‡	1.44 ‡	0.24
Croatia	−4.3	0.72	0.003
Switzerland	−4.67	1.02	0.007
Iceland	−	6.25	−
Malta	−	9.77	−
**Oceania**			
Australia	0.72	4.69 ‡	<0.001
New Zealand	−0.87	3.4	0.003

‡ best fitted Joinpoint model > 0 joins; * p−value for a difference between AAPC 20−49y and AAPC 50+y.

Appendix Figure 21: Average annual percent change (AAPC) for cancer incidence (2003-2017) by UN region, country and age - Oesophagal Cancer

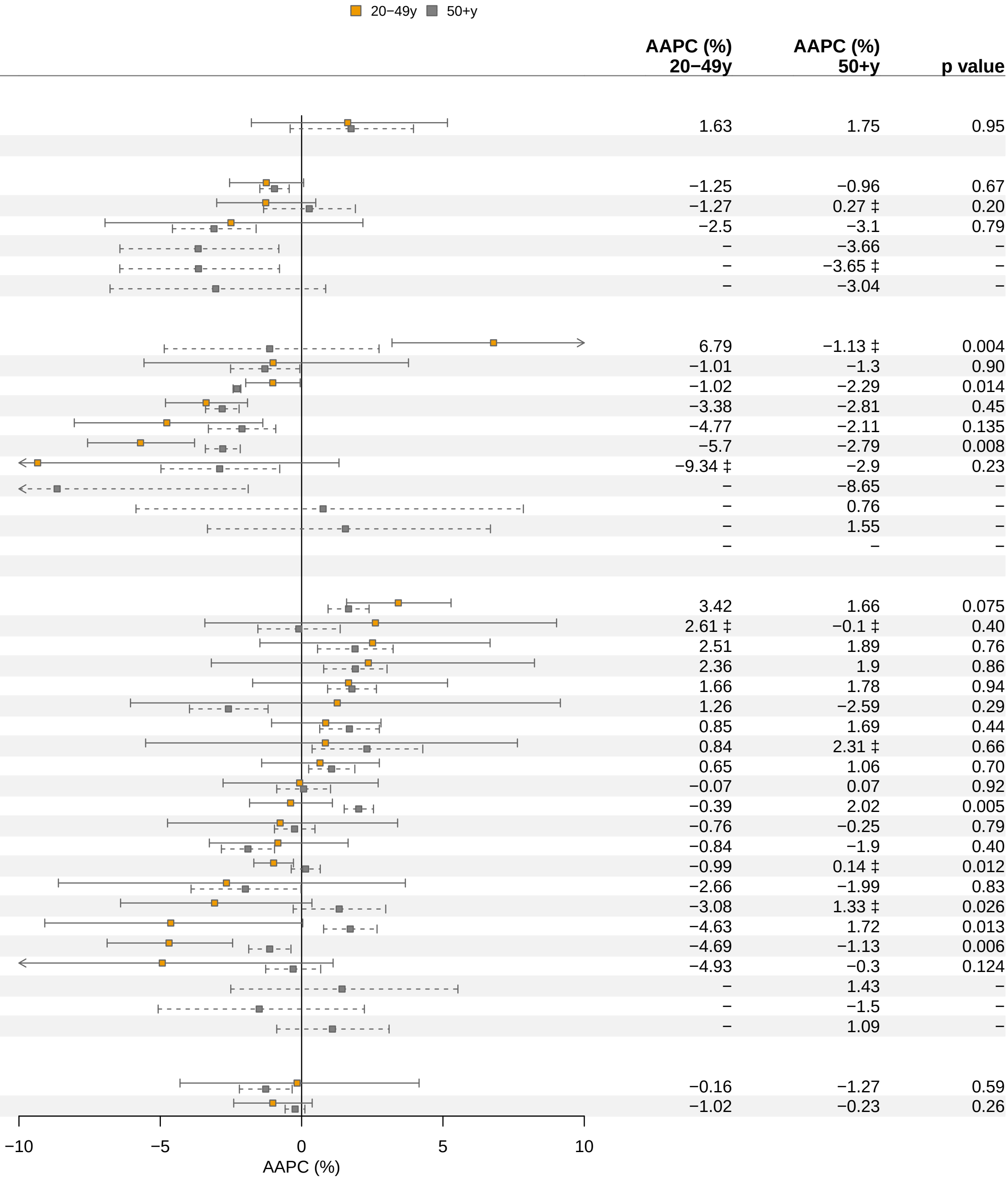

Country	AAPC (%) 20−49y	AAPC (%) 50+y	p value
**Africa**			
Uganda	1.63	1.75	0.95
**Americas**			
USA	−1.25	−0.96	0.67
Canada	−1.27	0.27 ‡	0.20
Argentina	−2.5	−3.1	0.79
Chile	−	−3.66	−
Colombia	−	−3.65 ‡	−
Ecuador	−	−3.04	−
**Asia**			
Thailand	6.79	−1.13 ‡	0.004
Israel	−1.01	−1.3	0.90
Republic of Korea	−1.02	−2.29	0.014
India	−3.38	−2.81	0.45
Türkiye	−4.77	−2.11	0.135
China	−5.7	−2.79	0.008
Philippines	−9.34 ‡	−2.9	0.23
Bahrain	−	−8.65	−
Cyprus	−	0.76	−
Kuwait	−	1.55	−
Qatar	−	−	−
**Europe**			
Belarus	3.42	1.66	0.075
Ireland	2.61 ‡	−0.1 ‡	0.40
Latvia	2.51	1.89	0.76
Estonia	2.36	1.9	0.86
Norway	1.66	1.78	0.94
Italy	1.26	−2.59	0.29
Lithuania	0.85	1.69	0.44
Finland	0.84	2.31 ‡	0.66
Germany	0.65	1.06	0.70
Sweden	−0.07	0.07	0.92
The Netherlands	−0.39	2.02	0.005
Austria	−0.76	−0.25	0.79
Croatia	−0.84	−1.9	0.40
UK	−0.99	0.14 ‡	0.012
Slovenia	−2.66	−1.99	0.83
Czechia	−3.08	1.33 ‡	0.026
Denmark	−4.63	1.72	0.013
France	−4.69	−1.13	0.006
Switzerland	−4.93	−0.3	0.124
Iceland	−	1.43	−
Malta	−	−1.5	−
Poland	−	1.09	−
**Oceania**			
New Zealand	−0.16	−1.27	0.59
Australia	−1.02	−0.23	0.26

‡ best fitted Joinpoint model > 0 joins; * p−value for a difference between AAPC 20−49y and AAPC 50+y.

Appendix Figure 22: Average annual percent change (AAPC) for cancer incidence (2003-2017) by UN region, country and age - Oral Cancer

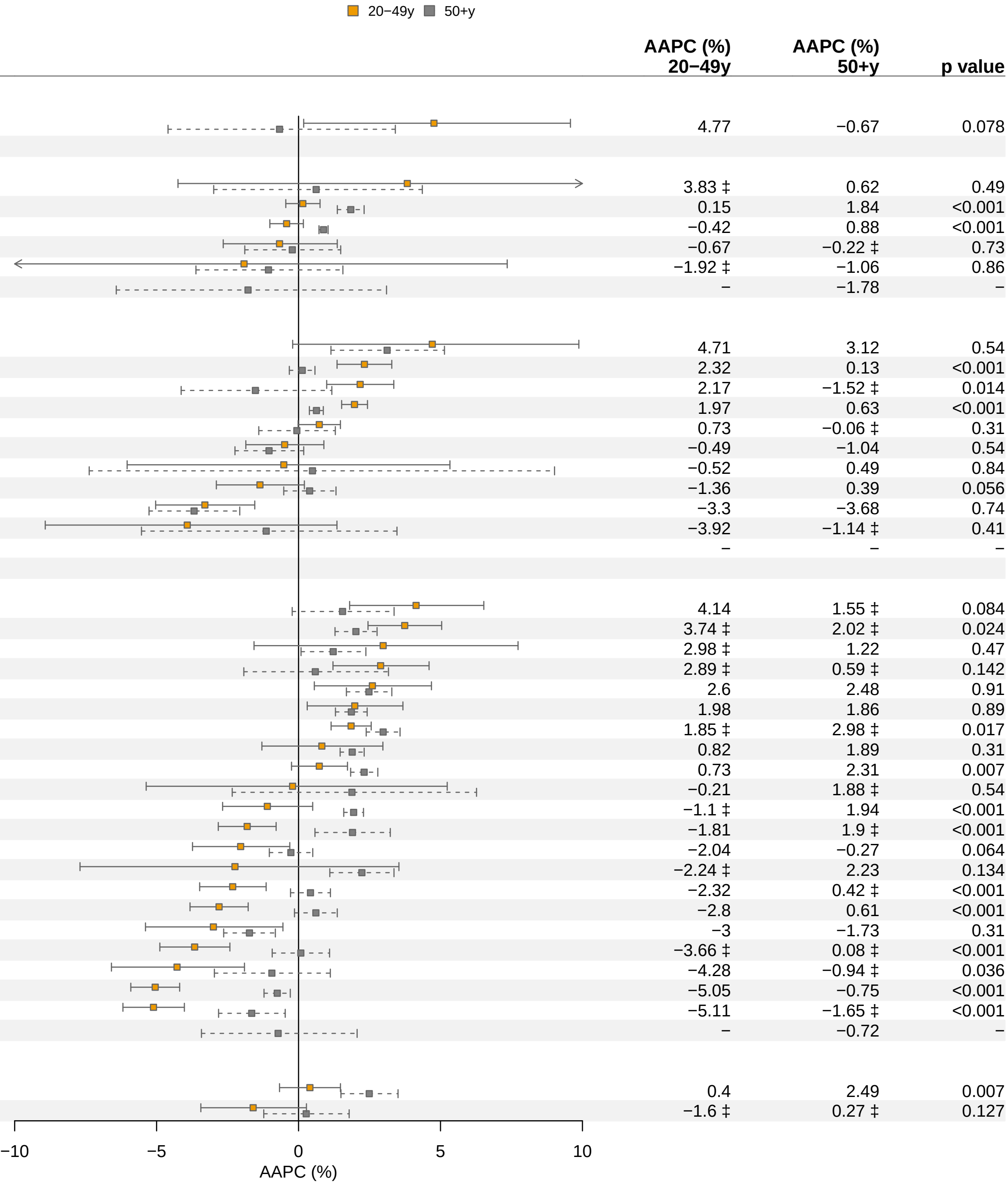

Country	AAPC (%) 20−49y	AAPC (%) 50+y	p value
**Africa**			
Uganda	4.77	−0.67	0.078
**Americas**			
Ecuador	3.83 ‡	0.62	0.49
Canada	0.15	1.84	<0.001
USA	−0.42	0.88	<0.001
Colombia	−0.67	−0.22 ‡	0.73
Argentina	−1.92 ‡	−1.06	0.86
Chile	–	−1.78	–
**Asia**			
Cyprus	4.71	3.12	0.54
India	2.32	0.13	<0.001
Thailand	2.17	−1.52 ‡	0.014
Republic of Korea	1.97	0.63	<0.001
China	0.73	−0.06 ‡	0.31
Türkiye	−0.49	−1.04	0.54
Bahrain	−0.52	0.49	0.84
Israel	−1.36	0.39	0.056
Philippines	−3.3	−3.68	0.74
Kuwait	−3.92	−1.14 ‡	0.41
Qatar	–	–	–
**Europe**			
Latvia	4.14	1.55 ‡	0.084
Belarus	3.74 ‡	2.02 ‡	0.024
Estonia	2.98 ‡	1.22	0.47
Lithuania	2.89 ‡	0.59 ‡	0.142
Ireland	2.6	2.48	0.91
Norway	1.98	1.86	0.89
UK	1.85 ‡	2.98 ‡	0.017
Finland	0.82	1.89	0.31
Sweden	0.73	2.31	0.007
Malta	−0.21	1.88 ‡	0.54
Czechia	−1.1 ‡	1.94	<0.001
Denmark	−1.81	1.9 ‡	<0.001
Slovenia	−2.04	−0.27	0.064
Poland	−2.24 ‡	2.23	0.134
The Netherlands	−2.32	0.42 ‡	<0.001
Austria	−2.8	0.61	<0.001
Italy	−3	−1.73	0.31
Germany	−3.66 ‡	0.08 ‡	<0.001
Switzerland	−4.28	−0.94 ‡	0.036
France	−5.05	−0.75	<0.001
Croatia	−5.11	−1.65 ‡	<0.001
Iceland	–	−0.72	–
**Oceania**			
New Zealand	0.4	2.49	0.007
Australia	−1.6 ‡	0.27 ‡	0.127

‡ best fitted Joinpoint model > 0 joins; * p−value for a difference between AAPC 20−49y and AAPC 50+y.

Appendix Figure 23: Average annual percent change (AAPC) for cancer incidence (2003-2017) by UN region, country and age - Pancreatic Cancer

■ 20−49y ■ 50+y

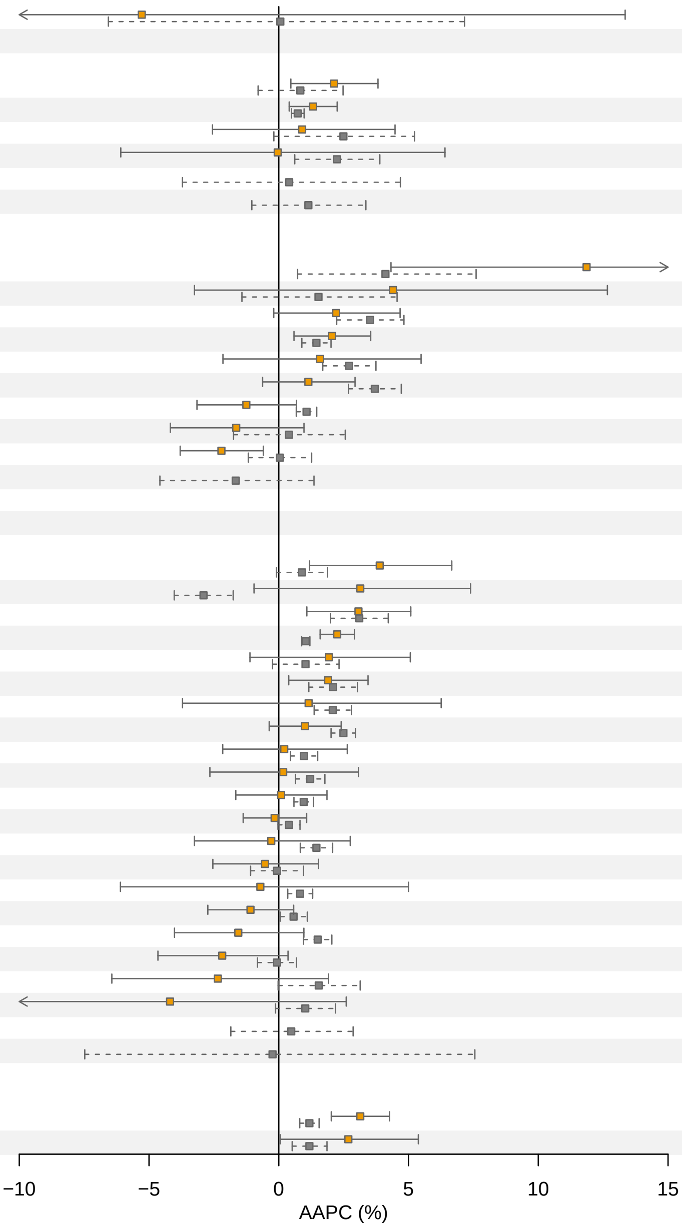

Country	AAPC (%) 20−49y	AAPC (%) 50+y	p value
**Africa**			
Uganda	−5.28 ‡	0.06	0.57
**Americas**			
Canada	2.13 ‡	0.83 ‡	0.28
USA	1.32	0.73	0.21
Argentina	0.9	2.49 ‡	0.47
Colombia	−0.04 ‡	2.24 ‡	0.49
Chile	−	0.4	−
Ecuador	−	1.14	−
**Asia**			
Cyprus	11.86	4.11 ‡	0.066
Kuwait	4.4	1.53	0.48
Thailand	2.21	3.52	0.33
Republic of Korea	2.05 ‡	1.45 ‡	0.45
Türkiye	1.59 ‡	2.71	0.58
India	1.14	3.7	0.018
China	−1.25	1.07	0.024
Philippines	−1.64	0.39 ‡	0.23
Israel	−2.21	0.04 ‡	0.030
Bahrain	−	−1.66	−
Qatar	−	−	−
**Europe**			
Switzerland	3.89	0.89 ‡	0.042
Poland	3.14	−2.9	0.008
France	3.07	3.1 ‡	0.98
UK	2.25	1.04	0.002
Ireland	1.93	1.03 ‡	0.58
The Netherlands	1.9	2.09 ‡	0.83
Sweden	1.15 ‡	2.08 ‡	0.72
Belarus	1.01	2.49	0.049
Lithuania	0.21	0.97	0.52
Italy	0.17	1.21	0.46
Austria	0.09	0.96	0.32
Czechia	−0.16	0.39	0.38
Latvia	−0.29	1.45	0.25
Denmark	−0.53	−0.07 ‡	0.68
Germany	−0.71 ‡	0.82	0.59
Finland	−1.09	0.57	0.059
Croatia	−1.56	1.5	0.024
Norway	−2.18	−0.07	0.108
Slovenia	−2.35	1.54 ‡	0.087
Estonia	−4.19 ‡	1.02	0.134
Iceland	−	0.48	−
Malta	−	−0.24 ‡	−
**Oceania**			
Australia	3.14	1.18	0.003
New Zealand	2.68	1.18	0.26

‡ best fitted Joinpoint model > 0 joins; * p−value for a difference between AAPC 20−49y and AAPC 50+y.

Appendix Figure 24: Average annual percent change (AAPC) for cancer incidence (2003-2017) by UN region, country and age - Prostate Cancer

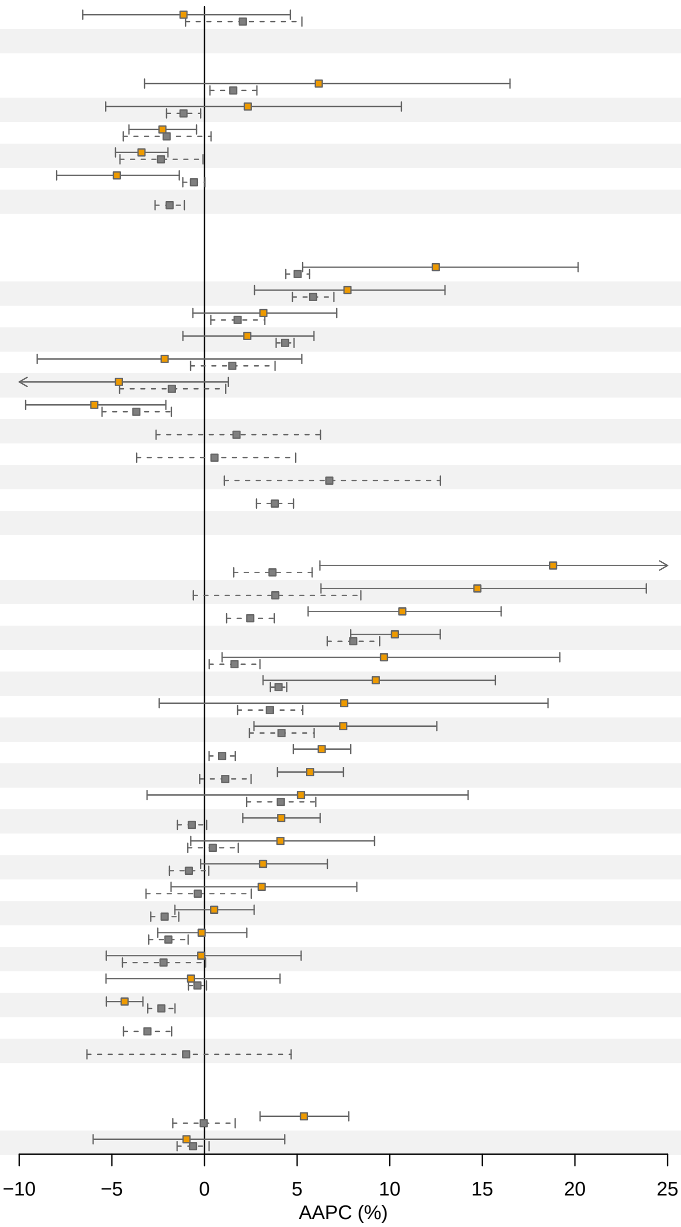

Country	AAPC (%) 20−49y	AAPC (%) 50+y	p value
**Africa**			
Uganda	−1.13	2.07 ‡	0.32
**Americas**			
Ecuador	6.17 ‡	1.55	0.35
Argentina	2.34	−1.13	0.36
Canada	−2.27 ‡	−2.04 ‡	0.88
USA	−3.4 ‡	−2.35 ‡	0.43
Colombia	−4.73	−0.57	0.021
Chile	−	−1.88	−
**Asia**			
China	12.49	5.03	0.044
Republic of Korea	7.72 ‡	5.86 ‡	0.49
Türkiye	3.18	1.79 ‡	0.48
India	2.31	4.35	0.24
Cyprus	−2.15	1.5 ‡	0.32
Philippines	−4.62	−1.76 ‡	0.37
Israel	−5.95	−3.68 ‡	0.28
Bahrain	−	1.73	−
Kuwait	−	0.54 ‡	−
Qatar	−	6.74	−
Thailand	−	3.8	−
**Europe**			
Estonia	18.82 ‡	3.67 ‡	0.019
Lithuania	14.73 ‡	3.82 ‡	0.026
Czechia	10.68 ‡	2.47 ‡	0.002
Belarus	10.28	8.03 ‡	0.107
Denmark	9.69 ‡	1.62 ‡	0.075
Latvia	9.25	4	0.087
Slovenia	7.54 ‡	3.53 ‡	0.45
Croatia	7.49	4.16 ‡	0.190
UK	6.33 ‡	0.95 ‡	<0.001
Norway	5.7	1.12 ‡	<0.001
Poland	5.21	4.12 ‡	0.80
Sweden	4.14	−0.68	<0.001
Ireland	4.1 ‡	0.45 ‡	0.158
The Netherlands	3.16 ‡	−0.84 ‡	0.026
Switzerland	3.09 ‡	−0.36 ‡	0.24
Germany	0.52	−2.15	0.023
Finland	−0.15	−1.95	0.163
France	−0.18 ‡	−2.21 ‡	0.48
Italy	−0.73	−0.38	0.88
Austria	−4.31	−2.33 ‡	0.003
Iceland	−	−3.08	−
Malta	−	−0.99 ‡	−
**Oceania**			
Australia	5.37 ‡	−0.04 ‡	<0.001
New Zealand	−0.97 ‡	−0.62	0.89

Data for male sex only.
‡ best fitted Joinpoint model > 0 joins; * p−value for a difference between AAPC 20−49y and AAPC 50+y.

Appendix Figure 25: Average annual percent change (AAPC) for cancer incidence (2003-2017) by UN region, country and age - Stomach Cancer

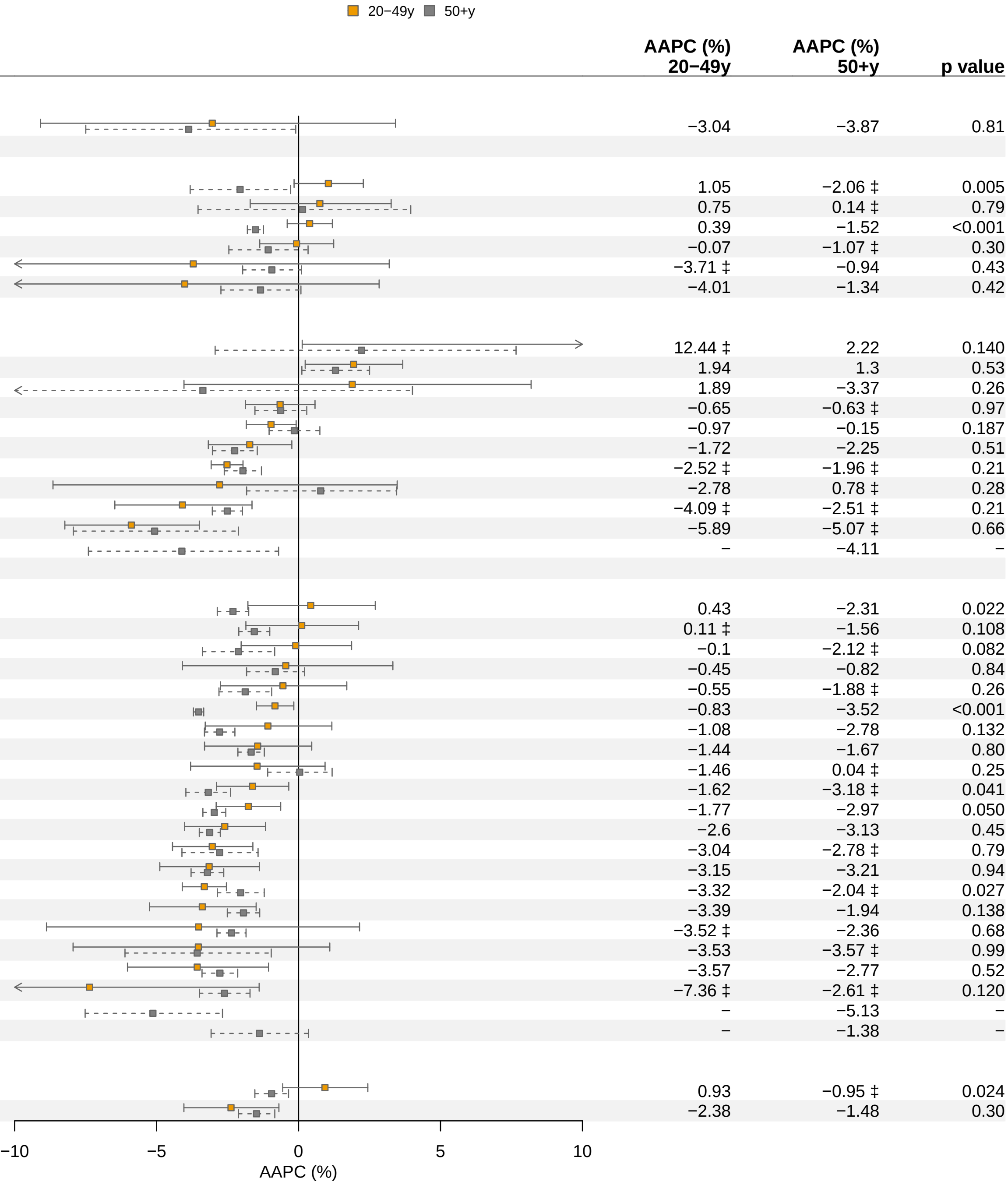

Country	AAPC (%) 20−49y	AAPC (%) 50+y	p value
**Africa**			
Uganda	−3.04	−3.87	0.81
**Americas**			
Colombia	1.05	−2.06 ‡	0.005
Ecuador	0.75	0.14 ‡	0.79
USA	0.39	−1.52	<0.001
Canada	−0.07	−1.07 ‡	0.30
Argentina	−3.71 ‡	−0.94	0.43
Chile	−4.01	−1.34	0.42
**Asia**			
Kuwait	12.44 ‡	2.22	0.140
Thailand	1.94	1.3	0.53
Qatar	1.89	−3.37	0.26
Türkiye	−0.65	−0.63 ‡	0.97
India	−0.97	−0.15	0.187
Israel	−1.72	−2.25	0.51
Republic of Korea	−2.52 ‡	−1.96 ‡	0.21
Cyprus	−2.78	0.78 ‡	0.28
China	−4.09 ‡	−2.51 ‡	0.21
Philippines	−5.89	−5.07 ‡	0.66
Bahrain	−	−4.11	−
**Europe**			
Sweden	0.43	−2.31	0.022
France	0.11 ‡	−1.56	0.108
Ireland	−0.1	−2.12 ‡	0.082
Switzerland	−0.45	−0.82	0.84
Latvia	−0.55	−1.88 ‡	0.26
UK	−0.83	−3.52	<0.001
Norway	−1.08	−2.78	0.132
Germany	−1.44	−1.67	0.80
Denmark	−1.46	0.04 ‡	0.25
The Netherlands	−1.62	−3.18 ‡	0.041
Austria	−1.77	−2.97	0.050
Czechia	−2.6	−3.13	0.45
Croatia	−3.04	−2.78 ‡	0.79
Finland	−3.15	−3.21	0.94
Belarus	−3.32	−2.04 ‡	0.027
Estonia	−3.39	−1.94	0.138
Lithuania	−3.52 ‡	−2.36	0.68
Poland	−3.53	−3.57 ‡	0.99
Italy	−3.57	−2.77	0.52
Slovenia	−7.36 ‡	−2.61 ‡	0.120
Iceland	−	−5.13	−
Malta	−	−1.38	−
**Oceania**			
Australia	0.93	−0.95 ‡	0.024
New Zealand	−2.38	−1.48	0.30

‡ best fitted Joinpoint model > 0 joins; * p−value for a difference between AAPC 20−49y and AAPC 50+y.

Appendix Figure 26: Average annual percent change (AAPC) for cancer incidence (2003-2017) by UN region, country and age - Thyroid Cancer

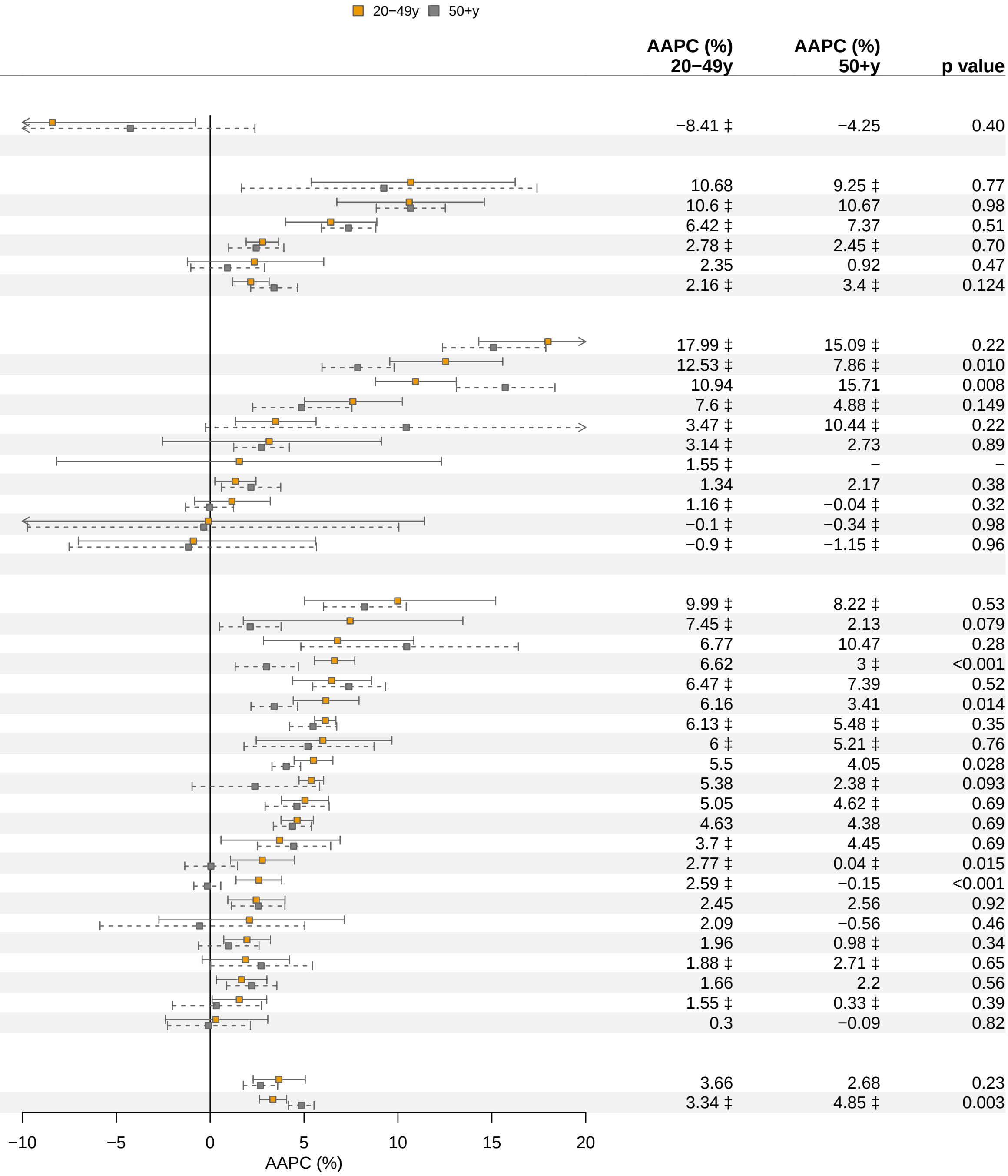

Country	AAPC (%) 20−49y	AAPC (%) 50+y	p value
**Africa**			
Uganda	−8.41 ‡	−4.25	0.40
**Americas**			
Chile	10.68	9.25 ‡	0.77
Ecuador	10.6 ‡	10.67	0.98
Colombia	6.42 ‡	7.37	0.51
USA	2.78 ‡	2.45 ‡	0.70
Argentina	2.35	0.92	0.47
Canada	2.16 ‡	3.4 ‡	0.124
**Asia**			
China	17.99 ‡	15.09 ‡	0.22
Türkiye	12.53 ‡	7.86 ‡	0.010
Cyprus	10.94	15.71	0.008
Republic of Korea	7.6 ‡	4.88 ‡	0.149
Kuwait	3.47 ‡	10.44 ‡	0.22
Thailand	3.14 ‡	2.73	0.89
Qatar	1.55 ‡	–	–
India	1.34	2.17	0.38
Israel	1.16 ‡	−0.04 ‡	0.32
Bahrain	−0.1 ‡	−0.34 ‡	0.98
Philippines	−0.9 ‡	−1.15 ‡	0.96
**Europe**			
Latvia	9.99 ‡	8.22 ‡	0.53
Poland	7.45 ‡	2.13	0.079
Malta	6.77	10.47	0.28
Croatia	6.62	3 ‡	<0.001
Denmark	6.47 ‡	7.39	0.52
Germany	6.16	3.41	0.014
UK	6.13 ‡	5.48 ‡	0.35
Ireland	6 ‡	5.21 ‡	0.76
Sweden	5.5	4.05	0.028
Czechia	5.38	2.38 ‡	0.093
Norway	5.05	4.62 ‡	0.69
The Netherlands	4.63	4.38	0.69
Switzerland	3.7 ‡	4.45	0.69
Austria	2.77 ‡	0.04 ‡	0.015
Belarus	2.59 ‡	−0.15	<0.001
Slovenia	2.45	2.56	0.92
Iceland	2.09	−0.56	0.46
Lithuania	1.96	0.98 ‡	0.34
Finland	1.88 ‡	2.71 ‡	0.65
Italy	1.66	2.2	0.56
France	1.55 ‡	0.33 ‡	0.39
Estonia	0.3	−0.09	0.82
**Oceania**			
New Zealand	3.66	2.68	0.23
Australia	3.34 ‡	4.85 ‡	0.003

‡ best fitted Joinpoint model > 0 joins; * p−value for a difference between AAPC 20−49y and AAPC 50+y.

Appendix Table 1: Source of cancer incidence data by country in GLOBOCAN

Country	Data provider	Method*
Argentina	Mendoza cancer registry	3c
Australia	National cancer registry	1
Austria	National cancer registry	1
Bahrain	Bahrain: Bahraini cancer registry	3c
Belarus	National cancer registry	1
Canada	Canada (Excl Nova Scotia, Northwest Territories, Nunavut, Quebec and Yukon) cancer registry	1
Chile	Valdivia cancer registry	3c
China	Shanghai City, Jiashan County, Zhongshan City, Nangang District, Harbin City cancer registries	2b
Colombia	Cali, Bucaramanga, Manizales, Pasto cancer registries	3c
Croatia	National cancer registry	1
Cyprus	Cyprus cancer registry	1
Czechia	National cancer registry	1
Denmark	National cancer registry (Nordcan database)	1
Ecuador	Quito cancer registry	3c
Estonia	National cancer registry	1
Finland	National cancer registry (Nordcan database)	1
France (metropolitan)	Calvados, Doubs, Haut-Rhin, Isère, Somme, Hérault, Loire-Atlantique, Manche, Vendée cancer registries	3a
Germany	Hamburg, Bremen, Schleswig-Holstein, Saarland cancer registries	3a
Iceland	National cancer registry (Nordcan database)	1
India	Mumbai, Chennai, Barshi, Dindigul Ambilikkai cancer registries	2b
Ireland	National cancer registry	1
Israel	National cancer registry	1
Italy	Trento, Syracuse, Palermo, South Tyrol cancer registries	3a
Kuwait	Kuwait: Kuwaiti cancer registry	1
Latvia	National cancer registry	1
Lithuania	National cancer registry	1
Malta	National cancer registry	1
New Zealand	National cancer registry	1
Norway	National cancer registry (Nordcan database)	1

Phillippines	Manila cancer registry	2b
Poland	Kielce cancer registry	1
Qatar	Qatar: Qatari cancer registry	3a
Republic of Korea	National cancer registry	1
Slovenia	National cancer registry	1
Sweden	National cancer registry (Nordcan database)	1
Switzerland	Geneva, Vaud, Valais, Ticino, Graubünden and Glarus cancer registries	3a
Thailand	Chiang Mai, Khon Kaen, Songkhla, Lampang cancer registries	2b
The Netherlands	National cancer registry	1
Türkiye	Izmir, Antalya cancer registries	2b
Uganda	Kyadondo County cancer registry	2b
UK	Combined data England cancer registry, Wales cancer registry and Scotland cancer registry	1
USA	SEER 9 (San Francisco-Oakland and Los Angeles Cancer Registries (California), Connecticut Tumor Registry, Atlanta Tumor Registry (Georgia), Hawaii Tumor Registry, State Health Registry of Iowa, Detroit Cancer Registry (Michigan), New Mexico Tumor Registry, Utah Cancer Registry, Seattle-Puget Sound Tumor Registry (Washington State)	1

*Method for estimating cancer incidence:

1 National (or sub-national with coverage greater than 50%) rates

2b Weighted/simple average of the most recent sub-national rates

3a Estimated from national mortality estimates by modelling, using mortality:incidence ratios derived from country-specific cancer registry data

3c Estimated from national mortality estimates by modelling, using mortality:incidence ratios derived from survival estimation

Appendix Table 2: ICD-10 codes for cancers based on Globocan

**Cancer**	**ICD-10 code**
Bladder	C67
Brain and CNS	C70-C72
Breast	C50
Cervix	C53
Colorectum	C18-C21
Endometrium	C54
Gallbladder	C23-C24
Hodgkin lymphoma	C81
Kidney	C64
Larynx	C32
Leukaemia	C91-95
Liver	C22
Lung	C33-C34
Melanoma of skin	C43
Multiple myeloma	C88,C90
Non-Hodkin lymphoma	C82-C86, C96
Oesophagus	C15
Oral	C00-C14
Ovary	C56
Pancreas	C25
Prostate	C61
Stomach	C16
Testis	C62
Thyroid	C73

Appendix Table 3: Number and percentage of countries where the average annual percentage change (AAPC) in cancer incidence is significantly greater in younger than older adults (and increasing AAPC in younger adults). Results show the number of countries with a statistically significant (P<0.05) AAPC difference, and the subset of those countries where the Bayes false discovery probability (BFDP) is <0.8[1], using a range of prior effect sizes for the expected magnitude of the AAPC difference. The prior probability of a true difference in AAPC was set to 0.05. BFDPs were calculated using three prior variances, corresponding to the prior beliefs that increases in AAPC from 0 to AAPCs greater than 1%, 2.5%, and 5% occur with a probability of 2.5%

		**Countries with significant (P<0.05) AAPC differences between age groups** [2]		**Countries with significant AAPC differences between age groups and BFDP <0.8**					
				**AAPC used in prior variance**					
				1%[3]		2.5%[3]		5%[3]	
Cancer	Total number of countries	n	%	n	%	n	%	n	%
Breast	42	4	10%	2	5%	2	5%	2	5%
Colorectum	42	16	38%	8	19%	11	26%	11	26%
Endometrium	36	5	14%	1	3%	1	3%	2	6%
Gallbladder	29	1	3%	0	0%	0	0%	0	0%
Kidney	40	10	25%	2	5%	6	15%	5	12%
Leukaemia	40	4	10%	1	2%	1	2%	1	2%
Liver	35	1	3%	0	0%	0	0%	0	0%
Oesophagus	32	1	3%	0	0%	0	0%	1	3%
Oral	39	4	10%	2	5%	2	5%	2	5%
Pancreas	36	4	11%	1	3%	2	6%	2	6%
Prostate	35	10	29%	1	3%	4	11%	5	14%
Stomach	39	4	10%	1	3%	2	5%	2	5%
Thyroid	41	6	15%	1	2%	2	5%	5	12%

[1] BFDP < 0.8 was used to identify "noteworthy findings" among those with p < 0.05.

[2] Based on countries where the AAPC for younger people is greater than zero (increasing trends) and larger in magnitude than the AAPC for older people.

[3]Prior effect sizes correspond to 97.5% quantile AAPC increases of 1%, 2.5% and 5% from an AAPC of 0, used to derive the prior variances for BFDP calculations.

Appendix Table 4: Segment specific annual percentage change (APC) for cancer incidence trends from 2003 to 2017 by country: breast cancer

	Joinpoint trend 1			Joinpoint trend 2			Joinpoint trend 3			
Country	Years	APC	95% CI	Years	APC	95% CI	Years	APC	95% CI	AAPC
Argentina										
20-49y	2003-2005	-10.59	-28.1, 11.18	2005-2017	1.12	-0.18, 2.43	-	-	-	-0.64
50+y	2003-2017	-1.12	-2.05, -0.19	-	-	-	-	-	-	-1.12
Australia										
20-49y	2003-2017	0.81	0.52, 1.1	-	-	-	-	-	-	0.81
50+y	2003-2017	1.17	0.83, 1.51	-	-	-	-	-	-	1.17
Austria										
20-49y	2003-2014	1.25	0.38, 2.12	2014-2017	-2.67	-8.71, 3.76	-	-	-	0.39
50+y	2003-2017	-0.50	-0.97, -0.03	-	-	-	-	-	-	-0.50
Bahrain										
20-49y	2003-2017	1.38	-1.37, 4.2	-	-	-	-	-	-	1.38
50+y	2003-2017	1.71	-0.16, 3.6	-	-	-	-	-	-	1.71
Belarus										
20-49y	2003-2013	0.23	-0.84, 1.31	2013-2017	3.94	-0.49, 8.56	-	-	-	1.28
50+y	2003-2017	2.20	1.94, 2.45	-	-	-	-	-	-	2.20
Canada										
20-49y	2003-2017	0.62	0.33, 0.92	-	-	-	-	-	-	0.62
50+y	2003-2017	0.74	0.49, 0.99	-	-	-	-	-	-	0.74
Chile										
20-49y	2003-2017	0.37	-2.72, 3.56	-	-	-	-	-	-	0.37
50+y	2003-2017	1.08	-1.13, 3.34	-	-	-	-	-	-	1.08
China										
20-49y	2003-2017	1.27	0.36, 2.19	-	-	-	-	-	-	1.27
50+y	2003-2017	2.53	2.03, 3.04	-	-	-	-	-	-	2.53
Colombia										
20-49y	2003-2017	0.08	-0.74, 0.91	-	-	-	-	-	-	0.08
50+y	2003-2006	4.81	-3.67, 14.03	2006-2011	-3.16	-8.19, 2.15	2011-2017	4.43	1.49, 7.45	1.73
Croatia										
20-49y	2003-2007	-1.15	-5.98, 3.92	2007-2017	2.72	1.46, 3.99	-	-	-	1.60
50+y	2003-2017	0.93	0.11, 1.76	-	-	-	-	-	-	0.93
Cyprus										
20-49y	2003-2017	1.17	-0.31, 2.68	-	-	-	-	-	-	1.17
50+y	2003-2017	0.87	0, 1.75	-	-	-	-	-	-	0.87
Czechia										
20-49y	2003-2017	2.03	1.62, 2.45	-	-	-	-	-	-	2.03
50+y	2003-2017	0.18	-0.35, 0.72	-	-	-	-	-	-	0.18
Denmark										
20-49y	2003-2017	1.10	0.58, 1.61	-	-	-	-	-	-	1.10
50+y	2003-2009	5.25	2.35, 8.23	2009-2017	-2.96	-4.69, -1.19	-	-	-	0.48
Ecuador										
20-49y	2003-2015	0.18	-2, 2.41	2015-2017	21.02	-16.65, 75.71	-	-	-	2.92
50+y	2003-2017	1.46	-0.1, 3.05	-	-	-	-	-	-	1.46
Estonia										
20-49y	2003-2017	1.91	0.81, 3.03	-	-	-	-	-	-	1.91

Appendix Table 4: Segment specific annual percentage change (APC) for cancer incidence trends from 2003 to 2017 by country: breast cancer

	Joinpoint trend 1			Joinpoint trend 2			Joinpoint trend 3			
Country	Years	APC	95% CI	Years	APC	95% CI	Years	APC	95% CI	AAPC
50+y	2003-2017	1.23	0.42, 2.05	-	-	-	-	-	-	1.23
Finland										
20-49y	2003-2015	-0.01	-0.68, 0.67	2015-2017	5.77	-5.64, 18.57	-	-	-	0.80
50+y	2003-2010	2.06	1.07, 3.06	2010-2017	0.50	-0.48, 1.49	-	-	-	1.28
France										
20-49y	2003-2017	0.57	0.17, 0.98	-	-	-	-	-	-	0.57
50+y	2003-2006	-2.90	-6.76, 1.12	2006-2017	0.09	-0.45, 0.64	-	-	-	-0.56
Germany										
20-49y	2003-2005	6.69	-3.77, 18.28	2005-2017	0.59	-0.02, 1.21	-	-	-	1.44
50+y	2003-2005	-5.48	-15.42, 5.64	2005-2008	7.96	-3.4, 20.65	2008-2017	-2.37	-3.36, -1.38	-0.71
Iceland										
20-49y	2003-2017	0.28	-0.97, 1.54	-	-	-	-	-	-	0.28
50+y	2003-2017	-0.54	-1.66, 0.59	-	-	-	-	-	-	-0.54
India										
20-49y	2003-2017	0.27	-0.64, 1.18	-	-	-	-	-	-	0.27
50+y	2003-2017	2.64	2.06, 3.23	-	-	-	-	-	-	2.64
Ireland										
20-49y	2003-2017	0.50	-0.13, 1.13	-	-	-	-	-	-	0.50
50+y	2003-2005	-4.20	-10.69, 2.77	2005-2008	6.31	-0.9, 14.03	2008-2017	-0.44	-1.07, 0.2	0.42
Israel										
20-49y	2003-2009	-1.55	-4, 0.96	2009-2015	2.69	-0.68, 6.16	2015-2017	-7.00	-19.87, 7.94	-0.57
50+y	2003-2017	0.09	-0.4, 0.58	-	-	-	-	-	-	0.09
Italy										
20-49y	2003-2006	-3.07	-7.36, 1.43	2006-2017	1.71	1.09, 2.33	-	-	-	0.67
50+y	2003-2017	0.93	0.46, 1.4	-	-	-	-	-	-	0.93
Kuwait										
20-49y	2003-2017	1.55	0.23, 2.88	-	-	-	-	-	-	1.55
50+y	2003-2017	2.86	1.63, 4.1	-	-	-	-	-	-	2.86
Latvia										
20-49y	2003-2017	2.81	1.83, 3.79	-	-	-	-	-	-	2.81
50+y	2003-2017	1.98	1.12, 2.84	-	-	-	-	-	-	1.98
Lithuania										
20-49y	2003-2005	-5.69	-20.05, 11.26	2005-2017	2.15	1.16, 3.16	-	-	-	1.00
50+y	2003-2015	2.23	1.3, 3.16	2015-2017	-6.58	-19.86, 8.89	-	-	-	0.92
Malta										
20-49y	2003-2005	17.48	-11.87, 56.62	2005-2017	-0.31	-1.99, 1.4	-	-	-	2.06
50+y	2003-2011	2.47	0.57, 4.41	2011-2017	-1.29	-4.11, 1.61	-	-	-	0.84
New Zealand										
20-49y	2003-2014	1.49	0.58, 2.41	2014-2017	-3.48	-9.74, 3.22	-	-	-	0.40
50+y	2003-2017	0.55	0.23, 0.88	-	-	-	-	-	-	0.55
Norway										
20-49y	2003-2017	0.97	0.29, 1.65	-	-	-	-	-	-	0.97
50+y	2003-2009	-1.86	-3.45, -0.25	2009-2017	2.07	1, 3.14	-	-	-	0.36
Philippines										
20-49y	2003-2017	-1.80	-2.8, -0.79	-	-	-	-	-	-	-1.80

Appendix Table 4: Segment specific annual percentage change (APC) for cancer incidence trends from 2003 to 2017 by country: breast cancer

	Joinpoint trend 1			Joinpoint trend 2			Joinpoint trend 3			
Country	Years	APC	95% CI	Years	APC	95% CI	Years	APC	95% CI	AAPC
50+y	2003-2017	-0.57	-1.42, 0.28	-	-	-	-	-	-	-0.57
Poland										
20-49y	2003-2017	1.19	0.01, 2.39	-	-	-	-	-	-	1.19
50+y	2003-2017	1.88	1.07, 2.69	-	-	-	-	-	-	1.88
Qatar										
20-49y	2003-2017	-2.61	-5.09, -0.07	-	-	-	-	-	-	-2.61
50+y	2003-2017	7.15	2.56, 11.95	-	-	-	-	-	-	7.15
Republic of Korea										
20-49y	2003-2017	4.31	4.03, 4.59	-	-	-	-	-	-	4.31
50+y	2003-2011	6.94	6.12, 7.77	2011-2014	2.97	-4.04, 10.48	2014-2017	7.01	3.31, 10.85	6.09
Slovenia										
20-49y	2003-2011	-0.11	-1.55, 1.34	2011-2017	4.36	2.04, 6.72	-	-	-	1.78
50+y	2003-2017	0.52	0.09, 0.96	-	-	-	-	-	-	0.52
Sweden										
20-49y	2003-2017	0.35	-0.08, 0.77	-	-	-	-	-	-	0.35
50+y	2003-2007	-1.82	-4.09, 0.52	2007-2012	1.93	-0.44, 4.35	2012-2017	-0.87	-2.51, 0.78	-0.15
Switzerland										
20-49y	2003-2017	0.09	-0.7, 0.9	-	-	-	-	-	-	0.09
50+y	2003-2017	-0.48	-0.8, -0.16	-	-	-	-	-	-	-0.48
Thailand										
20-49y	2003-2017	1.74	0.9, 2.58	-	-	-	-	-	-	1.74
50+y	2003-2010	3.15	1.87, 4.44	2010-2015	6.62	3.52, 9.82	2015-2017	-4.05	-12.61, 5.34	3.30
The Netherlands										
20-49y	2003-2017	0.51	0.17, 0.86	-	-	-	-	-	-	0.51
50+y	2003-2012	1.22	0.81, 1.63	2012-2017	-1.03	-2, -0.04	-	-	-	0.41
Türkiye										
20-49y	2003-2017	2.05	1.46, 2.64	-	-	-	-	-	-	2.05
50+y	2003-2017	1.76	1.06, 2.47	-	-	-	-	-	-	1.76
UK										
20-49y	2003-2007	-0.36	-1.93, 1.24	2007-2013	2.18	1.04, 3.34	2013-2017	-0.44	-2.01, 1.16	0.70
50+y	2003-2011	-0.12	-0.36, 0.11	2011-2014	2.32	0.15, 4.54	2014-2017	-1.41	-2.46, -0.35	0.12
USA										
20-49y	2003-2017	0.30	0.09, 0.51	-	-	-	-	-	-	0.30
50+y	2003-2017	-0.09	-0.2, 0.02	-	-	-	-	-	-	-0.09
Uganda										
20-49y	2003-2017	1.14	-0.59, 2.89	-	-	-	-	-	-	1.14
50+y	2003-2017	-0.52	-3.19, 2.21	-	-	-	-	-	-	-0.52

Appendix Table 5: Segment specific annual percentage change (APC) for cancer incidence trends from 2003 to 2017 by country: colorectum cancer

	Joinpoint trend 1			Joinpoint trend 2			Joinpoint trend 3			
Country	Years	APC	95% CI	Years	APC	95% CI	Years	APC	95% CI	AAPC
Argentina										
20-49y	2003-2017	3.10	0.9, 5.35	-	-	-	-	-	-	3.10
50+y	2003-2017	0.18	-0.72, 1.08	-	-	-	-	-	-	0.18
Australia										
20-49y	2003-2017	3.11	2.68, 3.55	-	-	-	-	-	-	3.11
50+y	2003-2007	0.65	-1.34, 2.68	2007-2017	-2.39	-2.87, -1.91	-	-	-	-1.53
Austria										
20-49y	2003-2017	0.45	-0.72, 1.63	-	-	-	-	-	-	0.45
50+y	2003-2017	-2.72	-2.99, -2.45	-	-	-	-	-	-	-2.72
Bahrain										
20-49y	2003-2017	-1.65	-4.88, 1.69	-	-	-	-	-	-	-1.65
50+y	2003-2017	3.39	1.69, 5.13	-	-	-	-	-	-	3.39
Belarus										
20-49y	2003-2015	0.95	-0.2, 2.11	2015-2017	12.34	-7.45, 36.36	-	-	-	2.50
50+y	2003-2017	2.34	2.09, 2.6	-	-	-	-	-	-	2.34
Canada										
20-49y	2003-2017	2.88	2.41, 3.35	-	-	-	-	-	-	2.88
50+y	2003-2015	-0.87	-1.28, -0.47	2015-2017	-5.66	-11.97, 1.1	-	-	-	-1.57
Chile										
20-49y	2003-2017	4.25	-0.73, 9.47	-	-	-	-	-	-	4.25
50+y	2003-2017	1.74	-0.32, 3.84	-	-	-	-	-	-	1.74
China										
20-49y	2003-2010	-3.14	-5.08, -1.16	2010-2013	9.80	-5.62, 27.73	2013-2017	-1.37	-5.98, 3.46	0.01
50+y	2003-2017	0.86	0.58, 1.15	-	-	-	-	-	-	0.86
Colombia										
20-49y	2003-2006	11.04	1.19, 21.85	2006-2017	0.00	-1.25, 1.26	-	-	-	2.27
50+y	2003-2017	1.25	0.59, 1.92	-	-	-	-	-	-	1.25
Croatia										
20-49y	2003-2017	0.75	-0.51, 2.03	-	-	-	-	-	-	0.75
50+y	2003-2017	1.22	0.71, 1.73	-	-	-	-	-	-	1.22
Cyprus										
20-49y	2003-2017	-0.19	-2.56, 2.23	-	-	-	-	-	-	-0.19
50+y	2003-2007	7.38	-0.27, 15.62	2007-2017	-1.46	-3.24, 0.35	-	-	-	0.99
Czechia										
20-49y	2003-2013	-1.56	-2.87, -0.23	2013-2017	3.88	-1.63, 9.69	-	-	-	-0.04
50+y	2003-2014	-1.30	-1.72, -0.87	2014-2017	-5.53	-8.51, -2.45	-	-	-	-2.22
Denmark										
20-49y	2003-2017	1.98	1.06, 2.92	-	-	-	-	-	-	1.98
50+y	2003-2017	1.30	0.57, 2.03	-	-	-	-	-	-	1.30
Ecuador										
20-49y	2003-2017	2.16	0.05, 4.3	-	-	-	-	-	-	2.16
50+y	2003-2017	2.63	1.34, 3.94	-	-	-	-	-	-	2.63
Estonia										
20-49y	2003-2017	2.55	-0.67, 5.86	-	-	-	-	-	-	2.55

Appendix Table 5: Segment specific annual percentage change (APC) for cancer incidence trends from 2003 to 2017 by country: colorectum cancer

	Joinpoint trend 1			Joinpoint trend 2			Joinpoint trend 3			
Country	Years	APC	95% CI	Years	APC	95% CI	Years	APC	95% CI	AAPC
50+y	2003-2017	1.16	0.57, 1.76	-	-	-	-	-	-	1.16
Finland										
20-49y	2003-2017	1.52	0.23, 2.83	-	-	-	-	-	-	1.52
50+y	2003-2017	0.71	0.43, 0.98	-	-	-	-	-	-	0.71
France										
20-49y	2003-2008	-1.23	-3.77, 1.38	2008-2017	2.23	1.14, 3.32	-	-	-	0.98
50+y	2003-2017	-0.35	-1.01, 0.31	-	-	-	-	-	-	-0.35
Germany										
20-49y	2003-2015	2.73	1.97, 3.5	2015-2017	-6.66	-17.7, 5.87	-	-	-	1.34
50+y	2003-2017	-1.75	-2.04, -1.45	-	-	-	-	-	-	-1.75
Iceland										
20-49y	2003-2017	4.27	1.02, 7.62	-	-	-	-	-	-	4.27
50+y	2003-2017	0.21	-1.14, 1.58	-	-	-	-	-	-	0.21
India										
20-49y	2003-2017	1.38	0.13, 2.64	-	-	-	-	-	-	1.38
50+y	2003-2007	5.18	1.72, 8.76	2007-2017	2.43	1.59, 3.28	-	-	-	3.21
Ireland										
20-49y	2003-2017	1.57	0.51, 2.64	-	-	-	-	-	-	1.57
50+y	2003-2007	1.46	-0.73, 3.71	2007-2017	-1.36	-1.89, -0.82	-	-	-	-0.56
Israel										
20-49y	2003-2017	0.64	-0.33, 1.61	-	-	-	-	-	-	0.64
50+y	2003-2017	-3.53	-4.07, -3	-	-	-	-	-	-	-3.53
Italy										
20-49y	2003-2017	0.61	-0.45, 1.68	-	-	-	-	-	-	0.61
50+y	2003-2010	1.21	-0.42, 2.86	2010-2017	-3.07	-4.63, -1.49	-	-	-	-0.96
Kuwait										
20-49y	2003-2005	51.05	-13.96, 165.17	2005-2017	-0.33	-3.59, 3.04	-	-	-	5.77
50+y	2003-2017	1.80	0.21, 3.42	-	-	-	-	-	-	1.80
Latvia										
20-49y	2003-2017	0.50	-1.58, 2.61	-	-	-	-	-	-	0.50
50+y	2003-2009	-1.45	-2.62, -0.27	2009-2012	5.98	-1.23, 13.73	2012-2017	-2.07	-3.6, -0.51	-0.13
Lithuania										
20-49y	2003-2005	-9.41	-28.26, 14.38	2005-2017	1.12	-0.27, 2.52	-	-	-	-0.46
50+y	2003-2010	1.67	-0.02, 3.39	2010-2017	-1.62	-3.26, 0.04	-	-	-	0.01
Malta										
20-49y	2003-2006	-20.69	-45.35, 15.1	2006-2017	6.99	1.75, 12.5	-	-	-	0.34
50+y	2003-2017	0.45	-0.92, 1.84	-	-	-	-	-	-	0.45
New Zealand										
20-49y	2003-2011	1.06	-1.01, 3.18	2011-2017	5.92	2.57, 9.38	-	-	-	3.12
50+y	2003-2014	-1.56	-1.93, -1.19	2014-2017	-4.15	-6.78, -1.46	-	-	-	-2.12
Norway										
20-49y	2003-2010	-0.80	-2.64, 1.08	2010-2013	10.69	-3.79, 27.36	2013-2017	0.03	-4.31, 4.57	1.80
50+y	2003-2015	0.55	0.36, 0.74	2015-2017	-1.53	-4.68, 1.73	-	-	-	0.25
Philippines										
20-49y	2003-2017	-1.52	-2.91, -0.12	-	-	-	-	-	-	-1.52

Appendix Table 5: Segment specific annual percentage change (APC) for cancer incidence trends from 2003 to 2017 by country: colorectum cancer

	Joinpoint trend 1			Joinpoint trend 2			Joinpoint trend 3			
Country	Years	APC	95% CI	Years	APC	95% CI	Years	APC	95% CI	AAPC
50+y	2003-2011	-3.43	-5.83, -0.97	2011-2017	1.50	-2.39, 5.54	-	-	-	-1.35
Poland										
20-49y	2003-2017	0.73	-1.18, 2.69	-	-	-	-	-	-	0.73
50+y	2003-2011	2.03	0.47, 3.62	2011-2017	-2.01	-4.32, 0.36	-	-	-	0.28
Qatar										
20-49y	2003-2017	0.24	-4.49, 5.2	-	-	-	-	-	-	0.24
50+y	2003-2017	1.11	-0.44, 2.67	-	-	-	-	-	-	1.11
Republic of Korea										
20-49y	2003-2012	4.32	3.47, 5.18	2012-2015	-7.71	-15.62, 0.94	2015-2017	1.50	-7.2, 11.02	1.22
50+y	2003-2010	5.04	4.04, 6.04	2010-2017	-3.81	-4.72, -2.88	-	-	-	0.52
Slovenia										
20-49y	2003-2017	0.80	-0.54, 2.15	-	-	-	-	-	-	0.80
50+y	2003-2010	3.05	0.48, 5.69	2010-2017	-5.58	-7.94, -3.17	-	-	-	-1.36
Sweden										
20-49y	2003-2017	2.73	1.95, 3.5	-	-	-	-	-	-	2.73
50+y	2003-2007	1.38	0.39, 2.38	2007-2014	-0.55	-1.07, -0.02	2014-2017	1.99	0.42, 3.59	0.54
Switzerland										
20-49y	2003-2017	2.29	0.2, 4.42	-	-	-	-	-	-	2.29
50+y	2003-2017	-1.16	-1.81, -0.5	-	-	-	-	-	-	-1.16
Thailand										
20-49y	2003-2017	2.56	1.36, 3.78	-	-	-	-	-	-	2.56
50+y	2003-2017	4.08	3.28, 4.89	-	-	-	-	-	-	4.08
The Netherlands										
20-49y	2003-2006	-1.23	-4.07, 1.7	2006-2009	4.90	-1.05, 11.2	2009-2017	0.96	0.32, 1.61	1.32
50+y	2003-2017	1.29	0.65, 1.94	-	-	-	-	-	-	1.29
Türkiye										
20-49y	2003-2017	2.07	1.22, 2.94	-	-	-	-	-	-	2.07
50+y	2003-2017	1.78	1.41, 2.14	-	-	-	-	-	-	1.78
UK										
20-49y	2003-2017	3.26	2.82, 3.71	-	-	-	-	-	-	3.26
50+y	2003-2011	1.35	1.08, 1.62	2011-2014	-3.00	-5.34, -0.6	2014-2017	-0.61	-1.82, 0.61	-0.02
USA										
20-49y	2003-2013	1.57	1.09, 2.06	2013-2017	4.68	2.67, 6.72	-	-	-	2.45
50+y	2003-2008	-2.48	-3.7, -1.25	2008-2011	-4.85	-10.05, 0.65	2011-2017	-1.04	-1.97, -0.09	-2.38
Uganda										
20-49y	2003-2017	0.80	-3.68, 5.49	-	-	-	-	-	-	0.80
50+y	2003-2017	2.75	-0.06, 5.65	-	-	-	-	-	-	2.75

Appendix Table 6: Segment specific annual percentage change (APC) for cancer incidence trends from 2003 to 2017 by country: endometrium cancer

	Joinpoint trend 1			Joinpoint trend 2			Joinpoint trend 3			
Country	Years	APC	95% CI	Years	APC	95% CI	Years	APC	95% CI	AAPC
Argentina										
20-49y	2003-2017	1.13	-2.92, 5.35	-	-	-	-	-	-	1.13
50+y	2003-2017	1.04	-0.52, 2.62	-	-	-	-	-	-	1.04
Australia										
20-49y	2003-2017	2.65	1.56, 3.75	-	-	-	-	-	-	2.65
50+y	2003-2013	1.87	1.4, 2.35	2013-2017	-0.63	-2.51, 1.29	-	-	-	1.15
Austria										
20-49y	2003-2017	-1.58	-3.52, 0.39	-	-	-	-	-	-	-1.58
50+y	2003-2017	-1.91	-2.26, -1.55	-	-	-	-	-	-	-1.91
Bahrain										
20-49y	-	-	-	-	-	-	-	-	-	-
50+y	2003-2017	5.37	-0.48, 11.57	-	-	-	-	-	-	5.37
Belarus										
20-49y	2003-2017	2.35	1.18, 3.54	-	-	-	-	-	-	2.35
50+y	2003-2017	3.31	2.92, 3.71	-	-	-	-	-	-	3.31
Canada										
20-49y	2003-2017	2.88	1.97, 3.79	-	-	-	-	-	-	2.88
50+y	2003-2008	1.68	0.57, 2.8	2008-2011	3.93	-1.06, 9.16	2011-2017	1.12	0.29, 1.97	1.92
Chile										
20-49y	-	-	-	-	-	-	-	-	-	-
50+y	2003-2017	5.49	1.76, 9.35	-	-	-	-	-	-	5.49
China										
20-49y	2003-2017	3.44	1.87, 5.04	-	-	-	-	-	-	3.44
50+y	2003-2017	1.42	0.54, 2.3	-	-	-	-	-	-	1.42
Colombia										
20-49y	2003-2017	0.74	-1.48, 3.02	-	-	-	-	-	-	0.74
50+y	2003-2017	1.55	0.72, 2.39	-	-	-	-	-	-	1.55
Croatia										
20-49y	2003-2005	25.37	-20.52, 97.76	2005-2017	1.83	-0.88, 4.61	-	-	-	4.90
50+y	2003-2017	2.49	1.73, 3.27	-	-	-	-	-	-	2.49
Cyprus										
20-49y	2003-2017	5.61	1.26, 10.15	-	-	-	-	-	-	5.61
50+y	2003-2017	1.09	-0.25, 2.44	-	-	-	-	-	-	1.09
Czechia										
20-49y	2003-2017	-0.33	-1.36, 0.72	-	-	-	-	-	-	-0.33
50+y	2003-2017	-0.33	-0.69, 0.03	-	-	-	-	-	-	-0.33
Denmark										
20-49y	2003-2017	3.23	1.11, 5.38	-	-	-	-	-	-	3.23
50+y	2003-2013	0.79	-0.14, 1.73	2013-2017	-2.50	-6.1, 1.24	-	-	-	-0.16
Ecuador										
20-49y	2003-2017	5.48	-1.94, 13.45	-	-	-	-	-	-	5.48
50+y	2003-2017	1.70	-0.77, 4.22	-	-	-	-	-	-	1.70
Estonia										
20-49y	2003-2017	2.79	0.25, 5.4	-	-	-	-	-	-	2.79

Appendix Table 6: Segment specific annual percentage change (APC) for cancer incidence trends from 2003 to 2017 by country: endometrium cancer

	Joinpoint trend 1			Joinpoint trend 2			Joinpoint trend 3			
**Country**	**Years**	**APC**	**95% CI**	**Years**	**APC**	**95% CI**	**Years**	**APC**	**95% CI**	**AAPC**
50+y	2003-2009	-0.61	-3.14, 1.98	2009-2017	2.92	1.22, 4.64	-	-	-	1.39
Finland										
20-49y	2003-2017	0.22	-1.7, 2.17	-	-	-	-	-	-	0.22
50+y	2003-2017	-1.14	-1.7, -0.59	-	-	-	-	-	-	-1.14
France										
20-49y	2003-2012	-4.16	-7.84, -0.33	2012-2017	6.25	-3.47, 16.94	-	-	-	-0.56
50+y	2003-2013	1.07	0.25, 1.9	2013-2017	-2.71	-5.9, 0.58	-	-	-	-0.03
Germany										
20-49y	2003-2017	1.57	0.22, 2.95	-	-	-	-	-	-	1.57
50+y	2003-2005	6.62	-4.87, 19.5	2005-2017	-0.78	-1.45, -0.11	-	-	-	0.24
Iceland										
20-49y	-	-	-	-	-	-	-	-	-	-
50+y	2003-2017	2.12	-1.48, 5.86	-	-	-	-	-	-	2.12
India										
20-49y	2003-2017	3.54	1.25, 5.88	-	-	-	-	-	-	3.54
50+y	2003-2011	8.36	5.62, 11.18	2011-2017	3.37	-0.66, 7.56	-	-	-	6.19
Ireland										
20-49y	2003-2017	2.06	0.38, 3.76	-	-	-	-	-	-	2.06
50+y	2003-2017	2.81	2.01, 3.61	-	-	-	-	-	-	2.81
Israel										
20-49y	2003-2017	-2.82	-4.22, -1.4	-	-	-	-	-	-	-2.82
50+y	2003-2014	0.08	-0.73, 0.89	2014-2017	-4.55	-10.09, 1.33	-	-	-	-0.93
Italy										
20-49y	2003-2017	0.45	-1.4, 2.32	-	-	-	-	-	-	0.45
50+y	2003-2017	-0.05	-1.02, 0.94	-	-	-	-	-	-	-0.05
Kuwait										
20-49y	-	-	-	-	-	-	-	-	-	-
50+y	2003-2017	5.09	2.67, 7.58	-	-	-	-	-	-	5.09
Latvia										
20-49y	2003-2017	-0.34	-3.32, 2.73	-	-	-	-	-	-	-0.34
50+y	2003-2017	-0.17	-0.92, 0.57	-	-	-	-	-	-	-0.17
Lithuania										
20-49y	2003-2017	2.99	1.33, 4.67	-	-	-	-	-	-	2.99
50+y	2003-2017	1.36	0.71, 2.02	-	-	-	-	-	-	1.36
Malta										
20-49y	2003-2005	67.83	-62.33, 647.63	2005-2012	-17.78	-36.13, 5.84	2012-2017	36.55	-2.23, 90.71	9.13
50+y	2003-2017	0.48	-1.63, 2.63	-	-	-	-	-	-	0.48
New Zealand										
20-49y	2003-2013	6.65	4.04, 9.32	2013-2017	-4.12	-13.31, 6.03	-	-	-	3.45
50+y	2003-2017	1.51	0.9, 2.13	-	-	-	-	-	-	1.51
Norway										
20-49y	2003-2017	-0.91	-2.22, 0.42	-	-	-	-	-	-	-0.91
50+y	2003-2017	-0.74	-1.55, 0.07	-	-	-	-	-	-	-0.74
Philippines										
20-49y	2003-2006	3.89	-9.57, 19.35	2006-2012	-7.33	-12.91, -1.4	2012-2017	6.30	-0.1, 13.11	-0.26

Appendix Table 6: Segment specific annual percentage change (APC) for cancer incidence trends from 2003 to 2017 by country: endometrium cancer

	Joinpoint trend 1			Joinpoint trend 2			Joinpoint trend 3			
Country	Years	APC	95% CI	Years	APC	95% CI	Years	APC	95% CI	AAPC
50+y	2003-2009	-5.14	-8.46, -1.71	2009-2017	2.81	0.47, 5.2	-	-	-	-0.68
Poland										
20-49y	2003-2017	-0.31	-3.83, 3.33	-	-	-	-	-	-	-0.31
50+y	2003-2008	-1.79	-5.04, 1.58	2008-2017	2.72	1.32, 4.14	-	-	-	1.09
Qatar										
20-49y	-	-	-	-	-	-	-	-	-	-
50+y	-	-	-	-	-	-	-	-	-	-
Republic of Korea										
20-49y	2003-2017	5.11	4.42, 5.8	-	-	-	-	-	-	5.11
50+y	2003-2017	4.87	4.25, 5.5	-	-	-	-	-	-	4.87
Slovenia										
20-49y	2003-2017	0.77	-1.24, 2.82	-	-	-	-	-	-	0.77
50+y	2003-2008	-2.59	-6.73, 1.74	2008-2017	1.30	-0.48, 3.12	-	-	-	-0.10
Sweden										
20-49y	2003-2017	1.74	0.45, 3.05	-	-	-	-	-	-	1.74
50+y	2003-2009	-0.02	-1.15, 1.13	2009-2017	-2.14	-2.85, -1.41	-	-	-	-1.23
Switzerland										
20-49y	2003-2017	1.39	-1.73, 4.61	-	-	-	-	-	-	1.39
50+y	2003-2011	-1.59	-3.84, 0.7	2011-2017	2.47	-1.13, 6.2	-	-	-	0.13
Thailand										
20-49y	2003-2017	4.58	1.84, 7.39	-	-	-	-	-	-	4.58
50+y	2003-2017	3.83	2.42, 5.27	-	-	-	-	-	-	3.83
The Netherlands										
20-49y	2003-2017	-0.88	-2.05, 0.3	-	-	-	-	-	-	-0.88
50+y	2003-2017	-0.76	-1.2, -0.33	-	-	-	-	-	-	-0.76
Türkiye										
20-49y	2003-2017	1.36	0.19, 2.54	-	-	-	-	-	-	1.36
50+y	2003-2017	2.20	1.42, 2.98	-	-	-	-	-	-	2.20
UK										
20-49y	2003-2017	2.54	1.93, 3.15	-	-	-	-	-	-	2.54
50+y	2003-2010	3.01	2.12, 3.91	2010-2017	0.37	-0.5, 1.24	-	-	-	1.68
USA										
20-49y	2003-2017	1.40	0.8, 2	-	-	-	-	-	-	1.40
50+y	2003-2010	1.77	1.02, 2.52	2010-2017	0.64	-0.1, 1.38	-	-	-	1.20
Uganda										
20-49y	-	-	-	-	-	-	-	-	-	-
50+y	2003-2017	2.50	-3.61, 8.98	-	-	-	-	-	-	2.50

Appendix Table 7: Segment specific annual percentage change (APC) for cancer incidence trends from 2003 to 2017 by country: gallbladder cancer

	Joinpoint trend 1			Joinpoint trend 2			Joinpoint trend 3			
**Country**	**Years**	**APC**	**95% CI**	**Years**	**APC**	**95% CI**	**Years**	**APC**	**95% CI**	**AAPC**
Argentina										
20-49y	-	-	-	-	-	-	-	-	-	-
50+y	2003-2010	0.82	-4.52, 6.46	2010-2017	-6.65	-11.6, -1.43	-	-	-	-2.99
Australia										
20-49y	2003-2017	0.85	-1.11, 2.85	-	-	-	-	-	-	0.85
50+y	2003-2013	0.57	-0.36, 1.51	2013-2017	4.55	0.68, 8.57	-	-	-	1.69
Austria										
20-49y	2003-2017	2.01	-1.75, 5.9	-	-	-	-	-	-	2.01
50+y	2003-2017	-0.58	-1.34, 0.19	-	-	-	-	-	-	-0.58
Bahrain										
20-49y	-	-	-	-	-	-	-	-	-	-
50+y	-	-	-	-	-	-	-	-	-	-
Belarus										
20-49y	2003-2017	2.38	-1.5, 6.42	-	-	-	-	-	-	2.38
50+y	2003-2005	12.15	-4.51, 31.71	2005-2011	-1.42	-4.9, 2.19	2011-2017	4.05	1.26, 6.92	2.76
Canada										
20-49y	2003-2005	20.98	-14.37, 70.93	2005-2017	2.97	0.89, 5.1	-	-	-	5.37
50+y	2003-2005	-4.98	-16.88, 8.63	2005-2017	2.26	1.45, 3.07	-	-	-	1.19
Chile										
20-49y	2003-2017	-5.58	-9.92, -1.03	-	-	-	-	-	-	-5.58
50+y	2003-2009	-7.80	-13.85, -1.32	2009-2017	-0.37	-4.64, 4.09	-	-	-	-3.62
China										
20-49y	2003-2011	-6.73	-12.08, -1.05	2011-2017	10.17	0.52, 20.74	-	-	-	0.17
50+y	2003-2017	-1.66	-2.28, -1.03	-	-	-	-	-	-	-1.66
Colombia										
20-49y	2003-2017	0.06	-3.05, 3.26	-	-	-	-	-	-	0.06
50+y	2003-2017	-1.56	-3.41, 0.34	-	-	-	-	-	-	-1.56
Croatia										
20-49y	2003-2017	-1.51	-8.05, 5.49	-	-	-	-	-	-	-1.51
50+y	2003-2017	-0.88	-1.9, 0.16	-	-	-	-	-	-	-0.88
Cyprus										
20-49y	-	-	-	-	-	-	-	-	-	-
50+y	2003-2017	1.32	-1.05, 3.75	-	-	-	-	-	-	1.32
Czechia										
20-49y	2003-2012	-9.87	-15.14, -4.27	2012-2017	7.11	-7.58, 24.14	-	-	-	-4.14
50+y	2003-2017	-3.53	-4.01, -3.04	-	-	-	-	-	-	-3.53
Denmark										
20-49y	2003-2017	1.77	-1.82, 5.49	-	-	-	-	-	-	1.77
50+y	2003-2007	7.91	-0.3, 16.8	2007-2017	-0.60	-2.52, 1.36	-	-	-	1.76
Ecuador										
20-49y	2003-2017	-1.59	-6.18, 3.24	-	-	-	-	-	-	-1.59
50+y	2003-2017	-1.74	-4.22, 0.8	-	-	-	-	-	-	-1.74
Estonia										
20-49y	-	-	-	-	-	-	-	-	-	-

Appendix Table 7: Segment specific annual percentage change (APC) for cancer incidence trends from 2003 to 2017 by country: gallbladder cancer

	Joinpoint trend 1			Joinpoint trend 2			Joinpoint trend 3			
Country	Years	APC	95% CI	Years	APC	95% CI	Years	APC	95% CI	AAPC
50+y	2003-2017	2.27	0.05, 4.54	-	-	-	-	-	-	2.27
Finland										
20-49y	2003-2008	-16.87	-34.62, 5.7	2008-2017	11.30	0.91, 22.77	-	-	-	0.29
50+y	2003-2017	0.03	-0.97, 1.05	-	-	-	-	-	-	0.03
France										
20-49y	2003-2017	-3.15	-7.82, 1.74	-	-	-	-	-	-	-3.15
50+y	2003-2017	0.26	-0.5, 1.02	-	-	-	-	-	-	0.26
Germany										
20-49y	2003-2008	11.61	-6.97, 33.9	2008-2017	-10.09	-16.53, -3.15	-	-	-	-2.87
50+y	2003-2017	-0.16	-1.3, 0.99	-	-	-	-	-	-	-0.16
Iceland										
20-49y	-	-	-	-	-	-	-	-	-	-
50+y	2003-2017	0.21	-5.43, 6.19	-	-	-	-	-	-	0.21
India										
20-49y	2003-2017	1.77	-0.44, 4.03	-	-	-	-	-	-	1.77
50+y	2003-2017	3.43	2.25, 4.63	-	-	-	-	-	-	3.43
Ireland										
20-49y	-	-	-	-	-	-	-	-	-	-
50+y	2003-2017	1.54	0.29, 2.82	-	-	-	-	-	-	1.54
Israel										
20-49y	2003-2017	-0.98	-6.13, 4.45	-	-	-	-	-	-	-0.98
50+y	2003-2009	-4.82	-10.1, 0.76	2009-2017	3.09	-0.64, 6.95	-	-	-	-0.38
Italy										
20-49y	2003-2012	-13.80	-20.93, -6.02	2012-2017	15.47	-6.56, 42.68	-	-	-	-4.31
50+y	2003-2009	-4.64	-7.31, -1.89	2009-2015	0.83	-2.89, 4.69	2015-2017	-11.59	-25.26, 4.59	-3.38
Kuwait										
20-49y	-	-	-	-	-	-	-	-	-	-
50+y	2003-2017	-1.83	-7.1, 3.74	-	-	-	-	-	-	-1.83
Latvia										
20-49y	-	-	-	-	-	-	-	-	-	-
50+y	2003-2017	-2.47	-4.47, -0.41	-	-	-	-	-	-	-2.47
Lithuania										
20-49y	-	-	-	-	-	-	-	-	-	-
50+y	2003-2010	-6.00	-9.09, -2.81	2010-2014	7.81	-4.85, 22.16	2014-2017	-12.08	-22.41, -0.38	-3.64
Malta										
20-49y	-	-	-	-	-	-	-	-	-	-
50+y	2003-2017	-2.04	-7.08, 3.29	-	-	-	-	-	-	-2.04
New Zealand										
20-49y	2003-2017	4.80	-0.37, 10.25	-	-	-	-	-	-	4.80
50+y	2003-2006	-19.66	-30.9, -6.58	2006-2009	18.35	-12.47, 60.02	2009-2017	-0.49	-3.71, 2.84	-1.35
Norway										
20-49y	2003-2017	-0.35	-4.07, 3.51	-	-	-	-	-	-	-0.35
50+y	2003-2014	6.26	3.4, 9.2	2014-2017	-12.30	-28.37, 7.37	-	-	-	1.98
Philippines										
20-49y	2003-2017	0.98	-4.62, 6.91	-	-	-	-	-	-	0.98

Appendix Table 7: Segment specific annual percentage change (APC) for cancer incidence trends from 2003 to 2017 by country: gallbladder cancer

	Joinpoint trend 1			Joinpoint trend 2			Joinpoint trend 3			
Country	Years	APC	95% CI	Years	APC	95% CI	Years	APC	95% CI	AAPC
50+y	2003-2005	11.45	-17.71, 50.94	2005-2009	-10.70	-23.27, 3.92	2009-2017	6.99	3.5, 10.59	2.20
Poland										
20-49y	-	-	-	-	-	-	-	-	-	-
50+y	2003-2017	-3.49	-5.71, -1.22	-	-	-	-	-	-	-3.49
Qatar										
20-49y	-	-	-	-	-	-	-	-	-	-
50+y	2003-2017	-0.60	-8, 7.4	-	-	-	-	-	-	-0.60
Republic of Korea										
20-49y	2003-2017	-2.52	-3.07, -1.96	-	-	-	-	-	-	-2.52
50+y	2003-2017	-0.03	-0.32, 0.26	-	-	-	-	-	-	-0.03
Slovenia										
20-49y	2003-2005	142.03	-25.29, 684.09	2005-2017	-8.31	-14.47, -1.71	-	-	-	5.33
50+y	2003-2005	23.67	3.01, 48.48	2005-2010	-3.10	-8.54, 2.67	2010-2017	1.96	-0.5, 4.48	2.92
Sweden										
20-49y	2003-2017	3.39	0.12, 6.77	-	-	-	-	-	-	3.39
50+y	2003-2008	-2.93	-7.79, 2.19	2008-2017	2.45	0.32, 4.62	-	-	-	0.49
Switzerland										
20-49y	2003-2017	5.13	0.87, 9.57	-	-	-	-	-	-	5.13
50+y	2003-2017	-1.38	-2.99, 0.27	-	-	-	-	-	-	-1.38
Thailand										
20-49y	2003-2017	0.49	-2.15, 3.2	-	-	-	-	-	-	0.49
50+y	2003-2017	3.08	2.26, 3.91	-	-	-	-	-	-	3.08
The Netherlands										
20-49y	2003-2017	1.65	-0.3, 3.64	-	-	-	-	-	-	1.65
50+y	2003-2017	1.74	0.96, 2.53	-	-	-	-	-	-	1.74
Türkiye										
20-49y	2003-2017	-0.35	-4.9, 4.42	-	-	-	-	-	-	-0.35
50+y	2003-2017	-0.66	-1.67, 0.36	-	-	-	-	-	-	-0.66
UK										
20-49y	2003-2017	2.60	1.46, 3.76	-	-	-	-	-	-	2.60
50+y	2003-2010	4.50	2.93, 6.09	2010-2017	1.01	-0.51, 2.55	-	-	-	2.74
USA										
20-49y	2003-2017	1.09	-0.22, 2.42	-	-	-	-	-	-	1.09
50+y	2003-2017	-0.02	-0.31, 0.27	-	-	-	-	-	-	-0.02
Uganda										
20-49y	-	-	-	-	-	-	-	-	-	-
50+y	-	-	-	-	-	-	-	-	-	-

Appendix Table 8: Segment specific annual percentage change (APC) for cancer incidence trends from 2003 to 2017 by country: kidney cancer

	Joinpoint trend 1			Joinpoint trend 2			Joinpoint trend 3			
Country	Years	APC	95% CI	Years	APC	95% CI	Years	APC	95% CI	AAPC
Argentina										
20-49y	2003-2006	-17.63	-36.58, 6.98	2006-2017	7.44	3.71, 11.29	-	-	-	1.49
50+y	2003-2007	-5.33	-11.04, 0.74	2007-2013	6.54	1.96, 11.33	2013-2017	-4.80	-10.54, 1.3	-0.26
Australia										
20-49y	2003-2007	7.95	2.35, 13.87	2007-2017	1.93	0.6, 3.28	-	-	-	3.62
50+y	2003-2017	1.76	1.42, 2.11	-	-	-	-	-	-	1.76
Austria										
20-49y	2003-2017	0.42	-0.78, 1.62	-	-	-	-	-	-	0.42
50+y	2003-2017	-1.24	-1.66, -0.83	-	-	-	-	-	-	-1.24
Bahrain										
20-49y	2003-2017	2.44	-6.08, 11.72	-	-	-	-	-	-	2.44
50+y	2003-2007	-24.13	-46.93, 8.47	2007-2017	6.72	-2.27, 16.54	-	-	-	-3.19
Belarus										
20-49y	2003-2017	2.55	1.59, 3.52	-	-	-	-	-	-	2.55
50+y	2003-2009	4.25	2.44, 6.1	2009-2017	1.78	0.63, 2.94	-	-	-	2.83
Canada										
20-49y	2003-2017	3.22	2.43, 4.01	-	-	-	-	-	-	3.22
50+y	2003-2017	1.93	1.48, 2.38	-	-	-	-	-	-	1.93
Chile										
20-49y	2003-2017	2.51	-3.25, 8.62	-	-	-	-	-	-	2.51
50+y	2003-2017	-0.24	-2.39, 1.95	-	-	-	-	-	-	-0.24
China										
20-49y	2003-2017	2.43	0.22, 4.69	-	-	-	-	-	-	2.43
50+y	2003-2012	3.48	2.19, 4.78	2012-2017	-0.56	-3.57, 2.53	-	-	-	2.02
Colombia										
20-49y	2003-2017	4.27	0.9, 7.76	-	-	-	-	-	-	4.27
50+y	2003-2017	3.66	2.59, 4.74	-	-	-	-	-	-	3.66
Croatia										
20-49y	2003-2017	1.97	0.81, 3.14	-	-	-	-	-	-	1.97
50+y	2003-2017	2.56	1.78, 3.34	-	-	-	-	-	-	2.56
Cyprus										
20-49y	2003-2005	-57.82	-88.17, 50.38	2005-2009	63.35	-13.49, 208.45	2009-2017	-8.66	-20.49, 4.93	-3.43
50+y	2003-2017	3.70	1.54, 5.91	-	-	-	-	-	-	3.70
Czechia										
20-49y	2003-2009	-2.95	-5.55, -0.27	2009-2017	1.46	-0.3, 3.25	-	-	-	-0.45
50+y	2003-2014	0.06	-0.49, 0.62	2014-2017	-2.55	-6.48, 1.54	-	-	-	-0.51
Denmark										
20-49y	2003-2017	4.77	2.94, 6.64	-	-	-	-	-	-	4.77
50+y	2003-2017	3.06	2.38, 3.74	-	-	-	-	-	-	3.06
Ecuador										
20-49y	2003-2009	-11.73	-24.15, 2.74	2009-2012	51.27	-38.35, 271.16	2012-2017	-8.95	-25.51, 11.28	0.17
50+y	2003-2017	4.06	1.42, 6.76	-	-	-	-	-	-	4.06
Estonia										
20-49y	2003-2013	2.16	-2.49, 7.03	2013-2017	-12.40	-27.5, 5.85	-	-	-	-2.23

Appendix Table 8: Segment specific annual percentage change (APC) for cancer incidence trends from 2003 to 2017 by country: kidney cancer

	Joinpoint trend 1			Joinpoint trend 2			Joinpoint trend 3			
Country	Years	APC	95% CI	Years	APC	95% CI	Years	APC	95% CI	AAPC
50+y	2003-2011	2.75	1.52, 4.01	2011-2015	-3.46	-8.67, 2.05	2015-2017	7.95	-3.39, 20.61	1.65
Finland										
20-49y	2003-2017	1.54	0.02, 3.1	-	-	-	-	-	-	1.54
50+y	2003-2017	0.80	0.29, 1.32	-	-	-	-	-	-	0.80
France										
20-49y	2003-2017	2.62	1.34, 3.91	-	-	-	-	-	-	2.62
50+y	2003-2017	1.43	1.03, 1.84	-	-	-	-	-	-	1.43
Germany										
20-49y	2003-2011	-0.77	-2.57, 1.07	2011-2015	8.78	0.01, 18.31	2015-2017	-12.10	-25.7, 3.99	0.12
50+y	2003-2017	0.01	-0.43, 0.45	-	-	-	-	-	-	0.01
Iceland										
20-49y	2003-2017	-0.59	-7.86, 7.25	-	-	-	-	-	-	-0.59
50+y	2003-2017	0.14	-2.38, 2.73	-	-	-	-	-	-	0.14
India										
20-49y	2003-2017	3.31	1.42, 5.24	-	-	-	-	-	-	3.31
50+y	2003-2017	2.66	1.76, 3.56	-	-	-	-	-	-	2.66
Ireland										
20-49y	2003-2017	5.11	3.23, 7.03	-	-	-	-	-	-	5.11
50+y	2003-2012	2.86	1.61, 4.12	2012-2017	-0.49	-3.41, 2.53	-	-	-	1.65
Israel										
20-49y	2003-2017	0.13	-1.42, 1.69	-	-	-	-	-	-	0.13
50+y	2003-2006	3.96	-3.51, 12.01	2006-2017	-2.75	-3.72, -1.76	-	-	-	-1.35
Italy										
20-49y	2003-2017	2.04	0.6, 3.5	-	-	-	-	-	-	2.04
50+y	2003-2017	2.09	1.37, 2.82	-	-	-	-	-	-	2.09
Kuwait										
20-49y	2003-2017	5.45	-1.21, 12.56	-	-	-	-	-	-	5.45
50+y	2003-2017	1.48	-2.06, 5.14	-	-	-	-	-	-	1.48
Latvia										
20-49y	2003-2017	2.85	-0.36, 6.17	-	-	-	-	-	-	2.85
50+y	2003-2017	2.24	1.26, 3.22	-	-	-	-	-	-	2.24
Lithuania										
20-49y	2003-2017	0.44	-1.75, 2.68	-	-	-	-	-	-	0.44
50+y	2003-2017	0.07	-0.82, 0.97	-	-	-	-	-	-	0.07
Malta										
20-49y	2003-2017	5.91	1.03, 11.02	-	-	-	-	-	-	5.91
50+y	2003-2006	22.96	5.72, 43	2006-2017	3.97	1.88, 6.11	-	-	-	7.78
New Zealand										
20-49y	2003-2015	2.57	0.99, 4.17	2015-2017	-11.93	-32.25, 14.47	-	-	-	0.36
50+y	2003-2017	1.18	0.24, 2.12	-	-	-	-	-	-	1.18
Norway										
20-49y	2003-2017	4.39	2.76, 6.05	-	-	-	-	-	-	4.39
50+y	2003-2017	2.22	1.64, 2.79	-	-	-	-	-	-	2.22
Philippines										
20-49y	2003-2017	-5.35	-8.25, -2.37	-	-	-	-	-	-	-5.35

Appendix Table 8: Segment specific annual percentage change (APC) for cancer incidence trends from 2003 to 2017 by country: kidney cancer

	Joinpoint trend 1			Joinpoint trend 2			Joinpoint trend 3			
Country	Years	APC	95% CI	Years	APC	95% CI	Years	APC	95% CI	AAPC
50+y	2003-2011	-2.38	-5.12, 0.44	2011-2017	2.62	-1.81, 7.25	-	-	-	-0.27
Poland										
20-49y	2003-2017	-1.76	-4.23, 0.78	-	-	-	-	-	-	-1.76
50+y	2003-2017	1.14	-0.41, 2.72	-	-	-	-	-	-	1.14
Qatar										
20-49y	-	-	-	-	-	-	-	-	-	-
50+y	2003-2017	1.97	-5.65, 10.2	-	-	-	-	-	-	1.97
Republic of Korea										
20-49y	2003-2017	5.41	4.65, 6.18	-	-	-	-	-	-	5.41
50+y	2003-2008	8.60	7.39, 9.81	2008-2017	1.71	1.24, 2.17	-	-	-	4.12
Slovenia										
20-49y	2003-2005	25.79	-27.56, 118.44	2005-2017	-3.15	-6.26, 0.06	-	-	-	0.54
50+y	2003-2010	3.95	1.46, 6.5	2010-2017	-0.81	-3.19, 1.62	-	-	-	1.54
Sweden										
20-49y	2003-2017	3.75	1.94, 5.59	-	-	-	-	-	-	3.75
50+y	2003-2017	1.63	1.03, 2.24	-	-	-	-	-	-	1.63
Switzerland										
20-49y	2003-2005	31.40	-9.68, 91.17	2005-2015	-0.57	-3.91, 2.89	2015-2017	-18.16	-43.74, 19.07	0.63
50+y	2003-2017	0.97	0.2, 1.74	-	-	-	-	-	-	0.97
Thailand										
20-49y	2003-2017	0.34	-2.85, 3.64	-	-	-	-	-	-	0.34
50+y	2003-2005	35.40	-11.3, 106.7	2005-2017	2.98	0.43, 5.59	-	-	-	7.08
The Netherlands										
20-49y	2003-2017	2.12	1.25, 3.01	-	-	-	-	-	-	2.12
50+y	2003-2007	3.88	0.67, 7.2	2007-2017	0.58	-0.19, 1.36	-	-	-	1.51
Türkiye										
20-49y	2003-2017	5.78	4.63, 6.94	-	-	-	-	-	-	5.78
50+y	2003-2017	2.83	1.71, 3.95	-	-	-	-	-	-	2.83
UK										
20-49y	2003-2005	-0.24	-10.53, 11.22	2005-2017	5.75	5.07, 6.43	-	-	-	4.87
50+y	2003-2014	3.98	3.46, 4.49	2014-2017	0.00	-3.6, 3.73	-	-	-	3.11
USA										
20-49y	2003-2017	2.21	1.56, 2.87	-	-	-	-	-	-	2.21
50+y	2003-2007	3.23	1.12, 5.38	2007-2017	-0.05	-0.56, 0.46	-	-	-	0.88
Uganda										
20-49y	2003-2017	2.72	-2.79, 8.54	-	-	-	-	-	-	2.72
50+y	-	-	-	-	-	-	-	-	-	-

Appendix Table 9: Segment specific annual percentage change (APC) for cancer incidence trends from 2003 to 2017 by country: leukaemia cancer

	Joinpoint trend 1			Joinpoint trend 2			Joinpoint trend 3			
Country	Years	APC	95% CI	Years	APC	95% CI	Years	APC	95% CI	AAPC
Argentina										
20-49y	2003-2017	-0.63	-2.92, 1.71	-	-	-	-	-	-	-0.63
50+y	2003-2017	-1.67	-3.26, -0.06	-	-	-	-	-	-	-1.67
Australia										
20-49y	2003-2017	0.95	0.26, 1.65	-	-	-	-	-	-	0.95
50+y	2003-2010	-0.33	-1.57, 0.92	2010-2017	3.34	2.07, 4.64	-	-	-	1.49
Austria										
20-49y	2003-2017	-1.57	-2.99, -0.13	-	-	-	-	-	-	-1.57
50+y	2003-2017	-1.97	-2.62, -1.32	-	-	-	-	-	-	-1.97
Bahrain										
20-49y	2003-2008	-20.31	-30.71, -8.34	2008-2011	45.27	-22.28, 171.51	2011-2017	-11.26	-20.16, -1.36	-5.09
50+y	2003-2017	-2.79	-6.76, 1.35	-	-	-	-	-	-	-2.79
Belarus										
20-49y	2003-2017	-0.42	-1.68, 0.86	-	-	-	-	-	-	-0.42
50+y	2003-2017	0.72	-0.46, 1.91	-	-	-	-	-	-	0.72
Canada										
20-49y	2003-2017	1.42	0.99, 1.86	-	-	-	-	-	-	1.42
50+y	2003-2017	1.67	0.98, 2.37	-	-	-	-	-	-	1.67
Chile										
20-49y	-	-	-	-	-	-	-	-	-	-
50+y	2003-2005	66.57	-30.86, 301.29	2005-2017	-1.17	-6.18, 4.1	-	-	-	6.48
China										
20-49y	2003-2011	-3.59	-7.05, 0.01	2011-2017	2.71	-2.95, 8.71	-	-	-	-0.93
50+y	2003-2017	-0.14	-0.74, 0.46	-	-	-	-	-	-	-0.14
Colombia										
20-49y	2003-2012	-2.72	-7.72, 2.55	2012-2017	12.38	-1.24, 27.88	-	-	-	2.43
50+y	2003-2017	1.69	-0.52, 3.94	-	-	-	-	-	-	1.69
Croatia										
20-49y	2003-2011	-6.39	-9.84, -2.81	2011-2017	9.22	3.05, 15.76	-	-	-	0.01
50+y	2003-2011	-3.48	-5.54, -1.37	2011-2017	3.64	0.23, 7.16	-	-	-	-0.49
Cyprus										
20-49y	2003-2017	1.52	-2.42, 5.63	-	-	-	-	-	-	1.52
50+y	2003-2017	-2.80	-4.79, -0.77	-	-	-	-	-	-	-2.80
Czechia										
20-49y	2003-2017	0.74	-0.38, 1.87	-	-	-	-	-	-	0.74
50+y	2003-2010	2.69	1.04, 4.36	2010-2017	-2.11	-3.68, -0.52	-	-	-	0.26
Denmark										
20-49y	2003-2017	1.21	-0.33, 2.78	-	-	-	-	-	-	1.21
50+y	2003-2017	0.64	0.14, 1.15	-	-	-	-	-	-	0.64
Ecuador										
20-49y	2003-2017	1.52	-0.57, 3.65	-	-	-	-	-	-	1.52
50+y	2003-2017	2.32	-0.04, 4.74	-	-	-	-	-	-	2.32
Estonia										
20-49y	2003-2017	2.48	-0.01, 5.03	-	-	-	-	-	-	2.48

Appendix Table 9: Segment specific annual percentage change (APC) for cancer incidence trends from 2003 to 2017 by country: leukaemia cancer

	Joinpoint trend 1			Joinpoint trend 2			Joinpoint trend 3			
Country	Years	APC	95% CI	Years	APC	95% CI	Years	APC	95% CI	AAPC
50+y	2003-2017	-0.19	-1.14, 0.76	-	-	-	-	-	-	-0.19
Finland										
20-49y	2003-2017	0.85	-1.25, 3	-	-	-	-	-	-	0.85
50+y	2003-2017	0.30	-0.65, 1.27	-	-	-	-	-	-	0.30
France										
20-49y	2003-2017	2.60	1.15, 4.06	-	-	-	-	-	-	2.60
50+y	2003-2017	0.83	0.35, 1.31	-	-	-	-	-	-	0.83
Germany										
20-49y	2003-2007	-4.74	-12.32, 3.5	2007-2017	2.75	0.67, 4.87	-	-	-	0.55
50+y	2003-2017	0.76	0.09, 1.43	-	-	-	-	-	-	0.76
Iceland										
20-49y	2003-2005	62.02	-26.73, 258.29	2005-2017	-4.15	-8.54, 0.46	-	-	-	3.32
50+y	2003-2017	-1.93	-4.99, 1.23	-	-	-	-	-	-	-1.93
India										
20-49y	2003-2017	0.78	-0.11, 1.67	-	-	-	-	-	-	0.78
50+y	2003-2015	1.56	0.28, 2.87	2015-2017	11.54	-10.1, 38.39	-	-	-	2.93
Ireland										
20-49y	2003-2017	0.42	-1.44, 2.32	-	-	-	-	-	-	0.42
50+y	2003-2017	-1.78	-2.67, -0.87	-	-	-	-	-	-	-1.78
Israel										
20-49y	2003-2017	-1.42	-3.09, 0.28	-	-	-	-	-	-	-1.42
50+y	2003-2017	-2.66	-3.44, -1.87	-	-	-	-	-	-	-2.66
Italy										
20-49y	2003-2017	-0.41	-2.39, 1.6	-	-	-	-	-	-	-0.41
50+y	2003-2017	-1.68	-2.86, -0.49	-	-	-	-	-	-	-1.68
Kuwait										
20-49y	2003-2017	1.81	-2.7, 6.52	-	-	-	-	-	-	1.81
50+y	2003-2017	-0.12	-2.89, 2.74	-	-	-	-	-	-	-0.12
Latvia										
20-49y	2003-2017	2.89	-0.32, 6.21	-	-	-	-	-	-	2.89
50+y	2003-2007	4.12	-5.84, 15.13	2007-2010	20.62	-12.24, 65.77	2010-2017	-6.97	-10.84, -2.93	1.57
Lithuania										
20-49y	2003-2017	0.78	-1.05, 2.64	-	-	-	-	-	-	0.78
50+y	2003-2006	-5.03	-14.41, 5.37	2006-2017	2.16	0.74, 3.6	-	-	-	0.57
Malta										
20-49y	2003-2008	-24.74	-40.64, -4.59	2008-2017	12.61	2.21, 24.06	-	-	-	-2.49
50+y	2003-2017	4.20	1.25, 7.24	-	-	-	-	-	-	4.20
New Zealand										
20-49y	2003-2017	-0.99	-2.41, 0.45	-	-	-	-	-	-	-0.99
50+y	2003-2006	-16.39	-20.15, -12.46	2006-2017	0.96	0.33, 1.58	-	-	-	-3.04
Norway										
20-49y	2003-2017	1.51	-0.7, 3.76	-	-	-	-	-	-	1.51
50+y	2003-2017	1.22	0.45, 2	-	-	-	-	-	-	1.22
Philippines										
20-49y	2003-2012	-5.44	-7.51, -3.33	2012-2017	1.65	-3.71, 7.3	-	-	-	-2.97

Appendix Table 9: Segment specific annual percentage change (APC) for cancer incidence trends from 2003 to 2017 by country: leukaemia cancer

	Joinpoint trend 1			Joinpoint trend 2			Joinpoint trend 3			
Country	Years	APC	95% CI	Years	APC	95% CI	Years	APC	95% CI	AAPC
50+y	2003-2017	-3.14	-4.4, -1.86	-	-	-	-	-	-	-3.14
Poland										
20-49y	2003-2017	0.94	-1.34, 3.27	-	-	-	-	-	-	0.94
50+y	2003-2017	-0.62	-1.88, 0.65	-	-	-	-	-	-	-0.62
Qatar										
20-49y	-	-	-	-	-	-	-	-	-	-
50+y	2003-2017	1.64	-6.7, 10.73	-	-	-	-	-	-	1.64
Republic of Korea										
20-49y	2003-2017	1.38	0.92, 1.85	-	-	-	-	-	-	1.38
50+y	2003-2017	1.20	0.85, 1.54	-	-	-	-	-	-	1.20
Slovenia										
20-49y	2003-2017	0.30	-2.24, 2.91	-	-	-	-	-	-	0.30
50+y	2003-2006	7.00	0.63, 13.78	2006-2017	-0.49	-1.31, 0.34	-	-	-	1.07
Sweden										
20-49y	2003-2017	0.63	-0.97, 2.25	-	-	-	-	-	-	0.63
50+y	2003-2017	0.47	-0.09, 1.04	-	-	-	-	-	-	0.47
Switzerland										
20-49y	2003-2017	0.01	-1.88, 1.92	-	-	-	-	-	-	0.01
50+y	2003-2017	-0.76	-1.76, 0.25	-	-	-	-	-	-	-0.76
Thailand										
20-49y	2003-2017	0.30	-1.07, 1.68	-	-	-	-	-	-	0.30
50+y	2003-2017	0.84	-0.32, 2.03	-	-	-	-	-	-	0.84
The Netherlands										
20-49y	2003-2017	0.41	-0.44, 1.27	-	-	-	-	-	-	0.41
50+y	2003-2012	-0.39	-1.1, 0.32	2012-2017	4.93	3.11, 6.79	-	-	-	1.48
Türkiye										
20-49y	2003-2017	0.88	-0.28, 2.05	-	-	-	-	-	-	0.88
50+y	2003-2017	0.79	0.06, 1.53	-	-	-	-	-	-	0.79
UK										
20-49y	2003-2017	1.29	0.73, 1.84	-	-	-	-	-	-	1.29
50+y	2003-2015	1.88	1.08, 2.68	2015-2017	-5.47	-17.24, 7.97	-	-	-	0.79
USA										
20-49y	2003-2017	1.60	0.95, 2.26	-	-	-	-	-	-	1.60
50+y	2003-2008	-0.46	-0.96, 0.05	2008-2011	4.41	2.08, 6.8	2011-2017	-1.17	-1.55, -0.79	0.26
Uganda										
20-49y	2003-2017	1.51	-3.38, 6.64	-	-	-	-	-	-	1.51
50+y	2003-2017	2.78	-2.79, 8.68	-	-	-	-	-	-	2.78

Appendix Table 10: Segment specific annual percentage change (APC) for cancer incidence trends from 2003 to 2017 by country: liver cancer

	Joinpoint trend 1			Joinpoint trend 2			Joinpoint trend 3			
Country	Years	APC	95% CI	Years	APC	95% CI	Years	APC	95% CI	AAPC
Argentina										
20-49y	2003-2005	146.33	-45.98, 1023.14	2005-2017	-6.15	-14.2, 2.66	-	-	-	7.72
50+y	2003-2017	4.90	3.38, 6.43	-	-	-	-	-	-	4.90
Australia										
20-49y	2003-2017	0.72	-0.39, 1.85	-	-	-	-	-	-	0.72
50+y	2003-2015	5.50	4.81, 6.2	2015-2017	-0.04	-10.55, 11.72	-	-	-	4.69
Austria										
20-49y	2003-2017	-3.05	-4.57, -1.5	-	-	-	-	-	-	-3.05
50+y	2003-2017	-0.57	-1.23, 0.09	-	-	-	-	-	-	-0.57
Bahrain										
20-49y	-	-	-	-	-	-	-	-	-	-
50+y	2003-2005	-35.89	-75.03, 64.56	2005-2017	4.49	-1.17, 10.48	-	-	-	-2.55
Belarus										
20-49y	2003-2017	2.24	-0.06, 4.59	-	-	-	-	-	-	2.24
50+y	2003-2017	2.88	1.86, 3.9	-	-	-	-	-	-	2.88
Canada										
20-49y	2003-2017	1.16	-0.24, 2.58	-	-	-	-	-	-	1.16
50+y	2003-2015	5.92	5.11, 6.74	2015-2017	-4.16	-15.85, 9.15	-	-	-	4.42
Chile										
20-49y	-	-	-	-	-	-	-	-	-	-
50+y	2003-2017	1.82	-2.13, 5.92	-	-	-	-	-	-	1.82
China										
20-49y	2003-2017	-3.03	-3.83, -2.22	-	-	-	-	-	-	-3.03
50+y	2003-2017	-1.80	-2.23, -1.38	-	-	-	-	-	-	-1.80
Colombia										
20-49y	2003-2017	-3.93	-7.96, 0.27	-	-	-	-	-	-	-3.93
50+y	2003-2017	1.23	-0.46, 2.96	-	-	-	-	-	-	1.23
Croatia										
20-49y	2003-2017	-4.30	-7.12, -1.4	-	-	-	-	-	-	-4.30
50+y	2003-2017	0.72	0.05, 1.39	-	-	-	-	-	-	0.72
Cyprus										
20-49y	-	-	-	-	-	-	-	-	-	-
50+y	2003-2017	2.46	0.46, 4.49	-	-	-	-	-	-	2.46
Czechia										
20-49y	2003-2017	-3.43	-6.45, -0.32	-	-	-	-	-	-	-3.43
50+y	2003-2017	-0.23	-0.76, 0.31	-	-	-	-	-	-	-0.23
Denmark										
20-49y	2003-2017	-0.49	-4.61, 3.81	-	-	-	-	-	-	-0.49
50+y	2003-2005	-9.08	-25.94, 11.61	2005-2014	5.54	3.2, 7.92	2014-2017	-3.56	-12.96, 6.86	1.34
Ecuador										
20-49y	2003-2017	-0.14	-8.6, 9.09	-	-	-	-	-	-	-0.14
50+y	2003-2012	5.69	3.09, 8.36	2012-2017	-2.71	-8.48, 3.42	-	-	-	2.61
Estonia										
20-49y	2003-2017	-2.85	-7.84, 2.41	-	-	-	-	-	-	-2.85

Appendix Table 10: Segment specific annual percentage change (APC) for cancer incidence trends from 2003 to 2017 by country: liver cancer

	Joinpoint trend 1			Joinpoint trend 2			Joinpoint trend 3			
Country	Years	APC	95% CI	Years	APC	95% CI	Years	APC	95% CI	AAPC
50+y	2003-2017	3.77	1.82, 5.76	-	-	-	-	-	-	3.77
Finland										
20-49y	2003-2017	0.48	-3.08, 4.17	-	-	-	-	-	-	0.48
50+y	2003-2005	13.62	-5.97, 37.3	2005-2017	0.89	-0.24, 2.02	-	-	-	2.61
France										
20-49y	2003-2006	-13.31	-30.85, 8.69	2006-2009	23.12	-21.67, 93.52	2009-2017	-9.33	-13.69, -4.74	-4.11
50+y	2003-2009	3.23	1.25, 5.25	2009-2017	0.11	-1.13, 1.38	-	-	-	1.44
Germany										
20-49y	2003-2017	0.36	-2.32, 3.12	-	-	-	-	-	-	0.36
50+y	2003-2014	2.44	1.44, 3.45	2014-2017	-5.42	-12.09, 1.74	-	-	-	0.70
Iceland										
20-49y	-	-	-	-	-	-	-	-	-	-
50+y	2003-2017	6.25	3.38, 9.2	-	-	-	-	-	-	6.25
India										
20-49y	2003-2017	1.48	-0.84, 3.85	-	-	-	-	-	-	1.48
50+y	2003-2017	3.47	2.46, 4.49	-	-	-	-	-	-	3.47
Ireland										
20-49y	2003-2012	15.13	7.38, 23.45	2012-2017	-10.79	-24.79, 5.82	-	-	-	5.11
50+y	2003-2017	4.92	3.94, 5.91	-	-	-	-	-	-	4.92
Israel										
20-49y	2003-2017	1.67	-2.01, 5.48	-	-	-	-	-	-	1.67
50+y	2003-2017	0.27	-0.96, 1.51	-	-	-	-	-	-	0.27
Italy										
20-49y	2003-2017	-1.88	-4.48, 0.78	-	-	-	-	-	-	-1.88
50+y	2003-2017	-2.19	-2.72, -1.66	-	-	-	-	-	-	-2.19
Kuwait										
20-49y	-	-	-	-	-	-	-	-	-	-
50+y	2003-2017	-0.54	-3.34, 2.34	-	-	-	-	-	-	-0.54
Latvia										
20-49y	2003-2017	1.15	-3.03, 5.52	-	-	-	-	-	-	1.15
50+y	2003-2017	2.74	1.46, 4.04	-	-	-	-	-	-	2.74
Lithuania										
20-49y	2003-2017	8.03	4.33, 11.87	-	-	-	-	-	-	8.03
50+y	2003-2017	3.87	2.56, 5.21	-	-	-	-	-	-	3.87
Malta										
20-49y	-	-	-	-	-	-	-	-	-	-
50+y	2003-2017	9.77	3, 16.98	-	-	-	-	-	-	9.77
New Zealand										
20-49y	2003-2017	-0.87	-3.14, 1.45	-	-	-	-	-	-	-0.87
50+y	2003-2017	3.40	2.34, 4.48	-	-	-	-	-	-	3.40
Norway										
20-49y	2003-2017	1.88	-2.31, 6.25	-	-	-	-	-	-	1.88
50+y	2003-2017	7.02	6.08, 7.96	-	-	-	-	-	-	7.02
Philippines										
20-49y	2003-2017	-5.31	-6.61, -3.99	-	-	-	-	-	-	-5.31

Appendix Table 10: Segment specific annual percentage change (APC) for cancer incidence trends from 2003 to 2017 by country: liver cancer

	Joinpoint trend 1			Joinpoint trend 2			Joinpoint trend 3			
Country	Years	APC	95% CI	Years	APC	95% CI	Years	APC	95% CI	AAPC
50+y	2003-2017	-2.61	-3.77, -1.43	-	-	-	-	-	-	-2.61
Poland										
20-49y	2003-2017	2.30	-3.72, 8.69	-	-	-	-	-	-	2.30
50+y	2003-2008	-8.32	-19.94, 4.99	2008-2017	8.35	2.52, 14.52	-	-	-	2.07
Qatar										
20-49y	-	-	-	-	-	-	-	-	-	-
50+y	2003-2017	-3.61	-9.15, 2.28	-	-	-	-	-	-	-3.61
Republic of Korea										
20-49y	2003-2009	-3.57	-4.6, -2.54	2009-2017	-5.51	-6.16, -4.86	-	-	-	-4.68
50+y	2003-2005	0.39	-2.21, 3.06	2005-2011	-2.11	-2.68, -1.53	2011-2017	-4.05	-4.47, -3.62	-2.59
Slovenia										
20-49y	2003-2017	-2.05	-8.14, 4.44	-	-	-	-	-	-	-2.05
50+y	2003-2017	2.32	0.97, 3.7	-	-	-	-	-	-	2.32
Sweden										
20-49y	2003-2017	2.31	-0.69, 5.4	-	-	-	-	-	-	2.31
50+y	2003-2006	-6.13	-15.49, 4.26	2006-2014	6.76	3.8, 9.8	2014-2017	-0.63	-10.54, 10.37	2.27
Switzerland										
20-49y	2003-2017	-4.67	-8.22, -0.99	-	-	-	-	-	-	-4.67
50+y	2003-2017	1.02	-0.03, 2.08	-	-	-	-	-	-	1.02
Thailand										
20-49y	2003-2017	-1.33	-2.28, -0.38	-	-	-	-	-	-	-1.33
50+y	2003-2005	-7.53	-19.98, 6.85	2005-2017	-0.23	-1.08, 0.63	-	-	-	-1.31
The Netherlands										
20-49y	2003-2017	3.63	2.05, 5.23	-	-	-	-	-	-	3.63
50+y	2003-2005	-2.72	-17.99, 15.41	2005-2017	7.53	6.45, 8.62	-	-	-	6.00
Türkiye										
20-49y	2003-2017	-3.06	-6.08, 0.05	-	-	-	-	-	-	-3.06
50+y	2003-2017	0.72	-0.63, 2.09	-	-	-	-	-	-	0.72
UK										
20-49y	2003-2017	2.89	1.61, 4.18	-	-	-	-	-	-	2.89
50+y	2003-2013	6.24	5.71, 6.77	2013-2017	1.82	-0.22, 3.9	-	-	-	4.96
USA										
20-49y	2003-2017	-2.81	-3.78, -1.82	-	-	-	-	-	-	-2.81
50+y	2003-2009	5.92	5.22, 6.63	2009-2015	2.77	1.86, 3.68	2015-2017	-2.76	-6.52, 1.16	3.29
Uganda										
20-49y	2003-2006	25.92	-8.39, 73.09	2006-2017	-5.25	-9.23, -1.09	-	-	-	0.71
50+y	2003-2017	-2.97	-5.42, -0.45	-	-	-	-	-	-	-2.97

Appendix Table 11: Segment specific annual percentage change (APC) for cancer incidence trends from 2003 to 2017 by country: oesophagus cancer

	Joinpoint trend 1			Joinpoint trend 2			Joinpoint trend 3			
**Country**	**Years**	**APC**	**95% CI**	**Years**	**APC**	**95% CI**	**Years**	**APC**	**95% CI**	**AAPC**
Argentina										
20-49y	2003-2017	-2.50	-6.96, 2.17	-	-	-	-	-	-	-2.50
50+y	2003-2017	-3.10	-4.57, -1.61	-	-	-	-	-	-	-3.10
Australia										
20-49y	2003-2017	-1.02	-2.4, 0.37	-	-	-	-	-	-	-1.02
50+y	2003-2017	-0.23	-0.58, 0.12	-	-	-	-	-	-	-0.23
Austria										
20-49y	2003-2017	-0.76	-4.74, 3.4	-	-	-	-	-	-	-0.76
50+y	2003-2017	-0.25	-0.96, 0.47	-	-	-	-	-	-	-0.25
Bahrain										
20-49y	-	-	-	-	-	-	-	-	-	-
50+y	2003-2017	-8.65	-14.94, -1.89	-	-	-	-	-	-	-8.65
Belarus										
20-49y	2003-2017	3.42	1.59, 5.29	-	-	-	-	-	-	3.42
50+y	2003-2017	1.66	0.94, 2.39	-	-	-	-	-	-	1.66
Canada										
20-49y	2003-2017	-1.27	-3, 0.5	-	-	-	-	-	-	-1.27
50+y	2003-2005	-3.57	-12.08, 5.76	2005-2009	3.61	-1.06, 8.51	2009-2017	-0.40	-1.4, 0.61	0.27
Chile										
20-49y	-	-	-	-	-	-	-	-	-	-
50+y	2003-2017	-3.66	-6.43, -0.81	-	-	-	-	-	-	-3.66
China										
20-49y	2003-2017	-5.70	-7.57, -3.79	-	-	-	-	-	-	-5.70
50+y	2003-2017	-2.79	-3.41, -2.17	-	-	-	-	-	-	-2.79
Colombia										
20-49y	-	-	-	-	-	-	-	-	-	-
50+y	2003-2010	-7.64	-11.9, -3.19	2010-2017	0.52	-4.11, 5.37	-	-	-	-3.65
Croatia										
20-49y	2003-2017	-0.84	-3.27, 1.64	-	-	-	-	-	-	-0.84
50+y	2003-2017	-1.90	-2.84, -0.96	-	-	-	-	-	-	-1.90
Cyprus										
20-49y	-	-	-	-	-	-	-	-	-	-
50+y	2003-2017	0.76	-5.86, 7.84	-	-	-	-	-	-	0.76
Czechia										
20-49y	2003-2017	-3.08	-6.4, 0.36	-	-	-	-	-	-	-3.08
50+y	2003-2013	2.57	1.2, 3.97	2013-2017	-1.73	-6.96, 3.8	-	-	-	1.33
Denmark										
20-49y	2003-2017	-4.63	-9.09, 0.04	-	-	-	-	-	-	-4.63
50+y	2003-2017	1.72	0.78, 2.67	-	-	-	-	-	-	1.72
Ecuador										
20-49y	-	-	-	-	-	-	-	-	-	-
50+y	2003-2017	-3.04	-6.78, 0.85	-	-	-	-	-	-	-3.04
Estonia										
20-49y	2003-2017	2.36	-3.19, 8.24	-	-	-	-	-	-	2.36

Appendix Table 11: Segment specific annual percentage change (APC) for cancer incidence trends from 2003 to 2017 by country: oesophagus cancer

	Joinpoint trend 1			Joinpoint trend 2			Joinpoint trend 3			
**Country**	**Years**	**APC**	**95% CI**	**Years**	**APC**	**95% CI**	**Years**	**APC**	**95% CI**	**AAPC**
50+y	2003-2017	1.90	0.78, 3.02	-	-	-	-	-	-	1.90
Finland										
20-49y	2003-2017	0.84	-5.52, 7.64	-	-	-	-	-	-	0.84
50+y	2003-2015	0.89	0.04, 1.75	2015-2017	11.25	-3.63, 28.42	-	-	-	2.31
France										
20-49y	2003-2017	-4.69	-6.88, -2.44	-	-	-	-	-	-	-4.69
50+y	2003-2017	-1.13	-1.87, -0.38	-	-	-	-	-	-	-1.13
Germany										
20-49y	2003-2017	0.65	-1.41, 2.75	-	-	-	-	-	-	0.65
50+y	2003-2017	1.06	0.25, 1.88	-	-	-	-	-	-	1.06
Iceland										
20-49y	-	-	-	-	-	-	-	-	-	-
50+y	2003-2017	1.43	-2.51, 5.53	-	-	-	-	-	-	1.43
India										
20-49y	2003-2017	-3.38	-4.82, -1.91	-	-	-	-	-	-	-3.38
50+y	2003-2017	-2.81	-3.4, -2.21	-	-	-	-	-	-	-2.81
Ireland										
20-49y	2003-2005	36.57	-3.21, 92.72	2005-2015	-5.49	-8.42, -2.48	2015-2017	16.33	-17.56, 64.15	2.61
50+y	2003-2014	-1.13	-2.05, -0.2	2014-2017	3.75	-3.2, 11.19	-	-	-	-0.10
Israel										
20-49y	2003-2017	-1.01	-5.58, 3.78	-	-	-	-	-	-	-1.01
50+y	2003-2017	-1.30	-2.51, -0.06	-	-	-	-	-	-	-1.30
Italy										
20-49y	2003-2017	1.26	-6.05, 9.15	-	-	-	-	-	-	1.26
50+y	2003-2017	-2.59	-3.97, -1.19	-	-	-	-	-	-	-2.59
Kuwait										
20-49y	-	-	-	-	-	-	-	-	-	-
50+y	2003-2017	1.55	-3.33, 6.68	-	-	-	-	-	-	1.55
Latvia										
20-49y	2003-2017	2.51	-1.48, 6.67	-	-	-	-	-	-	2.51
50+y	2003-2017	1.89	0.56, 3.24	-	-	-	-	-	-	1.89
Lithuania										
20-49y	2003-2017	0.85	-1.06, 2.81	-	-	-	-	-	-	0.85
50+y	2003-2017	1.69	0.64, 2.75	-	-	-	-	-	-	1.69
Malta										
20-49y	-	-	-	-	-	-	-	-	-	-
50+y	2003-2017	-1.50	-5.08, 2.22	-	-	-	-	-	-	-1.50
New Zealand										
20-49y	2003-2017	-0.16	-4.3, 4.16	-	-	-	-	-	-	-0.16
50+y	2003-2017	-1.27	-2.2, -0.33	-	-	-	-	-	-	-1.27
Norway										
20-49y	2003-2017	1.66	-1.73, 5.16	-	-	-	-	-	-	1.66
50+y	2003-2017	1.78	0.92, 2.65	-	-	-	-	-	-	1.78
Philippines										
20-49y	2003-2005	-47.17	-77.06, 21.66	2005-2017	-0.81	-5.58, 4.21	-	-	-	-9.34

Appendix Table 11: Segment specific annual percentage change (APC) for cancer incidence trends from 2003 to 2017 by country: oesophagus cancer

	Joinpoint trend 1			Joinpoint trend 2			Joinpoint trend 3			
Country	Years	APC	95% CI	Years	APC	95% CI	Years	APC	95% CI	AAPC
50+y	2003-2017	-2.90	-4.98, -0.77	-	-	-	-	-	-	-2.90
Poland										
20-49y	-	-	-	-	-	-	-	-	-	-
50+y	2003-2017	1.09	-0.88, 3.09	-	-	-	-	-	-	1.09
Qatar										
20-49y	-	-	-	-	-	-	-	-	-	-
50+y	-	-	-	-	-	-	-	-	-	-
Republic of Korea										
20-49y	2003-2017	-1.02	-1.98, -0.05	-	-	-	-	-	-	-1.02
50+y	2003-2017	-2.29	-2.42, -2.15	-	-	-	-	-	-	-2.29
Slovenia										
20-49y	2003-2017	-2.66	-8.6, 3.67	-	-	-	-	-	-	-2.66
50+y	2003-2017	-1.99	-3.91, -0.02	-	-	-	-	-	-	-1.99
Sweden										
20-49y	2003-2017	-0.07	-2.78, 2.71	-	-	-	-	-	-	-0.07
50+y	2003-2017	0.07	-0.88, 1.02	-	-	-	-	-	-	0.07
Switzerland										
20-49y	2003-2017	-4.93	-10.61, 1.11	-	-	-	-	-	-	-4.93
50+y	2003-2017	-0.30	-1.27, 0.68	-	-	-	-	-	-	-0.30
Thailand										
20-49y	2003-2017	6.79	3.2, 10.51	-	-	-	-	-	-	6.79
50+y	2003-2015	0.93	-0.77, 2.67	2015-2017	-12.68	-34.54, 16.48	-	-	-	-1.13
The Netherlands										
20-49y	2003-2017	-0.39	-1.84, 1.09	-	-	-	-	-	-	-0.39
50+y	2003-2017	2.02	1.51, 2.54	-	-	-	-	-	-	2.02
Türkiye										
20-49y	2003-2017	-4.77	-8.04, -1.37	-	-	-	-	-	-	-4.77
50+y	2003-2017	-2.11	-3.3, -0.91	-	-	-	-	-	-	-2.11
UK										
20-49y	2003-2017	-0.99	-1.69, -0.29	-	-	-	-	-	-	-0.99
50+y	2003-2007	1.17	0.11, 2.24	2007-2015	0.12	-0.33, 0.56	2015-2017	-1.76	-4.99, 1.57	0.14
USA										
20-49y	2003-2017	-1.25	-2.55, 0.07	-	-	-	-	-	-	-1.25
50+y	2003-2017	-0.96	-1.48, -0.44	-	-	-	-	-	-	-0.96
Uganda										
20-49y	2003-2017	1.63	-1.78, 5.16	-	-	-	-	-	-	1.63
50+y	2003-2017	1.75	-0.41, 3.96	-	-	-	-	-	-	1.75

Appendix Table 12: Segment specific annual percentage change (APC) for cancer incidence trends from 2003 to 2017 by country: oral cancer

	Joinpoint trend 1			Joinpoint trend 2			Joinpoint trend 3			
Country	Years	APC	95% CI	Years	APC	95% CI	Years	APC	95% CI	AAPC
Argentina										
20-49y	2003-2008	-18.30	-35.18, 2.99	2008-2017	8.56	-1.23, 19.33	-	-	-	-1.92
50+y	2003-2017	-1.06	-3.61, 1.56	-	-	-	-	-	-	-1.06
Australia										
20-49y	2003-2013	-0.03	-1.59, 1.56	2013-2017	-5.42	-11.29, 0.83	-	-	-	-1.60
50+y	2003-2005	-2.49	-10.46, 6.19	2005-2015	1.71	0.92, 2.51	2015-2017	-4.00	-11.85, 4.54	0.27
Austria										
20-49y	2003-2017	-2.80	-3.82, -1.77	-	-	-	-	-	-	-2.80
50+y	2003-2017	0.61	-0.14, 1.36	-	-	-	-	-	-	0.61
Bahrain										
20-49y	2003-2017	-0.52	-6.04, 5.33	-	-	-	-	-	-	-0.52
50+y	2003-2017	0.49	-7.37, 9.02	-	-	-	-	-	-	0.49
Belarus										
20-49y	2003-2010	1.86	-0.17, 3.93	2010-2017	5.65	3.55, 7.8	-	-	-	3.74
50+y	2003-2011	1.18	0.23, 2.14	2011-2017	3.16	1.66, 4.67	-	-	-	2.02
Canada										
20-49y	2003-2017	0.15	-0.45, 0.76	-	-	-	-	-	-	0.15
50+y	2003-2017	1.84	1.37, 2.31	-	-	-	-	-	-	1.84
Chile										
20-49y	-	-	-	-	-	-	-	-	-	-
50+y	2003-2017	-1.78	-6.42, 3.1	-	-	-	-	-	-	-1.78
China										
20-49y	2003-2017	0.73	-0.02, 1.47	-	-	-	-	-	-	0.73
50+y	2003-2008	-3.14	-6.44, 0.27	2008-2017	1.69	0.26, 3.14	-	-	-	-0.06
Colombia										
20-49y	2003-2017	-0.67	-2.65, 1.36	-	-	-	-	-	-	-0.67
50+y	2003-2012	-2.04	-3.76, -0.29	2012-2017	3.14	-1.24, 7.72	-	-	-	-0.22
Croatia										
20-49y	2003-2017	-5.11	-6.19, -4.02	-	-	-	-	-	-	-5.11
50+y	2003-2005	-7.40	-15.33, 1.27	2005-2017	-0.66	-1.18, -0.13	-	-	-	-1.65
Cyprus										
20-49y	2003-2017	4.71	-0.21, 9.87	-	-	-	-	-	-	4.71
50+y	2003-2017	3.12	1.14, 5.14	-	-	-	-	-	-	3.12
Czechia										
20-49y	2003-2010	-3.52	-5.97, -0.99	2010-2017	1.37	-1.21, 4.02	-	-	-	-1.10
50+y	2003-2017	1.94	1.59, 2.29	-	-	-	-	-	-	1.94
Denmark										
20-49y	2003-2017	-1.81	-2.83, -0.79	-	-	-	-	-	-	-1.81
50+y	2003-2012	3.23	1.83, 4.65	2012-2017	-0.46	-3.73, 2.92	-	-	-	1.90
Ecuador										
20-49y	2003-2010	13.78	-0.11, 29.6	2010-2017	-5.25	-16.82, 7.93	-	-	-	3.83
50+y	2003-2017	0.62	-2.99, 4.36	-	-	-	-	-	-	0.62
Estonia										
20-49y	2003-2012	-2.78	-7.27, 1.93	2012-2017	14.21	1.72, 28.23	-	-	-	2.98

Appendix Table 12: Segment specific annual percentage change (APC) for cancer incidence trends from 2003 to 2017 by country: oral cancer

	Joinpoint trend 1			Joinpoint trend 2			Joinpoint trend 3			
Country	Years	APC	95% CI	Years	APC	95% CI	Years	APC	95% CI	AAPC
50+y	2003-2017	1.22	0.09, 2.37	-	-	-	-	-	-	1.22
Finland										
20-49y	2003-2017	0.82	-1.29, 2.97	-	-	-	-	-	-	0.82
50+y	2003-2017	1.89	1.47, 2.31	-	-	-	-	-	-	1.89
France										
20-49y	2003-2017	-5.05	-5.91, -4.19	-	-	-	-	-	-	-5.05
50+y	2003-2017	-0.75	-1.22, -0.29	-	-	-	-	-	-	-0.75
Germany										
20-49y	2003-2014	-6.46	-7.23, -5.69	2014-2017	7.36	1.01, 14.1	-	-	-	-3.66
50+y	2003-2011	1.38	0.06, 2.72	2011-2017	-1.63	-3.61, 0.39	-	-	-	0.08
Iceland										
20-49y	-	-	-	-	-	-	-	-	-	-
50+y	2003-2017	-0.72	-3.42, 2.06	-	-	-	-	-	-	-0.72
India										
20-49y	2003-2017	2.32	1.36, 3.28	-	-	-	-	-	-	2.32
50+y	2003-2017	0.13	-0.32, 0.58	-	-	-	-	-	-	0.13
Ireland										
20-49y	2003-2017	2.60	0.56, 4.68	-	-	-	-	-	-	2.60
50+y	2003-2017	2.48	1.69, 3.29	-	-	-	-	-	-	2.48
Israel										
20-49y	2003-2017	-1.36	-2.9, 0.2	-	-	-	-	-	-	-1.36
50+y	2003-2017	0.39	-0.52, 1.32	-	-	-	-	-	-	0.39
Italy										
20-49y	2003-2017	-3.00	-5.39, -0.55	-	-	-	-	-	-	-3.00
50+y	2003-2017	-1.73	-2.64, -0.82	-	-	-	-	-	-	-1.73
Kuwait										
20-49y	2003-2017	-3.92	-8.92, 1.35	-	-	-	-	-	-	-3.92
50+y	2003-2012	6.43	1.48, 11.63	2012-2017	-13.43	-22.97, -2.71	-	-	-	-1.14
Latvia										
20-49y	2003-2017	4.14	1.8, 6.53	-	-	-	-	-	-	4.14
50+y	2003-2008	-2.04	-6.38, 2.5	2008-2017	3.61	1.71, 5.54	-	-	-	1.55
Lithuania										
20-49y	2003-2008	-4.06	-8.02, 0.08	2008-2017	6.97	5.14, 8.83	-	-	-	2.89
50+y	2003-2013	-1.61	-3.67, 0.49	2013-2017	6.30	-2.44, 15.82	-	-	-	0.59
Malta										
20-49y	2003-2017	-0.21	-5.37, 5.24	-	-	-	-	-	-	-0.21
50+y	2003-2005	24.54	-9.26, 70.95	2005-2017	-1.48	-3.31, 0.38	-	-	-	1.88
New Zealand										
20-49y	2003-2017	0.40	-0.67, 1.48	-	-	-	-	-	-	0.40
50+y	2003-2017	2.49	1.49, 3.5	-	-	-	-	-	-	2.49
Norway										
20-49y	2003-2017	1.98	0.31, 3.68	-	-	-	-	-	-	1.98
50+y	2003-2017	1.86	1.3, 2.42	-	-	-	-	-	-	1.86
Philippines										
20-49y	2003-2017	-3.30	-5.03, -1.54	-	-	-	-	-	-	-3.30

Appendix Table 12: Segment specific annual percentage change (APC) for cancer incidence trends from 2003 to 2017 by country: oral cancer

	Joinpoint trend 1			Joinpoint trend 2			Joinpoint trend 3			
Country	Years	APC	95% CI	Years	APC	95% CI	Years	APC	95% CI	AAPC
50+y	2003-2017	-3.68	-5.27, -2.07	-	-	-	-	-	-	-3.68
Poland										
20-49y	2003-2015	4.06	1.44, 6.74	2015-2017	-32.79	-56.31, 3.39	-	-	-	-2.24
50+y	2003-2017	2.23	1.1, 3.36	-	-	-	-	-	-	2.23
Qatar										
20-49y	-	-	-	-	-	-	-	-	-	-
50+y	-	-	-	-	-	-	-	-	-	-
Republic of Korea										
20-49y	2003-2017	1.97	1.52, 2.43	-	-	-	-	-	-	1.97
50+y	2003-2017	0.63	0.38, 0.87	-	-	-	-	-	-	0.63
Slovenia										
20-49y	2003-2017	-2.04	-3.73, -0.31	-	-	-	-	-	-	-2.04
50+y	2003-2017	-0.27	-1.03, 0.5	-	-	-	-	-	-	-0.27
Sweden										
20-49y	2003-2017	0.73	-0.25, 1.72	-	-	-	-	-	-	0.73
50+y	2003-2017	2.31	1.84, 2.79	-	-	-	-	-	-	2.31
Switzerland										
20-49y	2003-2017	-4.28	-6.59, -1.91	-	-	-	-	-	-	-4.28
50+y	2003-2005	-4.14	-12.76, 5.32	2005-2008	4.22	-5.14, 14.51	2008-2017	-1.90	-2.73, -1.05	-0.94
Thailand										
20-49y	2003-2017	2.17	0.99, 3.35	-	-	-	-	-	-	2.17
50+y	2003-2011	-3.36	-4.74, -1.97	2011-2014	7.84	-5.43, 22.97	2014-2017	-5.41	-11.42, 1.01	-1.52
The Netherlands										
20-49y	2003-2017	-2.32	-3.48, -1.14	-	-	-	-	-	-	-2.32
50+y	2003-2014	1.11	0.66, 1.56	2014-2017	-2.07	-5.28, 1.24	-	-	-	0.42
Türkiye										
20-49y	2003-2017	-0.49	-1.86, 0.89	-	-	-	-	-	-	-0.49
50+y	2003-2017	-1.04	-2.24, 0.18	-	-	-	-	-	-	-1.04
UK										
20-49y	2003-2012	2.91	2.17, 3.66	2012-2017	-0.03	-1.79, 1.76	-	-	-	1.85
50+y	2003-2013	3.84	3.34, 4.34	2013-2017	0.86	-1.1, 2.86	-	-	-	2.98
USA										
20-49y	2003-2017	-0.42	-1.01, 0.17	-	-	-	-	-	-	-0.42
50+y	2003-2017	0.88	0.72, 1.04	-	-	-	-	-	-	0.88
Uganda										
20-49y	2003-2017	4.77	0.18, 9.58	-	-	-	-	-	-	4.77
50+y	2003-2017	-0.67	-4.6, 3.41	-	-	-	-	-	-	-0.67

Appendix Table 13: Segment specific annual percentage change (APC) for cancer incidence trends from 2003 to 2017 by country: pancreas cancer

	Joinpoint trend 1			Joinpoint trend 2			Joinpoint trend 3			
Country	Years	APC	95% CI	Years	APC	95% CI	Years	APC	95% CI	AAPC
Argentina										
20-49y	2003-2017	0.90	-2.56, 4.48	-	-	-	-	-	-	0.90
50+y	2003-2005	18.43	-2.87, 44.41	2005-2017	0.04	-1.12, 1.22	-	-	-	2.49
Australia										
20-49y	2003-2017	3.14	2.02, 4.27	-	-	-	-	-	-	3.14
50+y	2003-2017	1.18	0.81, 1.55	-	-	-	-	-	-	1.18
Austria										
20-49y	2003-2017	0.09	-1.66, 1.86	-	-	-	-	-	-	0.09
50+y	2003-2017	0.96	0.58, 1.34	-	-	-	-	-	-	0.96
Bahrain										
20-49y	-	-	-	-	-	-	-	-	-	-
50+y	2003-2017	-1.66	-4.58, 1.36	-	-	-	-	-	-	-1.66
Belarus										
20-49y	2003-2017	1.01	-0.37, 2.4	-	-	-	-	-	-	1.01
50+y	2003-2017	2.49	2.01, 2.96	-	-	-	-	-	-	2.49
Canada										
20-49y	2003-2011	4.66	2.45, 6.92	2011-2017	-1.15	-4.36, 2.17	-	-	-	2.13
50+y	2003-2010	-0.64	-1.71, 0.44	2010-2013	5.88	-2.35, 14.8	2013-2017	-0.27	-2.79, 2.31	0.83
Chile										
20-49y	-	-	-	-	-	-	-	-	-	-
50+y	2003-2017	0.40	-3.71, 4.69	-	-	-	-	-	-	0.40
China										
20-49y	2003-2017	-1.25	-3.15, 0.68	-	-	-	-	-	-	-1.25
50+y	2003-2017	1.07	0.67, 1.46	-	-	-	-	-	-	1.07
Colombia										
20-49y	2003-2006	-23.49	-43.15, 2.96	2006-2017	7.53	3.3, 11.92	-	-	-	-0.04
50+y	2003-2012	0.29	-1.38, 1.98	2012-2017	5.86	1.6, 10.3	-	-	-	2.24
Croatia										
20-49y	2003-2017	-1.56	-4.02, 0.97	-	-	-	-	-	-	-1.56
50+y	2003-2017	1.50	0.95, 2.04	-	-	-	-	-	-	1.50
Cyprus										
20-49y	2003-2017	11.86	4.32, 19.94	-	-	-	-	-	-	11.86
50+y	2003-2007	11.18	-0.59, 24.35	2007-2017	1.41	-1.35, 4.24	-	-	-	4.11
Czechia										
20-49y	2003-2017	-0.16	-1.37, 1.07	-	-	-	-	-	-	-0.16
50+y	2003-2017	0.39	-0.03, 0.82	-	-	-	-	-	-	0.39
Denmark										
20-49y	2003-2017	-0.53	-2.54, 1.53	-	-	-	-	-	-	-0.53
50+y	2003-2009	1.40	-0.66, 3.5	2009-2017	-1.16	-2.46, 0.16	-	-	-	-0.07
Ecuador										
20-49y	-	-	-	-	-	-	-	-	-	-
50+y	2003-2017	1.14	-1.04, 3.36	-	-	-	-	-	-	1.14
Estonia										
20-49y	2003-2015	0.11	-2.89, 3.19	2015-2017	-26.38	-55.95, 23.05	-	-	-	-4.19

Appendix Table 13: Segment specific annual percentage change (APC) for cancer incidence trends from 2003 to 2017 by country: pancreas cancer

	Joinpoint trend 1			Joinpoint trend 2			Joinpoint trend 3			
Country	Years	APC	95% CI	Years	APC	95% CI	Years	APC	95% CI	AAPC
50+y	2003-2017	1.02	-0.12, 2.18	-	-	-	-	-	-	1.02
Finland										
20-49y	2003-2017	-1.09	-2.73, 0.57	-	-	-	-	-	-	-1.09
50+y	2003-2017	0.57	0.05, 1.1	-	-	-	-	-	-	0.57
France										
20-49y	2003-2017	3.07	1.08, 5.09	-	-	-	-	-	-	3.07
50+y	2003-2005	9.05	1.59, 17.04	2005-2012	3.20	1.98, 4.45	2012-2017	0.67	-0.92, 2.27	3.10
Germany										
20-49y	2003-2015	2.74	0.23, 5.32	2015-2017	-19.09	-46.78, 23.02	-	-	-	-0.71
50+y	2003-2017	0.82	0.35, 1.31	-	-	-	-	-	-	0.82
Iceland										
20-49y	-	-	-	-	-	-	-	-	-	-
50+y	2003-2017	0.48	-1.85, 2.86	-	-	-	-	-	-	0.48
India										
20-49y	2003-2017	1.14	-0.62, 2.94	-	-	-	-	-	-	1.14
50+y	2003-2017	3.70	2.68, 4.72	-	-	-	-	-	-	3.70
Ireland										
20-49y	2003-2017	1.93	-1.11, 5.07	-	-	-	-	-	-	1.93
50+y	2003-2006	7.40	1.11, 14.07	2006-2017	-0.64	-1.44, 0.17	-	-	-	1.03
Israel										
20-49y	2003-2017	-2.21	-3.8, -0.6	-	-	-	-	-	-	-2.21
50+y	2003-2013	1.16	0.13, 2.19	2013-2017	-2.70	-6.64, 1.4	-	-	-	0.04
Italy										
20-49y	2003-2017	0.17	-2.65, 3.07	-	-	-	-	-	-	0.17
50+y	2003-2017	1.21	0.65, 1.78	-	-	-	-	-	-	1.21
Kuwait										
20-49y	2003-2017	4.40	-3.25, 12.66	-	-	-	-	-	-	4.40
50+y	2003-2017	1.53	-1.42, 4.56	-	-	-	-	-	-	1.53
Latvia										
20-49y	2003-2017	-0.29	-3.25, 2.75	-	-	-	-	-	-	-0.29
50+y	2003-2017	1.45	0.83, 2.08	-	-	-	-	-	-	1.45
Lithuania										
20-49y	2003-2017	0.21	-2.16, 2.64	-	-	-	-	-	-	0.21
50+y	2003-2017	0.97	0.45, 1.5	-	-	-	-	-	-	0.97
Malta										
20-49y	-	-	-	-	-	-	-	-	-	-
50+y	2003-2015	3.93	0.52, 7.46	2015-2017	-21.98	-55.63, 37.2	-	-	-	-0.24
New Zealand										
20-49y	2003-2017	2.68	0.06, 5.38	-	-	-	-	-	-	2.68
50+y	2003-2017	1.18	0.52, 1.86	-	-	-	-	-	-	1.18
Norway										
20-49y	2003-2017	-2.18	-4.66, 0.36	-	-	-	-	-	-	-2.18
50+y	2003-2017	-0.07	-0.82, 0.68	-	-	-	-	-	-	-0.07
Philippines										
20-49y	2003-2017	-1.64	-4.18, 0.97	-	-	-	-	-	-	-1.64

Appendix Table 13: Segment specific annual percentage change (APC) for cancer incidence trends from 2003 to 2017 by country: pancreas cancer

	Joinpoint trend 1			Joinpoint trend 2			Joinpoint trend 3			
Country	Years	APC	95% CI	Years	APC	95% CI	Years	APC	95% CI	AAPC
50+y	2003-2009	-3.03	-7.13, 1.24	2009-2017	3.03	0.2, 5.94	-	-	-	0.39
Poland										
20-49y	2003-2017	3.14	-0.95, 7.39	-	-	-	-	-	-	3.14
50+y	2003-2017	-2.90	-4.03, -1.76	-	-	-	-	-	-	-2.90
Qatar										
20-49y	-	-	-	-	-	-	-	-	-	-
50+y	-	-	-	-	-	-	-	-	-	-
Republic of Korea										
20-49y	2003-2009	-1.05	-3.89, 1.87	2009-2017	4.44	2.5, 6.42	-	-	-	2.05
50+y	2003-2005	3.30	-0.9, 7.69	2005-2017	1.14	0.9, 1.39	-	-	-	1.45
Slovenia										
20-49y	2003-2017	-2.35	-6.43, 1.92	-	-	-	-	-	-	-2.35
50+y	2003-2007	5.18	-0.22, 10.88	2007-2017	0.12	-1.17, 1.43	-	-	-	1.54
Sweden										
20-49y	2003-2008	-3.07	-9.81, 4.18	2008-2015	8.22	2.48, 14.28	2015-2017	-11.17	-35.66, 22.64	1.15
50+y	2003-2008	-1.02	-2.79, 0.78	2008-2017	3.84	3.08, 4.61	-	-	-	2.08
Switzerland										
20-49y	2003-2017	3.89	1.19, 6.67	-	-	-	-	-	-	3.89
50+y	2003-2007	-3.10	-6.25, 0.16	2007-2017	2.53	1.7, 3.37	-	-	-	0.89
Thailand										
20-49y	2003-2017	2.21	-0.19, 4.68	-	-	-	-	-	-	2.21
50+y	2003-2017	3.52	2.23, 4.82	-	-	-	-	-	-	3.52
The Netherlands										
20-49y	2003-2017	1.90	0.38, 3.44	-	-	-	-	-	-	1.90
50+y	2003-2008	3.68	1.26, 6.15	2008-2017	1.22	0.25, 2.2	-	-	-	2.09
Türkiye										
20-49y	2003-2013	4.67	1.44, 8	2013-2017	-5.71	-16.98, 7.09	-	-	-	1.59
50+y	2003-2017	2.71	1.69, 3.74	-	-	-	-	-	-	2.71
UK										
20-49y	2003-2017	2.25	1.59, 2.92	-	-	-	-	-	-	2.25
50+y	2003-2017	1.04	0.88, 1.2	-	-	-	-	-	-	1.04
USA										
20-49y	2003-2017	1.32	0.4, 2.25	-	-	-	-	-	-	1.32
50+y	2003-2017	0.73	0.49, 0.98	-	-	-	-	-	-	0.73
Uganda										
20-49y	2003-2005	-46.78	-76.56, 20.84	2005-2008	61.89	-28.7, 267.6	2008-2017	-9.95	-16.45, -2.95	-5.28
50+y	2003-2017	0.06	-6.57, 7.16	-	-	-	-	-	-	0.06

Appendix Table 14: Segment specific annual percentage change (APC) for cancer incidence trends from 2003 to 2017 by country: prostate cancer

	Joinpoint trend 1			Joinpoint trend 2			Joinpoint trend 3			
**Country**	**Years**	**APC**	**95% CI**	**Years**	**APC**	**95% CI**	**Years**	**APC**	**95% CI**	**AAPC**
Argentina										
20-49y	2003-2017	2.34	-5.33, 10.63	-	-	-	-	-	-	2.34
50+y	2003-2017	-1.13	-2.05, -0.21	-	-	-	-	-	-	-1.13
Australia										
20-49y	2003-2009	21.29	15.88, 26.96	2009-2017	-5.19	-7.94, -2.35	-	-	-	5.37
50+y	2003-2008	6.05	3.47, 8.7	2008-2015	-4.66	-6.42, -2.87	2015-2017	1.74	-8.88, 13.61	-0.04
Austria										
20-49y	2003-2017	-4.31	-5.3, -3.32	-	-	-	-	-	-	-4.31
50+y	2003-2013	-4.68	-5.28, -4.08	2013-2017	3.81	1.19, 6.49	-	-	-	-2.33
Bahrain										
20-49y	-	-	-	-	-	-	-	-	-	-
50+y	2003-2017	1.73	-2.61, 6.26	-	-	-	-	-	-	1.73
Belarus										
20-49y	2003-2017	10.28	7.89, 12.73	-	-	-	-	-	-	10.28
50+y	2003-2014	9.56	8.65, 10.49	2014-2017	2.61	-3.58, 9.2	-	-	-	8.03
Canada										
20-49y	2003-2009	3.98	0.15, 7.96	2009-2017	-6.71	-8.94, -4.42	-	-	-	-2.27
50+y	2003-2007	2.33	-2.64, 7.55	2007-2015	-5.72	-7.68, -3.71	2015-2017	4.60	-10.62, 22.43	-2.04
Chile										
20-49y	-	-	-	-	-	-	-	-	-	-
50+y	2003-2017	-1.88	-2.67, -1.08	-	-	-	-	-	-	-1.88
China										
20-49y	2003-2017	12.49	5.3, 20.17	-	-	-	-	-	-	12.49
50+y	2003-2017	5.03	4.39, 5.67	-	-	-	-	-	-	5.03
Colombia										
20-49y	2003-2017	-4.73	-7.98, -1.36	-	-	-	-	-	-	-4.73
50+y	2003-2017	-0.57	-1.17, 0.02	-	-	-	-	-	-	-0.57
Croatia										
20-49y	2003-2017	7.49	2.67, 12.54	-	-	-	-	-	-	7.49
50+y	2003-2009	3.85	1.8, 5.94	2009-2014	-0.27	-3.92, 3.51	2014-2017	12.66	6.21, 19.49	4.16
Cyprus										
20-49y	2003-2017	-2.15	-9.03, 5.25	-	-	-	-	-	-	-2.15
50+y	2003-2005	10.76	-6.42, 31.09	2005-2017	0.04	-0.95, 1.04	-	-	-	1.50
Czechia										
20-49y	2003-2010	18.87	10.21, 28.22	2010-2017	3.05	-4.46, 11.16	-	-	-	10.68
50+y	2003-2010	5.55	3.44, 7.7	2010-2017	-0.51	-2.5, 1.52	-	-	-	2.47
Denmark										
20-49y	2003-2007	35.51	2.29, 79.53	2007-2017	0.79	-5.95, 8.02	-	-	-	9.69
50+y	2003-2008	8.93	5.23, 12.77	2008-2017	-2.23	-3.6, -0.84	-	-	-	1.62
Ecuador										
20-49y	2003-2013	20.00	11.06, 29.66	2013-2017	-21.82	-42.91, 7.06	-	-	-	6.17
50+y	2003-2017	1.55	0.3, 2.83	-	-	-	-	-	-	1.55
Estonia										
20-49y	2003-2005	126.86	-2.04, 425.36	2005-2017	6.68	1.51, 12.11	-	-	-	18.82

Appendix Table 14: Segment specific annual percentage change (APC) for cancer incidence trends from 2003 to 2017 by country: prostate cancer

	Joinpoint trend 1			Joinpoint trend 2			Joinpoint trend 3			
Country	Years	APC	95% CI	Years	APC	95% CI	Years	APC	95% CI	AAPC
50+y	2003-2011	8.79	5.94, 11.71	2011-2017	-2.77	-6.68, 1.3	-	-	-	3.67
Finland										
20-49y	2003-2017	-0.15	-2.52, 2.29	-	-	-	-	-	-	-0.15
50+y	2003-2017	-1.95	-3.01, -0.88	-	-	-	-	-	-	-1.95
France										
20-49y	2003-2007	15.00	-3.8, 37.47	2007-2017	-5.68	-9.73, -1.44	-	-	-	-0.18
50+y	2003-2005	7.15	-7.7, 24.39	2005-2013	-6.11	-7.97, -4.22	2013-2017	1.35	-3.32, 6.24	-2.21
Germany										
20-49y	2003-2017	0.52	-1.6, 2.68	-	-	-	-	-	-	0.52
50+y	2003-2017	-2.15	-2.9, -1.39	-	-	-	-	-	-	-2.15
Iceland										
20-49y	-	-	-	-	-	-	-	-	-	-
50+y	2003-2017	-3.08	-4.37, -1.77	-	-	-	-	-	-	-3.08
India										
20-49y	2003-2017	2.31	-1.16, 5.91	-	-	-	-	-	-	2.31
50+y	2003-2017	4.35	3.87, 4.83	-	-	-	-	-	-	4.35
Ireland										
20-49y	2003-2013	10.66	6.35, 15.15	2013-2017	-10.65	-23.96, 5	-	-	-	4.10
50+y	2003-2011	2.69	0.9, 4.51	2011-2017	-2.46	-5.08, 0.24	-	-	-	0.45
Israel										
20-49y	2003-2017	-5.95	-9.66, -2.08	-	-	-	-	-	-	-5.95
50+y	2003-2007	3.02	-3.55, 10.04	2007-2017	-6.23	-7.74, -4.7	-	-	-	-3.68
Italy										
20-49y	2003-2017	-0.73	-5.32, 4.08	-	-	-	-	-	-	-0.73
50+y	2003-2017	-0.38	-0.86, 0.11	-	-	-	-	-	-	-0.38
Kuwait										
20-49y	-	-	-	-	-	-	-	-	-	-
50+y	2003-2007	-6.68	-12.75, -0.18	2007-2010	22.00	-1.39, 50.93	2010-2017	-3.44	-6.15, -0.65	0.54
Latvia										
20-49y	2003-2017	9.25	3.16, 15.7	-	-	-	-	-	-	9.25
50+y	2003-2017	4.00	3.56, 4.44	-	-	-	-	-	-	4.00
Lithuania										
20-49y	2003-2007	49.73	15.56, 94.02	2007-2017	3.14	-3.23, 9.94	-	-	-	14.73
50+y	2003-2007	22.41	5.62, 41.87	2007-2017	-2.80	-6.27, 0.8	-	-	-	3.82
Malta										
20-49y	-	-	-	-	-	-	-	-	-	-
50+y	2003-2015	1.81	-0.67, 4.36	2015-2017	-16.24	-44.81, 27.13	-	-	-	-0.99
New Zealand										
20-49y	2003-2005	-27.71	-46.33, -2.62	2005-2009	22.04	5.15, 41.65	2009-2017	-3.50	-6.59, -0.31	-0.97
50+y	2003-2017	-0.62	-1.48, 0.25	-	-	-	-	-	-	-0.62
Norway										
20-49y	2003-2017	5.70	3.94, 7.5	-	-	-	-	-	-	5.70
50+y	2003-2007	4.98	0.22, 9.98	2007-2017	-0.38	-1.52, 0.76	-	-	-	1.12
Philippines										
20-49y	2003-2017	-4.62	-10.19, 1.29	-	-	-	-	-	-	-4.62

Appendix Table 14: Segment specific annual percentage change (APC) for cancer incidence trends from 2003 to 2017 by country: prostate cancer

	Joinpoint trend 1			Joinpoint trend 2			Joinpoint trend 3			
**Country**	**Years**	**APC**	**95% CI**	**Years**	**APC**	**95% CI**	**Years**	**APC**	**95% CI**	**AAPC**
50+y	2003-2006	5.74	-1.51, 13.53	2006-2009	-12.44	-24.04, 0.93	2009-2017	-0.22	-1.76, 1.34	-1.76
Poland										
20-49y	2003-2017	5.21	-3.1, 14.23	-	-	-	-	-	-	5.21
50+y	2003-2010	-2.28	-5.06, 0.58	2010-2017	10.95	7.8, 14.2	-	-	-	4.12
Qatar										
20-49y	-	-	-	-	-	-	-	-	-	-
50+y	2003-2017	6.74	1.07, 12.73	-	-	-	-	-	-	6.74
Republic of Korea										
20-49y	2003-2008	21.41	7.43, 37.22	2008-2017	0.79	-4.12, 5.96	-	-	-	7.72
50+y	2003-2009	12.62	10.25, 15.03	2009-2017	1.06	-0.31, 2.46	-	-	-	5.86
Slovenia										
20-49y	2003-2009	27.86	5.11, 55.53	2009-2017	-5.55	-16.77, 7.18	-	-	-	7.54
50+y	2003-2009	9.22	5.55, 13.01	2009-2017	-0.54	-2.71, 1.68	-	-	-	3.53
Sweden										
20-49y	2003-2017	4.14	2.07, 6.25	-	-	-	-	-	-	4.14
50+y	2003-2017	-0.68	-1.46, 0.12	-	-	-	-	-	-	-0.68
Switzerland										
20-49y	2003-2005	27.61	-11.39, 83.77	2005-2017	-0.51	-2.64, 1.65	-	-	-	3.09
50+y	2003-2011	0.19	-1.32, 1.72	2011-2014	-8.04	-19.97, 5.67	2014-2017	6.43	-0.71, 14.09	-0.36
Thailand										
20-49y	-	-	-	-	-	-	-	-	-	-
50+y	2003-2017	3.80	2.81, 4.81	-	-	-	-	-	-	3.80
The Netherlands										
20-49y	2003-2006	19.90	2.39, 40.39	2006-2017	-0.99	-3.07, 1.14	-	-	-	3.16
50+y	2003-2011	0.50	-0.89, 1.9	2011-2017	-2.59	-4.67, -0.48	-	-	-	-0.84
Türkiye										
20-49y	2003-2017	3.18	-0.63, 7.14	-	-	-	-	-	-	3.18
50+y	2003-2007	12.76	7.43, 18.36	2007-2017	-2.30	-3.45, -1.12	-	-	-	1.79
UK										
20-49y	2003-2010	8.97	6.45, 11.55	2010-2017	3.77	1.37, 6.22	-	-	-	6.33
50+y	2003-2013	1.68	1.09, 2.27	2013-2017	-0.84	-3.17, 1.54	-	-	-	0.95
USA										
20-49y	2003-2009	3.91	0.89, 7.02	2009-2017	-8.54	-10.27, -6.79	-	-	-	-3.40
50+y	2003-2009	-0.17	-2.86, 2.59	2009-2014	-8.76	-13.29, -3.98	2014-2017	4.64	-3.47, 13.43	-2.35
Uganda										
20-49y	2003-2017	-1.13	-6.57, 4.64	-	-	-	-	-	-	-1.13
50+y	2003-2012	5.08	1.75, 8.52	2012-2017	-3.13	-10.48, 4.82	-	-	-	2.07

Appendix Table 15: Segment specific annual percentage change (APC) for cancer incidence trends from 2003 to 2017 by country: stomach cancer

	Joinpoint trend 1			Joinpoint trend 2			Joinpoint trend 3			
Country	Years	APC	95% CI	Years	APC	95% CI	Years	APC	95% CI	AAPC
Argentina										
20-49y	2003-2012	2.33	-4.83, 10.03	2012-2017	-13.71	-27.76, 3.08	-	-	-	-3.71
50+y	2003-2017	-0.94	-1.97, 0.1	-	-	-	-	-	-	-0.94
Australia										
20-49y	2003-2017	0.93	-0.56, 2.44	-	-	-	-	-	-	0.93
50+y	2003-2009	-2.06	-3.23, -0.87	2009-2017	-0.11	-0.88, 0.67	-	-	-	-0.95
Austria										
20-49y	2003-2017	-1.77	-2.9, -0.63	-	-	-	-	-	-	-1.77
50+y	2003-2017	-2.97	-3.37, -2.56	-	-	-	-	-	-	-2.97
Bahrain										
20-49y	-	-	-	-	-	-	-	-	-	-
50+y	2003-2017	-4.11	-7.4, -0.7	-	-	-	-	-	-	-4.11
Belarus										
20-49y	2003-2017	-3.32	-4.1, -2.54	-	-	-	-	-	-	-3.32
50+y	2003-2005	1.20	-5, 7.8	2005-2017	-2.57	-2.93, -2.2	-	-	-	-2.04
Canada										
20-49y	2003-2017	-0.07	-1.37, 1.24	-	-	-	-	-	-	-0.07
50+y	2003-2011	-2.29	-3.14, -1.42	2011-2015	2.59	-1.45, 6.8	2015-2017	-3.34	-10.82, 4.76	-1.07
Chile										
20-49y	2003-2017	-4.01	-10.4, 2.84	-	-	-	-	-	-	-4.01
50+y	2003-2017	-1.34	-2.73, 0.08	-	-	-	-	-	-	-1.34
China										
20-49y	2003-2013	-5.84	-7.8, -3.84	2013-2017	0.44	-7.77, 9.39	-	-	-	-4.09
50+y	2003-2008	-3.74	-5.08, -2.39	2008-2017	-1.82	-2.37, -1.25	-	-	-	-2.51
Colombia										
20-49y	2003-2017	1.05	-0.16, 2.28	-	-	-	-	-	-	1.05
50+y	2003-2005	1.08	-8.82, 12.06	2005-2009	-7.69	-12.33, -2.81	2009-2017	0.08	-1.04, 1.21	-2.06
Croatia										
20-49y	2003-2017	-3.04	-4.44, -1.61	-	-	-	-	-	-	-3.04
50+y	2003-2006	-5.91	-11.89, 0.46	2006-2017	-1.90	-2.77, -1.03	-	-	-	-2.78
Cyprus										
20-49y	2003-2017	-2.78	-8.65, 3.47	-	-	-	-	-	-	-2.78
50+y	2003-2008	8.00	0.97, 15.51	2008-2017	-3.02	-5.65, -0.32	-	-	-	0.78
Czechia										
20-49y	2003-2017	-2.60	-4.01, -1.16	-	-	-	-	-	-	-2.60
50+y	2003-2017	-3.13	-3.5, -2.76	-	-	-	-	-	-	-3.13
Denmark										
20-49y	2003-2017	-1.46	-3.8, 0.93	-	-	-	-	-	-	-1.46
50+y	2003-2013	-1.23	-2.16, -0.29	2013-2017	3.29	-0.6, 7.34	-	-	-	0.04
Ecuador										
20-49y	2003-2017	0.75	-1.7, 3.26	-	-	-	-	-	-	0.75
50+y	2003-2005	17.17	-11.49, 55.11	2005-2017	-2.45	-4.06, -0.82	-	-	-	0.14
Estonia										
20-49y	2003-2017	-3.39	-5.25, -1.49	-	-	-	-	-	-	-3.39

Appendix Table 15: Segment specific annual percentage change (APC) for cancer incidence trends from 2003 to 2017 by country: stomach cancer

	Joinpoint trend 1			Joinpoint trend 2			Joinpoint trend 3			
Country	Years	APC	95% CI	Years	APC	95% CI	Years	APC	95% CI	AAPC
50+y	2003-2017	-1.94	-2.51, -1.37	-	-	-	-	-	-	-1.94
Finland										
20-49y	2003-2017	-3.15	-4.89, -1.38	-	-	-	-	-	-	-3.15
50+y	2003-2017	-3.21	-3.79, -2.64	-	-	-	-	-	-	-3.21
France										
20-49y	2003-2011	-2.68	-5.15, -0.14	2011-2017	3.95	-0.11, 8.17	-	-	-	0.11
50+y	2003-2017	-1.56	-2.11, -1.01	-	-	-	-	-	-	-1.56
Germany										
20-49y	2003-2017	-1.44	-3.31, 0.46	-	-	-	-	-	-	-1.44
50+y	2003-2017	-1.67	-2.14, -1.21	-	-	-	-	-	-	-1.67
Iceland										
20-49y	-	-	-	-	-	-	-	-	-	-
50+y	2003-2017	-5.13	-7.52, -2.68	-	-	-	-	-	-	-5.13
India										
20-49y	2003-2017	-0.97	-1.84, -0.08	-	-	-	-	-	-	-0.97
50+y	2003-2017	-0.15	-1.04, 0.75	-	-	-	-	-	-	-0.15
Ireland										
20-49y	2003-2017	-0.10	-2.02, 1.87	-	-	-	-	-	-	-0.10
50+y	2003-2015	-0.07	-0.65, 0.51	2015-2017	-13.57	-21.6, -4.72	-	-	-	-2.12
Israel										
20-49y	2003-2017	-1.72	-3.18, -0.24	-	-	-	-	-	-	-1.72
50+y	2003-2017	-2.25	-3.03, -1.46	-	-	-	-	-	-	-2.25
Italy										
20-49y	2003-2017	-3.57	-6.02, -1.06	-	-	-	-	-	-	-3.57
50+y	2003-2017	-2.77	-3.4, -2.14	-	-	-	-	-	-	-2.77
Kuwait										
20-49y	2003-2005	80.36	-12.99, 273.86	2005-2014	-6.60	-13.74, 1.13	2014-2017	43.15	-0.57, 106.1	12.44
50+y	2003-2017	2.22	-2.94, 7.66	-	-	-	-	-	-	2.22
Latvia										
20-49y	2003-2017	-0.55	-2.75, 1.7	-	-	-	-	-	-	-0.55
50+y	2003-2011	-2.91	-4.09, -1.71	2011-2017	-0.49	-2.36, 1.42	-	-	-	-1.88
Lithuania										
20-49y	2003-2005	-19.36	-43.47, 15.04	2005-2010	5.59	-5.63, 18.14	2010-2017	-4.79	-9.2, -0.16	-3.52
50+y	2003-2017	-2.36	-2.87, -1.85	-	-	-	-	-	-	-2.36
Malta										
20-49y	-	-	-	-	-	-	-	-	-	-
50+y	2003-2017	-1.38	-3.08, 0.35	-	-	-	-	-	-	-1.38
New Zealand										
20-49y	2003-2017	-2.38	-4.04, -0.69	-	-	-	-	-	-	-2.38
50+y	2003-2017	-1.48	-2.12, -0.84	-	-	-	-	-	-	-1.48
Norway										
20-49y	2003-2017	-1.08	-3.29, 1.17	-	-	-	-	-	-	-1.08
50+y	2003-2017	-2.78	-3.31, -2.24	-	-	-	-	-	-	-2.78
Philippines										
20-49y	2003-2017	-5.89	-8.23, -3.49	-	-	-	-	-	-	-5.89

Appendix Table 15: Segment specific annual percentage change (APC) for cancer incidence trends from 2003 to 2017 by country: stomach cancer

	Joinpoint trend 1			Joinpoint trend 2			Joinpoint trend 3			
Country	Years	APC	95% CI	Years	APC	95% CI	Years	APC	95% CI	AAPC
50+y	2003-2011	-8.34	-11.91, -4.62	2011-2017	-0.54	-6.48, 5.78	-	-	-	-5.07
Poland										
20-49y	2003-2017	-3.53	-7.94, 1.1	-	-	-	-	-	-	-3.53
50+y	2003-2005	-14.15	-29.73, 4.89	2005-2017	-1.69	-2.84, -0.52	-	-	-	-3.57
Qatar										
20-49y	2003-2017	1.89	-4.04, 8.19	-	-	-	-	-	-	1.89
50+y	2003-2017	-3.37	-10.24, 4.01	-	-	-	-	-	-	-3.37
Republic of Korea										
20-49y	2003-2010	-0.24	-1.16, 0.69	2010-2017	-4.75	-5.63, -3.86	-	-	-	-2.52
50+y	2003-2011	-0.08	-0.94, 0.79	2011-2017	-4.42	-5.69, -3.12	-	-	-	-1.96
Slovenia										
20-49y	2003-2006	-15.34	-32.03, 5.43	2006-2011	6.51	-7.29, 22.37	2011-2017	-13.73	-19.9, -7.08	-7.36
50+y	2003-2015	-2.04	-2.44, -1.64	2015-2017	-5.93	-12.17, 0.76	-	-	-	-2.61
Sweden										
20-49y	2003-2017	0.43	-1.78, 2.7	-	-	-	-	-	-	0.43
50+y	2003-2017	-2.31	-2.86, -1.76	-	-	-	-	-	-	-2.31
Switzerland										
20-49y	2003-2017	-0.45	-4.09, 3.32	-	-	-	-	-	-	-0.45
50+y	2003-2017	-0.82	-1.83, 0.21	-	-	-	-	-	-	-0.82
Thailand										
20-49y	2003-2017	1.94	0.23, 3.67	-	-	-	-	-	-	1.94
50+y	2003-2017	1.30	0.12, 2.5	-	-	-	-	-	-	1.30
The Netherlands										
20-49y	2003-2017	-1.62	-2.89, -0.34	-	-	-	-	-	-	-1.62
50+y	2003-2011	-2.12	-3.14, -1.08	2011-2017	-4.59	-6.14, -3.02	-	-	-	-3.18
Türkiye										
20-49y	2003-2017	-0.65	-1.87, 0.58	-	-	-	-	-	-	-0.65
50+y	2003-2007	1.01	-2.08, 4.21	2007-2017	-1.28	-2.03, -0.52	-	-	-	-0.63
UK										
20-49y	2003-2017	-0.83	-1.48, -0.17	-	-	-	-	-	-	-0.83
50+y	2003-2017	-3.52	-3.7, -3.34	-	-	-	-	-	-	-3.52
USA										
20-49y	2003-2017	0.39	-0.4, 1.19	-	-	-	-	-	-	0.39
50+y	2003-2017	-1.52	-1.8, -1.24	-	-	-	-	-	-	-1.52
Uganda										
20-49y	2003-2017	-3.04	-9.09, 3.42	-	-	-	-	-	-	-3.04
50+y	2003-2017	-3.87	-7.5, -0.1	-	-	-	-	-	-	-3.87

Appendix Table 16: Segment specific annual percentage change (APC) for cancer incidence trends from 2003 to 2017 by country: thyroid cancer

	Joinpoint trend 1			Joinpoint trend 2			Joinpoint trend 3			
Country	Years	APC	95% CI	Years	APC	95% CI	Years	APC	95% CI	AAPC
Argentina										
20-49y	2003-2017	2.35	-1.21, 6.05	-	-	-	-	-	-	2.35
50+y	2003-2017	0.92	-1.03, 2.9	-	-	-	-	-	-	0.92
Australia										
20-49y	2003-2013	4.06	3.45, 4.68	2013-2017	1.57	-0.83, 4.02	-	-	-	3.34
50+y	2003-2014	6.02	5.58, 6.46	2014-2017	0.65	-2.42, 3.82	-	-	-	4.85
Austria										
20-49y	2003-2008	11.41	6.78, 16.23	2008-2017	-1.74	-3.43, -0.02	-	-	-	2.77
50+y	2003-2008	8.34	4.53, 12.3	2008-2017	-4.29	-5.68, -2.88	-	-	-	0.04
Bahrain										
20-49y	2003-2007	-27.71	-50.05, 4.62	2007-2017	13.70	3.81, 24.53	-	-	-	-0.10
50+y	2003-2009	-12.48	-28.29, 6.82	2009-2017	9.87	-3.39, 24.94	-	-	-	-0.34
Belarus										
20-49y	2003-2014	1.19	0.42, 1.96	2014-2017	7.89	1.97, 14.16	-	-	-	2.59
50+y	2003-2017	-0.15	-0.87, 0.57	-	-	-	-	-	-	-0.15
Canada										
20-49y	2003-2014	4.78	4.15, 5.42	2014-2017	-6.88	-10.99, -2.58	-	-	-	2.16
50+y	2003-2013	6.89	5.82, 7.97	2013-2017	-4.83	-8.64, -0.85	-	-	-	3.40
Chile										
20-49y	2003-2017	10.68	5.38, 16.24	-	-	-	-	-	-	10.68
50+y	2003-2013	1.36	-4.54, 7.63	2013-2017	31.76	3.25, 68.16	-	-	-	9.25
China										
20-49y	2003-2008	15.46	9.63, 21.6	2008-2013	28.23	19.17, 37.98	2013-2017	9.25	1.53, 17.56	17.99
50+y	2003-2014	18.61	16.81, 20.44	2014-2017	3.07	-8, 15.47	-	-	-	15.09
Colombia										
20-49y	2003-2009	2.82	-1.8, 7.65	2009-2017	9.20	6.01, 12.49	-	-	-	6.42
50+y	2003-2017	7.37	5.93, 8.82	-	-	-	-	-	-	7.37
Croatia										
20-49y	2003-2017	6.62	5.54, 7.7	-	-	-	-	-	-	6.62
50+y	2003-2015	4.38	3.63, 5.14	2015-2017	-4.90	-15.86, 7.48	-	-	-	3.00
Cyprus										
20-49y	2003-2017	10.94	8.81, 13.1	-	-	-	-	-	-	10.94
50+y	2003-2017	15.71	13.11, 18.36	-	-	-	-	-	-	15.71
Czechia										
20-49y	2003-2017	5.38	4.73, 6.04	-	-	-	-	-	-	5.38
50+y	2003-2005	16.07	-3.87, 40.15	2005-2015	1.72	-0.02, 3.49	2015-2017	-6.74	-22.77, 12.6	2.38
Denmark										
20-49y	2003-2006	1.46	-7.64, 11.45	2006-2017	7.88	6.52, 9.25	-	-	-	6.47
50+y	2003-2017	7.39	5.46, 9.34	-	-	-	-	-	-	7.39
Ecuador										
20-49y	2003-2012	13.95	9.8, 18.26	2012-2017	4.82	-4.29, 14.8	-	-	-	10.60
50+y	2003-2017	10.67	8.84, 12.52	-	-	-	-	-	-	10.67
Estonia										
20-49y	2003-2017	0.30	-2.39, 3.07	-	-	-	-	-	-	0.30

Appendix Table 16: Segment specific annual percentage change (APC) for cancer incidence trends from 2003 to 2017 by country: thyroid cancer

	Joinpoint trend 1			Joinpoint trend 2			Joinpoint trend 3			
Country	Years	APC	95% CI	Years	APC	95% CI	Years	APC	95% CI	AAPC
50+y	2003-2017	-0.09	-2.27, 2.15	-	-	-	-	-	-	-0.09
Finland										
20-49y	2003-2005	-9.17	-23.49, 7.83	2005-2017	3.84	2.8, 4.9	-	-	-	1.88
50+y	2003-2005	-8.84	-25.23, 11.14	2005-2017	4.77	3.55, 6.01	-	-	-	2.71
France										
20-49y	2003-2013	4.17	2.93, 5.42	2013-2017	-4.72	-9.23, 0.02	-	-	-	1.55
50+y	2003-2009	4.97	2.3, 7.71	2009-2015	-0.04	-3.39, 3.43	2015-2017	-11.44	-23.97, 3.15	0.33
Germany										
20-49y	2003-2017	6.16	4.42, 7.93	-	-	-	-	-	-	6.16
50+y	2003-2017	3.41	2.17, 4.66	-	-	-	-	-	-	3.41
Iceland										
20-49y	2003-2017	2.09	-2.73, 7.15	-	-	-	-	-	-	2.09
50+y	2003-2017	-0.56	-5.86, 5.04	-	-	-	-	-	-	-0.56
India										
20-49y	2003-2017	1.34	0.25, 2.44	-	-	-	-	-	-	1.34
50+y	2003-2017	2.17	0.61, 3.76	-	-	-	-	-	-	2.17
Ireland										
20-49y	2003-2013	11.33	8.21, 14.54	2013-2017	-6.24	-16.46, 5.24	-	-	-	6.00
50+y	2003-2013	9.85	6.88, 12.91	2013-2017	-5.56	-15.52, 5.59	-	-	-	5.21
Israel										
20-49y	2003-2014	3.86	2.54, 5.2	2014-2017	-8.16	-16.48, 1	-	-	-	1.16
50+y	2003-2013	3.06	1.97, 4.16	2013-2017	-7.39	-11.29, -3.31	-	-	-	-0.04
Italy										
20-49y	2003-2017	1.66	0.32, 3.02	-	-	-	-	-	-	1.66
50+y	2003-2017	2.20	0.87, 3.55	-	-	-	-	-	-	2.20
Kuwait										
20-49y	2003-2007	-8.45	-14.65, -1.79	2007-2017	8.67	6.8, 10.56	-	-	-	3.47
50+y	2003-2005	65.65	-22.71, 255.06	2005-2017	3.22	-1.33, 7.98	-	-	-	10.44
Latvia										
20-49y	2003-2013	14.04	9.72, 18.53	2013-2017	0.48	-14.11, 17.56	-	-	-	9.99
50+y	2003-2008	14.83	9, 20.98	2008-2017	4.71	2.5, 6.96	-	-	-	8.22
Lithuania										
20-49y	2003-2017	1.96	0.73, 3.21	-	-	-	-	-	-	1.96
50+y	2003-2008	7.06	2.78, 11.52	2008-2017	-2.24	-3.86, -0.6	-	-	-	0.98
Malta										
20-49y	2003-2017	6.77	2.84, 10.85	-	-	-	-	-	-	6.77
50+y	2003-2017	10.47	4.83, 16.41	-	-	-	-	-	-	10.47
New Zealand										
20-49y	2003-2017	3.66	2.29, 5.06	-	-	-	-	-	-	3.66
50+y	2003-2017	2.68	1.77, 3.6	-	-	-	-	-	-	2.68
Norway										
20-49y	2003-2017	5.05	3.8, 6.31	-	-	-	-	-	-	5.05
50+y	2003-2009	1.66	-1.62, 5.05	2009-2017	6.89	4.65, 9.18	-	-	-	4.62
Philippines										
20-49y	2003-2012	-4.07	-6.59, -1.49	2012-2015	15.08	-14, 53.99	2015-2017	-8.32	-31.49, 22.67	-0.90

Appendix Table 16: Segment specific annual percentage change (APC) for cancer incidence trends from 2003 to 2017 by country: thyroid cancer

	Joinpoint trend 1			Joinpoint trend 2			Joinpoint trend 3			
Country	Years	APC	95% CI	Years	APC	95% CI	Years	APC	95% CI	AAPC
50+y	2003-2012	-3.48	-6.12, -0.76	2012-2015	15.57	-14.74, 56.66	2015-2017	-12.92	-35.75, 18.04	-1.15
Poland										
20-49y	2003-2010	3.11	-1.45, 7.88	2010-2015	19.37	7.26, 32.85	2015-2017	-4.56	-31.95, 33.85	7.45
50+y	2003-2017	2.13	0.5, 3.78	-	-	-	-	-	-	2.13
Qatar										
20-49y	2003-2012	-7.13	-16.42, 3.2	2012-2017	19.28	-7.87, 54.43	-	-	-	1.55
50+y	-	-	-	-	-	-	-	-	-	-
Republic of Korea										
20-49y	2003-2011	22.14	18.37, 26.03	2011-2017	-9.12	-13.43, -4.6	-	-	-	7.60
50+y	2003-2011	21.91	18, 25.96	2011-2017	-14.20	-18.43, -9.75	-	-	-	4.88
Slovenia										
20-49y	2003-2017	2.45	0.94, 3.99	-	-	-	-	-	-	2.45
50+y	2003-2017	2.56	1.15, 3.98	-	-	-	-	-	-	2.56
Sweden										
20-49y	2003-2017	5.50	4.47, 6.54	-	-	-	-	-	-	5.50
50+y	2003-2017	4.05	3.29, 4.82	-	-	-	-	-	-	4.05
Switzerland										
20-49y	2003-2013	7.30	4.6, 10.07	2013-2017	-4.77	-14.15, 5.62	-	-	-	3.70
50+y	2003-2017	4.45	2.52, 6.42	-	-	-	-	-	-	4.45
Thailand										
20-49y	2003-2005	-13.71	-43.53, 31.84	2005-2017	6.25	3.62, 8.95	-	-	-	3.14
50+y	2003-2017	2.73	1.26, 4.22	-	-	-	-	-	-	2.73
The Netherlands										
20-49y	2003-2017	4.63	3.77, 5.49	-	-	-	-	-	-	4.63
50+y	2003-2017	4.38	3.36, 5.4	-	-	-	-	-	-	4.38
Türkiye										
20-49y	2003-2005	57.15	28.61, 92.03	2005-2017	6.44	5.19, 7.71	-	-	-	12.53
50+y	2003-2008	17.14	11.91, 22.61	2008-2017	3.02	1.12, 4.96	-	-	-	7.86
UK										
20-49y	2003-2013	7.26	6.79, 7.73	2013-2017	3.36	1.52, 5.24	-	-	-	6.13
50+y	2003-2014	6.38	5.57, 7.2	2014-2017	2.23	-3.39, 8.18	-	-	-	5.48
USA										
20-49y	2003-2009	6.09	5.12, 7.08	2009-2015	2.03	0.8, 3.28	2015-2017	-4.48	-9.56, 0.87	2.78
50+y	2003-2009	7.46	5.79, 9.15	2009-2015	0.69	-1.38, 2.79	2015-2017	-6.48	-14.76, 2.6	2.45
Uganda										
20-49y	2003-2013	-0.55	-6.95, 6.3	2013-2017	-25.44	-43.11, -2.29	-	-	-	-8.41
50+y	2003-2017	-4.25	-10.46, 2.38	-	-	-	-	-	-	-4.25

Table 17: Number and % of eligible countries in Europe with increasing cancer rates (AAPC>0) in younger adults, and also in older adults and the number of countries with cancer rates in younger adults increasing at a faster rate than in older adults

	**Eligible countries (age 20–49y)**	**↑ age 20–49y**	**↑ age 20–49y & ↑ 50+y**	**↑ age 20–49y > 50+y**
**Cancer**	**n (%)[1]**	**n (%)**	**n (%)**	**n (%)**
Breast	22 (100)	22 (100)	16 (73)	18 (82)
Thyroid	22 (100)	22 (100)	19 (86)	17 (77)
Colorectum	22 (100)	20 (91)	11 (50)	18 (82)
Kidney	22 (100)	18 (82)	17 (77)	12 (55)
Leukaemia	22 (100)	18 (82)	12 (55)	15 (68)
Prostate	20 (100)	16 (80)	12 (60)	12 (60)
Endometrium	21 (100)	14 (67)	9 (43)	12 (57)
Gallbladder	15 (100)	9 (60)	7 (47)	6 (40)
Liver	20 (100)	11 (55)	11 (55)	4 (20)
Pancreas	20 (100)	11 (55)	10 (50)	4 (20)
Oesophagus	19 (100)	9 (47)	7 (37)	5 (26)
Oral	21 (100)	9 (43)	9 (43)	6 (29)
Stomach	20 (100)	2 (10)	0 (0)	2 (10)

[1]AAPC was not estimable if countries had 0 cases in any year.

Table 18: Number and % of eligible countries in Americas with increasing cancer rates (AAPC>0) in younger adults, and also in older adults and the number of countries with cancer rates in younger adults increasing at a faster rate than in older adults

	Eligible countries (age 20–49y)	↑ age 20–49y	↑ age 20–49y & ↑ 50+y	↑ age 20–49y > 50+y
**Cancer**	**n (%)[1]**	**n (%)**	**n (%)**	**n (%)**
Colorectum	6 (100)	6 (100)	4 (67)	5 (83)
Endometrium	5 (100)	5 (100)	5 (100)	4 (80)
Kidney	6 (100)	6 (100)	4 (67)	5 (83)
Thyroid	6 (100)	6 (100)	6 (100)	2 (33)
Breast	6 (100)	5 (83)	4 (67)	2 (33)
Leukaemia	5 (100)	4 (80)	4 (80)	2 (40)
Pancreas	4 (100)	3 (75)	3 (75)	2 (50)
Gallbladder	5 (100)	3 (60)	1 (20)	3 (60)
Stomach	6 (100)	3 (50)	1 (17)	3 (50)
Liver	5 (100)	2 (40)	2 (40)	1 (20)
Oral	5 (100)	2 (40)	2 (40)	1 (20)
Prostate	5 (100)	2 (40)	1 (20)	2 (40)
Oesophagus	3 (100)	0 (0)	0 (0)	0 (0)

[1]AAPC was not estimable if countries had 0 cases in any year.

Table 19: Number and % of eligible countries in Asia with increasing cancer rates (AAPC>0) in younger adults, and also in older adults and the number of countries with cancer rates in younger adults increasing at a faster rate than in older adults

	**Eligible countries (age 20–49y)**	**↑ age 20–49y**	**↑ age 20–49y & ↑ 50+y**	**↑ age 20–49y > 50+y**
**Cancer**	**n (%)[1]**	**n (%)**	**n (%)**	**n (%)**
Kidney	10 (100)	8 (80)	6 (60)	7 (70)
Thyroid	10 (100)	8 (80)	7 (70)	5 (50)
Endometrium	8 (100)	6 (75)	6 (75)	4 (50)
Breast	11 (100)	8 (73)	8 (73)	2 (18)
Colorectum	11 (100)	8 (73)	7 (64)	4 (36)
Pancreas	9 (100)	6 (67)	6 (67)	2 (22)
Leukaemia	10 (100)	6 (60)	4 (40)	4 (40)
Gallbladder	7 (100)	4 (57)	3 (43)	1 (14)
Prostate	7 (100)	4 (57)	4 (57)	2 (29)
Oral	10 (100)	5 (50)	3 (30)	5 (50)
Stomach	10 (100)	3 (30)	2 (20)	2 (20)
Liver	7 (100)	2 (29)	2 (29)	1 (14)
Oesophagus	7 (100)	1 (14)	0 (0)	1 (14)

[1]AAPC was not estimable if countries had 0 cases in any year.

Table 20: Number and % of eligible countries in Europe with increasing cancer rates (AAPC>0) in younger adults, and also in older adults and the number of countries with cancer rates in younger adults increasing at a faster rate than in older adults: females

	Eligible countries (age 20-49y)	↑ age 20-49y	↑ age 20-49y & ↑ 50+y	↑ age 20-49y > 50+y
**Cancer**	**n (%)**[1]	**n (%)**	**n (%)**	**n (%)**
Thyroid	41 (100)	38 (93)	35 (85)	22 (54)
Breast	42 (100)	38 (90)	30 (71)	23 (55)
Colorectum	41 (100)	37 (90)	21 (51)	32 (78)
Kidney	35 (100)	27 (77)	24 (69)	20 (57)
Pancreas	33 (100)	25 (76)	23 (70)	17 (52)
Endometrium	36 (100)	27 (75)	22 (61)	22 (61)
Leukaemia	38 (100)	22 (58)	16 (42)	13 (34)
Liver	28 (100)	16 (57)	13 (46)	6 (21)
Gallbladder	27 (100)	15 (56)	8 (30)	11 (41)
Oral	36 (100)	18 (50)	14 (39)	11 (31)
Oesophagus	15 (100)	5 (33)	4 (27)	3 (20)
Stomach	35 (100)	10 (29)	3 (9)	10 (29)

[1]AAPC was not estimable if countries had 0 cases in any year.

Table 21: Number and % of eligible countries in Europe with increasing cancer rates (AAPC>0) in younger adults, and also in older adults and the number of countries with cancer rates in younger adults increasing at a faster rate than in older adults

	**Eligible countries (age 20-49y)**	**↑ age 20-49y**	**↑ age 20-49y & ↑ 50+y**	**↑ age 20-49y > 50+y**
**Cancer**	**n countries[1]**	**n (%)**	**n (%)**	**n (%)**
Thyroid	34 (100)	34 (100)	32 (94)	22 (65)
Kidney	35 (100)	29 (83)	27 (77)	24 (69)
Colorectum	41 (100)	32 (78)	20 (49)	25 (61)
Leukaemia	39 (100)	28 (72)	22 (56)	20 (51)
Prostate	35 (100)	23 (66)	17 (49)	17 (49)
Gallbladder	22 (100)	13 (59)	13 (59)	8 (36)
Pancreas	33 (100)	19 (58)	18 (55)	10 (30)
Oral	37 (100)	17 (46)	15 (41)	12 (32)
Liver	32 (100)	12 (38)	12 (38)	2 (6)
Oesophagus	32 (100)	10 (31)	10 (31)	4 (12)
Stomach	38 (100)	5 (13)	2 (5)	5 (13)

[1]AAPC was not estimable if countries had 0 cases in any year.
